# The Association Between Lysosomal Storage Disorder Genes and Parkinson’s Disease: A Large Cohort Study in Chinese Mainland Population

**DOI:** 10.3389/fnagi.2021.749109

**Published:** 2021-11-15

**Authors:** Yu-wen Zhao, Hong-xu Pan, Zhenhua Liu, Yige Wang, Qian Zeng, Zheng-huan Fang, Teng-fei Luo, Kun Xu, Zheng Wang, Xun Zhou, Runcheng He, Bin Li, Guihu Zhao, Qian Xu, Qi-ying Sun, Xin-xiang Yan, Jie-qiong Tan, Jin-chen Li, Ji-feng Guo, Bei-sha Tang

**Affiliations:** ^1^Department of Neurology, Xiangya Hospital, Central South University, Changsha, China; ^2^National Clinical Research Center for Geriatric Disorders, Xiangya Hospital, Central South University, Changsha, China; ^3^Centre for Medical Genetics & Hunan Key Laboratory of Medical Genetics, School of Life Sciences, Central South University, Changsha, China; ^4^Department of Geriatrics, Xiangya Hospital, Central South University, Changsha, China; ^5^Key Laboratory of Hunan Province in Neurodegenerative Disorders, Central South University, Changsha, China

**Keywords:** Parkinson’s disease, lysosomal storage disorders, lysosome, GBA, rare putative damaging variants

## Abstract

**Background:** Recent years have witnessed an increasing number of studies indicating an essential role of the lysosomal dysfunction in Parkinson’s disease (PD) at the genetic, biochemical, and cellular pathway levels. In this study, we investigated the association between rare variants in lysosomal storage disorder (LSD) genes and Chinese mainland PD.

**Methods:** We explored the association between rare variants of 69 LSD genes and PD in 3,879 patients and 2,931 controls from Parkinson’s Disease & Movement Disorders Multicenter Database and Collaborative Network in China (PD-MDCNC) using next-generation sequencing, which were analyzed by using the optimized sequence kernel association test.

**Results:** We identified the significant burden of rare putative LSD gene variants in Chinese mainland patients with PD. This association was robust in familial or sporadic early-onset patients after excluding the *GBA* variants but not in sporadic late-onset patients. The burden analysis of variant sets in genes of LSD subgroups revealed a suggestive significant association between variant sets in genes of sphingolipidosis deficiency disorders and familial or sporadic early-onset patients. In contrast, variant sets in genes of sphingolipidoses, mucopolysaccharidoses, and post-translational modification defect disorders were suggestively associated with sporadic late-onset patients. Then, *SMPD1* and other four novel genes (i.e., *GUSB, CLN6, PPT1*, and *SCARB2*) were suggestively associated with sporadic early-onset or familial patients, whereas *GALNS* and *NAGA* were suggestively associated with late-onset patients.

**Conclusion:** Our findings supported the association between LSD genes and PD and revealed several novel risk genes in Chinese mainland patients with PD, which confirmed the importance of lysosomal mechanisms in PD pathogenesis. Moreover, we identified the genetic heterogeneity in early-onset and late-onset of patients with PD, which may provide valuable suggestions for the treatment.

## Introduction

As the second most common neurodegenerative disease, Parkinson’s disease (PD) is characterized by the pathological changes of the misfolding and accumulation of α-synuclein, as well as the progressive loss of dopaminergic neurons in the substantia nigra (He et al., 2018). In addition, the other currently known pathogenic molecular mechanisms of PD include autophagy-lysosomal and ubiquitin-proteasome system defects, mitochondrial dysfunction, neuroinflammation, and oxidative stress (Nguyen et al., 2019; Jankovic and Tan, 2020). Studies in recent decades supported that genetic factors play essential roles in PD (Blauwendraat et al., 2020), including the common risk variants and rare damaging mutations. Although the mechanism involved in the development of PD is not yet clear, more and more studies have indicated that PD has a strong connection with lysosomes at the genetic, biochemical, and cellular pathway levels (Robak et al., 2017; Klein and Mazzulli, 2018; Hopfner et al., 2020). In addition, some studies suggested that the lysosomal enzyme activity in biological fluids could even be considered as a biomarker for the diagnosis of synucleinopathies, such as PD (Paciotti et al., 2019; Parnetti et al., 2019).

Lysosomal storage disorders (LSDs) comprised more than 70 diseases characterized by lysosomal dysfunction (Platt et al., 2018), most of which are inherited in an autosomal recessive manner. Mutations in LSD pathogenic genes may affect the function of the encoded protein, which may lead to lysosomal dysfunction, accumulation of substrates inside the lysosome, and further cell dysfunction and cell death. Since many monogenic LSDs have been described, they could be further classified according to the biochemical types of stored substances (such as sphingolipids, mucopolysaccharides, and glycoproteins) (Platt et al., 2018).

The burden of cumulative rare variants in LSD genes was identified and validated in a large European population with PD (Robak et al., 2017), even excluding *GBA*, the established most common risk factor of PD (Sidransky et al., 2009). Heterozygous, homozygous, or compound heterozygous mutations in the *GBA* gene have been robustly confirmed as the most important genetic risk factor for PD, increasing the risk of PD by more than five times (Sidransky et al., 2009; Chahine et al., 2013). Furthermore, mutations in the *SMPD1* gene have also been reported to be associated with an increased risk of PD. *SMPD1* gene encodes the lysosomal enzyme acid sphingomyelinase (ASMase), which is a lysosomal gene involved in sphingolipid metabolism (Foo et al., 2013; Gan-Or et al., 2013; Wu et al., 2014; Li et al., 2015; Mao et al., 2017; Alcalay et al., 2019). In addition, the rare damaging variants of other LSD genes, including *CTSD*, *SLC17A5*, *ASAH1, LAMP1*, and *TMEM175*, were found to be associated with PD as well (Robak et al., 2017; Hopfner et al., 2020). Recent years have witnessed the findings of the link between LSD genes, including *PSAP* (Oji et al., 2020), *ARSA* (Lee et al., 2019), and the Mendelian inheritance form of PD. Specifically, common variants in *GUSB*, *GRN*, and *NEU1* loci may alter the susceptibility to PD (Nalls et al., 2019). Nowadays, more and more genetic studies have shown that PD may have a more complex inheritance pattern, including oligogenic and polygenic inheritance of the same or related PD pathway genes, which further strengthens the synergistic relationship between them (Lubbe et al., 2016; Smolders and Van Broeckhoven, 2020).

Overall, these results indicated an important role of lysosomal dysfunction in PD, which has also been replicated and reviewed by more and more researchers (Klein and Mazzulli, 2018; Ysselstein et al., 2019; Hopfner et al., 2020). However, previous studies have found that there was significant genetic heterogeneity among different populations. For example, p.N370S exclusively increased PD risks in European and West Asians, as were p.R120W in Japanese populations, whereas p.L444P increased the risk of PD in mainland China, as well as the other non-Ashkenazi Jewish ethnicity (Sun et al., 2010; Zhang et al., 2018). In this study, we explored the association between rare variants in LSD genes and PD in a large Chinese mainland cohort.

## Materials and Methods

### Study Participants

The patients with PD were recruited from the Xiangya Hospital (Central South University) and other cooperating hospitals of Parkinson’s Disease & Movement Disorders Multicenter Database and Collaborative Network in China (PD-MDCNC)^1^ (Zhao et al., 2020) and were diagnosed by neurologists according to the UK brain bank criteria (Hughes et al., 1992) or Movement Disorders Society clinical diagnostic criteria (Postuma et al., 2015). Matched controls who were free from neurological diseases were also recruited. The protocol used in this study was approved by the Ethics of Committee of Xiangya Hospital, Central South University; written informed consent was obtained from all subjects.

We have mined the sequencing data from two cohorts. The first one is our previous whole-exome sequencing (WES) cohort (Zhao et al., 2020), which includes familial patients or sporadic early-onset patients and ethnic-matched controls (this cohort has also expanded its sample size in the past year). The other one is our follow-up whole-genome sequencing (WGS) cohort, which included sporadic late-onset patients and sex/age/ethnic-matched controls. Early-onset is defined as the age-at-onset (AAO) ≤ 50 years old; late-onset is defined as the AAO > 50 years old. The primary demographic data of the two cohorts are listed in Supplementary Table 1.

### Genotyping and Quality Control

Subjects from the WES cohort follow these settings: library preparation with an Agilent SureSelect Human All Exon V6 Kit, sequencing on the Illumina X10 platform in a paired-end 2 × 150 bp sequencing mode, with 123-fold of average sequencing depth; and subjects from the WGS cohort follow these settings: library preparation with an Illumina TruSeq Library Preparation Kit, sequencing on the Illumina Nova platform in a paired-end 2 × 150 bp sequencing mode, with 12-fold of average sequencing depth. Then, the sequencing data were analyzed using the BWA-GATK-ANNOVAR pipeline as follows: the sequencing data were aligned to the human reference genome (hg19 version) using the Burrows-Wheeler Aligner (BWA) (Li, 2014), then variant calling was performed using the Genome Analysis Toolkit (GATK) (Van Der Auwera et al., 2013), subsequent variants were annotated using ANNOVAR (Wang et al., 2010) and Varcards^2^ (Li et al., 2018a), as described in our previous study (Zhao et al., 2020). Similar to the quality control standards used in our earlier study (Pan et al., 2020), individuals with low genotype rate, ambiguous gender, or cryptic relatedness were excluded from this study.

Our analyses were included in 69 LSD genes, which were reviewed or reported in previous studies (30275469, 29140481, and 32036093) or OMIM database,^3^ and then according to the biochemical types of stored substances of the corresponding genes, they are divided into 11 subtypes, as shown in Supplementary Table 2.

The high-quality variants in those 69 LSD genes were extracted: allele depth (AD) ≥ 5, total depth (DP) ≥ 10, genotype quality (GQ) ≥ 20, and missingness rate < 5% for variants from the WES cohort, whereas AD ≥ 2, DP ≥ 5, GQ ≥ 15 for SNPs, GQ ≥ 30 for indels, and missingness rate < 5% for variants from the WGS cohort. Then, these variants were categorized into missense variants, likely damaging missense variants with ReVe (Li et al., 2018b) >0.7, and loss-of-function variants (LoF, including nonsense, frameshift, or potential splicing variants). Specifically, our previous study suggested that REVEL (Ioannidis et al., 2016) and VEST3 (Carter et al., 2013) have the best overall performance among several prediction software, so that the ReVe (Li et al., 2018b) program was developed to predict destructive missense variants. Moreover, in this study, those likely damaging missense variants and LoF variants were defined as putative damaging variants. Within these categories, variants were filtered based on two minor allele frequency (MAF) thresholds of 0.01 and 0.03. Usually, only MAF < 0.01 was used for the analysis of rare variants. But in this study, we considered including more common LSD variants (MAF < 0.03), which could provide better sensitivity for detecting significant aggregate variant associations (Robak et al., 2017). Of note, patients with pathogenic/likely pathogenic variants of 23 PD-causing genes from the WES cohort, including carriers of *ATP13A2* biallelic variants, were excluded from this study, as described in our previous study (Zhao et al., 2020).

### Statistical Analysis

Gene-based or gene-set-based burden analyses were analyzed using sequence kernel association test-optimal (SKAT-O) tests. The sex, age/AAO, and top five principal components were treated as covariates in the WGS cohort, whereas only the sex and the top five principal components were treated as covariates in the WES cohort (given the controls in the WES cohort were the healthy elderly people, we did not include the age/AAO as the covariates) (Lee et al., 2012; Pan et al., 2020). To adjust for multiple comparisons, we applied the Bonferroni correction to control the family-wise error rate based on a significance level, α of 0.05, and results with a *P*-value of < 0.05, but not surviving the Bonferroni correction, as “suggestive.” To assess the potential oligogenic inheritance of the LSD genes in PD, we explored the proportion of PD cases and controls carrying two or more rare damaging variants of LSD genes.

## Results

For the familial or sporadic early-onset PD cohort, a total of 3,569 unrelated subjects were included, consisting of 1,917 patients with PD and 1,652 controls. To investigate whether there is an aggregate burden of LSD gene variant set on the risk of PD, we first conducted the SKAT-O analysis on the WES cohort. We identified suggestive significant associations among LSD gene different variant sets, especially in the rare or low-frequency putative damaging variant category (*P* [MAF < 1%] = 1.02E-09, *P* [MAF < 3%] = 1.07E-06) (Table 1). To confirm the involvement of other LSD genes other than *GBA*, given the significant effects of *GBA*, a secondary burden analysis was performed after the exclusion of all variants in *GBA*. The results still showed the significant evidence of association between remaining genes and PD in the rare or low-frequency putative damaging variant category (*P* [MAF < 1%] = 0.009, *P* [MAF < 3%] = 0.033) (Table 1). Then, to explore the variant set in which the LSD subgroup gene played an important role in the mechanism of PD, we performed SKAT-O analysis with 11 different subgroups of LSD gene sets on the WES cohort. Specifically, the subgroup of LSDs was classified according to the biochemical types of stored substances of the corresponding genes. We only identified significant association among sphingolipidosis deficiency disorder gene variant sets, especially in the rare or low-frequency putative damaging variant category (*P* [MAF < 1%] = 8.72E-16) (Table 2). Similarly, a secondary burden analysis was conducted after excluding all *GBA* variants, which confirmed the involvement of other sphingolipidosis deficiency disorder genes. It showed suggestive significant evidence between remaining genes and PD only in the rare or low-frequency missense variant category (*P* [MAF < 1%] = 0.025) (Table 2).

**TABLE 1:** Analysis of lysosomal storage disorder (LSD) associated gene rare variant burden in Parkinson’s disease (PD).

	WES cohort				WGS cohort			
	
Variants group	(a) MAF < 1%		(b) MAF < 3%		(c) MAF < 1%		(d) MAF < 3%	
	
	Number^a^	*P*-Value^b^	Number^a^	*P*-Value^b^	Number^a^	*P*-Value^b^	Number^a^	*P*-Value^b^
(1) Missense	1,998(1,942)	1.02E−09(0.009)	2021 (1965)	1.07E−06(0.033)	1596 (1560)	0.010 (0.059)	1,619(1,583)	0.026 (0.142)
(2) Dmis	657 (613)	5.51E−11(0.042)	662 (618)	9.55E−10(0.048)	508 (481)	0.028 (0.206)	513 (486)	0.020 (0.145)
(3) LoF	177 (156)	0.0107 (0.743)	177 (156)	0.0107 (0.743)	53 (53)	0.372 (0.638)	58 (53)	0.372 (0.638)
(4) LoF + Dmis	834 (769)	8.37E−07(0.051)	839 (774)	4.18E−07(0.058)	566 (534)	0.014 (0.148)	571 (539)	0.010 (0.104)

*^a^Number, total number of LSD variant (number of variants excluding GBA). The number of variants excluding those in GBA is shown in parentheses.*

*^b^P-value was calculated by sequence kernel association test-optimal (SKAT-O). Similar with original article, primary analyses consider the variant burden among 69 LSD genes, and secondary analyses were performed excluding all variants in GBA (shown in parentheses).*

*MAF, minor allele frequency; LoF, loss of function; Dmis, damaging missense (ReVe > 0.7). The estimated number of independent tests was 6 in the analysis of LSD gene sets, corresponding to a Bonferroni-corrected significance threshold of P < 0.0083. We considered those results with a P-value < 0.05, but not surviving the Bonferroni correction, as “suggestive”.*

**TABLE 2:** Analysis of LSD subgroups associated gene rare variant burden in PD (MAF < 0.01).

	WES cohort				WGS cohort			
	
Variants group	(1) Missense	(2) Dmis	(3) LoF	(4) LoF + Dmis	(1) Missense	(2) Dmis	(3) LoF	(4) LoF + Dmis
Sphingolipidoses	**339 (1.35E-13)**	**143 (3.50E-13)**	**51 (0.00073)**	**194 (8.72E-16)**	**261 (0.00046)**	**113 (0.058)**	**15 (0.015)**	**128 (0.008)**
Sphingolipidoses (exclude *GBA*)	**283 (0.025)**	99 (0.127)	30 (0.705)	129 (0.116)	225 (0.088)	86 (0.849)	10 (0.068)	96 (0.454)
Mucopolysaccharidoses	295 (0.445)	101 (0.163)	27 (0.777)	128 (0.181)	**213 (0.002)**	78 (0.098)	9 (0.675)	87 (0.086)
Glycogen storage disease	61 (0.270)	28 (0.080)	6 (0.701)	34 (0.084)	146 (0.410)	52 (0.309)	4 (0.138)	56 (0.280)
Glycoproteinoses	179 (0.266)	76 (0.880)	14 (0.220)	90 (0.866)	61 (0.386)	23 (0.102)	3 (0.388)	26 (0.113)
Lipid storage diseases	15 (0.787)	3 (0.755)	–	3 (0.755)	15 (0.399)	2 (0.742)	–	2 (0.742)
Post-translational modification defects	92 (0.170)	42 (0.102)	18 (0.630)	60 (0.103)	**79 (0.031)**	36 (0.604)	6 (0.567)	42 (0.607)
Integral membrane protein disorders	162 (0.149)	52 (0.107)	7 (0.409)	59 (0.084)	121 (0.169)	48 (0.286)	3 (0.446)	51 (0.282)
Neuronal ceroid lipofuscinoses	287 (0.265)	82 (0.595)	19 (0.906)	101 (0.664)	239 (0.600)	62 (0.235)	6 (0.663)	68 (0.219)
Lysosome-related organelles disorders	581 (0.160)	129 (0.229)	33 (0.647)	162 (0.420)	461 (0.253)	86 (0.859)	13 (0.428)	99 (0.840)
Pycnodysostosis	3 (1.000)	1 (0.521)	–	1 (0.521)	6 (0.218)	2 (0.077)	1 (0.297)	3 (0.086)
Sialuria	21 (0.700)	10 (0.681)	2 (0.690)	12 (0.878)	16 (0.651)	10 (0.575)	1 (0.377)	11 (0.440)

*Data were shown as number of variants (SKAT-O P-value).*

*MAF, minor allele frequency; LoF, loss of function; Dmis, damaging missense (ReVe > 0.7). The estimated number of independent tests was 6 in the analysis of LSD gene sets, corresponding to a Bonferroni-corrected significance threshold of P < 0.0083. We considered those results with a P-value < 0.05, but not surviving the Bonferroni correction, as “suggestive”.*

*Bold text indicates a statistically significant assciation with a p-value < 0.05.*

For the sporadic late-onset PD cohort, 1,962 patients with PD and 1,279 controls were included. The suggestive difference was still detected between LSD gene variant sets and PD in the rare putative damaging variant category (*P* [MAF < 1%] = 0.014, *P* [MAF < 3%] = 0.010) (Table 1). Nevertheless, this suggestive difference was not persisted after excluding the *GBA* variants. Then, we also conducted SKAT-O analysis with different subgroups of LSD gene sets on the WGS cohort. We identified a significant association among variant sets in genes of sphingolipidosis deficiency disorders, especially in the rare or low-frequency missense variant category (*P* [MAF < 1%] = 0.00046). Nevertheless, this suggestive difference was not persisted after excluding the *GBA* variants, either. Furthermore, we also detected suggestive significant association among variant sets in genes of mucopolysaccharidosis deficiency disorders (missense variants category, *P* [MAF < 1%] = 0.002) and post-translational modification defect disorders (missense variant category, *P* [MAF < 1%] = 0.031).

For the gene-based analysis, we focused on the rare variants with MAF < 1%. The results showed the most robust association between the LSD gene set and PD. Among the WES cohort with familial PD or sporadic early-onset patients with PD, except for the established *GBA* (*P* = 1.58E-22), the rare putative damaging variant category of additional genes also shown suggestively significant difference between cases and controls, including the potential susceptibility gene *SMPD1* (*P* = 0.015), as well as other four novel genes: *GUSB* (*P* = 0.003), *CLN6* (*P* = 0.015), *PPT1* (*P* = 0.022), and *SCARB2* (*P* = 0.034) (Table 3). With the similar analysis in the WGS cohort (sporadic late-onset PD cases), except for the established *GBA* (*P* = 5.80E-06), the rare putative damaging variant category of additional genes also shown a suggestive significant difference between cases and controls in our cohort, including *GALNS* (*P* = 0.01) and *NAGA* (*P* = 0.05) (Table 3). Specifically, the variants for those 69 genes included in this study are shown in Supplementary Table 3.

**TABLE 3:** Analysis of rare variant burden within each LSD associated gene in PD (MAF < 1%).

Gene	WES cohort				WGS cohort			
	Missense	Dmis	LoF	LoF + Dmis	Missense	Dmis	LoF	LoF + Dmis
**Sphingolipidoses**								
*ARSA*	38 (0.787)	13 (0.183)	3 (0.166)	16 (0.254)	26 (1.000)	13 (0.792)	4 (0.078)	17 (1.000)
*ASAH1*	27 (0.863)	7 (0.637)	3 (0.452)	10 (0.644)	17 (0.264)	5 (0.389)	–	5 (0.389)
*GALC*	40 (0.332)	17 (0.524)	2 (0.154)	19 (0.520)	46 (0.762)	22 (0.857)	0 (1.000)	22 (0.857)
*GBA*	**56 (3.89E-16)**	**44 (1.71E-17)**	**21 (1.14E-05)**	**65 (1.58E-22)**	**36 (1.98E-07)**	**27 (2.93E-05)**	**5 (0.099)**	**32 (5.80E-06)**
*GLA*	3 (0.246)	3 (0.246)	–	3 (0.246)	0 (1.000)	0 (1.000)	0 (1.000)	0 (1.000)
*GM2A*	**14 (0.006)**	2 (0.117)	1 (0.165)	3 (0.203)	12 (0.662)	2 (0.315)	0 (1.000)	2 (0.315)
*HEXA*	22 (0.149)	12 (0.401)	3 (0.116)	15 (0.203)	17 (1.000)	11 (1.000)	1 (0.402)	12 (0.884)
*HEXB*	24 (0.120)	8 (0.251)	3 (1.000)	11 (0.290)	10 (0.427)	2 (0.434)	0 (1.000)	2 (0.434)
*PSAP*	24 (0.636)	8 (0.894)	4 (0.157)	12 (0.405)	23 (0.119)	6 (0.788)	1 (0.439)	7 (0.637)
*SMPD1*	**40 (0.020)**	**18 (0.014)**	4 (0.815)	**22 (0.015)**	39 (0.690)	20 (0.469)	0 (1.000)	20 (0.469)
*ST3GAL5*	14 (0.398)	1 (0.341)	4 (0.626)	5 (0.661)	13 (0.280)	1 (0.487)	1 (0.591)	2 (0.569)
*GLB1*	37 (0.207)	10 (0.598)	3 (0.456)	13 (0.616)	22 (0.078)	4 (0.337)	3 (0.480)	7 (0.581)
**Mucopolysaccharidoses**								
*ARSB*	23 (0.587)	5 (0.581)	2 (0.267)	7 (0.810)	15 (1.000)	5 (0.839)	1 (0.465)	6 (0.836)
*GALNS*	24 (0.073)	16 (0.494)	2 (0.529)	18 (0.487)	25 (0.148)	**17 (0.009)**	–	**17 (0.009)**
*GNS*	22 (0.809)	3 (0.573)	1 (0.154)	4 (0.374)	11 (0.267)	1 (0.393)	–	1 (0.393)
*GUSB*	**21 (0.005)**	**8 (0.003)**	**4 (0.040)**	**12 (0.003)**	21 (0.525)	6 (0.717)	1 (0.449)	7 (0.746)
*HGSNAT*	26 (0.579)	9 (0.160)	2 (0.643)	11 (0.185)	20 (0.207)	6 (0.763)	3 (0.759)	9 (0.858)
*HYAL1*	25 (0.211)	3 (0.468)	2 (0.166)	5 (0.581)	**12 (0.044)**	4 (0.108)	0 (1.000)	4 (0.108)
*IDS*	17 (0.619)	7 (0.617)	–	7 (0.617)	0 (1.000)	0 (1.000)	–	0 (1.000)
*IDUA*	**24 (0.036)**	13 (0.060)	6 (0.731)	19 (0.054)	31 (0.395)	12 (0.465)	1 (0.186)	13 (0.334)
*NAGLU*	29 (0.522)	9 (0.391)	2 (0.472)	11 (0.577)	32 (0.446)	16 (0.087)	–	16 (0.087)
*SGSH*	47 (0.681)	18 (0.608)	3 (0.785)	21 (0.761)	24 (0.238)	7 (0.507)	0 (1.000)	7 (0.507)
*GLB1*	37 (0.207)	10 (0.598)	3 (0.456)	13 (0.616)	22 (0.078)	4 (0.337)	3 (0.480)	7 (0.581)
**Glycogen storage disease**								
*GAA*	61 (0.270)	28 (0.080)	6 (0.701)	34 (0.084)	61 (0.386)	23 (0.102)	3 (0.388)	26 (0.113)
**Glycoproteinoses**								
*AGA*	16 (0.155)	4 (0.506)	2 (0.514)	6 (0.388)	9 (0.457)	2 (0.445)	–	2 (0.445)
*CTSA*	26 (0.198)	8 (0.709)	1 (0.173)	9 (0.555)	26 (0.844)	7 (0.472)	1 (0.149)	8 (0.698)
*FUCA1*	20 (0.526)	10 (0.647)	1 (0.423)	11 (0.677)	16 (0.691)	7 (0.663)	1 (0.094)	8 (0.574)
*MAN2B1*	46 (0.180)	21 (0.205)	3 (0.271)	24 (0.434)	35 (0.746)	8 (0.902)	0 (1.000)	8 (0.902)
*MANBA*	44 (0.340)	18 (0.290)	5 (0.117)	23 (0.303)	36 (0.391)	13 (0.774)	1 (0.247)	14 (0.756)
*NAGA*	27 (0.928)	15 (0.891)	2 (0.774)	17 (0.913)	**24 (0.029)**	**15 (0.047)**	1 (0.456)	16 (0.050)
*NEU1*	–	–	–	–	–	–	–	–
**Lipid storage diseases**								
*LIPA*	15 (0.787)	3 (0.755)	–	3 (0.755)	15 (0.399)	2 (0.742)	–	2 (0.742)
**Post-translational modification defects**								
*GNPTAB*	55 (0.137)	27 (0.100)	10 (0.575)	37 (0.102)	43 (0.537)	23 (0.623)	4 (0.449)	27 (0.618)
*GNPTG*	21 (0.833)	8 (0.763)	8 (0.612)	16 (0.778)	**24 (0.023)**	6 (0.893)	2 (0.392)	8 (0.780)
*SUMF1*	16 (0.236)	7 (0.334)	–	7 (0.334)	12 (0.327)	7 (0.195)	–	7 (0.195)
**Integral membrane protein disorders**								
*CTNS*	32 (0.896)	11 (0.149)	1 (0.332)	12 (0.153)	23 (0.640)	11 (0.341)	2 (0.126)	13 (0.723)
*LAMP2*	9 (0.770)	2 (0.395)	–	2 (0.395)	0 (1.000)	0 (1.000)	–	0 (1.000)
*MCOLN1*	30 (0.723)	7 (0.563)	–	7 (0.563)	21 (0.161)	6 (0.354)	–	6 (0.354)
*NPC1*	53 (0.224)	20 (0.578)	2 (0.566)	22 (0.496)	38 (0.509)	16 (0.164)	0 (1.000)	16 (0.164)
*NPC2*	4 (0.553)	–	–	–	4 (0.880)	1 (0.391)	–	1 (0.391)
*SCARB2*	**20 (0.004)**	7 (0.084)	2 (0.139)	**9 (0.034)**	**21 (0.034)**	7 (0.370)	1 (0.470)	8 (0.381)
*SLC17A5*	14 (0.814)	5 (0.407)	2 (0.212)	7 (0.384)	14 (0.459)	7 (0.415)	–	7 (0.415)
**Neuronal ceroid lipofuscinoses**								
*ATP13A2*	73 (0.171)	14 (0.781)	–	14 (0.781)	58 (0.445)	10 (0.269)	1 (0.459)	11 (0.206)
*CLN3*	19 (0.491)	5 (0.417)	3 (0.801)	8 (0.758)	15 (0.448)	5 (0.683)	0 (1.000)	5 (0.683)
*CLN5*	15 (0.724)	7 (1.000)	–	7 (1.000)	18 (0.574)	6 (0.565)	1 (0.226)	7 (0.619)
*CLN6*	**16 (0.018)**	**12 (0.014)**	1 (0.482)	**13 (0.015)**	14 (0.911)	6 (0.480)	–	6 (0.480)
*CLN8*	17 (0.519)	4 (0.644)	–	4 (0.644)	14 (0.199)	4 (0.418)	1 (0.322)	5 (0.430)
*CTSD*	20 (0.417)	2 (0.676)	–	2 (0.676)	17 (0.567)	1 (0.195)	–	1 (0.195)
*CTSF*	11 (1.000)	2 (0.619)	5 (0.137)	7 (0.289)	11 (0.527)	2 (0.401)	1 (0.233)	3 (0.202)
*DNAJC5*	6 (0.463)	2 (0.620)	–	2 (0.620)	6 (0.625)	3 (0.503)	–	3 (0.503)
*GRN*	37 (0.527)	6 (0.264)	4 (0.743)	10 (0.463)	34 (0.643)	10 (0.468)	1 (0.457)	11 (0.376)
*KCTD7*	14 (0.880)	5 (0.870)	2 (0.101)	7 (0.611)	4 (0.776)	1 (0.848)	–	1 (0.848)
*MFSD8*	24 (0.236)	9 (0.186)	1 (0.373)	10 (0.200)	24 (0.340)	7 (0.383)	1 (0.447)	8 (0.294)
*PPT1*	10 (0.729)	**5 (0.022)**	–	**5 (0.022)**	12 (0.477)	3 (0.705)	–	3 (0.705)
*TPP1*	25 (0.194)	9 (0.647)	3 (0.444)	12 (0.406)	12 (0.161)	4 (0.616)	–	4 (0.616)
**LRO disorders**								
*HPS1*	52 (0.235)	6 (0.790)	5 (0.759)	11 (0.837)	45 (1.000)	4 (0.156)	1 (0.398)	5 (0.129)
*AP3B1*	**38 (0.010)**	10 (0.643)	–	10 (0.643)	20 (0.857)	4 (0.820)	–	4 (0.820)
*HPS3*	51 (0.670)	21 (0.626)	5 (0.459)	26 (0.685)	37 (0.832)	13 (0.695)	2 (0.210)	15 (0.781)
*HPS4*	47 (1.000)	5 (0.423)	3 (0.308)	8 (0.805)	41 (0.145)	6 (0.626)	–	6 (0.626)
*HPS5*	41 (0.392)	11 (0.750)	3 (0.595)	14 (0.605)	40 (0.092)	9 (0.080)	1 (0.274)	10 (0.080)
*HPS6*	36 (0.747)	10 (0.189)	6 (0.052)	16 (0.145)	26 (0.147)	5 (0.796)	2 (0.185)	7 (0.606)
*DTNBP1*	21 (0.267)	3 (0.075)	3 (0.171)	6 (0.326)	20 (0.414)	2 (0.756)	2 (0.423)	4 (0.763)
*BLOC1S3*	14 (0.139)	1 (0.411)	1 (0.505)	2 (0.685)	13 (0.064)	–	1 (0.373)	1 (0.373)
*BLOC1S6*	8 (0.783)	2 (0.452)	1 (0.414)	3 (0.544)	8 (0.232)	3 (0.772)	–	3 (0.772)
*AP3D1*	64 (0.652)	5 (0.650)	2 (0.256)	7 (0.434)	41 (0.512)	3 (0.859)	–	3 (0.859)
*LYST*	**146 (0.016)**	24 (0.249)	1 (0.633)	25 (0.254)	116 (0.736)	17 (0.264)	1 (0.335)	18 (0.266)
*MYO5A*	50 (0.369)	25 (0.231)	2 (0.594)	27 (0.236)	43 (0.210)	16 (0.740)	2 (0.682)	18 (0.750)
*RAB27A*	13 (0.350)	6 (0.450)	1 (0.369)	7 (0.475)	11 (0.544)	4 (0.287)	1 (0.478)	5 (0.524)
**Enzyme cathepsin K**								
*CTSK*	3 (1.000)	1 (0.521)	–	1 (0.521)	6 (0.218)	2 (0.077)	1 (0.297)	3 (0.086)
**N-acetylneuraminic acid synthase**								
*GNE*	21 (0.700)	10 (0.681)	2 (0.690)	12 (0.878)	16 (0.651)	10 (0.575)	1 (0.377)	11 (0.440)

*Data were shown as number of variants (SKAT-O P-value). MAF, minor allele frequency; Dmis, damaging missense variant; LoF, loss of function.*

*Bold text indicates a statistically significant assciation with a p-value less than 0.05.*

**FIGURE 1:**
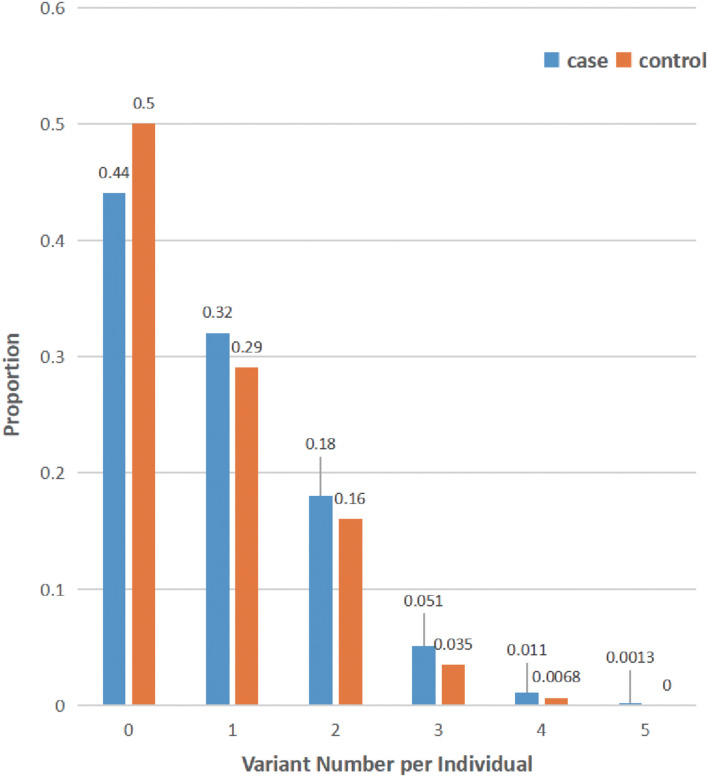
Distribution of lysosomal storage disorder (LSD) variants in all subjects. The number of rare damaging LSD variants (MAF < 1%, ReVe score > 0.7) in each individual is shown as the proportion of cases (blue) or controls (orange). Many subjects harbor multiple LSD variants, and all variants of 69 LSD genes were considered.

Finally, the distribution of rare damaging variants of the LSD gene in our cohort was also analyzed (MAF < 1%): the average variant burden among cases (0.879 alleles per individual) was slightly higher than that seen in controls (0.751 alleles per individual) within our cohort (Figure 1). Of note, only 13 of 1,962 patients with PD carried homozygous putative damaging variants of LSD genes (Supplementary Table 4). Additionally, 56.3% of cases have at least rare likely damaging variants in one LSD gene, whereas 24.0% carry multiple alleles.

## Discussion

In this study, our results strengthen the vital connection between the LSD genes and PD in the Chinese mainland population, which was supported by the burden of non-synonymous alleles of 69 LSDs causing genes, especially putative damaging variants, in the association with PD. Specifically, the associations were statistically significant in the early-onset cohort of patients with PD, even excluding individuals carrying *GBA* variants. Then, the new potential specific subgroups of LSD and novel potential susceptibility genes identified in this study further implicate the importance of lysosomal mechanisms in PD pathogenesis. Moreover, the results also indicated certain genetic differences between early-onset PD and late-onset PD.

As described above, we identified suggestive associations between sphingolipidosis storage disease genes and PD in both cohorts. Specifically, among the sporadic late-onset PD cohort, we also identified the potential connection between post-translational modification defects, mucopolysaccharidosis storage disease genes, and PD. Considering the combined consideration of LSDs or their subgroup genes may improve statistical power over a single gene (Zuk et al., 2014), we should pay more attention to the relationship between the mechanisms of post-translational modification defects, mucopolysaccharidosis storage disease, and PD, especially in the lysosomal mechanism of late-onset patients with PD. Neurodegeneration is a remarkable phenotype of almost all LSDs (Platt et al., 2018), indicating the correlation between lysosomal degradation and the maintenance of neuronal health. Several studies have proved that activities of GBA and several other sphingolipid hydrolase enzymes are reduced in the brain or other body fluid of PD (Balducci et al., 2007; Alcalay et al., 2015; Chiasserini et al., 2015; Huebecker et al., 2019). Interestingly, it has been shown that phosphorylation, SUMOylation, and ubiquitination play a role in protein aggregation, exocytosis, and degradation (Junqueira et al., 2019). Specifically, for several mucopolysaccharidosis storage diseases, enzyme replacement therapy or hematopoietic stem cell therapy has been available (Platt et al., 2018), which may have reference value for the treatment of late-onset patients with PD.

Lysosomal storage disorders were mainly childhood-onset Mendelian disorders, but PD was a neurodegenerative disease significantly associated with aging (Ysselstein et al., 2019). As an essential pathway for α-synuclein degradation, lysosomal biology plays an important role in PD (Moors et al., 2016). This study supported shared genetic factors between the LSDs and PD in the Chinese mainland population, as the similar results from previous studies (Robak et al., 2017; Hopfner et al., 2020), which may provide design ideas for future experimental research on the pathogenesis of idiopathic PD. Understanding the pathophysiology of the endosome-lysosome-autophagy system is of great significance for the development of new therapeutic strategies not only for LSDs but also for PD (Paciotti et al., 2020).

Then, we verified the robust association of *GBA* and PD, as well as the suggestive link between different additional susceptible genes and PD in the familial or sporadic early-onset PD cohort (*SMPD1, GUSB, CLN6, PPT1*, and *SCARB2*) and sporadic late-onset PD cohort (*GALNS* and *NAGA*). As we could see, *GBA* and *SMPD1* were identified as the susceptibility genes in both European-ancestry populations and our population, which suggested that they might play a robust role in the diverse populations. Specifically, *SMPD1* encodes for acid sphingomyelinase (ASM), and its deficiency may affect the hydrolysis of sphingomyelin to phosphocholine and ceramide in late endosomes and lysosomes (Ysselstein et al., 2019). The evidence in our study and previous studies supported that *SMPD1* variants increase the risk for PD, and then it may also suggest the potential role of the reduced ASMase activity in α-synuclein accumulation (Alcalay et al., 2019). However, there are still very few studies about it. Therefore, it needs more attention in the follow-up experimental functional studies in PD. Specifically, the association of variants of the *SCARB2* gene and PD was previously reported (Nalls et al., 2014; Alcalay et al., 2016; Rudakou et al., 2021) in European cohorts. *SCARB2* encodes the membrane protein required for correct targeting of glucocerebrosidase (GCase) to the lysosome (Zeigler et al., 2014). The association between the *SCARB2* gene and PD was hypothesized to be due to differential trafficking of GCase to the lysosome (Dardis et al., 2009). Furthermore, the differences in the additional susceptible genes identified in our cohort and European-ancestry populations might implicate the genetic heterogeneity of the different populations and different AAO ranges (Pan et al., 2020). The genetic heterogeneity of different subtypes of PD may also indicate that their underlying mechanisms could be different, and future genetic research may also need further analysis for different subtypes of PD.

Interestingly, it suggestively supported that six genes were associated with the familial or sporadic early-onset cases, whereas only three were linked to the sporadic late-onset cases. We may speculate the possibility that familial or early-onset cases were more likely to be caused by putative damaging variants with statistically significant amounts in certain susceptibility genes. Nevertheless, only associated genes of one LSD subgroup were suggestively associated with the familial or sporadic early-onset cases, whereas associated genes of three LSD subgroups were linked to the sporadic late-onset cases. We may infer that sporadic late-onset cases may be prone to be caused by the accumulation of a small amount of putative damaging variants in plenty of LSD genes. However, this idea still needs further replication in a larger multiethnic population. In addition, considering the cumulative effect of putative damaging variants of the LSD genes in PD, the role of oligogenic variants for missing heritability in PD (Pihlstrom and Toft, 2011) may need more attention.

Of note, for several potential novel PD risk genes identified in our study and specific genes implicated from other studies, it may also help provide valuable suggestions for the treatment of PD based on the novel treatment of LSDs. Mutations in *GUSB, CLN6*, and *PPT1* cause Sly disease, neuronal ceroid lipofuscinosis (CLN6), and neuronal ceroid lipofuscinosis (CLN1), respectively, whereas mutations in *GALNS* and *NAGA* cause Morquio A disease and Schindler disease/Kanzaki disease, respectively. Among them, the proportion of neuronal ceroid lipofuscinosis (NCL) is relatively large. Moreover, previous studies have also implicated the association between NCL and PD, including the evidence of the analysis of the brain tissues of Atp13a2 conditional-knockout mice (Sato et al., 2016) and the identification of the reduced dopamine transporter density in the striatum in juvenile NCL patients with parkinsonism (Aberg et al., 2000). Overall, the potential mechanism between these LSD genes and PD needs further experimental verification. Furthermore, there was plenty of evidence supporting the relationship between autophagy-lysosomal pathway damage and misfolded α-synuclein aggregation and propagation, so compounds targeting autophagic-lysosomal pathway restoration may serve as a promising disease-modifying strategy for PD treatment (Bellomo et al., 2020).

However, there are still several limitations in this study. First, two different methods of sequencing (e.g., WES vs. WGS) were used for the studied cohorts, and the WES variants or the WGS variants were not validated by a second independent sequencing technology and may include certain false positives. Second, no further verification of the novel susceptibility genes discovered in another larger cohort and a larger PD sequencing data set with higher statistical power are needed to resolve specific LSD genes that cause PD risk. Third, there was no in-depth exploration of the new and known susceptibility genes in terms of functional mechanisms.

## Conclusion

Our results supported the association between LSD genes and PD, which may help design experimental studies to elucidate the lysosomal mechanisms in the pathogenesis of PD. However, a larger sample size is needed to confirm these associations in multiethnic populations, and the risk of a single LSD gene should be cautiously interpreted, especially in different ethnicities. Moreover, we revealed several novel risk genes in patients with PD from mainland China. We identified the genetic heterogeneity in early-onset and late-onset patients with PD, which may provide valuable suggestions for treating different subtypes of PD.

## Data Availability Statement

According to national legislation/guidelines, specifically theAdministrative Regulations of the People’s Republic of China on HumanGenetic Resources (http://www.gov.cn/zhengce/content/2019-06/10/content_5398829.htm, http://english.www.gov.cn/policies/latest_releases/2019/06/10/content_281476708945462.htm), no additional raw data is available at this time. Data of this project can be accessed after an approval application to the China National Genebank(CNGB, https://db.cngb.org/cnsa/). Please refer to https://db.cngb.org/, oremail: CNGBdb@cngb.org for detailed application guidance. The accession code CNP0002235 should be included in the application.

## Ethics Statement

The studies involving human participants were reviewed and approved by the Xiangya Hospital, Central South University. The patients/participants provided their written informed consent to participate in this study.

## Author Contributions

B-ST: obtained funding, study supervision, had full access to all the data in this study, and takes responsibility for the integrity of the data and the accuracy of the data analysis. Y-WZ and B-ST: study concept and design and drafting of the manuscript. Y-WZ, H-XP, ZL, YW, QZ, Z-HF, KX, ZW, RH, BL, GZ, QX, Q-YS, X-XY, and J-FG: acquisition, analysis, or interpretation of data. J-CL, J-FG, and B-ST: administrative, technical, or material support. All authors: critical revision of the manuscript for important intellectual content.

## Conflict of Interest

The authors declare that the research was conducted in the absence of any commercial or financial relationships that could be construed as a potential conflict of interest.

## Publisher’s Note

All claims expressed in this article are solely those of the authors and do not necessarily represent those of their affiliated organizations, or those of the publisher, the editors and the reviewers. Any product that may be evaluated in this article, or claim that may be made by its manufacturer, is not guaranteed or endorsed by the publisher.

## Footnotes

1
http://pd-mdcnc.com/

2
http://varcards.biols.ac.cn/

3
https://www.omim.org/
