# Supplementary material for: The Association Between Lysosomal Storage Disorder Genes and Parkinson’s Disease: A Large Cohort Study in Chinese Mainland Population
Source: Front Aging Neurosci. 2021 Nov 15;13:749109. doi: 10.3389/fnagi.2021.749109 (PMC8634711; doi:10.3389/fnagi.2021.749109)
Supplement: Supplementary file 1 [file Data_Sheet_1.PDF]

Supplementary Table 1. Basic demographic characteristics of included subjects.

Cohorts	WES cohort				WGS cohort	
	AD probands (n = 327)	AR probands (n = 150)	EOPD (n = 1,440)	Control group 1 (n = 1,652)	LOPD (n = 1,962)	Control group 2 (n = 1,279)
Age	56.74 ± 11.66	60.7 ± 11.3	50.25 ± 7.136	62.03 ± 12.59	66.76 ± 7.078	62.32 ± 7.109
Age at onset	51.78 ± 10.8	54.98 ± 11.98	44.17 ± 5.786	-	61.88 ± 6.927	-
Sex (male/female)	180/147	82/68	786/654	795/857	984/978	613/666
Genotyping method	WES	WES	WES	WES	WGS	WGS

* WES = whole-exome sequencing; WGS = whole-genome sequencing.

Supplementary Table 2. Summary of targeted genes in this study

Gene	Disease name	Gene full name	OMIM	Location	Ref
**Sphingolipidoses** *					
ARSA	Metachromatic leukodystrophy	Arylsulfatase A	607574	22q13.33	[1]
ASAH1	Farber Lipogranulomatosis	N-acylsphingosine amidohydrolase 1	613468	8p22	[1]
GALC	Krabbe disease	Galactosylceramidase	606890	14q31.3	[1]
GBA	Gaucher disease	Glucosylceramidase beta	606463	1q22	[1]
GLA	Fabry disease	Galactosidase alpha	300644	Xq22.1	[1]
GM2A	GM2-gangliosidosis	GM2 ganglioside activator	613109	5q33.1	[1]
HEXA	Tay-Sachs disease	Hexosaminidase subunit alpha	606869	15q23	[1]
HEXB	Sandhoff disease	Hexosaminidase subunit beta	606873	5q13.3	[1]
PSAP	Sphingolipid-activator deficiency	Prosaposin	176801	10q22.1	[1]
SMPD1	Niemann-Pick disease type A/B	Sphingomyelin phosphodiesterase 1	607608	11p15.4	[1]
ST3GAL5	GM3-gangliosidosis	ST3 beta-galactoside alpha-2,3-sialyltransferase 5	604402	2p11.2	[2]
GLB1	GM1-gangliosidosis/Morquio B	Galactosidase beta 1	611458	3p22.3	[1]
**Mucopolysaccharidoses** *					
ARSB	Maroteaux-Lamy disease	Arylsulfatase B	611542	5q14.1	[1]
GALNS	Morquio A disease	Galactosamine (N-acetyl)-6-sulfatase	612222	16q24.3	[1]
GNS	Sanfilippo D syndrome	Glucosamine (N-acetyl)-6-sulfatase	607664	12q14.3	[1]
GUSB	Sly disease	Glucuronidase beta	611499	7q11.21	[1]
HGSNAT	Sanfilippo C syndrome	Heparan-alpha-glucosaminide N-acetyltransferase	610453	8p11.21-p11.1	[1]
HYAL1	Mucopolysaccharidosis type IX	Hyaluronoglucosaminidase 1	607071	3p21.31	[1]
IDS	Hunter syndrome	Iduronate 2-sulfatase	300823	Xq28	[1]
IDUA	Hurler syndrome	Alpha-L-Iduronidase	252800	4p16.3	[1]
NAGLU	Sanfilippo B syndrome	N-acetyl-alpha-glucosaminidase	609701	17q21.2	[1]
SGSH	Sanfilippo A syndrome	N-sulfoglucosamine sulfohydrolase	605270	17q25.3	[1]
GLB1	GM1-gangliosidosis/Morquio B	Galactosidase beta 1	611458	3p22.3	[1]
**Glycogen storage disease**					
GAA	Pompe disease	Glucosidase alpha, acid	606800	17q25.3	[1]
**Glycoproteinoses**					
AGA	Aspartylglucosaminuria	Aspartylglucosaminidase	613228	4q34.3	[1]
CTSA	Galactosialidosis	Cathepsin A	613111	20q13.12	[1]
FUCA1	Fucosidosis	alpha-L-fucosidase 1	612280	1p36.11	[1]
MAN2B1	Alpha-mannosidosis	Mannosidase alpha class 2B member 1	609458	19p13.13	[1]
MANBA	Beta-mannosidosis	Mannosidase beta	609489	4q24	[1]
NAGA	Schindler disease/Kanzaki disease	alpha-N-acetylgalactosaminidase	104170	22q13.2	[1]
NEU1	Sialidosis	Neuraminidase 1	608272	6p21.33	[1]
**Lipid storage diseases**					
LIPA	Wolman disease	Lipase A, Lysosomal Acid Type	613497	10q23.31	[1]
**Post-translational modification defects***					
GNPTAB	I-Cell disease	N-acetylglucosamine-1-phosphate transferase alpha and beta subunits	607840	12q23.2	[1]
GNPTG	Mucolipidosis III gamma	N-acetylglucosamine-1-phosphate transferase gamma subunit	607838	16p13.3	[1]

SUMF1	Multiple sulfatase deficiency	Sulfatase modifying factor 1	607939	3p26.1	[1]
**Integral membrane protein disorders**					
CTNS	Cystinosis	Cystinosin, lysosomal cystine transporter	606272	17p13.2	[1]
LAMP2	Danon disease	Lysosomal associated membrane protein 2	309060	Xq24	[1]
MCOLN1	Mucolipidosis type IV	Mucolipin 1	605248	19p13.2	[1]
NPC1	Niemann-Pick disease type C1	NPC intracellular cholesterol transporter 1	607623	18q11.2	[1]
NPC2	Niemann-Pick disease type C2	NPC intracellular cholesterol transporter 2	601015	14q24.3	[1]
SCARB2	Action mycolonus-renal failure syndrome	Scavenger receptor class B member 2	602257	4q21.1	[1]
SLC17A5	Salla disease	Solute carrier family 17 member 5	604322	6q13	[1]
**Neuronal ceroid lipofuscinoses** *					
ATP13A2	Kufor-Rakeb syndrome	ATPase 13A2	610513	1p36.13	[1]
CLN3	Neuronal ceroid lipofuscinosis	CLN3, battenin	607042	16p12.1	[1]
CLN5	Neuronal ceroid lipofuscinosis	CLN5, intracellular trafficking protein	608102	13q22.3	[1]
CLN6	Neuronal ceroid lipofuscinosis	CLN6, transmembrane ER protein	606725	15q23	[1]
CLN8	Neuronal ceroid lipofuscinosis	CLN8, transmembrane ER and ERGIC protein	607837	8p23.3	[1]
CTSD	Neuronal ceroid lipofuscinosis	Cathepsin D	116840	11p15.5	[1]
CTSF	Neuronal ceroid lipofuscinosis	Cathepsin F	603539	11q13.2	[1]
DNAJC5	Neuronal ceroid lipofuscinosis	DnaJ heat shock protein family (Hsp40) member C5	611203	20q13.33	[1]
GRN	Neuronal ceroid lipofuscinosis	Granulin precursor	138945	17q21.31	[1]
KCTD7	Neuronal ceroid lipofuscinosis	Potassium channel tetramerization domain containing 7	611725	7q11.21	[1]
MFSD8	Neuronal ceroid lipofuscinosis	Major facilitator superfamily domain containing 8	611124	4q28.2	[1]
PPT1	Neuronal ceroid lipofuscinosis	Palmitoyl-protein thioesterase 1	600722	1p34.2	[1]
TPP1	Neuronal ceroid lipofuscinosis	Tripeptidyl peptidase 1	607998	11p15.4	[1]
**Lysosome-related organelles disorders**					
HPS1	Hermansky-Pudlak Syndrome Type 1	HPS1, biogenesis of lysosomal organelles complex 3 subunit 1	604982	10q24.2	[1]
AP3B1	Hermansky-Pudlak Syndrome Type 2	Adaptor related protein complex 3 beta 1 subunit	603401	5q14.1	[1]
HPS3	Hermansky-Pudlak Syndrome Type 3	HPS3, biogenesis of lysosomal organelles complex 2 subunit 1	606118	3q24	[1]
HPS4	Hermansky-Pudlak Syndrome Type 4	HPS4, biogenesis of lysosomal organelles complex 3 subunit 2	606682	22q12.1	[1]
HPS5	Hermansky-Pudlak Syndrome Type 5	HPS5, biogenesis of lysosomal organelles complex 2 subunit 2	607521	11p15.1	[1]
HPS6	Hermansky-Pudlak Syndrome Type 6	HPS6, biogenesis of lysosomal organelles complex 2 subunit 3	607522	10q24.32	[1]
DTNBP1	Hermansky-Pudlak Syndrome Type 7	Dystrobrevin binding protein 1	607145	6p22.3	[1]
BLOC1S3	Hermansky-Pudlak Syndrome Type 8	Biogenesis of lysosomal organelles complex 1 subunit 3	609762	19q13.32	[1]

BLOC1S6	Hermansky-Pudlak Syndrome Type 9	Biogenesis of lysosomal organelles complex 1 subunit 6	604310	15q21.1	[1]
AP3D1	Hermansky-Pudlak syndrome Type 10	Adaptor related protein complex 3 delta 1 subunit	607246	19p13.3	[3]
LYST	Chédiak–Higashi disease	lysosomal trafficking regulator	606897	1q42.3	[1]
MYO5A	Griscelli syndrome 1, also known as Elejalde syndrome	Myosin VA	160777	15q21.2	[1]
RAB27A	Griscelli syndrome 2	RAB27A, member RAS oncogene family	603868	15q21.3	[1]
**Enzyme cathepsin K** *					
CTSK	Pycnodysostosis	Cathepsin K	601105	1q21.3	[2]
**N-acetylneuraminic acid synthase** *					
GNE	Sialuria	glucosamine (UDP-N-acetyl)-2-epimerase/N-acetyl mannosamine kinase	603824	9p13.3	[4]

*Denotes enzyme deficiency disorders.

[1] Platt FM, d'Azzo A, Davidson BL, Neufeld EF, Tifft CJ. Lysosomal storage diseases. Nat Rev Dis Primers 2018;4(1):27.

[2] Robak LA, Jansen IE, van Rooij J, et al. Excessive burden of lysosomal storage disorder gene variants in Parkinson's disease. Brain 2017;140(12):3191-3203.

[3] https://www.omim.org/entry/607246?search=AP3D1&highlight=ap3d1

[4] Zanetti A, D'Avanzo F, Bertoldi L, et al. Setup and Validation of a Targeted Next-Generation Sequencing Approach for the Diagnosis of Lysosomal Storage Disorders. J Mol Diagn. 2020;22(4):488-502.

Supplementary Table 3. Rare nonsynonymous variants of LSDs genes identified in our cohort

Gene	Position (hg19)	Ref	Alt	NM number	AAChange	Consequence	gnomAD_exome_EAS [a]	gnomAD_genome_EAS [a]	ExAC_EAS [a]	ReVe [b]	WES cohort		WGS cohort		Pathogenic in Clinvar [c]
											Case (n=1917)	Control (n=1652)	Case (n=1962)	Control (n=1279)	
ATP13A2	1:17312526	C	T	NM_001141974	c.3431G>A:p.R1144H	missense	0	0	0	0.272:T	0	0	1	0	-
ATP13A2	1:17312565	C	T	NM_001141974	c.3392G>A:p.G1131E	missense	0.0001	-	-	0.299:T	0	1	0	1	-
ATP13A2	1:17312592	G	A	NM_001141974	c.3365C>T:p.P1122L	missense	0	0	0	0.181:T	0	1	1	0	B
ATP13A2	1:17312643	G	T	NM_001141974	c.3314C>A:p.T1105K	missense	-	-	-	0.258:T	0	1	2	1	-
ATP13A2	1:17312667	T	C	NM_001141974	c.3290A>G:p.H1097R	missense	5.91E-05	-	-	0.227:T	0	1	0	1	-
ATP13A2	1:17312707	G	A	NM_001141974	c.3250C>T:p.H1084Y	missense	-	-	-	0.246:T	0	0	1	0	-
ATP13A2	1:17312725	G	T	NM_001141974	c.3232C>A:p.P1078T	missense	-	-	-	0.486:T	0	1	0	0	-
ATP13A2	1:17312733	C	T	NM_001141974	c.3224G>A:p.R1075H	missense	0.0001	-	-	0.223:T	1	0	0	0	-
ATP13A2	1:17312749	G	T	NM_001141974	c.3208C>A:p.L1070M	missense	-	-	-	0.542:T	0	0	1	0	-
ATP13A2	1:17312758	G	A	NM_001141974	c.3199C>T:p.R1067X	stopgain	6.2E-05	0	0.0002	0.162:T	0	0	1	0	-
ATP13A2	1:17312804	T	C	NM_022089	c.3455A>G:p.K1152R	missense	-	-	-	0.117:T	0	1	0	1	-
ATP13A2	1:17312815	G	A	NM_001141974	c.3142C>T:p.P1048S	missense	-	-	-	0.488:T	0	1	0	0	-
ATP13A2	1:17312817	G	A	NM_001141974	c.3140C>T:p.P1047L	missense	0	-	0	0.102:T	1	1	0	0	-
ATP13A2	1:17312851	G	A	NM_001141974	c.3106C>T:p.R1036C	missense	0	-	0	0.634:T	0	0	0	1	-
ATP13A2	1:17312978	C	T	NM_022089	c.3385G>A:p.V1129M	missense	0.0002	-	0.0004	0.303:T	3	2	3	2	-
ATP13A2	1:17312992	A	T	NM_022089	c.3371T>A:p.V1124D	missense	-	-	-	0.629:T	0	0	0	1	-
ATP13A2	1:17313092	C	T	NM_022089	c.3271G>A:p.V1091I	missense	5.82E-05	-	0	0.073:T	0	2	1	0	-
ATP13A2	1:17313110	G	C	NM_022089	c.3253C>G:p.L1085V	missense	-	-	-	0.377:T	1	0	0	0	-
ATP13A2	1:17313342	C	T	NM_001141974	c.3061G>A:p.V1021M	missense	0	0	0	0.040:T	0	1	0	0	US
ATP13A2	1:17313584	C	T	NM_001141974	c.2908G>A:p.G970S	missense	0.0004	0	0.0011	0.234:T	1	2	0	4	US
ATP13A2	1:17313643	C	T	NM_001141974	c.2849G>A:p.G950E	missense	5.8E-05	-	0.0001	0.589:T	0	0	0	1	-
ATP13A2	1:17313643	C	A	NM_001141974	c.2849G>T:p.G950V	missense	-	-	-	0.626:T	0	0	1	0	-
ATP13A2	1:17313656	C	T	NM_001141974	c.2836G>A:p.V946M	missense	5.8E-05	-	-	0.415:T	0	0	1	0	-
ATP13A2	1:17313682	G	A	NM_001141974	c.2810C>T:p.T937M	missense	0	0	0	0.630:T	1	0	0	0	CIP
ATP13A2	1:17313686	G	A	NM_001141974	c.2806C>T:p.R936C	missense	0	-	-	0.831:D	0	1	0	0	-
ATP13A2	1:17313716	T	A	NM_001141974	c.2776A>T:p.I926F	missense	-	-	-	0.616:T	0	0	1	0	-
ATP13A2	1:17314634	G	A	NM_001141974	c.2726C>T:p.T909M	missense	0	0.0006	-	0.799:D	0	0	1	0	-
ATP13A2	1:17314827	T	C	NM_001141974	c.2620A>G:p.M874V	missense	-	0	-	0.218:T	0	0	1	0	-

ATP13A2	1:17314833	C	T	NM_001141974	c.2614G>A:p.V872M	missense	0	-	0	0.810:D	1	0	0	0	US
ATP13A2	1:17314837	C	A	NM_001141974	c.2610G>T:p.E870D	missense	5.80E-05	-	-	0.296:T	1	0	0	0	-
ATP13A2	1:17314937	T	G	NM_001141974	c.2510A>C:p.N837T	missense	-	-	-	0.892:D	1	0	0	0	-
ATP13A2	1:17314941	C	T	NM_001141974	c.2506G>A:p.A836T	missense	-	-	-	0.805:D	0	1	0	0	-
ATP13A2	1:17314956	T	A	NM_001141974	c.2491A>T:p.M831L	missense	0.0001	-	0.0001	0.866:D	1	0	0	0	-
ATP13A2	1:17314965	A	T	NM_001141974	c.2482T>A:p.C828S	missense	-	-	-	0.532:T	0	1	0	0	-
ATP13A2	1:17316252	C	A	NM_001141974	c.2411G>T:p.G804V	missense	-	-	-	0.495:T	0	0	0	1	-
ATP13A2	1:17316455	C	T	NM_022089	c.2456G>A:p.R819Q	missense	0	0	0	0.289:T	0	0	1	0	p.R819X
ATP13A2	1:17316459	G	A	NM_022089	c.2452C>T:p.P818S	missense	0	-	-	0.205:T	1	0	1	0	-
ATP13A2	1:17316482	G	A	NM_022089	c.2429C>T:p.A810V	missense	0.0002	-	0.0005	0.229:T	1	0	0	0	-
ATP13A2	1:17316489	G	C	NM_022089	c.2422C>G:p.Q808E	missense	0	-	0	0.490:T	1	0	0	0	US
ATP13A2	1:17316627	C	T	NM_001141974	c.2392G>A:p.V798I	missense	5.80E-05	-	0	0.259:T	1	0	0	0	US
ATP13A2	1:17316674	G	A	NM_001141974	c.2345C>T:p.A782V	missense	-	-	-	0.540:T	0	0	0	1	-
ATP13A2	1:17316735	C	T	NM_001141974	c.2284G>A:p.V762M	missense	-	-	-	0.622:T	0	0	1	0	-
ATP13A2	1:17316756	C	T	NM_001141974	c.2263G>A:p.V755M	missense	0.0004	0.0006	0.0005	0.907:D	2	0	1	0	-
ATP13A2	1:17318244	C	T	NM_001141974	c.2221G>A:p.A741T	missense	0.0015	0.0012	0.0017	0.178:T	17	5	11	7	US
ATP13A2	1:17318261	C	T	NM_001141974	c.2204G>A:p.R735Q	missense	0	-	-	0.480:T	1	0	0	0	-
ATP13A2	1:17318267	G	C	NM_001141974	c.2198C>G:p.A733G	missense	0.0001	0.0006	0.0001	0.209:T	3	3	5	0	-
ATP13A2	1:17318282	G	A	NM_001141974	c.2183C>T:p.T728M	missense	0.0038	0.0025	0.0042	0.276:T	5	5	11	7	CIP
ATP13A2	1:17318308	C	T	NM_001141974	c.2157G>A:p.M719I	missense	-	-	-	0.443:T	0	1	0	0	-
ATP13A2	1:17318526	C	G	NM_001141974	c.2089G>C:p.E697Q	missense	-	-	-	0.097:T	0	0	1	0	-
ATP13A2	1:17318765	C	T	NM_001141974	c.1963G>A:p.V655M	missense	-	-	-	0.809:D	1	0	0	0	-
ATP13A2	1:17318782	T	C	NM_001141974	c.1946A>G:p.K649R	missense	-	-	-	0.921:D	1	0	0	0	-
ATP13A2	1:17318821	A	G	NM_001141974	c.1907T>C:p.V636A	missense	0.0004	-	0.0005	0.252:T	4	1	2	1	-
ATP13A2	1:17318864	G	A	NM_001141974	c.1864C>T:p.R622C	missense	5.82E-05	-	-	0.774:D	0	2	0	0	-
ATP13A2	1:17318983	T	C	NM_001141974	c.1828A>G:p.M610V	missense	0.0003	0.0006	0.0003	0.211:T	2	4	3	1	US
ATP13A2	1:17318997	G	C	NM_001141974	c.1814C>G:p.P605R	missense	-	-	0.0001	0.277:T	0	1	1	0	-
ATP13A2	1:17319004	A	C	NM_001141974	c.1807T>G:p.W603G	missense	-	-	-	0.201:T	0	5	1	1	-
ATP13A2	1:17319067	C	T	NM_001141974	c.1744G>A:p.E582K	missense	-	-	-	0.661:T	0	1	0	0	-
ATP13A2	1:17320141	C	G	NM_001141974	c.1717G>C:p.V573L	missense	-	-	-	0.356:T	0	0	1	0	-
ATP13A2	1:17320162	C	T	NM_001141974	c.1696G>A:p.D566N	missense	6.53E-05	0	0	0.577:T	1	0	0	0	CIP
ATP13A2	1:17320185	C	T	NM_001141974	c.1673G>A:p.R558Q	missense	6.50E-05	0	0.0003	0.055:T	0	1	0	0	LB

ATP13A2	1:17320186	G	A	NM_001141974	c.1672C>T:p.R558W	missense	0.0001	0	0.0003	0.205:T	2	0	1	1	-
ATP13A2	1:17320300	C	T	NM_001141974	c.1558G>A:p.V520M	missense	0	-	-	0.813:D	0	0	0	1	-
ATP13A2	1:17322476	C	T	NM_001141974	c.1522G>A:p.D508N	missense	0	-	0	0.769:D	0	0	1	0	-
ATP13A2	1:17322521	G	T	NM_001141974	c.1477C>A:p.P493T	missense	0	-	-	0.888:D	0	0	1	0	-
ATP13A2	1:17322593	G	C	NM_001141974	c.1405C>G:p.P469A	missense	0.0003	-	0.0007	0.815:D	0	1	0	0	US
ATP13A2	1:17322626	C	T	NM_001141974	c.1372G>A:p.D458N	missense	5.8E-05	-	0	0.635:T	0	0	0	2	-
ATP13A2	1:17322635	G	A	NM_001141974	c.1363C>T:p.R455W	missense	0.0004	0.0006	0.0005	0.907:D	0	1	1	0	-
ATP13A2	1:17322750	C	T	NM_001141974	c.1337G>A:p.R446Q	missense	0	0	0	0.302:T	0	1	0	0	CIP
ATP13A2	1:17322756	C	T	NM_001141974	c.1331G>A:p.R444Q	missense	5.8E-05	-	0.0001	0.824:D	0	0	1	0	p.R444X
ATP13A2	1:17322790	G	A	NM_001141974	c.1297C>T:p.L433F	missense	-	-	-	0.283:T	0	1	0	0	-
ATP13A2	1:17322944	G	A	NM_001141974	c.1228C>T:p.R410W	missense	0	0	0	0.617:T	2	0	1	1	-
ATP13A2	1:17322974	C	T	NM_001141974	c.1198G>A:p.G400R	missense	-	-	-	0.989:D	0	0	0	1	-
ATP13A2	1:17322985	C	T	NM_001141974	c.1187G>A:p.C396Y	missense	0.0007	-	0.001	0.547:T	1	2	0	0	US
ATP13A2	1:17323539	C	T	NM_001141974	c.1156G>A:p.V386I	missense	0.0005	0	0.0005	0.523:T	1	0	0	0	US
ATP13A2	1:17323631	C	T	NM_001141974	c.1064G>A:p.G355E	missense	0.0027	0.0031	0.0021	0.661:T	15	12	14	9	CIP
ATP13A2	1:17323637	G	A	NM_001141974	c.1058C>T:p.P353L	missense	5.80E-05	-	0.0001	0.534:T	0	3	0	1	-
ATP13A2	1:17323646	G	A	NM_001141974	c.1049C>T:p.T350M	missense	0	0	0	0.829:D	0	1	0	0	US
ATP13A2	1:17326612	C	G	NM_001141974	c.918G>C:p.E306D	missense	0	-	0	0.296:T	1	0	0	0	-
ATP13A2	1:17326750	G	A	NM_001141974	c.883C>T:p.P295S	missense	-	-	-	0.462:T	1	0	0	0	-
ATP13A2	1:17326767	C	T	NM_001141974	c.866G>A:p.R289Q	missense	0	0.0006	0	0.153:T	1	0	0	0	B/LB
ATP13A2	1:17326768	G	A	NM_001141974	c.865C>T:p.R289W	missense	0	0	0	0.581:T	0	0	0	1	-
ATP13A2	1:17326902	G	T	NM_001141974	c.818C>A:p.T273N	missense	0.0008	0.0006	0.0014	0.446:T	3	7	4	9	-
ATP13A2	1:17326923	A	G	NM_001141974	c.797T>C:p.I266T	missense	-	-	-	0.763:D	1	0	1	0	-
ATP13A2	1:17326989	G	A	NM_001141974	c.731C>T:p.A244V	missense	0.0002	0.0006	0	0.774:D	1	1	1	0	CIP
ATP13A2	1:17326990	C	T	NM_001141974	c.730G>A:p.A244T	missense	0.0016	0.0012	0.0016	0.286:T	12	13	4	7	US
ATP13A2	1:17328532	G	T	NM_001141974	c.687C>A:p.D229E	missense	-	-	-	0.402:T	0	0	1	0	-
ATP13A2	1:17328824	C	A	NM_001141974	c.587G>T:p.R196L	missense	0.0005	-	0.0004	0.312:T	0	2	1	0	-
ATP13A2	1:17328836	A	T	NM_001141974	c.575T>A:p.V192D	missense	-	-	-	0.510:T	1	0	0	0	-
ATP13A2	1:17328837	C	A	NM_001141974	c.574G>T:p.V192F	missense	-	-	-	0.462:T	1	0	0	0	-
ATP13A2	1:17330837	A	T	NM_001141974	c.532T>A:p.Y178N	missense	-	-	-	0.395:T	1	0	0	0	-
ATP13A2	1:17330855	C	T	NM_001141974	c.514G>A:p.E172K	missense	0	-	-	0.214:T	1	0	0	0	-
ATP13A2	1:17330878	T	C	NM_001141974	c.491A>G:p.Q164R	missense	-	-	-	0.358:T	0	0	1	0	-

ATP13A2	1:17331192	C	T	NM_022089	c.472G>A:p.G158R	missense	0.0002	0.0006	0.0003	0.041:T	0	0	1	0	US
ATP13A2	1:17331203	G	A	NM_001141974	c.461C>T:p.A154V	missense	5.8E-05	0	0.0001	0.215:T	0	0	1	0	US
ATP13A2	1:17331257	G	A	NM_001141974	c.407C>T:p.A136V	missense	0.001	0.0006	0.0007	0.250:T	5	4	5	3	US
ATP13A2	1:17331296	G	C	NM_001141974	c.368C>G:p.S123C	missense	-	-	-	0.088:T	0	1	0	0	-
ATP13A2	1:17331925	G	A	NM_001141974	c.232C>T:p.R78W	missense	0	-	-	0.343:T	0	1	0	0	-
ATP13A2	1:17331930	C	T	NM_001141974	c.227G>A:p.R76Q	missense	0	-	0	0.296:T	1	0	0	0	-
ATP13A2	1:17331945	C	G	NM_001141974	c.212G>C:p.W71S	missense	0	-	0	0.572:T	1	0	0	0	US
ATP13A2	1:17331945	C	A	NM_001141974	c.212G>T:p.W71L	missense	0.0004	0	0.0002	0.402:T	0	0	0	1	US
ATP13A2	1:17332000	C	T	NM_001141974	c.157G>A:p.V53M	missense	0	0	0	0.062:T	1	2	2	0	US
ATP13A2	1:17332211	T	C	NM_001141974	c.73A>G:p.I25V	missense	5.81E-05	-	0.0001	0.043:T	1	0	0	0	-
ATP13A2	1:17332225	G	A	NM_001141974	c.59C>T:p.T20M	missense	0	-	0	0.330:T	0	1	0	0	-
ATP13A2	1:17332249	G	C	NM_001141974	c.35C>G:p.T12R	missense	0	-	-	0.365:T	0	0	1	0	-
FUCA1	1:24172270	G	A	NM_000147	c.1336C>T:p.Q446X	stopgain	-	-	0	0.262:T	0	0	0	1	-
FUCA1	1:24172587	G	A	NM_000147	c.1237C>T:p.P413S	missense	5.80E-05	-	0.0001	0.818:D	1	0	1	0	-
FUCA1	1:24172661	T	C	NM_000147	c.1163A>G:p.Y388C	missense	-	-	-	0.895:D	0	1	0	0	-
FUCA1	1:24175173	G	A	NM_000147	c.1126C>T:p.R376W	missense	0	0	0	0.888:D	0	0	1	0	-
FUCA1	1:24175293	G	C	NM_000147	c.1006C>G:p.L336V	missense	-	-	-	0.936:D	0	0	0	1	-
FUCA1	1:24180855	T	C	NM_000147	c.964A>G:p.I322V	missense	0.0003	-	0.0002	0.331:T	1	0	0	0	-
FUCA1	1:24180867	C	T	NM_000147	c.952G>A:p.E318K	missense	0.0001	-	0.0002	0.183:T	0	0	1	0	-
FUCA1	1:24180897	G	T	NM_000147	c.922C>A:p.R308S	missense	-	-	-	0.391:T	0	2	0	2	-
FUCA1	1:24181028	C	T	NM_000147	c.791G>A:p.R264Q	missense	0	-	0	0.930:D	0	1	0	0	p.R264X
FUCA1	1:24186391	T	C	NM_000147	c.665A>G:p.Y222C	missense	0	-	-	0.936:D	0	1	1	0	-
FUCA1	1:24189625	T	C	NM_000147	c.661A>G:p.S221G;-	missense	-	-	-	0.088:T	1	0	0	0	-
FUCA1	1:24189697	T	C	NM_000147	c.589A>G:p.K197E	missense	-	-	-	0.718:D	1	0	0	1	-
FUCA1	1:24189762	C	T	NM_000147	c.525-1G>A	splicing	-	-	-	0.550:T	1	0	0	0	-
FUCA1	1:24191987	C	T	NM_000147	c.518G>A:p.R173Q	missense	0.0003	0	0.0003	0.590:T	2	0	3	3	-
FUCA1	1:24192020	C	T	NM_000147	c.485G>A:p.R162Q	missense	0.0002	0.0006	0.0001	0.898:D	1	0	0	0	-
FUCA1	1:24192033	C	G	NM_000147	c.472G>C:p.V158L	missense	0.0001	-	-	0.192:T	0	0	1	0	-
FUCA1	1:24192068	G	C	NM_000147	c.437C>G:p.P146R	missense	0.0003	0.0006	0.0003	0.626:T	0	1	1	0	-
FUCA1	1:24194417	C	G	NM_000147	c.360G>C:p.W120C	missense	0.0014	0.0012	0.0036	0.859:D	3	3	4	1	-
FUCA1	1:24194429	G	C	NM_000147	c.348C>G:p.H116Q	missense	-	-	-	0.170:T	0	0	1	0	-
FUCA1	1:24194470	A	G	NM_000147	c.307T>C:p.Y103H	missense	-	-	-	0.972:D	0	1	0	0	-

FUCA1	1:24194494	C	T	NM_000147	c.283G>A:p.D95N	missense	-	-	-	0.005:T	0	1	0	0	-
FUCA1	1:24194514	T	A	NM_000147	c.263A>T:p.Q88L	missense	0	-	-	0.173:T	0	0	0	1	-
FUCA1	1:24194548	A	G	NM_000147	c.229T>C:p.F77L	missense	-	-	-	0.833:D	1	0	0	1	-
FUCA1	1:24194599	C	A	NM_000147	c.178G>T:p.V60L	missense	-	-	-	0.277:T	0	0	1	0	-
FUCA1	1:24194603	G	C	NM_000147	c.174C>G:p.F58L	missense	-	-	-	0.438:T	0	1	0	0	-
FUCA1	1:24194621	C	G	NM_000147	c.156G>C:p.W52C	missense	-	-	-	0.950:D	0	1	0	0	-
FUCA1	1:24194658	T	C	NM_000147	c.119A>G:p.D40G	missense	-	-	-	0.667:T	0	1	0	0	-
FUCA1	1:24194662	G	T	NM_000147	c.115C>A:p.P39T	missense	-	-	-	0.644:T	0	0	0	1	-
FUCA1	1:24194713	G	A	NM_000147	c.64C>T:p.L22F	missense	-	-	-	0.306:T	1	0	0	0	-
FUCA1	1:24194770	C	G	NM_000147	c.7G>C:p.A3P	missense	0	0	0	0.248:T	0	2	0	0	B/LB
PPT1	1:40539813	C	T	NM_000310	c.841G>A:p.V281M	missense	-	-	-	0.843:D	0	1	0	1	-
PPT1	1:40539851	C	T	NM_000310	c.803G>A:p.R268H	missense	0.0006	0.0012	0.0003	0.794:D	1	3	5	2	US
PPT1	1:40542519	T	C	NM_000310	c.793A>G:p.T265A	missense	-	-	-	0.381:T	1	0	0	0	-
PPT1	1:40555083	G	A	NM_000310	c.535C>T:p.R179C	missense	0	0	0	0.554:T	0	0	0	1	CIP
PPT1	1:40555097	T	C	NM_000310	c.521A>G:p.K174R	missense	0	-	0	0.340:T	1	0	0	0	-
PPT1	1:40555132	G	T	NM_000310	c.486C>A:p.F162L	missense	-	-	-	0.378:T	1	1	1	2	-
PPT1	1:40555144	G	T	NM_000310	c.474C>A:p.H158Q	missense	0.0004	0.0006	0.0002	0.499:T	2	1	2	2	LB
PPT1	1:40555161	G	C	NM_000310	c.457C>G:p.P153A	missense	0	-	-	0.842:D	0	0	1	0	-
PPT1	1:40557021	G	A	NM_000310	c.413C>T:p.S138L	missense	5.80E-05	-	0.0001	0.984:D	0	1	0	0	US
PPT1	1:40557741	C	G	NM_000310	c.338G>C:p.G113A	missense	-	-	-	0.925:D	0	1	0	0	-
PPT1	1:40557841	C	A	NM_000310	c.238G>T:p.V80L	missense	-	-	-	0.296:T	0	0	1	0	-
PPT1	1:40558135	T	C	NM_000310	c.169A>G:p.M57V	missense	-	-	-	0.866:D	0	1	0	0	-
PPT1	1:40562844	C	T	NM_000310	c.67G>A:p.A23T	missense	-	0	-	0.449:T	0	0	0	1	-
PPT1	1:40562861	C	G	NM_000310	c.50G>C:p.W17S	missense	-	-	-	0.452:T	0	0	1	0	-
PPT1	1:40562865	G	A	NM_000310	c.46C>T:p.P16S	missense	-	-	-	0.295:T	0	0	0	1	-
PPT1	1:40562894	C	G	NM_000310	c.17G>C:p.C6S	missense	5.81E-05	-	0.0002	0.296:T	2	0	0	1	US
PPT1	1:40562904	A	G	NM_000310	c.7T>C:p.S3P	missense	-	-	-	0.597:T	0	0	1	0	-
CTSK	1:150769327	T	C	NM_000396	c.938A>G:p.N313S	missense	-	-	-	0.613:T	0	0	1	0	-
CTSK	1:150769367	C	T	NM_000396	c.898G>A:p.E300K	missense	-	-	-	0.768:D	0	1	0	0	-
CTSK	1:150771750	C	T	NM_000396	c.785-1G>A	splicing	-	-	-	0.754:D	0	0	0	1	-
CTSK	1:150772119	C	T	NM_000396	c.685G>A:p.E229K	missense	0.0002	-	0.0005	0.392:T	0	0	1	0	US
CTSK	1:150772171	C	T	NM_000396	c.633G>A:p.M211I	missense	0.0003	-	0.0003	0.569:T	2	1	1	1	-

CTSK	1:150778434	C	T	NM_000396	c.302G>A:p.R101H	missense	5.8E-05	0	0	0.346:T	0	0	1	0	-
CTSK	1:150778468	T	C	NM_000396	c.268A>G:p.M90V	missense	0.0005	0.0012	0.0007	0.896:D	0	0	2	0	-
CTSK	1:150778663	T	A	NM_000396	c.158A>T:p.N53I	missense	0	-	-	0.873:D	0	0	0	3	-
CTSK	1:150779266	C	A	NM_000396	c.16G>T:p.V6F	missense	0	0	0	0.460:T	0	1	0	0	-
GBA	1:155204793	C	T	NM_000157	c.1604G>A:p.R535H	missense	0	0	0	0.876:D	1	0	1	0	P
GBA	1:155204812	G	A	NM_000157	c.1585C>T:p.H529Y	missense	-	-	-	0.807:D	1	0	0	0	-
GBA	1:155204890	A	G	NM_000157	c.1507T>C:p.S503P	missense	-	-	-	0.897:D	1	0	0	0	-
GBA	1:155204996	C	T	NM_000157	c.1495G>A:p.V499M	missense	0.0002	0	0.0003	0.953:D	2	0	0	0	US
GBA	1:155204996	C	A	NM_000157	c.1495G>T:p.V499L	missense	-	-	-	0.932:D	0	0	1	0	-
GBA	1:155205008	C	G	NM_000157	c.1483G>C:p.A495P	missense	0.0001	0	0.0002	0.929:D	4	2	1	1	B
GBA	1:155205025	AT	-	NM_000157	c.1465_1466del:p.M489Afs*15	frameshift deletion	-	-	-	-	1	0	0	0	-
GBA	1:155205029	GTGCCACTGCGTCCAG	-	NM_000157	c.1447_1462del:p.L483fs*0	frameshift deletion	-	-	-	-	1	0	0	0	-
GBA	1:155205039	G	-	NM_000157	c.1452delC:p.D484Efs*4	frameshift deletion	-	-	-	-	1	0	0	0	-
GBA	1:155205043	A	G	NM_000157	c.1448T>C:p.L483P	missense	0.0012	0.0012	0.0013	0.991:D	72	4	13	2	P
GBA	1:155205043	A	C	NM_000157	c.1448T>G:p.L483R	missense	5.80E-05	-	-	0.992:D	1	0	1	0	P/LP
GBA	1:155205043	A	-	NM_000157	c.1448delT:p.L483Rfs*5	frameshift deletion	-	-	-	-	1	0	0	0	-
GBA	1:155205088	T	C	NM_000157	c.1403A>G:p.E468G	missense	-	-	-	0.912:D	0	1	0	0	-
GBA	1:155205471	C	T	NM_000157	c.1388+1G>A	splicing	-	-	-	0.614:T	1	0	0	0	P
GBA	1:155205488	G	-	NM_000157	c.1372delC:p.H458Tfs*30	frameshift deletion	-	-	-	-	3	0	1	0	-
GBA	1:155205499	G	C	NM_000157	c.1361C>G:p.P454R	missense	-	-	-	0.991:D	1	0	0	0	P
GBA	1:155205518	C	G	NM_000157	c.1342G>C:p.D448H	missense	0	0.0006	0	0.910:D	3	0	2	0	P/LP
GBA	1:155205548	C	T	NM_000157	c.1312G>A:p.D438N	missense	-	-	-	0.717:D	1	0	2	0	P
GBA	1:155205560	G	A	NM_000157	c.1300C>T:p.R434C	missense	0	-	-	0.934:D	2	0	0	0	-
GBA	1:155205563	C	T	NM_000157	c.1297G>A:p.V433M	missense	-	-	-	0.910:D	0	0	2	0	p.V433L
GBA	1:155205568	T	C	NM_000157	c.1292A>G:p.N431S	missense	0.0001	-	0.0001	0.608:T	1	0	3	0	-
GBA	1:155205574	C	A	NM_000157	c.1286G>T:p.G429V	missense	-	-	-	0.999:D	0	0	1	0	-

GBA	1:155205581	C	T	NM_000157	c.1279G>A:p.E427K	missense	0	0	0	0.695:T	0	0	1	0	US
GBA	1:155205585	G	T	NM_000157	c.1275C>A:p.N425K	missense	-	-	-	0.737:D	0	0	1	0	-
GBA	1:155205592	G	A	NM_000157	c.1268C>T:p.A423V	missense	0	-	0	0.914:D	0	1	0	0	-
GBA	1:155205595	A	C	NM_000157	c.1265T>G:p.L422R	missense	-	-	-	0.997:D	2	0	1	0	-
GBA	1:155205599	T	C	NM_000157	c.1261A>G:p.N421D	missense	-	-	-	0.840:D	1	0	0	0	-
GBA	1:155205611	A	T	NM_000157	c.1249T>A:p.W417R	missense	-	-	-	0.978:D	1	0	0	0	-
GBA	1:155205614	C	T	NM_000157	c.1246G>A:p.G416S	missense	0	0	0	0.977:D	2	0	0	0	P
GBA	1:155205634	T	C	NM_000157	c.1226A>G:p.N409S	missense	0	0	0	0.533:T	0	0	1	0	P/LP
GBA	1:155206040	A	T	NM_000157	c.1220T>A:p.I407N	missense	-	-	-	0.844:D	1	0	0	0	-
GBA	1:155206043	A	T	NM_000157	c.1217T>A:p.I406N	missense	-	-	-	0.915:D	1	0	0	0	-
GBA	1:155206059	G	A	NM_000157	c.1201C>T:p.Q401X	stopgain	-	-	-	0.470:T	1	0	0	0	-
GBA	1:155206086	G	A	NM_000157	c.1174C>T:p.R392W	missense	0	-	-	0.825:D	0	1	0	0	P
GBA	1:155206088	A	C	NM_000157	c.1172T>G:p.V391G	missense	-	-	-	0.895:D	1	0	0	0	p.V391L
GBA	1:155206101	A	C	NM_000157	c.1159T>G:p.W387G	missense	5.8E-05	-	0.0001	0.833:D	0	0	0	1	-
GBA	1:155206104	A	G	NM_000157	c.1156T>C:p.F386L	missense	0.0006	-	0.0002	0.775:D	0	1	1	0	-
GBA	1:155206158	G	C	NM_000157	c.1102C>G:p.R368G	missense	-	-	-	0.850:D	0	0	1	0	CIP
GBA	1:155206167	C	T	NM_000157	c.1093G>A:p.E365K	missense	5.80E-05	0	0.0002	0.693:T	0	1	1	0	CIP
GBA	1:155206170	C	T	NM_000157	c.1090G>A:p.G364R	missense	0	-	-	0.902:D	0	1	0	0	LP
GBA	1:155206173	G	T	NM_000157	c.1087C>A:p.L363I	missense	-	-	-	0.810:D	0	1	0	0	-
GBA	1:155206181	T	C	NM_000157	c.1079A>G:p.K360R	missense	-	-	-	0.675:T	0	0	1	0	-
GBA	1:155206194	G	C	NM_000157	c.1066C>G:p.L356V	missense	-	-	-	0.839:D	0	1	0	0	-
GBA	1:155206248	G	T	NM_000157	c.1012C>A:p.P338T	missense	-	-	-	0.784:D	1	0	0	0	-
GBA	1:155207141	CCAGTGGG	-	NM_000157	c.983_990del:p.P328Rfs*11	frameshift deletion	-	-	-	-	1	0	0	0	-
GBA	1:155207193	T	C	NM_000157	c.938A>G:p.H313R	missense	-	-	0.0001	0.569:T	0	2	0	0	-
GBA	1:155207202	C	G	NM_000157	c.929G>C:p.S310T	missense	-	-	-	0.875:D	0	0	1	0	-
GBA	1:155207203	T	C	NM_000157	c.928A>G:p.S310G	missense	0.0002	0.0006	0	0.921:D	3	2	6	0	P/LP
GBA	1:155207209	C	T	NM_000157	c.922G>A:p.A308T	missense	0	-	0	0.829:D	0	1	0	0	-
GBA	1:155207224	G	T	NM_000157	c.907C>A:p.L303I	missense	5.80E-05	-	-	0.822:D	8	0	0	0	-
GBA	1:155207224	G	C	NM_000157	c.907C>G:p.L303V	missense	-	-	-	0.889:D	3	0	0	0	-
GBA	1:155207229	C	T	NM_000157	c.902G>A:p.R301H	missense	5.8E-05	0	0	0.895:D	0	0	1	1	US
GBA	1:155207230	G	A	NM_000157	c.901C>T:p.R301C	missense	0.0002	-	0.0001	0.898:D	1	0	0	0	-

GBA	1:155207255	A	-	NM_000157	c.876delT:p.E293Nfs*10	frameshift deletion	-	-	-	-	1	0	0	0	-
GBA	1:155207272	A	G	NM_000157	c.859T>C:p.C287R	missense	-	-	-	0.888:D	0	0	1	0	-
GBA	1:155207330	C	T	NM_000157	c.801G>A:p.W267X	stopgain	-	-	-	0.669:T	2	0	0	0	-
GBA	1:155207355	T	C	NM_000157	c.776A>G:p.Y259C	missense	-	-	-	0.948:D	1	0	0	0	-
GBA	1:155207366	G	C	NM_000157	c.765C>G:p.F255L	missense	-	-	-	0.937:D	1	0	0	0	p.F255Y
GBA	1:155207370	C	G	NM_000157	c.762-1G>C	splicing	5.82E-05	0.0006	-	0.552:T	4	2	1	0	-
GBA	1:155207932	A	T	NM_000157	c.754T>A:p.F252I	missense	0.0001	0	0	0.949:D	4	0	0	0	P
GBA	1:155207942	C	T	NM_000157	c.744G>A:p.W248X	stopgain	-	-	-	0.655:T	3	0	1	0	-
GBA	1:155207965	C	T	NM_000157	c.721G>A:p.G241R	missense	5.80E-05	-	0	0.969:D	5	0	4	0	P
GBA	1:155207974	C	T	NM_000157	c.712G>A:p.G238R	missense	-	-	-	0.963:D	1	0	0	0	-
GBA	1:155207979	A	G	NM_000157	c.707T>C:p.L236P	missense	-	-	-	0.936:D	2	0	0	0	-
GBA	1:155207983	A	G	NM_000157	c.703T>C:p.S235P	missense	0	0	0	0.904:D	1	0	0	0	P
GBA	1:155208001	C	T	NM_000157	c.685G>A:p.A229T	missense	-	-	-	0.907:D	1	0	0	0	-
GBA	1:155208005	A	C	NM_000157	c.681T>G:p.N227K	missense	0	0	0	0.757:D	4	0	1	0	P
GBA	1:155208006	T	C	NM_000157	c.680A>G:p.N227S	missense	0.0002	0	0.0001	0.636:T	7	0	6	0	P/LP
GBA	1:155208056	G	-	NM_000157	c.630delC:p.V211Ffs*19	frameshift deletion	-	-	-	-	1	0	0	0	-
GBA	1:155208060	C	T	NM_000157	c.626G>A:p.R209H	missense	0	0	0	0.443:T	0	1	0	0	p.R209C
GBA	1:155208081	C	T	NM_000157	c.605G>A:p.R202Q	missense	0.003	0.0025	0.004	0.352:T	12	16	14	3	B
GBA	1:155208375	T	C	NM_000157	c.521A>G:p.Y174C	missense	5.8E-05	-	0.0001	0.955:D	0	0	1	0	LP
GBA	1:155208409	C	T	NM_000157	c.487G>A:p.A163T	missense	-	-	-	0.799:D	0	0	2	0	P
GBA	1:155208412	T	C	NM_000157	c.484A>G:p.M162V	missense	0	-	0	0.889:D	0	0	2	0	-
GBA	1:155208414	G	C	NM_000157	c.482C>G:p.P161R	missense	-	-	-	0.940:D	1	0	0	0	p.P161S
GBA	1:155208420	C	T	NM_000157	c.476G>A:p.R159Q	missense	0	-	-	0.996:D	2	0	0	0	LP
GBA	1:155208421	G	A	NM_000157	c.475C>T:p.R159W	missense	5.8E-05	0	-	0.972:D	0	0	1	0	P
GBA	1:155209405	A	T	NM_000157	c.454+2T>A	splicing	-	-	-	0.452:T	1	0	0	0	-
GBA	1:155209424	G	A	NM_000157	c.437C>T:p.S146L	missense	0	-	0	0.860:D	0	0	1	0	US
GBA	1:155209431	G	T	NM_000157	c.430C>A:p.L144I	missense	-	-	-	0.673:T	0	1	0	0	-
GBA	1:155209461	C	A	NM_000157	c.400G>T:p.A134S	missense	-	-	-	0.390:T	2	0	0	0	-
GBA	1:155209676	C	A	NM_000157	c.307+1G>T	splicing	-	-	-	0.528:T	0	0	1	0	-
GBA	1:155209685	G	A	NM_000157	c.299C>T:p.T100M	missense	0	-	0	0.710:D	1	0	0	0	-

GBA	1:155209690	-	T	NM_000157	c.293dupA:p.N98Kfs*47	frameshift insertion	-	-	-	-	1	0	0	0	-
GBA	1:155209725	G	A	NM_000157	c.259C>T:p.R87W	missense	0	-	0	0.929:D	0	0	0	1	P
GBA	1:155209728	G	A	NM_000157	c.256C>T:p.R86X	stopgain	-	-	-	0.601:T	1	0	0	0	-
GBA	1:155209780	-	G	NM_000157	c.203dupC:p.T69Dfs*11	frameshift insertion	0	-	-	-	1	0	0	0	-
GBA	1:155209814	C	G	NM_000157	c.170G>C:p.C57S	missense	-	-	-	0.910:D	0	0	1	0	-
GBA	1:155209817	AC	-	NM_000157	c.166_167del:p.V56Lfs*6	frameshift deletion	-	-	-	-	1	0	0	0	-
GBA	1:155209827	C	-	NM_000157	c.157delG:p.V53Wfs*37	frameshift deletion	-	-	-	-	1	0	0	0	-
GBA	1:155209836	A	G	NM_000157	c.148T>C:p.Y50H	missense	0.0002	-	0.0001	0.385:T	1	0	0	0	-
GBA	1:155209860	G	C	NM_000157	c.124C>G:p.P42A	missense	-	-	-	0.585:T	0	1	0	0	-
GBA	1:155210420	C	T	NM_000157	c.115+1G>A	splicing	0.0001	0	0.0002	0.479:T	3	0	1	0	P/LP
GBA	1:155210420	C	A	NM_000157	c.115+1G>T	splicing	-	-	-	0.481:T	1	0	0	0	c.115+1G>A
GBA	1:155210421	C	G	NM_000157	c.115G>C:p.G39R	missense	-	-	-	0.702:D	3	0	0	0	-
GBA	1:155210438	G	A	NM_000157	c.98C>T:p.A33V	missense	0.0003	0.0006	0.0002	0.512:T	1	0	0	0	-
GBA	1:155210478	T	C	NM_000157	c.58A>G:p.I20V	missense	0.002	0.0006	0.0038	0.147:T	0	1	1	0	LB
GBA	1:155210496	G	T	NM_000157	c.40C>A:p.P14T	missense	-	-	-	0.350:T	0	0	1	0	-
GBA	1:155210876	C	A	NM_000157	c.27+1G>T	splicing	-	-	-	0.409:T	0	0	1	0	-
LYST	1:235826253	T	C	NM_000081	c.11393A>G:p.Y3798C	missense	5.80E-05	-	-	0.811:D	1	0	0	0	US
LYST	1:235826292	C	T	NM_000081	c.11354G>A:p.R3785H	missense	0.0001	-	0.0001	0.603:T	1	0	0	0	US
LYST	1:235826326	T	A	NM_000081	c.11320A>T:p.T3774S	missense	0.0001	-	-	0.363:T	1	2	0	2	-
LYST	1:235826363	A	C	NM_000081	c.11283T>G:p.C3761W	missense	-	-	-	0.870:D	1	0	0	0	-
LYST	1:235827789	C	T	NM_000081	c.11171G>A:p.G3724E	missense	-	-	-	0.908:D	0	1	0	0	-
LYST	1:235840421	A	C	NM_000081	c.11008T>G:p.S3670A	missense	-	-	-	0.153:T	0	0	1	0	-
LYST	1:235840424	T	C	NM_000081	c.11005A>G:p.T3669A	missense	-	-	-	0.567:T	0	0	0	1	-
LYST	1:235840842	T	A	NM_000081	c.10878A>T:p.K3626N	missense	-	-	-	0.502:T	5	0	0	1	-
LYST	1:235840882	T	C	NM_000081	c.10838A>G:p.Y3613C	missense	0	0	0	0.864:D	1	0	0	0	US

LYST	1:235850322	T	C	NM_000081	c.10727A>G:p.D3576G	missense	-	-	-	0.898:D	0	0	1	0	-
LYST	1:235856693	C	T	NM_000081	c.10658G>A:p.S3553N	missense	5.80E-05	-	-	0.226:T	0	1	0	0	-
LYST	1:235856696	T	G	NM_000081	c.10655A>C:p.Q3552P	missense	-	-	-	0.724:D	1	0	0	0	-
LYST	1:235856748	C	A	NM_000081	c.10603G>T:p.A3535S	missense	-	-	-	0.613:T	1	0	0	0	-
LYST	1:235856765	G	A	NM_000081	c.10586C>T:p.T3529M	missense	-	-	-	0.779:D	0	1	0	0	-
LYST	1:235860397	T	C	NM_000081	c.10550A>G:p.Y3517C	missense	5.80E-05	0.0006	0.0001	0.838:D	1	0	0	0	-
LYST	1:235860421	C	T	NM_000081	c.10526G>A:p.R3509Q	missense	0.0008	0.0012	0.001	0.096:T	6	4	3	3	US
LYST	1:235866048	G	A	NM_000081	c.10373C>T:p.P3458L	missense	0	-	-	0.789:D	0	1	0	0	-
LYST	1:235866049	G	A	NM_000081	c.10372C>T:p.P3458S	missense	5.8E-05	-	0	0.635:T	0	0	0	1	-
LYST	1:235866094	A	G	NM_000081	c.10327T>C:p.F3443L	missense	-	-	-	0.138:T	1	0	0	0	-
LYST	1:235866104	C	A	NM_000081	c.10317G>T:p.L3439F	missense	0.0002	-	0.0001	0.431:T	1	0	0	0	-
LYST	1:235866135	A	G	NM_000081	c.10286T>C:p.I3429T	missense	-	-	-	0.352:T	0	0	1	0	-
LYST	1:235866136	T	C	NM_000081	c.10285A>G:p.I3429V	missense	0.0003	0.0012	0.0001	0.102:T	1	0	0	0	-
LYST	1:235866177	A	C	NM_000081	c.10244T>G:p.F3415C	missense	-	-	-	0.989:D	0	0	0	1	-
LYST	1:235866243	T	A	NM_000081	c.10178A>T:p.D3393V	missense	0	-	0	0.906:D	0	1	0	0	-
LYST	1:235866246	T	G	NM_000081	c.10175A>C:p.E3392A	missense	-	-	0.0001	0.839:D	2	0	0	0	-
LYST	1:235866252	G	A	NM_000081	c.10169C>T:p.A3390V	missense	-	-	-	0.630:T	0	1	0	0	-
LYST	1:235875386	T	C	NM_000081	c.9896A>G:p.Y3299C	missense	0	0	0	0.945:D	0	0	1	0	US
LYST	1:235875462	A	T	NM_000081	c.9820T>A:p.S3274T	missense	-	-	-	0.899:D	1	1	0	1	-
LYST	1:235878560	G	A	NM_000081	c.9725C>T:p.T3242I	missense	-	-	-	0.863:D	1	0	0	0	-
LYST	1:235878635	T	A	NM_000081	c.9650A>T:p.K3217I	missense	-	-	-	0.743:D	0	0	1	0	-
LYST	1:235880031	C	T	NM_000081	c.9608G>A:p.R3203H	missense	0	-	0	0.697:T	0	0	2	0	US
LYST	1:235884007	C	T	NM_000081	c.9514G>A:p.D3172N	missense	0.0001	0.0012	0.0001	0.607:T	0	1	0	1	-
LYST	1:235884048	T	C	NM_000081	c.9473A>G:p.N3158S	missense	0	-	0	0.495:T	0	0	1	0	US
LYST	1:235884079	G	A	NM_000081	c.9442C>T:p.H3148Y	missense	5.8E-05	-	0.0001	0.820:D	0	0	1	1	-
LYST	1:235884183	T	C	NM_000081	c.9338A>G:p.N3113S	missense	0.0002	0	0.0001	0.233:T	0	1	0	0	US
LYST	1:235887384	C	T	NM_000081	c.9259G>A:p.V3087I	missense	-	-	-	0.475:T	0	1	0	0	-
LYST	1:235892949	C	G	NM_000081	c.9053G>C:p.R3018P	missense	-	-	-	0.744:D	1	0	0	0	-
LYST	1:235896896	T	C	NM_000081	c.8708A>G:p.K2903R	missense	-	-	-	0.652:T	1	0	0	0	-
LYST	1:235896918	G	A	NM_000081	c.8686C>T:p.L2896F	missense	-	-	-	0.436:T	0	0	0	1	-
LYST	1:235896924	C	T	NM_000081	c.8680G>A:p.V2894M	missense	0	0	-	0.625:T	1	0	0	0	-
LYST	1:235896980	C	T	NM_000081	c.8624G>A:p.R2875H	missense	0.0025	0.0031	0.0024	0.767:D	10	5	5	9	LB

LYST	1:235897194	A	G	NM_000081	c.8548T>C:p.Y2850H	missense	-	-	-	0.414:T	0	0	2	0	-
LYST	1:235897804	G	T	NM_000081	c.8514C>A:p.D2838E	missense	0.0003	-	0.0003	0.188:T	4	1	1	0	-
LYST	1:235897815	T	C	NM_000081	c.8503A>G:p.T2835A	missense	0	-	0	0.201:T	1	0	0	0	US
LYST	1:235897894	T	G	NM_000081	c.8424A>C:p.E2808D	missense	0.0001	-	0.0002	0.076:T	2	0	0	0	-
LYST	1:235897950	T	G	NM_000081	c.8368A>C:p.K2790Q	missense	0.01	0.0117	0.0105	0.636:T	50	55	51	28	B/LB
LYST	1:235904729	G	C	NM_000081	c.8351C>G:p.S2784C	missense	0.0001	-	0.0001	0.279:T	1	0	0	0	-
LYST	1:235904928	C	T	NM_000081	c.8152G>A:p.G2718S	missense	-	-	-	0.203:T	0	1	0	0	-
LYST	1:235907419	C	T	NM_000081	c.8011G>A:p.E2671K	missense	-	-	-	0.557:T	0	1	0	0	-
LYST	1:235907433	A	T	NM_000081	c.7997T>A:p.I2666N	missense	0.0003	-	0.0003	0.674:T	1	1	0	0	US
LYST	1:235907436	T	C	NM_000081	c.7994A>G:p.D2665G	missense	0.0008	0.0012	0.0009	0.779:D	4	1	2	2	US
LYST	1:235909689	G	A	NM_000081	c.7919C>T:p.A2640V	missense	0.0002	-	0.0001	0.227:T	0	2	2	3	-
LYST	1:235909690	C	A	NM_000081	c.7918G>T:p.A2640S	missense	-	-	-	0.221:T	0	1	0	0	-
LYST	1:235909698	G	A	NM_000081	c.7910C>T:p.T2637M	missense	0	-	0	0.349:T	1	0	2	0	-
LYST	1:235909738	G	A	NM_000081	c.7870C>T:p.R2624W	missense	0	0	0	0.821:D	0	1	0	0	CIP
LYST	1:235914508	G	A	NM_000081	c.7780+2C>T	splicing	0	-	0	0.445:T	0	1	1	0	CIP
LYST	1:235914542	T	C	NM_000081	c.7748A>G:p.H2583R	missense	0.0001	-	-	0.318:T	0	1	1	2	-
LYST	1:235914612	T	C	NM_000081	c.7678A>G:p.I2560V	missense	0.0007	-	0.0005	0.308:T	3	1	0	1	-
LYST	1:235914629	T	C	NM_000081	c.7661A>G:p.Q2554R	missense	0.0003	-	0.0001	0.622:T	2	1	4	2	US
LYST	1:235914654	C	T	NM_000081	c.7636G>A:p.V2546I	missense	0.0001	-	0	0.103:T	0	1	0	1	-
LYST	1:235915321	T	G	NM_000081	c.7611A>C:p.K2537N	missense	-	-	-	0.443:T	0	0	1	0	-
LYST	1:235915397	C	G	NM_000081	c.7535G>C:p.C2512S	missense	-	-	-	0.793:D	1	0	0	0	-
LYST	1:235915415	G	A	NM_000081	c.7517C>T:p.A2506V	missense	-	-	-	0.636:T	0	1	0	0	-
LYST	1:235915467	G	T	NM_000081	c.7465C>A:p.P2489T	missense	-	-	-	0.572:T	0	0	1	0	-
LYST	1:235916368	G	C	NM_000081	c.7436C>G:p.A2479G	missense	-	-	-	0.473:T	0	2	0	1	-
LYST	1:235916431	C	T	NM_000081	c.7373G>A:p.C2458Y	missense	-	-	-	0.860:D	0	0	1	0	-
LYST	1:235918813	T	A	NM_000081	c.7194A>T:p.E2398D	missense	0.0001	-	0.0001	0.444:T	0	1	0	0	-
LYST	1:235918815	C	T	NM_000081	c.7192G>A:p.E2398K	missense	0	0	0	0.731:D	1	0	0	0	US
LYST	1:235918838	T	G	NM_000081	c.7169A>C:p.Q2390P	missense	-	-	-	0.785:D	0	1	1	0	-
LYST	1:235918847	C	T	NM_000081	c.7160G>A:p.R2387Q	missense	0	-	0	0.108:T	1	0	0	0	US
LYST	1:235918924	T	A	NM_000081	c.7083A>T:p.R2361S	missense	-	-	-	0.860:D	0	0	0	1	-
LYST	1:235918940	T	C	NM_000081	c.7067A>G:p.D2356G	missense	0.0001	-	0.0001	0.388:T	1	2	0	0	-
LYST	1:235920622	C	T	NM_000081	c.7018G>A:p.V2340I	missense	5.8E-05	-	0	0.236:T	0	0	0	1	US

LYST	1:235920733	T	G	NM_000081	c.6907A>C:p.I2303L	missense	-	-	-	0.486:T	1	0	1	0	-
LYST	1:235922312	C	A	NM_000081	c.6841G>T:p.G2281C	missense	-	-	-	0.363:T	0	1	0	0	-
LYST	1:235922320	T	C	NM_000081	c.6833A>G:p.Y2278C	missense	0	0	0	0.323:T	1	0	0	0	US
LYST	1:235922357	C	G	NM_000081	c.6796G>C:p.V2266L	missense	0.0002	-	0.0002	0.157:T	1	3	0	0	US
LYST	1:235922371	C	T	NM_000081	c.6782G>A:p.R2261H	missense	0	0	0	0.566:T	0	1	0	0	B
LYST	1:235922378	C	T	NM_000081	c.6775G>A:p.V2259I	missense	0.0009	-	0.001	0.007:T	9	2	2	3	US
LYST	1:235922450	A	C	NM_000081	c.6703T>G:p.S2235A	missense	-	-	-	0.395:T	0	1	0	0	US
LYST	1:235922476	C	T	NM_000081	c.6677G>A:p.R2226Q	missense	-	-	-	0.688:T	1	1	1	0	-
LYST	1:235922479	C	T	NM_000081	c.6674G>A:p.R2225H	missense	0	0	0	0.095:T	0	1	1	0	-
LYST	1:235922489	A	G	NM_000081	c.6664T>C:p.S2222P	missense	-	-	-	0.245:T	0	0	1	0	-
LYST	1:235922518	C	G	NM_000081	c.6635G>C:p.R2212T	missense	-	-	-	0.391:T	0	0	1	0	-
LYST	1:235922535	C	G	NM_000081	c.6618G>C:p.Q2206H	missense	-	-	-	0.137:T	1	0	0	0	-
LYST	1:235922623	A	G	NM_000081	c.6530T>C:p.M2177T	missense	-	-	-	0.170:T	0	0	0	1	-
LYST	1:235922642	C	G	NM_000081	c.6511G>C:p.A2171P	missense	-	-	-	0.765:D	0	1	0	1	-
LYST	1:235922749	A	G	NM_000081	c.6404T>C:p.I2135T	missense	0	-	0	0.210:T	0	0	0	1	-
LYST	1:235922791	A	G	NM_000081	c.6362T>C:p.L2121P	missense	-	-	-	0.532:T	0	0	0	1	-
LYST	1:235922848	C	T	NM_000081	c.6305G>A:p.R2102H	missense	0	-	0.0001	0.265:T	0	0	0	1	US
LYST	1:235926075	C	A	NM_000081	c.6198G>T:p.M2066I	missense	-	-	-	0.160:T	1	0	0	0	-
LYST	1:235926115	A	G	NM_000081	c.6158T>C:p.M2053T	missense	-	-	0.0001	0.159:T	0	0	1	0	-
LYST	1:235926116	T	C	NM_000081	c.6157A>G:p.M2053V	missense	0.0005	-	0.0002	0.068:T	1	2	2	1	-
LYST	1:235926124	C	T	NM_000081	c.6149G>A:p.R2050Q	missense	0.0005	0.0006	0.0005	0.088:T	1	1	4	2	US
LYST	1:235929410	A	T	NM_000081	c.6090T>A:p.N2030K	missense	-	-	-	0.319:T	0	0	1	0	-
LYST	1:235929421	C	T	NM_000081	c.6079G>A:p.V2027I	missense	0	0	0	0.311:T	1	0	0	0	-
LYST	1:235929421	C	G	NM_000081	c.6079G>C:p.V2027L	missense	0.0005	0.0019	0.0006	0.587:T	2	1	3	1	US
LYST	1:235929469	T	C	NM_000081	c.6031A>G:p.I2011V	missense	5.80E-05	-	-	0.112:T	0	2	0	3	-
LYST	1:235929543	C	T	NM_000081	c.5957G>A:p.R1986Q	missense	0.0009	0.0006	0.0008	0.103:T	3	1	3	0	US
LYST	1:235937263	T	A	NM_000081	c.5663A>T:p.D1888V	missense	5.81E-05	-	0.0001	0.428:T	1	0	0	0	-
LYST	1:235938249	G	C	NM_000081	c.5598C>G:p.I1866M	missense	0	-	0	0.184:T	0	1	0	0	US
LYST	1:235938269	T	C	NM_000081	c.5578A>G:p.M1860V	missense	0	0	-	0.564:T	0	1	0	1	US
LYST	1:235938283	C	T	NM_000081	c.5564G>A:p.C1855Y	missense	-	-	-	0.460:T	0	0	0	1	-
LYST	1:235938328	G	A	NM_000081	c.5519C>T:p.S1840L	missense	0	-	0	0.632:T	0	3	0	1	-
LYST	1:235938388	-	A	NM_000081	c.5461-2->T	splicing	0.0112	0.0006	0.0124	-	4	0	5	1	-

LYST	1:235938388	-	AA	NM_000081	c.5461-2->TT	splicing	0.0127	0.0038	0.0097	-	10	3	17	9	-
LYST	1:235940388	C	T	NM_000081	c.5435G>A:p.G1812D	missense	5.8E-05	0.0006	-	0.860:D	0	0	3	2	-
LYST	1:235940415	C	G	NM_000081	c.5408G>C:p.G1803A	missense	5.81E-05	-	0	0.265:T	0	1	0	0	-
LYST	1:235940508	T	C	NM_000081	c.5315A>G:p.Q1772R	missense	0.0003	0.0006	0.0005	0.533:T	5	2	2	2	-
LYST	1:235940533	C	T	NM_000081	c.5290G>A:p.G1764S	missense	0.0002	-	0.0003	0.394:T	7	2	2	1	-
LYST	1:235944200	T	C	NM_000081	c.5179A>G:p.M1727V	missense	-	-	-	0.498:T	1	0	0	1	-
LYST	1:235944304	T	C	NM_000081	c.5075A>G:p.N1692S	missense	0	-	0	0.254:T	0	1	0	1	-
LYST	1:235945278	A	G	NM_000081	c.4972T>C:p.F1658L	missense	0.0003	0.0012	0.0001	0.126:T	1	0	0	0	-
LYST	1:235945278	A	C	NM_000081	c.4972T>G:p.F1658V	missense	0.0008	0.0012	0.0009	0.105:T	0	0	1	0	US
LYST	1:235945298	C	G	NM_000081	c.4952G>C:p.C1651S	missense	0.0003	-	0.0004	0.687:T	1	0	0	0	-
LYST	1:235945361	A	G	NM_000081	c.4889T>C:p.M1630T	missense	-	-	-	0.127:T	2	0	0	0	-
LYST	1:235950519	T	C	NM_000081	c.4843A>G:p.I1615V	missense	-	-	-	0.165:T	2	0	0	0	-
LYST	1:235950551	T	C	NM_000081	c.4811A>G:p.Q1604R	missense	-	-	-	0.191:T	0	0	0	1	-
LYST	1:235950554	G	A	NM_000081	c.4808C>T:p.P1603L	missense	-	-	-	0.497:T	0	0	1	0	US
LYST	1:235950672	C	T	NM_000081	c.4690G>A:p.V1564M	missense	0.0001	-	0.0002	0.083:T	1	2	1	2	-
LYST	1:235952019	T	C	NM_000081	c.4670A>G:p.N1557S	missense	0	-	-	0.038:T	1	0	0	0	US
LYST	1:235952029	C	G	NM_000081	c.4660G>C:p.D1554H	missense	-	-	-	0.671:T	0	1	0	0	-
LYST	1:235952050	A	T	NM_000081	c.4639T>A:p.L1547M	missense	-	-	-	0.642:T	0	0	1	0	-
LYST	1:235952095	T	C	NM_000081	c.4594A>G:p.I1532V	missense	-	-	-	0.256:T	0	1	0	0	-
LYST	1:235952100	C	G	NM_000081	c.4589G>C:p.R1530T	missense	0.0007	0.0006	0.0004	0.192:T	4	5	6	4	US
LYST	1:235952111	A	T	NM_000081	c.4578T>A:p.N1526K	missense	0.0032	0.0049	0.0028	0.187:T	11	15	10	5	US
LYST	1:235952116	T	C	NM_000081	c.4573A>G:p.I1525V	missense	0.0001	0.0006	0.0001	0.114:T	3	2	2	1	-
LYST	1:235952131	C	G	NM_000081	c.4558G>C:p.E1520Q	missense	-	-	-	0.089:T	0	1	0	0	-
LYST	1:235955008	C	T	NM_000081	c.4534G>A:p.D1512N	missense	-	-	-	0.285:T	0	1	0	0	-
LYST	1:235955013	C	G	NM_000081	c.4529G>C:p.S1510T	missense	-	-	-	0.304:T	2	0	1	0	US
LYST	1:235955057	C	G	NM_000081	c.4485G>C:p.K1495N	missense	-	-	-	0.210:T	0	0	1	0	-
LYST	1:235955097	T	C	NM_000081	c.4445A>G:p.E1482G	missense	-	-	-	0.655:T	0	0	0	1	-
LYST	1:235955205	C	T	NM_000081	c.4337G>A:p.R1446Q	missense	0	0	0	0.114:T	4	0	0	0	CIP
LYST	1:235955271	C	T	NM_000081	c.4271G>A:p.G1424D	missense	0.0005	-	0.0003	0.648:T	0	0	2	0	US
LYST	1:235955293	T	A	NM_000081	c.4249A>T:p.I1417F	missense	-	-	-	0.471:T	1	0	0	0	-
LYST	1:235955423	T	G	NM_000081	c.4119A>C:p.K1373N	missense	-	-	-	0.142:T	1	0	0	0	-
LYST	1:235963655	A	G	NM_000081	c.3971T>C:p.L1324S	missense	-	-	-	0.563:T	0	1	0	0	-

LYST	1:235964177	C	T	NM_000081	c.3933G>A:p.M1311I	missense	0	-	-	0.153:T	0	1	0	0	-
LYST	1:235964242	C	A	NM_000081	c.3868G>T:p.A1290S	missense	-	-	-	0.087:T	0	1	0	1	-
LYST	1:235964257	T	G	NM_000081	c.3853A>C:p.K1285Q	missense	-	-	-	0.165:T	0	0	1	0	-
LYST	1:235964355	C	A	NM_000081	c.3755G>T:p.S1252I	missense	-	-	-	0.161:T	0	0	1	0	-
LYST	1:235964380	C	G	NM_000081	c.3730G>C:p.E1244Q	missense	-	-	-	0.569:T	0	0	1	1	-
LYST	1:235966223	C	T	NM_000081	c.3697G>A:p.E1233K	missense	0.0004	0	0.0005	0.207:T	3	2	1	0	US
LYST	1:235966289	T	G	NM_000081	c.3631A>C:p.S1211R	missense	0.0001	-	0.0001	0.240:T	0	1	0	1	-
LYST	1:235966325	C	G	NM_000081	c.3595G>C:p.D1199H	missense	-	-	-	0.441:T	0	0	1	0	-
LYST	1:235966334	G	C	NM_000081	c.3586C>G:p.Q1196E	missense	-	-	-	0.511:T	1	1	0	1	-
LYST	1:235967851	C	T	NM_000081	c.3508G>A:p.G1170R	missense	0	-	0	0.758:D	1	0	0	0	-
LYST	1:235969050	G	T	NM_000081	c.3386C>A:p.P1129H	missense	-	-	-	0.403:T	2	1	0	0	-
LYST	1:235969066	T	C	NM_000081	c.3370A>G:p.M1124V	missense	-	-	-	0.057:T	0	1	0	0	US
LYST	1:235969074	T	C	NM_000081	c.3362A>G:p.Q1121R	missense	0.0002	-	0.0002	0.417:T	1	0	1	0	US
LYST	1:235969078	T	C	NM_000081	c.3358A>G:p.S1120G	missense	0.0001	-	-	0.144:T	2	0	0	0	-
LYST	1:235969258	C	T	NM_000081	c.3178G>A:p.D1060N	missense	-	-	-	0.107:T	1	0	0	0	-
LYST	1:235969328	T	G	NM_000081	c.3108A>C:p.E1036D	missense	-	-	-	0.117:T	0	0	1	0	-
LYST	1:235969329	T	G	NM_000081	c.3107A>C:p.E1036A	missense	0.0008	0.0019	0.0008	0.316:T	2	1	1	0	-
LYST	1:235969342	T	C	NM_000081	c.3094A>G:p.R1032G	missense	-	-	-	0.125:T	2	0	0	0	-
LYST	1:235969455	A	G	NM_000081	c.2981T>C:p.I994T	missense	0	-	-	0.791:D	1	0	1	0	-
LYST	1:235969464	T	C	NM_000081	c.2972A>G:p.H991R	missense	0.0001	-	-	0.231:T	1	0	0	0	-
LYST	1:235969495	A	C	NM_000081	c.2941T>G:p.F981V	missense	-	-	-	0.900:D	0	0	1	0	-
LYST	1:235969623	C	A	NM_000081	c.2813G>T:p.S938I	missense	0.0002	0.0006	0.0005	0.158:T	0	0	2	1	-
LYST	1:235969639	C	T	NM_000081	c.2797G>A:p.A933T	missense	-	-	-	0.341:T	0	0	1	0	-
LYST	1:235969674	T	C	NM_000081	c.2762A>G:p.N921S	missense	0	-	0	0.198:T	1	0	0	0	-
LYST	1:235969687	T	C	NM_000081	c.2749A>G:p.R917G	missense	0.0005	0.0006	0.0005	0.403:T	2	0	1	0	-
LYST	1:235969711	C	T	NM_000081	c.2725G>A:p.V909I	missense	0	0	0	0.188:T	1	0	0	0	US
LYST	1:235969779	C	T	NM_000081	c.2657G>A:p.R886Q	missense	0	-	0	0.036:T	1	0	0	0	-
LYST	1:235969789	G	C	NM_000081	c.2647C>G:p.P883A	missense	0.0018	0.0012	0.0014	0.534:T	4	0	3	0	US
LYST	1:235969962	A	G	NM_000081	c.2474T>C:p.I825T	missense	0.0002	-	0.0001	0.348:T	1	0	1	0	-
LYST	1:235969981	C	T	NM_000081	c.2455G>A:p.A819T	missense	0	0	0	0.385:T	0	0	1	0	US
LYST	1:235971756	T	A	NM_000081	c.2362A>T:p.R788W	missense	-	-	-	0.863:D	1	2	0	0	-
LYST	1:235971863	A	T	NM_000081	c.2255T>A:p.L752Q	missense	0.0003	-	0.0002	0.358:T	0	1	1	0	US

LYST	1:235971946	T	C	NM_000081	c.2172A>G:p.I724M	missense	0.0011	0.0006	0.0006	0.052:T	2	3	1	0	US
LYST	1:235971950	T	C	NM_000081	c.2168A>G:p.N723S	missense	0	0	-	0.401:T	0	1	0	0	US
LYST	1:235972087	G	C	NM_000081	c.2031C>G:p.I677M	missense	0.0001	-	0.0001	0.576:T	1	0	0	2	-
LYST	1:235972143	A	G	NM_000081	c.1975T>C:p.C659R	missense	0.0003	0.0006	0.0003	0.011:T	0	1	3	0	US
LYST	1:235972173	G	C	NM_000081	c.1945C>G:p.Q649E	missense	-	-	-	0.018:T	1	0	0	0	-
LYST	1:235972216	T	G	NM_000081	c.1902A>C:p.K634N	missense	0.0001	-	0.0002	0.047:T	0	0	1	0	-
LYST	1:235972290	G	A	NM_000081	c.1828C>T:p.H610Y	missense	0	-	-	0.437:T	0	0	1	0	-
LYST	1:235972394	G	A	NM_000081	c.1724C>T:p.S575L	missense	0	-	0	0.450:T	1	0	0	0	US
LYST	1:235972427	G	A	NM_000081	c.1691C>T:p.A564V	missense	0	-	0	0.267:T	0	1	0	0	-
LYST	1:235972470	T	C	NM_000081	c.1648A>G:p.I550V	missense	0	-	0	0.305:T	1	0	0	0	-
LYST	1:235972577	C	T	NM_000081	c.1541G>A:p.R514Q	missense	0.0007	-	0.0003	0.590:T	4	0	2	2	US
LYST	1:235972637	G	A	NM_000081	c.1481C>T:p.S494L	missense	0	0	0	0.803:D	0	0	2	0	US
LYST	1:235972693	A	C	NM_000081	c.1425T>G:p.S475R	missense	-	-	-	0.666:T	0	0	1	0	-
LYST	1:235972707	C	A	NM_000081	c.1411G>T:p.A471S	missense	-	-	0.0001	0.089:T	0	0	1	0	-
LYST	1:235972731	G	T	NM_000081	c.1387C>A:p.P463T	missense	-	-	-	0.096:T	1	0	0	0	-
LYST	1:235972838	C	T	NM_000081	c.1280G>A:p.S427N	missense	-	-	-	0.332:T	0	0	0	1	-
LYST	1:235972878	A	G	NM_000081	c.1240T>C:p.C414R	missense	0.0005	0.0012	0.0006	0.351:T	3	8	1	1	-
LYST	1:235972935	G	A	NM_000081	c.1183C>T:p.R395C	missense	0.0002	0.0006	0.0005	0.353:T	0	0	2	1	-
LYST	1:235973009	T	C	NM_000081	c.1109A>G:p.Q370R	missense	-	-	-	0.095:T	1	0	0	0	-
LYST	1:235973094	C	T	NM_000081	c.1024G>A:p.A342T	missense	-	-	-	0.110:T	0	0	2	0	US
LYST	1:235973193	G	C	NM_000081	c.925C>G:p.R309G	missense	-	-	-	0.139:T	0	0	0	1	-
LYST	1:235973208	C	T	NM_000081	c.910G>A:p.D304N	missense	0	-	0.0001	0.061:T	1	0	0	0	-
LYST	1:235973288	T	A	NM_000081	c.830A>T:p.H277L	missense	0.0001	-	-	0.567:T	1	1	1	0	-
LYST	1:235973294	A	C	NM_000081	c.824T>G:p.L275W	missense	0.0009	0.0031	0.0011	0.709:D	5	8	7	4	-
LYST	1:235973423	C	G	NM_000081	c.695G>C:p.G232A	missense	0.0008	0.0025	0.0006	0.084:T	1	1	1	1	-
LYST	1:235973573	T	C	NM_000081	c.545A>G:p.H182R	missense	0	-	0	0.105:T	0	0	1	0	-
LYST	1:235973624	G	A	NM_000081	c.494C>T:p.T165I	missense	-	-	-	0.548:T	1	0	0	0	-
LYST	1:235973666	T	C	NM_000081	c.452A>G:p.H151R	missense	0.0017	0.0025	0.0009	0.351:T	14	2	1	3	US
LYST	1:235973750	T	C	NM_000081	c.368A>G:p.H123R	missense	0.0111	0.0105	0.0095	0.056:T	58	45	37	24	B/LB
LYST	1:235976273	G	A	NM_000081	c.281C>T:p.T94I	missense	0.0032	0.0056	0.0035	0.178:T	2	7	4	3	CIP
LYST	1:235976319	G	C	NM_000081	c.235C>G:p.L79V	missense	-	-	-	0.736:D	1	0	0	0	US
LYST	1:235993537	T	C	NM_000081	c.181A>G:p.I61V	missense	0	-	0	0.109:T	1	0	0	0	-

LYST	1:235993597	T	C	NM_000081	c.121A>G:p.T41A	missense	-	-	-	0.076:T	0	0	0	1	-
LYST	1:235993599	G	A	NM_000081	c.119C>T:p.A40V	missense	0	-	0	0.536:T	0	1	1	1	-
LYST	1:235993603	T	C	NM_000081	c.115A>G:p.M39V	missense	-	-	-	0.631:T	0	1	0	0	-
LYST	1:235993609	T	C	NM_000081	c.109A>G:p.T37A	missense	0	0	0	0.419:T	1	0	0	0	-
LYST	1:235993618	C	T	NM_000081	c.100G>A:p.E34K	missense	-	-	-	0.537:T	0	1	0	0	-
LYST	1:235993647	A	G	NM_000081	c.71T>C:p.V24A	missense	0	0	0	0.429:T	0	0	1	0	US
LYST	1:235993656	T	C	NM_000081	c.62A>G:p.N21S	missense	0	-	-	0.155:T	0	1	0	0	-
LYST	1:235993666	G	A	NM_000081	c.52C>T:p.R18W	missense	5.8E-05	-	0	0.637:T	0	0	1	0	US
ST3GAL5	2:86067278	G	A	NM_003896	c.1246C>T:p.R416C	missense	0.0003	0.0006	0.0003	0.233:T	1	0	1	0	US
ST3GAL5	2:86067346	G	A	NM_003896	c.1178C>T:p.T393M	missense	0.0006	-	0.0006	0.286:T	2	2	1	1	US
ST3GAL5	2:86067383	C	T	NM_003896	c.1141G>A:p.A381T	missense	-	-	-	0.073:T	1	0	1	0	-
ST3GAL5	2:86067433	T	G	NM_003896	c.1091A>C:p.D364A	missense	-	-	-	0.399:T	0	0	1	0	-
ST3GAL5	2:86067464	C	T	NM_003896	c.1060G>A:p.D354N	missense	0	-	0	0.592:T	0	0	0	1	-
ST3GAL5	2:86067480	T	A	NM_003896	c.1044A>T:p.L348F	missense	5.80E-05	-	-	0.798:D	1	0	1	0	-
ST3GAL5	2:86067488	C	T	NM_003896	c.1036G>A:p.V346I	missense	0.0034	0.0018	0.0036	0.246:T	16	7	16	5	CIP
ST3GAL5	2:86071526	C	T	NM_003896	c.1001G>A:p.R334Q	missense	0	-	0	0.061:T	1	0	0	0	-
ST3GAL5	2:86071527	G	A	NM_003896	c.1000C>T:p.R334X	stopgain	0	0	0	0.580:T	0	1	0	0	P
ST3GAL5	2:86071527	G	-	NM_003896	c.1000delC:p.R334Efs*14	frameshift deletion	0.0001	-	-	-	0	0	2	0	-
ST3GAL5	2:86071570	A	C	NM_003896	c.957T>G:p.F319L	missense	0.0006	-	0.0006	0.156:T	4	3	2	1	US
ST3GAL5	2:86073550	TAAA	-	NM_003896	c.796_799del:p.F266Rfs*11	frameshift deletion	-	-	-	-	1	0	0	0	-

ST3GAL5	2:86075102	C	T	NM_003896	c.544G>A:p.D182N	missense	0	0	0	0.044:T	1	0	0	0	US
ST3GAL5	2:86075152	C	T	NM_003896	c.494G>A:p.R165Q	missense	0	-	0	0.200:T	0	2	0	2	US
ST3GAL5	2:86075153	G	A	NM_003896	c.493C>T:p.R165W	missense	0	-	0	0.645:T	1	0	0	0	-
ST3GAL5	2:86075191	C	T	NM_003896	c.455G>A:p.S152N	missense	-	-	-	0.133:T	0	1	0	0	-
ST3GAL5	2:86075224	A	G	NM_003896	c.422T>C:p.L141S	missense	0.0008	-	0.0007	0.503:T	2	3	6	1	US
ST3GAL5	2:86075257	G	A	NM_003896	c.389C>T:p.A130V	missense	5.8E-05	0	0.0001	0.075:T	0	0	0	1	CIP
ST3GAL5	2:86075259	C	T	NM_003896	c.387G>A:p.M129I	missense	-	-	-	0.567:T	0	2	0	0	-
ST3GAL5	2:86075505	C	T	NM_001354233	UTR5	splicing	-	-	-	-	0	1	0	0	-
ST3GAL5	2:86090558	G	T	NM_003896	c.133C>A:p.P45T	missense	-	-	-	0.169:T	1	0	0	0	-
ST3GAL5	2:86090566	C	T	NM_003896	c.125G>A:p.C42Y	missense	-	-	-	0.415:T	0	0	0	1	-
ST3GAL5	2:86094727	C	T	NM_001042437	c.13+1G>A	splicing	0.0014	0.0006	0.0012	0.128:T	6	3	5	2	-
ST3GAL5	2:86115992	G	A	NM_003896	c.37C>T:p.P13S	missense	0	0	0	0.065:T	2	0	0	0	B
ST3GAL5	2:86116015	G	T	NM_003896	c.14C>A:p.A5E	missense	0.0013	-	-	0.388:T	0	0	1	1	-
SUMF1	3:4403835	A	G	NM_001164674	c.1043T>C:p.M348T	missense	-	-	-	0.234:T	0	1	0	0	-
SUMF1	3:4403836	T	C	NM_001164674	c.1042A>G:p.M348V	missense	-	-	-	0.293:T	0	0	1	0	-
SUMF1	3:4403848	G	A	NM_001164674	c.1030C>T:p.R344C	missense	0	0	0	0.469:T	0	0	1	0	-
SUMF1	3:4403874	T	C	NM_001164674	c.1004A>G:p.N335S	missense	0	-	0	0.565:T	1	0	0	0	-
SUMF1	3:4452611	C	T	NM_001164674	c.817G>A:p.A273T	missense	5.80E-05	-	-	0.787:D	1	0	0	0	-
SUMF1	3:4452616	C	G	NM_001164674	c.812G>C:p.G271A	missense	-	-	-	0.966:D	0	1	0	0	-

SUMF1	3:4452620	C	G	NM_001164674	c.808G>C:p.V270L	missense	-	-	-	0.909:D	0	0	1	0	-
SUMF1	3:4458843	G	A	NM_001164674	c.734C>T:p.T245I	missense	5.8E-05	0.0006	-	0.878:D	0	0	0	1	-
SUMF1	3:4459712	C	T	NM_001164674	c.632G>A:p.R211Q	missense	0.0002	-	0.0001	0.950:D	4	1	4	1	-
SUMF1	3:4459778	G	A	NM_001164674	c.566C>T:p.A189V	missense	0	-	0	0.948:D	1	1	0	1	-
SUMF1	3:4459809	G	A	NM_001164674	c.535C>T:p.H179Y	missense	5.80E-05	-	-	0.930:D	0	1	0	0	-
SUMF1	3:4461794	C	T	NM_001164674	c.481G>A:p.A161T	missense	0	0	0	0.915:D	0	0	1	0	-
SUMF1	3:4494592	T	C	NM_001164674	c.412A>G:p.K138E	missense	-	-	-	0.620:T	0	0	0	1	-
SUMF1	3:4494643	T	C	NM_001164674	c.361A>G:p.I121V	missense	5.80E-05	-	0.0001	0.269:T	1	0	0	0	-
SUMF1	3:4494660	G	C	NM_001164674	c.344C>G:p.P115R	missense	-	-	-	0.997:D	0	0	1	0	-
SUMF1	3:4494711	A	G	NM_001164674	c.293T>C:p.V98A	missense	-	-	-	0.367:T	1	0	0	0	-
SUMF1	3:4494721	G	C	NM_001164674	c.283C>G:p.P95A	missense	-	-	-	0.782:D	0	1	0	0	-
SUMF1	3:4508691	A	T	NM_001164674	c.239T>A:p.V80E	missense	0.0005	-	0.0008	0.288:T	1	1	0	2	US
SUMF1	3:4508742	C	T	NM_001164674	c.188G>A:p.S63N	missense	0.0017	0	0.0046	0.283:T	2	12	4	2	B
SUMF1	3:4508784	G	A	NM_001164674	c.146C>T:p.S49F	missense	8.79E-05	-	0	0.796:D	1	3	0	3	-
SUMF1	3:4508791	C	T	NM_001164674	c.139G>A:p.A47T	missense	0	-	0	0.246:T	0	1	0	0	-
SUMF1	3:4508842	C	T	NM_001164674	c.88G>A:p.G30R	missense	-	-	-	0.225:T	1	0	0	0	-
SUMF1	3:4508892	C	A	NM_001164674	c.38G>T:p.C13F	missense	0	0	0	0.447:T	0	2	0	0	-
GLB1	3:33038569	T	A	NM_000404	c.2002A>T:p.K668X	stopgain	0.0002	0.0012	0.0001	0.331:T	1	3	9	3	US
GLB1	3:33038574	G	A	NM_000404	c.1997C>T:p.P666L	missense	0	0.0006	0	0.297:T	2	0	2	0	-
GLB1	3:33038574	G	C	NM_000404	c.1997C>G:p.P666R	missense	0.0001	-	-	0.329:T	1	1	0	0	-
GLB1	3:33038619	T	C	NM_000404	c.1952A>G:p.D651G	missense	0	-	-	0.224:T	0	0	1	0	-
GLB1	3:33038661	G	A	NM_000404	c.1910C>T:p.T637M	missense	0	0.0006	0	0.337:T	1	0	0	0	-
GLB1	3:33038739	G	A	NM_000404	c.1832C>T:p.S611L	missense	-	-	-	0.583:T	1	0	0	0	-
GLB1	3:33038830	C	T	NM_000404	c.1741G>A:p.V581I	missense	-	-	-	0.268:T	0	1	0	0	-
GLB1	3:33055548	C	A	NM_000404	c.1734G>T:p.K578N	missense	-	-	-	0.663:T	0	1	0	0	p.K578R
GLB1	3:33055567	T	C	NM_000404	c.1715A>G:p.Q572R	missense	0.0066	0.0062	0.0067	0.294:T	16	15	21	5	LB
GLB1	3:33055625	T	C	NM_000404	c.1657A>G:p.M553V	missense	-	-	-	0.232:T	0	0	1	0	-
GLB1	3:33055643	T	G	NM_000404	c.1639A>C:p.T547P	missense	0.0002	-	0.0001	0.474:T	2	2	0	0	-
GLB1	3:33055720	C	T	NM_000404	c.1562G>A:p.C521Y	missense	5.80E-05	0	0	0.140:T	1	0	0	0	-
GLB1	3:33055730	C	G	NM_000404	c.1552G>C:p.D518H	missense	-	-	-	0.278:T	5	0	1	0	-
GLB1	3:33055738	T	C	NM_000404	c.1544A>G:p.D515G	missense	5.80E-05	-	0.0001	0.281:T	0	1	0	0	-
GLB1	3:33055742	G	C	NM_000404	c.1540C>G:p.L514V	missense	-	-	-	0.754:D	0	1	0	0	-

GLB1	3:33058242	T	C	NM_000404	c.1438A>G:p.M480V	missense	0	-	-	0.808:D	0	1	0	0	US
GLB1	3:33058310	C	T	NM_000404	c.1370G>A:p.R457Q	missense	0	-	-	0.841:D	1	0	0	1	P
GLB1	3:33059944	T	A	NM_000404	c.1343A>T:p.D448V	missense	0.0002	-	0.0002	0.665:T	1	0	0	0	CIP
GLB1	3:33059977	T	C	NM_000404	c.1310A>G:p.N437S	missense	0.0005	0.0006	0.0006	0.197:T	1	0	0	1	CIP
GLB1	3:33063060	G	A	NM_000404	c.1231C>T:p.Q411X	stopgain	-	-	-	0.845:D	0	0	1	0	-
GLB1	3:33063063	T	G	NM_000404	c.1228A>C:p.K410Q	missense	0.0009	0.0006	0.0006	0.566:T	7	5	8	3	-
GLB1	3:33063095	T	C	NM_000404	c.1196A>G:p.K399R	missense	5.80E-05	-	-	0.408:T	1	2	0	1	-
GLB1	3:33063113	C	T	NM_000404	c.1178G>A:p.C393Y	missense	-	-	-	0.518:T	0	0	0	1	-
GLB1	3:33063122	T	C	NM_000404	c.1169A>G:p.D390G	missense	0.0006	-	0.0007	0.257:T	1	0	0	0	-
GLB1	3:33093405	T	C	NM_000404	c.884A>G:p.D295G	missense	0.0005	0	0.0003	0.437:T	0	1	5	0	-
GLB1	3:33093439	T	C	NM_000404	c.850A>G:p.I284V	missense	0	0.0006	0	0.315:T	0	1	0	0	-
GLB1	3:33099601	G	A	NM_000404	c.713C>T:p.T238I	missense	-	-	-	0.656:T	1	0	0	0	-
GLB1	3:33099740	A	G	NM_000404	c.574T>C:p.Y192H	missense	5.80E-05	-	-	0.951:D	0	1	0	0	-
GLB1	3:33099750	T	G	NM_000404	c.564A>C:p.E188D	missense	-	-	-	0.926:D	1	0	0	0	-
GLB1	3:33107011	G	A	NM_001135602	c.284C>T:p.S95F	missense	0.0003	-	0.0003	0.761:D	0	1	1	1	-
GLB1	3:33109733	G	A	NM_000404	c.446C>T:p.S149F	missense	5.80E-05	-	0.0001	0.986:D	1	0	0	0	CIP
GLB1	3:33109733	G	T	NM_000404	c.446C>A:p.S149Y	missense	-	-	-	0.950:D	0	0	1	0	-
GLB1	3:33109755	T	A	NM_000404	c.424A>T:p.K142X	stopgain	5.80E-05	-	0.0001	0.802:D	1	0	0	0	-
GLB1	3:33109781	C	G	NM_000404	c.398G>C:p.G133A	missense	-	-	-	0.957:D	0	1	0	0	-
GLB1	3:33110377	C	T	NM_000404	c.331G>A:p.A111T	missense	-	-	-	0.539:T	0	0	0	1	-
GLB1	3:33110383	G	A	NM_000404	c.325C>T:p.R109W	missense	5.80E-05	0	0	0.462:T	1	0	0	0	B/LB
GLB1	3:33110410	C	T	NM_000404	c.298G>A:p.E100K	missense	-	-	-	0.590:T	1	0	0	0	-
GLB1	3:33110438	C	A	NM_000404	c.270G>T:p.E90D	missense	-	-	-	0.781:D	0	1	0	0	-
GLB1	3:33110460	T	C	NM_000404	c.248A>G:p.Y83C	missense	-	-	-	0.995:D	0	0	0	1	US
GLB1	3:33114060	A	G	NM_000404	c.221T>C:p.M74T	missense	-	-	-	0.531:T	0	0	1	0	-
GLB1	3:33114106	G	A	NM_000404	c.175C>T:p.R59C	missense	0	-	0	0.823:D	1	0	0	0	P/LP
GLB1	3:33114162	G	A	NM_000404	c.119C>T:p.S40F	missense	0	-	-	0.467:T	3	1	2	1	-
GLB1	3:33114168	C	T	NM_000404	c.113G>A:p.R38Q	missense	0	0	0	0.394:T	0	2	0	1	-
GLB1	3:33114193	T	A	NM_000404	c.88A>T:p.R30W	missense	-	-	-	0.665:T	1	0	0	0	-
GLB1	3:33118596	C	T	NM_001317040	c.209G>A:p.R70Q	missense	0.0007	0.0025	0.0076	0.238:T	10	4	5	3	-
GLB1	3:33118597	G	A	NM_001317040	c.208C>T:p.R70W	missense	0.0002	-	-	0.277:T	2	0	1	0	-
GLB1	3:33118632	T	C	NM_001317040	c.173A>G:p.H58R	missense	9.9E-05	-	-	0.307:T	0	0	1	0	-

GLB1	3:33118696	G	C	NM_001317040	c.109C>G:p.R37G	missense	7.88E-05	0.0006	-	0.289:T	1	0	2	1	-
GLB1	3:33118726	G	A	NM_001317040	c.79C>T:p.R27X	stopgain	6.37E-05	0.0006	0	0.365:T	2	1	1	1	-
HYAL1	3:50337931	G	A	NM_153281	c.1291C>T:p.R431W	missense	5.80E-05	-	0.0002	0.248:T	1	0	0	0	US
HYAL1	3:50338021	G	A	NM_153281	c.1201C>T:p.R401W	missense	0.0005	0.0019	0.001	0.133:T	7	6	6	0	-
HYAL1	3:50338080	A	T	NM_153281	c.1142T>A:p.L381H	missense	-	0	-	0.283:T	1	0	2	0	-
HYAL1	3:50338108	G	A	NM_153281	c.1114C>T:p.R372C	missense	0	0	0	0.436:T	1	0	0	0	-
HYAL1	3:50338129	C	T	NM_153281	c.1093G>A:p.G365S	missense	0	0	0	0.253:T	0	1	0	0	-
HYAL1	3:50338130	G	-	NM_153281	c.1092delC:p.G365Afs*33	frameshift deletion	-	-	-	-	0	0	1	0	-
HYAL1	3:50338228	A	T	NM_153281	c.994T>A:p.S332T	missense	-	-	-	0.232:T	1	0	0	0	-
HYAL1	3:50338429	G	A	NM_153281	c.980C>T:p.T327I	missense	-	-	-	0.458:T	1	0	0	1	-
HYAL1	3:50338486	C	A	NM_153281	c.923G>T:p.G308V	missense	-	-	-	0.873:D	1	0	0	1	-
HYAL1	3:50339489	A	-	NM_153281	c.899delT:p.L300Rfs*22;-	frameshift deletion	-	-	-	-	0	1	0	1	-
HYAL1	3:50339516	T	C	NM_153281	c.872A>G:p.Y291C	missense	0.0003	-	0.0003	0.901:D	3	1	5	0	-
HYAL1	3:50339541	C	G	NM_153281	c.847G>C:p.V283L	missense	0.0003	-	0.0003	0.591:T	1	1	0	0	-
HYAL1	3:50339552	G	C	NM_153281	c.836C>G:p.P279R	missense	5.80E-05	-	-	0.325:T	1	0	0	0	-
HYAL1	3:50339622	C	T	NM_153281	c.766G>A:p.G256R	missense	0.0009	0.0018	0.0013	0.074:T	1	3	0	2	B
HYAL1	3:50339643	G	A	NM_153281	c.745C>T:p.P249S	missense	0.0175	0.0185	0.0169	0.114:T	36	33	42	22	B
HYAL1	3:50339690	C	A	NM_153281	c.698G>T:p.G233V	missense	-	-	-	0.413:T	0	0	0	1	-
HYAL1	3:50339711	C	T	NM_153281	c.677G>A:p.R226H	missense	5.80E-05	0	0	0.013:T	1	0	0	0	-
HYAL1	3:50339712	G	A	NM_153281	c.676C>T:p.R226C	missense	0	-	0	0.036:T	0	0	1	0	-
HYAL1	3:50339751	G	C	NM_153281	c.637C>G:p.L213V	missense	0	-	0	0.041:T	1	0	0	0	-
HYAL1	3:50339801	C	T	NM_153281	c.587G>A:p.R196H	missense	0.0001	0	0.0002	0.044:T	0	0	2	0	US
HYAL1	3:50339807	C	T	NM_153281	c.581G>A:p.R194H	missense	0	-	0.0001	0.841:D	1	1	2	4	-
HYAL1	3:50339808	G	A	NM_153281	c.580C>T:p.R194C	missense	-	-	0	0.876:D	0	0	1	0	-
HYAL1	3:50339849	C	T	NM_153281	c.539G>A:p.R180Q	missense	0	-	0	0.008:T	2	0	0	0	-
HYAL1	3:50339889	C	T	NM_153281	c.499G>A:p.V167M	missense	-	-	-	0.674:T	1	0	0	0	-
HYAL1	3:50339897	G	A	NM_153281	c.491C>T:p.A164V	missense	0	-	0	0.076:T	0	1	0	0	-
HYAL1	3:50339907	C	T	NM_153281	c.481G>A:p.D161N	missense	-	-	0.0001	0.028:T	1	0	0	0	-
HYAL1	3:50339976	C	T	NM_153281	c.412G>A:p.A138T	missense	-	-	-	0.400:T	0	1	0	0	-
HYAL1	3:50340026	T	A	NM_153281	c.362A>T:p.D121V	missense	0.0003	-	0.0003	0.472:T	1	0	0	0	-

HYAL1	3:50340051	T	G	NM_153281	c.337A>C:p.I113L	missense	-	-	-	0.256:T	0	0	1	0	-
HYAL1	3:50340065	C	T	NM_153281	c.323G>A:p.R108H	missense	0	0	0	0.033:T	1	0	0	0	B
HYAL1	3:50340066	G	A	NM_153281	c.322C>T:p.R108C	missense	0	0	0	0.286:T	1	0	0	0	-
HYAL1	3:50340167	A	-	NM_153281	c.221delT:p.F74Sfs*28	frameshift deletion	5.80E-05	-	-	-	1	2	3	0	-
HYAL1	3:50340300	G	A	NM_153281	c.88C>T:p.R30W	missense	5.80E-05	-	-	0.387:T	1	0	0	0	-
HYAL1	3:50340342	G	A	NM_153281	c.46C>T:p.L16F	missense	0.0001	0.0006	-	0.145:T	0	1	0	1	-
HPS3	3:148847534	C	A	NM_032383	c.24C>A:p.H8Q	missense	-	-	-	0.843:D	0	1	0	0	-
HPS3	3:148847592	G	T	NM_032383	c.82G>T:p.G28W	missense	0.0001	-	0.0001	0.738:D	1	0	0	0	-
HPS3	3:148847657	G	T	NM_032383	c.147G>T:p.Q49H	missense	0	-	-	0.190:T	0	0	1	0	-
HPS3	3:148847668	A	G	NM_032383	c.158A>G:p.Q53R	missense	0.0149	0.0136	0.0251	0.064:T	53	71	52	44	B
HPS3	3:148847669	G	C	NM_032383	c.159G>C:p.Q53H	missense	-	-	-	0.189:T	0	0	0	1	US
HPS3	3:148847673	C	T	NM_032383	c.163C>T:p.R55W	missense	0.0003	-	0.0003	0.733:D	1	1	3	0	-
HPS3	3:148847700	G	T	NM_032383	c.190G>T:p.V64L	missense	0.0003	-	0	0.799:D	0	0	1	0	-
HPS3	3:148847713	C	A	NM_032383	c.203C>A:p.A68D	missense	9.28E-05	-	-	0.657:T	1	0	0	0	-
HPS3	3:148857905	A	G	NM_032383	c.332A>G:p.H111R	missense	-	-	-	0.798:D	0	0	1	0	-
HPS3	3:148857971	C	T	NM_032383	c.398C>T:p.S133L	missense	0.0014	0.0006	0.0012	0.457:T	1	4	2	2	US
HPS3	3:148858022	T	A	NM_032383	c.449T>A:p.V150D	missense	-	-	-	0.985:D	2	0	1	0	-
HPS3	3:148858035	-	AA	NM_032383	c.462_463insAA:p.L156Nfs*1	frameshift insertion	-	-	-	-	0	1	0	0	-
HPS3	3:148858040	T	C	NM_032383	c.467T>C:p.L156S	missense	5.80E-05	-	0.0001	0.926:D	0	1	0	0	-
HPS3	3:148858250	A	G	NM_032383	c.677A>G:p.H226R	missense	0.0007	0.0012	0.0008	0.031:T	4	1	0	0	US
HPS3	3:148858287	T	A	NM_032383	c.712+2T>A	splicing	-	-	-	0.589:T	0	1	0	0	-
HPS3	3:148858831	A	G	NM_032383	c.740A>G:p.E247G	missense	-	-	-	0.579:T	0	0	1	0	-
HPS3	3:148858872	C	-	NM_032383	c.781delC:p.G262Vfs*17	frameshift deletion	-	-	-	-	1	0	0	0	-
HPS3	3:148858872	C	T	NM_032383	c.781C>T:p.L261F	missense	5.80E-05	-	0.0001	0.108:T	0	1	0	0	-
HPS3	3:148858872	C	-	NM_032383	c.781delC:p.G262Vfs*17	frameshift deletion	-	-	-	-	1	0	0	0	-
HPS3	3:148858875	G	C	NM_032383	c.784G>C:p.G262R	missense	-	-	-	0.719:D	1	0	0	0	-
HPS3	3:148858972	A	G	NM_032383	c.881A>G:p.Y294C	missense	0	0.0006	-	0.887:D	1	0	2	0	-

HPS3	3:148859083	C	T	NM_032383	c.886C>T:p.R296C;-	missense	0	0	0	0.633:T	1	0	0	0	-
HPS3	3:148859102	C	T	NM_032383	c.905C>T:p.S302L	missense	0.001	0.0019	0.0015	0.620:T	3	3	0	0	US
HPS3	3:148859140	C	T	NM_032383	c.943C>T:p.L315F	missense	5.80E-05	0	0.0001	0.760:D	1	0	0	0	-
HPS3	3:148863140	G	T	NM_032383	c.971-1G>T	splicing	-	-	-	0.535:T	0	0	1	0	-
HPS3	3:148863166	T	A	NM_032383	c.996T>A:p.N332K	missense	-	-	-	0.028:T	1	0	0	0	-
HPS3	3:148863306	C	T	NM_032383	c.1136C>T:p.T379M	missense	0	0	0	0.770:D	1	0	0	0	US
HPS3	3:148863316	T	A	NM_032383	c.1146T>A:p.F382L	missense	-	-	-	0.608:T	2	2	0	0	-
HPS3	3:148868412	G	A	NM_032383	c.1190G>A:p.R397Q	missense	0	-	0	0.958:D	0	0	1	0	-
HPS3	3:148868421	C	T	NM_032383	c.1199C>T:p.A400V	missense	5.8E-05	-	0.0001	0.669:T	0	0	1	0	-
HPS3	3:148868450	A	G	NM_032383	c.1228A>G:p.M410V	missense	0.0043	0.0068	0.0043	0.131:T	4	1	1	0	LB
HPS3	3:148871305	G	A	NM_032383	c.1270G>A:p.V424I	missense	-	-	-	0.210:T	3	4	1	1	-
HPS3	3:148871362	A	G	NM_032383	c.1327A>G:p.N443D	missense	-	-	-	0.372:T	0	0	0	1	-
HPS3	3:148871422	C	T	NM_032383	c.1387C>T:p.P463S	missense	0.0007	0	0.001	0.037:T	1	2	0	0	-
HPS3	3:148872896	C	T	NM_032383	c.1403C>T:p.S468L	missense	0	0	0	0.575:T	0	0	0	1	CIP
HPS3	3:148872931	C	A	NM_032383	c.1438C>A:p.P480T	missense	-	-	-	0.215:T	0	1	0	1	-
HPS3	3:148872958	T	C	NM_032383	c.1465T>C:p.Y489H	missense	-	-	-	0.965:D	1	0	0	0	-
HPS3	3:148875144	A	G	NM_032383	c.1517A>G:p.Y506C	missense	0.0004	-	0.0006	0.959:D	1	1	1	0	-
HPS3	3:148875241	G	C	NM_032383	c.1614G>C:p.Q538H	missense	-	-	-	0.329:T	0	1	0	1	-
HPS3	3:148875254	G	A	NM_032383	c.1627G>A:p.E543K	missense	5.80E-05	-	0.0001	0.511:T	1	0	0	0	-
HPS3	3:148875256	G	C	NM_032383	c.1629G>C:p.E543D	missense	0	-	0	0.218:T	1	0	0	0	-
HPS3	3:148875308	T	C	NM_032383	c.1681T>C:p.C561R	missense	-	-	-	0.939:D	1	0	0	0	-
HPS3	3:148876472	C	T	NM_032383	c.1711C>T:p.H571Y	missense	0.0053	0.0031	0.0069	0.733:D	29	24	21	15	LB
HPS3	3:148876527	C	T	NM_032383	c.1766C>T:p.A589V	missense	-	-	-	0.164:T	0	0	1	0	-
HPS3	3:148876530	G	A	NM_032383	c.1769G>A:p.R590H	missense	0	0	0	0.806:D	1	0	0	0	LB
HPS3	3:148876532	A	T	NM_032383	c.1771A>T:p.T591S	missense	-	-	-	0.099:T	0	0	1	0	-
HPS3	3:148876533	C	T	NM_032383	c.1772C>T:p.T591M	missense	0.001	-	0.0012	0.060:T	6	5	5	6	-
HPS3	3:148876599	C	G	NM_032383	c.1838C>G:p.S613X	stopgain	0.0006	0.0012	0.0008	0.701:D	5	2	1	3	P
HPS3	3:148876607	G	A	NM_032383	c.1846G>A:p.E616K	missense	-	-	-	0.858:D	0	1	0	0	-
HPS3	3:148877955	G	C	NM_032383	c.1995G>C:p.K665N	missense	-	-	-	0.075:T	1	0	0	0	-
HPS3	3:148878028	C	T	NM_032383	c.2068C>T:p.L690F	missense	-	-	-	0.489:T	0	0	1	0	-
HPS3	3:148879965	C	A	NM_032383	c.2137C>A:p.P713T	missense	-	-	-	0.924:D	0	1	1	0	-
HPS3	3:148879965	C	T	NM_032383	c.2137C>T:p.P713S	missense	-	-	-	0.880:D	0	1	0	0	-

HPS3	3:148880010	G	A	NM_032383	c.2182G>A:p.E728K	missense	0.001	0	0.0013	0.186:T	1	0	0	0	-
HPS3	3:148880017	C	G	NM_032383	c.2189C>G:p.A730G	missense	-	-	-	0.649:T	1	0	1	0	-
HPS3	3:148880038	A	G	NM_032383	c.2210A>G:p.Q737R	missense	0.0001	0.0012	0.0001	0.038:T	3	1	1	2	-
HPS3	3:148880092	G	T	NM_032383	c.2264G>T:p.G755V	missense	5.80E-05	-	-	0.484:T	2	1	0	1	-
HPS3	3:148880477	G	T	NM_032383	c.2293G>T:p.V765L;-	missense	-	-	-	0.153:T	0	1	0	2	-
HPS3	3:148880567	C	T	NM_032383	c.2383C>T:p.L795F	missense	-	-	-	0.381:T	0	0	1	0	-
HPS3	3:148880631	C	T	NM_032383	c.2447C>T:p.P816L	missense	0	-	-	0.571:T	1	0	0	0	-
HPS3	3:148880655	C	T	NM_032383	c.2471C>T:p.S824L	missense	0.0011	0.0012	0.0013	0.354:T	2	3	1	2	p.S824X
HPS3	3:148881671	C	T	NM_032383	c.2524C>T:p.H842Y	missense	0.0013	0.0012	0.0014	0.059:T	9	6	8	2	US
HPS3	3:148881677	G	A	NM_032383	c.2530G>A:p.V844I	missense	0	0.0006	0	0.031:T	0	1	0	0	-
HPS3	3:148881708	A	G	NM_032383	c.2561A>G:p.N854S	missense	-	-	-	0.130:T	0	1	0	0	-
HPS3	3:148884824	C	A	NM_032383	c.2593C>A:p.L865I	missense	-	-	-	0.749:D	0	0	0	1	-
HPS3	3:148884864	C	T	NM_032383	c.2633C>T:p.P878L	missense	0	0	0	0.874:D	0	0	0	1	-
HPS3	3:148884899	G	C	NM_032383	c.2668G>C:p.G890R	missense	-	-	-	0.937:D	0	0	1	0	-
HPS3	3:148884920	T	C	NM_032383	c.2689T>C:p.C897R	missense	-	-	-	0.983:D	1	0	0	0	-
HPS3	3:148884929	C	T	NM_032383	c.2698C>T:p.R900C	missense	5.80E-05	-	0	0.922:D	1	1	0	0	-
HPS3	3:148884930	G	A	NM_032383	c.2699G>A:p.R900H	missense	0	0	0	0.887:D	0	2	0	1	US
HPS3	3:148885025	C	T	NM_032383	c.2794C>T:p.R932W	missense	0	0	0	0.187:T	1	0	0	0	-
HPS3	3:148889948	G	T	NM_032383	c.2954G>T:p.G985V	missense	5.81E-05	0.0006	-	0.968:D	0	1	1	0	-
HPS3	3:148889989	C	T	NM_032383	c.2995C>T:p.R999X	stopgain	5.81E-05	-	-	0.610:T	1	2	0	0	-
HPS3	3:148889990	G	A	NM_032383	c.2996G>A:p.R999Q	missense	0.0002	0.0006	0.0001	0.069:T	1	0	0	1	-
HPS3	3:148889990	G	T	NM_032383	c.2996G>T:p.R999L	missense	0.0005	-	0.0005	0.603:T	1	1	2	1	-
IDUA	4:980874	T	C	NM_000203	c.2T>C:p.M1T	missense	0	-	0	0.835:D	0	0	0	3	P
IDUA	4:980877	G	T	NM_000203	c.5G>T:p.R2L	missense	-	-	-	0.393:T	0	0	2	0	-
IDUA	4:980937	C	T	NM_000203	c.65C>T:p.P22L	missense	0.0012	0.0012	-	0.396:T	2	1	2	0	US
IDUA	4:980948	G	A	NM_000203	c.76G>A:p.A26T	missense	0.0034	0.0031	0.0114	0.074:T	22	11	21	15	CIP
IDUA	4:980991	T	C	NM_000203	c.119T>C:p.L40P	missense	0	-	-	0.681:T	0	0	1	0	-
IDUA	4:981003	G	A	NM_000203	c.131G>A:p.R44Q	missense	-	-	-	0.642:T	0	0	0	1	-
IDUA	4:981602	C	A	NM_000203	c.164C>A:p.P55Q	missense	5.84E-05	-	-	0.788:D	1	0	0	0	-
IDUA	4:981661	G	A	NM_000203	c.223G>A:p.A75T	missense	0	0	0	0.737:D	1	0	0	0	P
IDUA	4:981686	G	A	NM_000203	c.248G>A:p.R83H	missense	0.0002	-	0.0002	0.201:T	0	0	2	0	US
IDUA	4:981703	C	T	NM_000203	c.265C>T:p.R89W	missense	0	-	0	0.981:D	3	0	1	1	P

IDUA	4:994455	G	T	NM_000203	c.355G>T:p.D119Y	missense	0.0002	0.0006	0.0002	0.842:D	0	0	1	1	-
IDUA	4:994457	C	A	NM_000203	c.357C>A:p.D119E	missense	-	-	-	0.499:T	0	0	1	0	-
IDUA	4:994694	C	T	NM_000203	c.410C>T:p.S137L	missense	0	-	0	0.766:D	0	0	1	0	-
IDUA	4:995261	T	C	NM_000203	c.499T>C:p.Y167H	missense	-	-	-	0.850:D	1	1	0	0	-
IDUA	4:995263	C	A	NM_000203	c.501C>A:p.Y167X	stopgain	-	-	-	0.609:T	1	0	0	0	P
IDUA	4:995271	C	T	NM_000203	c.509C>T:p.A170V	missense	0	-	0	0.207:T	0	0	1	0	-
IDUA	4:995318	C	T	NM_000203	c.556C>T:p.H186Y	missense	0.0001	-	-	0.602:T	2	2	0	0	-
IDUA	4:995333	G	C	NM_000203	c.571G>C:p.V191L	missense	0.0007	0.0018	0.0002	0.485:T	0	0	1	0	-
IDUA	4:995343	C	G	NM_000203	c.581C>G:p.T194S	missense	0.0007	0.0006	0.0012	0.542:T	2	1	2	3	US
IDUA	4:995641	G	C	NM_000203	c.764G>C:p.R255P	missense	6.69E-05	-	-	0.889:D	1	0	0	0	-
IDUA	4:995888	T	-	NM_000203	c.911delT:p.V304Gfs*12	frameshift deletion	-	-	-	-	0	1	0	0	-
IDUA	4:996114	G	A	NM_000203	c.1030G>A:p.A344T	missense	0.0002	0	0.0005	0.201:T	1	0	1	0	-
IDUA	4:996115	C	G	NM_000203	c.1031C>G:p.A344G	missense	0.0042	-	0.0042	0.400:T	10	7	9	2	CIP
IDUA	4:996121	T	G	NM_000203	c.1037T>G:p.L346R	missense	5.81E-05	-	0.0001	0.997:D	1	0	0	0	P
IDUA	4:996130	-	C	NM_000203	c.1047dupC:p.N350Qfs*48	frameshift insertion	-	-	-	-	0	1	0	1	-
IDUA	4:996163	T	G	NM_000203	c.1079T>G:p.F360C	missense	-	-	-	0.939:D	3	0	2	0	-
IDUA	4:996177	C	G	NM_000203	c.1093C>G:p.L365V	missense	0.0025	0.0025	0.0028	0.831:D	8	4	7	5	CIP
IDUA	4:996225	C	G	NM_000203	c.1141C>G:p.L381V	missense	0.0002	-	0	0.558:T	0	0	1	0	-
IDUA	4:996253	T	C	NM_000203	c.1169T>C:p.M390T	missense	0.0001	-	-	0.928:D	0	1	0	2	-
IDUA	4:996555	G	C	NM_000203	c.1225G>C:p.G409R	missense	0.0147	0.0204	0.0231	0.703:D	65	31	51	24	CIP
IDUA	4:996565	T	C	NM_000203	c.1235T>C:p.L412P	missense	-	-	-	0.827:D	2	0	0	1	-
IDUA	4:996567	G	C	NM_000203	c.1237G>C:p.D413H	missense	-	-	-	0.511:T	1	0	0	0	-
IDUA	4:996658	G	A	NM_000203	c.1328G>A:p.S443N	missense	0.0006	-	0.01	0.470:T	0	1	0	0	-
IDUA	4:996719	-	C	NM_000203	c.1390dupC:p.G466Rfs*42	frameshift insertion	-	-	-	-	2	1	0	0	-
IDUA	4:996733	G	T	NM_000203	c.1402+1G>T	splicing	-	-	-	0.409:T	1	0	0	0	-
IDUA	4:996910	A	G	NM_000203	c.1489A>G:p.T497A	missense	0	-	-	0.362:T	2	1	0	3	-
IDUA	4:996932	T	C	NM_000203	c.1511T>C:p.M504T	missense	-	-	-	0.924:D	0	0	1	0	-

IDUA	4:997133	G	A	NM_000203	c.1525G>A:p.D509N;-	missense	-	-	-	0.462:T	2	0	2	1	-
IDUA	4:997169	G	A	NM_000203	c.1561G>A:p.G521S	missense	0.0002	0.0006	-	0.251:T	0	1	1	1	-
IDUA	4:997205	C	A	NM_000203	c.1597C>A:p.P533T	missense	-	-	-	0.849:D	1	0	0	0	-
IDUA	4:997205	C	T	NM_000203	c.1597C>T:p.P533S	missense	0.0005	-	-	0.815:D	1	0	0	0	-
IDUA	4:997237	C	-	NM_000203	c.1629delC:p.E545Rfs*13	frameshift deletion	-	-	-	-	1	0	0	0	-
IDUA	4:997341	C	G	NM_000203	c.1655C>G:p.T552R	missense	-	-	-	0.401:T	1	0	0	0	-
IDUA	4:997371	G	A	NM_000203	c.1685G>A:p.G562E	missense	-	0	-	0.655:T	0	0	1	0	-
IDUA	4:997379	G	A	NM_000203	c.1693G>A:p.V565I	missense	0	-	-	0.121:T	0	0	0	1	-
IDUA	4:997401	A	G	NM_000203	c.1715A>G:p.H572R	missense	0.0005	-	0.0004	0.150:T	1	0	0	0	-
IDUA	4:997802	G	A	NM_000203	c.1730G>A:p.C577Y	missense	-	-	-	0.923:D	0	0	1	0	-
IDUA	4:997810	A	G	NM_000203	c.1738A>G:p.T580A	missense	-	-	-	0.562:T	1	2	0	0	-
IDUA	4:997816	G	A	NM_000203	c.1744G>A:p.E582K	missense	0	0	0	0.785:D	0	0	1	0	-
IDUA	4:997858	A	G	NM_000203	c.1786A>G:p.S596G	missense	-	-	-	0.132:T	0	0	1	0	-
IDUA	4:998059	G	A	NM_000203	c.1840G>A:p.V614I	missense	-	-	-	0.281:T	0	0	1	0	-
IDUA	4:998084	C	T	NM_000203	c.1865C>T:p.A622V	missense	5.8E-05	0.0006	-	0.892:D	0	0	1	0	-
IDUA	4:998093	A	C	NM_000203	c.1874A>C:p.Y625S	missense	-	-	-	0.946:D	0	1	0	1	p.Y625C
IDUA	4:998143	C	G	NM_000203	c.1924C>G:p.P642A	missense	-	-	-	0.263:T	0	0	0	1	-
SCARB2	4:77082874	G	A	NM_005506	c.1429C>T:p.R477X	stopgain	5.80E-05	-	0	0.445:T	0	1	1	0	-
SCARB2	4:77084391	C	T	NM_005506	c.1385G>A:p.G462E	missense	0.0034	0.0025	0.0037	0.723:D	2	5	3	1	CIP
SCARB2	4:77084413	A	G	NM_005506	c.1363T>C:p.W455R	missense	-	-	-	0.752:D	0	0	0	1	-
SCARB2	4:77084445	G	A	NM_005506	c.1331C>T:p.A444V	missense	0	0	0	0.594:T	1	0	0	0	US
SCARB2	4:77084462	T	C	NM_005506	c.1314A>G:p.I438M	missense	-	-	-	0.774:D	0	0	0	1	-
SCARB2	4:77084514	G	A	NM_005506	c.1262C>T:p.T421M	missense	0.0003	0	0.0003	0.428:T	3	1	0	0	US
SCARB2	4:77084518	C	T	NM_005506	c.1258G>A:p.E420K	missense	5.8E-05	-	0.0001	0.328:T	0	0	0	1	-
SCARB2	4:77087413	T	A	NM_005506	c.1229A>T:p.Y410F	missense	5.80E-05	-	0.0001	0.563:T	1	0	4	0	US
SCARB2	4:77087432	T	C	NM_005506	c.1210A>G:p.M404V	missense	0.0019	0.0019	0.0012	0.200:T	5	5	6	3	US
SCARB2	4:77087449	G	A	NM_005506	c.1193C>T:p.T398M	missense	0.0008	-	0.0008	0.836:D	1	3	3	0	LB
SCARB2	4:77091034	C	T	NM_005506	c.1099G>A:p.V367M	missense	-	-	-	0.506:T	0	1	0	0	-
SCARB2	4:77091076	T	C	NM_005506	c.1057A>G:p.I353V	missense	0.0008	0.0012	0.0008	0.210:T	2	1	0	1	US
SCARB2	4:77091078	G	A	NM_005506	c.1055C>T:p.A352V	missense	-	-	-	0.647:T	0	0	1	0	-
SCARB2	4:77095365	G	T	NM_005506	c.926C>A:p.A309D	missense	0.0001	-	0.0001	0.689:T	0	0	0	1	-

SCARB2	4:77095377	G	A	NM_005506	c.914C>T:p.T305M	missense	0.0013	0.0019	0.0014	0.406:T	1	3	1	1	US
SCARB2	4:77095410	C	T	NM_005506	c.881G>A:p.R294Q	missense	0	-	-	0.954:D	0	0	0	1	-
SCARB2	4:77096996	G	A	NM_005506	c.772C>T:p.P258S	missense	-	-	-	0.930:D	0	1	0	0	-
SCARB2	4:77097004	G	A	NM_005506	c.764C>T:p.S255F	missense	-	-	-	0.609:T	1	0	2	0	-
SCARB2	4:77097052	C	A	NM_005506	c.716G>T:p.W239L	missense	-	0.0006	0.0001	0.942:D	0	1	0	0	US
SCARB2	4:77100702	C	T	NM_005506	c.580G>A:p.D194N	missense	0.0023	0.0031	0.003	0.196:T	21	5	13	1	LB
SCARB2	4:77100735	C	T	NM_005506	c.547G>A:p.E183K	missense	-	-	-	0.243:T	0	0	1	0	-
SCARB2	4:77100767	G	A	NM_005506	c.515C>T:p.T172I	missense	0.0013	0.0025	0.0012	0.810:D	4	9	6	8	US
SCARB2	4:77100767	G	T	NM_005506	c.515C>A:p.T172K	missense	-	-	-	0.829:D	2	0	2	2	-
SCARB2	4:77100852	T	A	NM_005506	c.430A>T:p.I144L	missense	0.0043	0.0012	0.0054	0.307:T	6	6	9	3	CIP
SCARB2	4:77102151	C	T	NM_005506	c.379G>A:p.D127N	missense	-	-	-	0.334:T	0	1	0	0	-
SCARB2	4:77102189	T	C	NM_005506	c.341A>G:p.N114S	missense	5.80E-05	-	-	0.267:T	1	0	0	0	-
SCARB2	4:77102235	T	C	NM_005506	c.295A>G:p.N99D	missense	-	-	-	0.194:T	0	0	0	1	-
SCARB2	4:77116976	C	T	NM_005506	c.159G>A:p.W53X	stopgain	-	-	-	0.723:D	0	1	0	0	-
SCARB2	4:77134590	C	T	NM_005506	c.107G>A:p.S36N	missense	-	-	-	0.057:T	0	0	1	0	-
SCARB2	4:77134591	T	G	NM_005506	c.106A>C:p.S36R	missense	0.0003	0.0006	0.0005	0.260:T	0	1	0	0	-
SCARB2	4:77134659	G	A	NM_005506	c.38C>T:p.S13F	missense	0	0	0	0.807:D	1	0	0	0	US
SCARB2	4:77134671	G	A	NM_005506	c.26C>T:p.A9V	missense	0	-	-	0.163:T	0	0	1	0	-
MANBA	4:103553246	A	G	NM_005908	c.2608T>C:p.F870L	missense	-	-	-	0.222:T	0	1	0	0	-
MANBA	4:103553312	C	T	NM_005908	c.2542G>A:p.E848K	missense	0.0011	0.0006	0.0008	0.158:T	11	4	7	9	-
MANBA	4:103553318	T	G	NM_005908	c.2536A>C:p.M846L	missense	0	0	0	0.243:T	0	1	0	1	-
MANBA	4:103553362	T	C	NM_005908	c.2492A>G:p.D831G	missense	0	-	-	0.939:D	1	0	0	0	-
MANBA	4:103553365	A	C	NM_005908	c.2489T>G:p.L830W	missense	-	-	-	0.903:D	1	1	0	0	US
MANBA	4:103553381	C	A	NM_005908	c.2473G>T:p.A825S	missense	0.0066	0.0055	0.0068	0.569:T	21	13	15	7	-
MANBA	4:103553381	C	T	NM_005908	c.2473G>A:p.A825T	missense	0.0003	-	0.0002	0.613:T	2	1	4	0	US
MANBA	4:103553402	C	-	NM_005908	c.2452delG:p.D818Tfs*14	frameshift deletion	-	-	-	-	0	0	1	0	-
MANBA	4:103553419	C	G	NM_005908	c.2435G>C:p.G812A	missense	0	-	-	0.057:T	1	0	0	0	-
MANBA	4:103555951	C	G	NM_005908	c.2409G>C:p.Q803H	missense	0	-	-	0.193:T	0	0	1	0	-
MANBA	4:103556064	G	A	NM_005908	c.2296C>T:p.R766W	missense	0	0	0	0.918:D	0	0	1	0	US
MANBA	4:103556159	C	T	NM_005908	c.2201G>A:p.R734H	missense	0	0	0	0.011:T	0	1	0	0	US
MANBA	4:103557065	T	C	NM_005908	c.2114A>G:p.Y705C	missense	0.0001	-	0.0001	0.786:D	1	0	0	0	-

MANBA	4:103557091	A	-	NM_005908	c.2088delT:p.F696Lfs*20	frameshift deletion	-	-	-	-	0	1	0	0	-
MANBA	4:103557118	G	T	NM_005908	c.2061C>A:p.F687L	missense	-	-	-	0.841:D	0	3	0	0	-
MANBA	4:103557142	C	G	NM_005908	c.2037G>C:p.M679I	missense	0.0003	-	0.0003	0.678:T	1	1	0	0	-
MANBA	4:103557142	C	T	NM_005908	c.2037G>A:p.M679I	missense	5.8E-05	0.0006	0.0001	0.678:T	0	0	0	1	-
MANBA	4:103560929	A	G	NM_005908	c.1955T>C:p.M652T	missense	-	-	-	0.930:D	0	1	0	0	-
MANBA	4:103560951	C	G	NM_005908	c.1933G>C:p.V645L	missense	-	-	-	0.410:T	1	0	0	0	-
MANBA	4:103560971	C	T	NM_005908	c.1913G>A:p.R638H	missense	0.0009	0.0031	0.0007	0.722:D	3	2	3	1	-
MANBA	4:103560996	C	T	NM_005908	c.1888G>A:p.V630I	missense	-	-	-	0.217:T	0	1	0	1	-
MANBA	4:103571717	T	C	NM_005908	c.1846A>G:p.K616E	missense	0.0002	0.0006	0.0002	0.334:T	3	5	1	3	-
MANBA	4:103571829	A	T	NM_005908	c.1734T>A:p.N578K	missense	-	-	-	0.278:T	1	0	0	0	-
MANBA	4:103571854	G	A	NM_005908	c.1709C>T:p.S570L	missense	0.0015	0.0012	0.0015	0.916:D	1	0	0	1	-
MANBA	4:103578864	C	A	NM_005908	c.1679G>T:p.W560L	missense	-	-	-	0.807:D	4	0	1	1	-
MANBA	4:103578873	T	C	NM_005908	c.1670A>G:p.Y557C	missense	-	-	-	0.827:D	0	1	0	1	-
MANBA	4:103578894	C	T	NM_005908	c.1649G>A:p.R550Q	missense	0	-	0	0.921:D	1	0	1	0	US
MANBA	4:103578898	C	G	NM_005908	c.1645G>C:p.A549P	missense	-	-	-	0.774:D	0	0	1	0	-
MANBA	4:103578929	A	T	NM_005908	c.1614T>A:p.S538R	missense	-	-	-	0.375:T	0	0	1	0	-
MANBA	4:103585856	C	G	NM_005908	c.1471G>C:p.E491Q	missense	-	-	-	0.323:T	0	0	1	0	-
MANBA	4:103585906	C	T	NM_005908	c.1421G>A:p.R474Q	missense	0.0001	-	0.0001	0.257:T	0	0	1	1	-
MANBA	4:103585927	T	C	NM_005908	c.1400A>G:p.Y467C	missense	-	-	-	0.660:T	0	2	0	0	-
MANBA	4:103585927	T	-	NM_005908	c.1400delA:p.Y467Ffs*15	frameshift deletion	-	-	-	-	0	1	0	0	-
MANBA	4:103585931	AATTCATC	-	NM_005908	c.1389_1396del:p.M464Vfs*6	frameshift deletion	-	-	-	-	0	1	0	0	-
MANBA	4:103585952	C	T	NM_005908	c.1375G>A:p.E459K	missense	0.0003	0.0006	0.0001	0.962:D	0	1	1	0	-
MANBA	4:103585963	T	C	NM_005908	c.1364A>G:p.N455S	missense	-	-	-	0.641:T	1	1	0	0	-
MANBA	4:103585969	C	T	NM_005908	c.1358G>A:p.S453N	missense	0	-	-	0.664:T	0	1	0	0	-
MANBA	4:103585988	G	A	NM_005908	c.1339C>T:p.P447S	missense	0.0006	-	0.0008	0.907:D	3	2	0	1	-
MANBA	4:103590136	G	C	NM_005908	c.1301C>G:p.A434G	missense	-	-	-	0.255:T	0	0	1	0	-
MANBA	4:103590206	C	T	NM_005908	c.1231G>A:p.V411I	missense	0.0002	-	-	0.300:T	0	0	1	0	-
MANBA	4:103592476	C	A	NM_005908	c.1192G>T:p.D398Y	missense	5.8E-05	-	-	0.981:D	0	0	0	1	-
MANBA	4:103592506	G	A	NM_005908	c.1162C>T:p.R388W	missense	0	0	0	0.982:D	0	0	0	1	-

MANBA	4:103592551	G	A	NM_005908	c.1117C>T:p.R373W	missense	0	-	0	0.411:T	1	3	0	1	US
MANBA	4:103610756	C	G	NM_005908	c.935G>C:p.G312A	missense	0.0001	-	-	0.290:T	0	0	0	1	US
MANBA	4:103611815	T	C	NM_005908	c.787A>G:p.T263A	missense	-	-	-	0.041:T	0	2	0	1	-
MANBA	4:103611842	C	T	NM_005908	c.760G>A:p.A254T	missense	-	-	-	0.026:T	0	0	1	0	-
MANBA	4:103644044	A	C	NM_005908	c.533T>G:p.V178G	missense	-	-	-	0.845:D	1	0	0	0	-
MANBA	4:103644047	T	C	NM_005908	c.530A>G:p.H177R	missense	0.0037	0.0049	0.003	0.677:T	13	10	15	4	-
MANBA	4:103644074	G	A	NM_005908	c.503C>T:p.P168L	missense	-	-	-	0.802:D	1	0	0	0	-
MANBA	4:103644075	G	A	NM_005908	c.502C>T:p.P168S	missense	5.80E-05	-	-	0.747:D	1	0	0	0	-
MANBA	4:103644090	C	T	NM_005908	c.487G>A:p.V163I	missense	5.80E-05	-	0.0001	0.357:T	1	0	1	0	-
MANBA	4:103644102	T	C	NM_005908	c.475A>G:p.T159A	missense	-	-	-	0.164:T	1	0	0	0	-
MANBA	4:103644108	C	G	NM_005908	c.469G>C:p.A157P	missense	0.0003	0.0006	0.0001	0.565:T	3	2	4	3	-
MANBA	4:103644158	A	G	NM_005908	c.419T>C:p.I140T	missense	0	-	-	0.743:D	0	1	0	0	-
MANBA	4:103644191	T	C	NM_005908	c.386A>G:p.D129G	missense	0.0005	0.0012	0.0005	0.867:D	1	1	1	0	-
MANBA	4:103645050	A	G	NM_005908	c.347T>C:p.I116T	missense	0.0001	0	0	0.864:D	0	1	0	0	US
MANBA	4:103645057	C	T	NM_005908	c.340G>A:p.V114I	missense	0.0001	-	-	0.073:T	1	1	1	0	-
MANBA	4:103645117	G	T	NM_005908	c.280C>A:p.Q94K	missense	0.001	0.0012	0.0009	0.362:T	5	12	11	6	-
MANBA	4:103647753	C	A	NM_005908	c.265G>T:p.E89X	stopgain	-	-	-	0.499:T	0	1	0	1	-
MANBA	4:103647794	AA	-	NM_005908	c.223_224del:p.L75Gfs*1	frameshift deletion	-	-	-	-	0	1	0	0	-
MANBA	4:103647812	T	C	NM_005908	c.206A>G:p.N69S	missense	5.81E-05	-	0.0001	0.081:T	2	1	5	1	-
MANBA	4:103647821	T	C	NM_005908	c.197A>G:p.N66S	missense	0.0002	-	0.0002	0.614:T	1	2	0	0	-
MANBA	4:103681927	C	T	NM_005908	c.125G>A:p.G42E	missense	-	-	-	0.786:D	0	0	1	0	-
MANBA	4:103682023	G	A	NM_005908	c.29C>T:p.A10V	missense	-	-	-	0.223:T	1	0	0	0	-
MFSD8	4:128841850	G	A	NM_152778	c.1492C>T:p.L498F	missense	-	-	-	0.484:T	0	1	0	0	-
MFSD8	4:128841870	A	T	NM_152778	c.1472T>A:p.I491K	missense	-	-	-	0.770:D	1	0	0	0	-
MFSD8	4:128841898	G	A	NM_152778	c.1444C>T:p.R482X	stopgain	0	0.0006	0	0.691:T	0	0	1	0	P/LP
MFSD8	4:128841934	T	C	NM_152778	c.1408A>G:p.M470V	missense	0	-	0	0.747:D	0	0	1	0	US
MFSD8	4:128841951	G	A	NM_152778	c.1391C>T:p.A464V	missense	0	0.0006	0	0.949:D	0	1	0	1	US
MFSD8	4:128842683	G	A	NM_152778	c.1346C>T:p.P449L	missense	0.0001	-	0.0001	0.891:D	3	0	2	0	-
MFSD8	4:128842699	T	G	NM_152778	c.1330A>C:p.I444L	missense	-	-	-	0.425:T	1	0	0	0	-
MFSD8	4:128842721	C	T	NM_152778	c.1308G>A:p.M436I	missense	5.8E-05	-	-	0.540:T	0	0	0	1	-
MFSD8	4:128842728	T	C	NM_152778	c.1301A>G:p.N434S	missense	-	-	-	0.172:T	0	0	1	1	-

MFSD8	4:128842753	T	A	NM_152778	c.1276A>T:p.I426L	missense	-	-	-	0.312:T	0	0	1	0	-
MFSD8	4:128842804	G	A	NM_152778	c.1225C>T:p.L409F	missense	0	-	-	0.343:T	0	1	0	0	-
MFSD8	4:128842816	G	T	NM_152778	c.1213C>A:p.Q405K	missense	5.80E-05	-	0.0001	0.801:D	1	1	0	0	-
MFSD8	4:128842824	G	A	NM_152778	c.1205C>T:p.S402L	missense	0	0	0	0.505:T	0	0	1	0	US
MFSD8	4:128842836	G	C	NM_152778	c.1193C>G:p.P398R	missense	-	-	-	0.832:D	0	0	1	0	-
MFSD8	4:128842848	T	C	NM_152778	c.1181A>G:p.D394G	missense	-	-	-	0.305:T	0	0	1	0	-
MFSD8	4:128842878	A	G	NM_152778	c.1151T>C:p.I384T	missense	5.80E-05	-	0.0001	0.564:T	0	1	0	0	-
MFSD8	4:128842913	A	T	NM_152778	c.1116T>A:p.N372K	missense	0.0002	-	0.0002	0.333:T	1	2	1	0	US
MFSD8	4:128843028	T	G	NM_152778	c.1089A>C:p.K363N	missense	-	-	-	0.293:T	0	1	0	0	-
MFSD8	4:128843060	T	C	NM_152778	c.1057A>G:p.I353V	missense	0.0001	-	0.0001	0.260:T	0	1	0	0	-
MFSD8	4:128843081	C	T	NM_152778	c.1036G>A:p.V346I	missense	0	-	0	0.265:T	1	0	0	0	US
MFSD8	4:128851875	C	T	NM_152778	c.961G>A:p.V321I	missense	5.80E-05	-	0	0.211:T	0	1	0	0	US
MFSD8	4:128851896	C	T	NM_152778	c.940G>A:p.A314T	missense	-	-	-	0.415:T	0	0	0	1	US
MFSD8	4:128851931	T	C	NM_152778	c.905A>G:p.Q302R	missense	-	-	-	0.278:T	0	0	0	1	-
MFSD8	4:128859985	C	T	NM_152778	c.707G>A:p.R236H	missense	0	0	0	0.404:T	0	0	1	0	US
MFSD8	4:128861011	A	G	NM_152778	c.695T>C:p.L232P	missense	-	-	-	0.845:D	0	2	0	0	-
MFSD8	4:128861032	A	G	NM_152778	c.674T>C:p.I225T	missense	-	-	-	0.797:D	2	0	0	0	-
MFSD8	4:128861077	T	C	NM_152778	c.629A>G:p.Y210C	missense	-	-	-	0.918:D	1	0	0	0	-
MFSD8	4:128861117	C	G	NM_152778	c.589G>C:p.G197R	missense	-	-	-	0.943:D	0	1	0	0	-
MFSD8	4:128861149	A	G	NM_152778	c.557T>C:p.F186S	missense	-	-	-	0.870:D	0	1	0	0	-
MFSD8	4:128863212	T	A	NM_152778	c.541A>T:p.I181F	missense	0	-	0	0.855:D	0	0	1	0	-
MFSD8	4:128865003	C	T	NM_152778	c.343G>A:p.V115M	missense	0.001	0.0019	0.0015	0.692:T	6	2	1	3	US
MFSD8	4:128865090	C	T	NM_152778	c.256G>A:p.G86S	missense	0.0007	0.0006	0.001	0.939:D	0	0	1	1	-
MFSD8	4:128865128	-	T	NM_152778	c.217dupA:p.T73Nfs*11	frameshift insertion	0.0002	-	-	-	1	0	2	0	P
MFSD8	4:128865140	G	A	NM_152778	c.206C>T:p.P69L	missense	0.0034	0.0019	0.0028	0.351:T	17	17	11	12	CIP
MFSD8	4:128870994	C	T	NM_152778	c.163G>A:p.V55I	missense	-	-	-	0.302:T	1	0	0	0	-
MFSD8	4:128870994	C	G	NM_152778	c.163G>C:p.V55L	missense	-	-	-	0.442:T	0	0	1	0	-
MFSD8	4:128878713	T	C	NM_152778	c.97A>G:p.K33E	missense	-	-	-	0.486:T	0	1	0	1	-
MFSD8	4:128878732	T	G	NM_152778	c.78A>C:p.L26F	missense	0.0002	-	0.0001	0.208:T	1	2	1	0	-
MFSD8	4:128886269	T	C	NM_152778	c.20A>G:p.E7G	missense	-	-	-	0.283:T	0	0	1	0	-

MFSD8	4:128886271	G	C	NM_152778	c.18C>G:p.N6K	missense	0.0013	0.0006	0.0008	0.155:T	10	2	3	0	US
MFSD8	4:128886282	C	T	NM_152778	c.7G>A:p.G3S	missense	5.8E-05	-	0.0001	0.240:T	0	0	1	0	US
MFSD8	4:128886286	C	G	NM_152778	c.3G>C:p.M1I	missense	-	-	-	0.741:D	0	0	3	0	-
AGA	4:178354368	C	T	NM_000027	c.940G>A:p.G314S	missense	5.80E-05	-	0	0.969:D	1	0	0	0	-
AGA	4:178354398	C	T	NM_000027	c.910G>A:p.V304I	missense	0.0001	-	0.0001	0.505:T	0	1	0	0	-
AGA	4:178354427	T	C	NM_000027	c.881A>G:p.Q294R	missense	-	-	-	0.274:T	0	1	0	0	-
AGA	4:178355548	C	A	NM_000027	c.794G>T:p.R265L	missense	5.8E-05	-	0	0.995:D	0	0	1	0	-
AGA	4:178355597	C	T	NM_000027	c.745G>A:p.D249N	missense	0.001	0.0006	0.0013	0.332:T	1	4	4	0	-
AGA	4:178357432	A	T	NM_000027	c.696T>A:p.H232Q	missense	-	-	-	0.278:T	1	0	1	1	-
AGA	4:178357470	C	A	NM_000027	c.658G>T:p.A220S	missense	-	-	-	0.566:T	1	0	0	0	-
AGA	4:178358583	C	T	NM_000027	c.598G>A:p.D200N	missense	0.0009	0.0019	0.0006	0.228:T	2	0	0	0	US
AGA	4:178359924	C	T	NM_000027	c.482G>A:p.R161Q	missense	5.80E-05	0	0.0001	0.772:D	0	1	0	1	LB
AGA	4:178359925	G	A	NM_000027	c.481C>T:p.R161W	missense	0	0	0	0.508:T	0	1	0	1	-
AGA	4:178359967	A	G	NM_000027	c.439T>C:p.S147P	missense	0	0	-	0.934:D	1	0	0	0	LP
AGA	4:178360732	G	A	NM_000027	c.392C>T:p.S131L	missense	-	-	-	0.897:D	1	0	0	0	-
AGA	4:178360811	G	T	NM_000027	c.313C>A:p.L105I	missense	0	0	0	0.614:T	0	1	0	0	B/LB
AGA	4:178361516	A	-	NM_000027	c.192delT:p.C64Wfs*9	frameshift deletion	-	-	-	-	0	1	0	0	LP
AGA	4:178363412	T	A	NM_000027	c.118A>T:p.T40S	missense	-	-	-	0.460:T	1	0	0	0	-
AGA	4:178363474	G	A	NM_000027	c.56C>T:p.A19V	missense	-	-	-	0.359:T	0	0	0	1	-
AGA	4:178363502	G	A	NM_000027	c.28C>T:p.L10F	missense	0	-	0	0.308:T	1	0	0	0	-
AGA	4:178363513	T	A	NM_000027	c.17A>T:p.N6I	missense	-	-	-	0.438:T	0	1	0	0	-
AGA	4:178363517	A	G	NM_000027	c.13T>C:p.S5P	missense	5.80E-05	-	0.0001	0.396:T	1	0	1	0	-
AGA	4:178363521	C	-	NM_000027	c.9delG:p.K4Sfs*16	frameshift deletion	-	-	-	-	1	0	0	0	-
AGA	4:178363523	G	A	NM_000027	c.7C>T:p.R3W	missense	-	-	-	0.342:T	0	0	1	1	-
AGA	4:178363525	G	A	NM_000027	c.5C>T:p.A2V	missense	-	-	-	0.484:T	0	0	1	0	-
HEXB	5:73981180	A	T	NM_000521	c.95A>T:p.Q32L	missense	-	-	-	0.283:T	1	0	0	0	p.Q32X
HEXB	5:73981221	C	T	NM_000521	c.136C>T:p.P46S	missense	-	-	-	0.414:T	1	0	0	0	-
HEXB	5:73981249	C	T	NM_000521	c.164C>T:p.A55V	missense	0.0008	0.0012	0.001	0.282:T	2	1	2	0	-
HEXB	5:73981306	C	G	NM_000521	c.221C>G:p.P74R	missense	-	-	-	0.378:T	0	0	1	0	-
HEXB	5:73981330	G	A	NM_000521	c.245G>A:p.S82N	missense	-	-	-	0.154:T	0	0	0	1	-

HEXB	5:73981336	A	G	NM_000521	c.251A>G:p.N84S	missense	0	0.0006	0	0.196:T	0	0	1	1	-
HEXB	5:73981341	A	G	NM_000521	c.256A>G:p.T86A	missense	-	-	-	0.283:T	1	0	0	0	-
HEXB	5:73985164	A	G	NM_000521	c.311A>G:p.Y104C	missense	0.0001	0.0006	0	0.873:D	1	2	0	0	US
HEXB	5:73985199	G	C	NM_000521	c.346G>C:p.A116P	missense	-	-	-	0.321:T	0	1	0	0	-
HEXB	5:73985299	-	T	NM_000521	c.445+1->T	splicing	-	-	-	-	1	0	0	0	-
HEXB	5:73992868	-	A	NM_000521	c.607dupA:p.R203Kfs*5	frameshift insertion	-	-	-	-	0	0	1	0	-
HEXB	5:74001048	C	T	NM_000521	c.674C>T:p.A225V	missense	-	-	-	0.843:D	1	0	0	0	-
HEXB	5:74009338	A	T	NM_000521	c.779A>T:p.Y260F	missense	-	-	-	0.493:T	1	0	0	0	-
HEXB	5:74009362	C	A	NM_000521	c.803C>A:p.P268Q	missense	-	-	-	0.482:T	0	1	0	0	-
HEXB	5:74009373	C	T	NM_000521	c.814C>T:p.R272C	missense	0	-	0	0.657:T	1	0	0	0	-
HEXB	5:74009383	T	C	NM_000521	c.824T>C:p.I275T	missense	0.0006	0.0006	0.0003	0.753:D	1	1	1	0	-
HEXB	5:74009412	G	A	NM_000521	c.853G>A:p.V285I	missense	-	-	-	0.463:T	0	0	0	1	-
HEXB	5:74011499	G	A	NM_000521	c.1066G>A:p.V356M	missense	0.0003	0.0006	0.0001	0.806:D	3	1	0	0	-
HEXB	5:74014136	C	T	NM_000521	c.1190C>T:p.T397I	missense	-	-	-	0.369:T	2	0	0	0	-
HEXB	5:74014626	C	T	NM_000521	c.1247C>T:p.A416V	missense	5.80E-05	0	0	0.399:T	1	0	1	0	-
HEXB	5:74014629	C	T	NM_000521	c.1250C>T:p.P417L	missense	0.001	0.0006	0.0016	0.851:D	1	0	0	0	P
HEXB	5:74014655	G	A	NM_000521	c.1276G>A:p.D426N	missense	0.0001	-	0.0001	0.014:T	2	2	0	2	-
HEXB	5:74014759	G	T	NM_000521	c.1380G>T:p.W460C	missense	-	-	-	0.982:D	1	0	0	0	-
HEXB	5:74014778	G	A	NM_000521	c.1399G>A:p.E467K	missense	5.80E-05	-	-	0.649:T	0	1	0	0	-
HEXB	5:74014783	T	-	NM_000521	c.1404delT:p.D470Ifs*59	frameshift deletion	-	-	-	-	1	0	0	0	-
HEXB	5:74016265	AG	-	NM_000521	c.1430_1431del:p.K478Tfs*18	frameshift deletion	0	-	0	-	0	0	1	0	-
HEXB	5:74016472	C	T	NM_000521	c.1513C>T:p.R505W	missense	5.80E-05	0.0012	0.0001	0.787:D	1	0	0	0	US
HEXB	5:74016517	A	G	NM_000521	c.1558A>G:p.R520G	missense	0	-	-	0.536:T	1	0	1	0	-
HEXB	5:74016520	G	T	NM_000521	c.1561G>T:p.D521Y	missense	5.80E-05	-	-	0.627:T	2	0	0	0	-
HEXB	5:74016523	A	G	NM_000521	c.1564A>G:p.M522V	missense	-	-	-	0.315:T	0	1	0	0	-
HEXB	5:74016954	C	A	NM_000521	c.1625C>A:p.A542D	missense	0	-	0	0.645:T	0	1	0	0	-
HEXB	5:74016956	G	A	NM_000521	c.1627G>A:p.A543T	missense	0	0	0	0.859:D	1	0	0	0	-
HEXB	5:74016978	-	TTGTA	NM_000521	c.1649_1650insTTGTA	stoploss	0	-	0	-	1	1	1	0	-

			ACCATGAGAACATG		ACCATGAGAACATG:p.*557delinsL*										
HEXB	5:74016981	G	A	NM_000521	c.1652G>A:p.C551Y	missense	-	-	-	0.999:D	0	0	1	0	US
AP3B1	5:77298814	G	A	NM_003664	c.3197C>T:p.S1066F	missense	0.0008	0.0037	0.0009	0.420:T	12	1	4	6	-
AP3B1	5:77311330	G	A	NM_003664	c.3035C>T:p.A1012V	missense	-	-	-	0.084:T	1	0	0	0	-
AP3B1	5:77311342	G	T	NM_003664	c.3023C>A:p.A1008D	missense	-	-	-	0.484:T	1	0	0	0	-
AP3B1	5:77330230	C	T	NM_003664	c.2849G>A:p.G950D	missense	-	-	-	0.964:D	1	0	0	0	-
AP3B1	5:77334870	T	C	NM_003664	c.2806A>G:p.I936V	missense	5.83E-05	-	0	0.170:T	2	1	2	0	-
AP3B1	5:77334896	C	T	NM_003664	c.2780G>A:p.G927D	missense	-	-	-	0.637:T	0	1	0	0	-
AP3B1	5:77334897	C	T	NM_003664	c.2779G>A:p.G927S	missense	0.0009	-	0.0004	0.458:T	0	2	2	1	US
AP3B1	5:77335015	G	T	NM_003664	c.2661C>A:p.F887L	missense	0.0001	0.0006	0	0.147:T	0	1	1	1	CIP
AP3B1	5:77385222	T	A	NM_003664	c.2572A>T:p.I858F	missense	-	-	-	0.648:T	0	1	0	0	-
AP3B1	5:77385258	G	C	NM_003664	c.2536C>G:p.L846V	missense	-	-	-	0.357:T	1	0	0	0	-
AP3B1	5:77385259	A	T	NM_003664	c.2535T>A:p.D845E	missense	-	-	-	0.496:T	0	1	0	0	-
AP3B1	5:77385273	T	C	NM_003664	c.2521A>G:p.S841G	missense	0	-	0	0.262:T	0	1	5	1	-
AP3B1	5:77396842	T	C	NM_003664	c.2405A>G:p.E802G	missense	-	-	-	0.124:T	0	0	0	1	US
AP3B1	5:77409586	T	G	NM_003664	c.2239A>C:p.K747Q	missense	-	-	-	0.183:T	1	0	0	0	-
AP3B1	5:77409592	T	C	NM_003664	c.2233A>G:p.K745E	missense	-	-	-	0.128:T	0	1	0	0	-
AP3B1	5:77409636	C	T	NM_003664	c.2189G>A:p.R730Q	missense	0	-	-	0.069:T	0	0	1	0	-
AP3B1	5:77409649	T	C	NM_003664	c.2176A>G:p.S726G	missense	-	-	-	0.266:T	1	0	0	0	-
AP3B1	5:77409693	T	A	NM_003664	c.2132A>T:p.D711V	missense	0.0003	-	0.0007	0.292:T	6	0	3	2	US
AP3B1	5:77409744	C	T	NM_003664	c.2081G>A:p.S694N	missense	0.0002	-	-	0.312:T	1	0	0	0	-
AP3B1	5:77411968	C	T	NM_003664	c.2059G>A:p.D687N	missense	-	-	-	0.109:T	0	0	0	1	-
AP3B1	5:77412020	C	A	NM_003664	c.2007G>T:p.E669D	missense	0.0001	-	0.0002	0.046:T	0	0	1	0	-
AP3B1	5:77412058	C	G	NM_003664	c.1969G>C:p.A657P;-	missense	-	-	-	0.677:T	1	0	0	0	-
AP3B1	5:77423871	C	G	NM_003664	c.1951G>C:p.V651L	missense	0.0002	-	0.0003	0.687:T	0	1	1	1	US
AP3B1	5:77425064	C	T	NM_003664	c.1718G>A:p.R573H	missense	0	-	-	0.822:D	1	0	0	0	US
AP3B1	5:77436977	T	C	NM_003664	c.1640A>G:p.N547S	missense	0	-	-	0.525:T	0	1	0	0	-
AP3B1	5:77437095	C	T	NM_003664	c.1522G>A:p.E508K	missense	0	-	-	0.744:D	1	0	0	0	-
AP3B1	5:77452129	G	A	NM_003664	c.1426C>T:p.H476Y	missense	-	-	-	0.819:D	0	0	0	1	-
AP3B1	5:77452187	T	C	NM_003664	c.1368A>G:p.I456M	missense	-	-	-	0.191:T	1	0	0	0	-

AP3B1	5:77458693	A	G	NM_003664	c.1313T>C:p.V438A	missense	-	-	-	0.875:D	0	1	0	0	-
AP3B1	5:77461448	G	A	NM_003664	c.1216C>T:p.L406F	missense	0.0001	-	0.0001	0.897:D	0	0	1	0	-
AP3B1	5:77471628	T	C	NM_003664	c.1075A>G:p.T359A	missense	0.0003	0.0006	0.0002	0.802:D	0	5	4	2	US
AP3B1	5:77473172	C	T	NM_003664	c.1031G>A:p.R344H	missense	0	0	0	0.508:T	1	0	0	0	US
AP3B1	5:77473181	C	T	NM_003664	c.1022G>A:p.R341H	missense	0	0	0	0.940:D	1	0	0	0	US
AP3B1	5:77477362	G	A	NM_003664	c.911C>T:p.T304I	missense	0.0007	-	0.0002	0.840:D	5	3	0	0	-
AP3B1	5:77477369	T	C	NM_003664	c.904A>G:p.R302G	missense	0	-	0	0.888:D	1	1	0	0	p.R302X
AP3B1	5:77477431	T	C	NM_003664	c.842A>G:p.K281R	missense	0	0.0006	-	0.021:T	0	1	1	0	-
AP3B1	5:77477452	T	C	NM_003664	c.821A>G:p.Y274C	missense	0	-	-	0.869:D	1	0	0	0	US
AP3B1	5:77511886	C	G	NM_003664	c.779G>C:p.W260S	missense	-	-	-	0.572:T	0	1	0	0	p.W260X
AP3B1	5:77511985	C	T	NM_003664	c.680G>A:p.R227H	missense	5.81E-05	-	0.0001	0.878:D	2	0	1	0	-
AP3B1	5:77511986	G	A	NM_003664	c.679C>T:p.R227C	missense	0	0	0	0.864:D	0	1	0	0	US
AP3B1	5:77524051	C	T	NM_003664	c.292G>A:p.V98I	missense	0.0013	0.0006	0.0011	0.614:T	0	0	1	0	US
AP3B1	5:77590298	G	A	NM_003664	c.106C>T:p.L36F	missense	-	-	-	0.378:T	1	0	0	0	-
AP3B1	5:77590300	C	A	NM_003664	c.104G>T:p.G35V	missense	-	-	-	0.610:T	0	1	0	0	-
AP3B1	5:77590318	G	A	NM_003664	c.86C>T:p.S29F	missense	0	0	0	0.342:T	1	0	0	0	-
AP3B1	5:77590339	T	A	NM_003664	c.65A>T:p.Q22L	missense	0.0001	-	-	0.542:T	1	2	1	1	US
AP3B1	5:77590361	C	T	NM_003664	c.43G>A:p.G15R	missense	5.8E-05	-	-	0.479:T	0	0	1	0	-
AP3B1	5:77590378	T	C	NM_003664	c.26A>G:p.N9S	missense	0	-	-	0.131:T	0	0	1	0	US
AP3B1	5:77590396	C	G	NM_003664	c.8G>C:p.S3T	missense	5.80E-05	-	-	0.192:T	2	0	1	1	-
ARSB	5:78076245	G	T	NM_000046	c.1577C>A:p.T526N	missense	-	-	-	0.479:T	0	1	0	0	-
ARSB	5:78076263	C	T	NM_000046	c.1559G>A:p.R520H	missense	5.80E-05	0	-	0.444:T	0	1	1	0	-
ARSB	5:78076288	C	T	NM_000046	c.1534G>A:p.V512M	missense	5.80E-05	0	0	0.369:T	0	1	0	0	-
ARSB	5:78076305	T	C	NM_000046	c.1517A>G:p.H506R	missense	-	0	0	0.463:T	0	1	0	0	-
ARSB	5:78076320	C	T	NM_000046	c.1502G>A:p.R501H	missense	5.80E-05	-	0	0.809:D	1	0	0	0	US
ARSB	5:78076429	A	T	NM_000046	c.1393T>A:p.S465T	missense	-	-	-	0.261:T	0	0	1	0	-
ARSB	5:78076435	G	T	NM_000046	c.1387C>A:p.P463T	missense	0.0002	0.0006	0.0002	0.482:T	0	2	1	0	-
ARSB	5:78076468	G	A	NM_000046	c.1354C>T:p.P452S	missense	-	-	-	0.839:D	0	0	1	0	-
ARSB	5:78076471	A	G	NM_000046	c.1351T>C:p.F451L	missense	-	-	-	0.666:T	0	0	1	0	-
ARSB	5:78077732	T	G	NM_000046	c.1279A>C:p.T427P	missense	0	-	0	0.746:D	1	0	0	0	-
ARSB	5:78077740	G	C	NM_000046	c.1271C>G:p.A424G	missense	0	-	0	0.393:T	0	1	1	0	-
ARSB	5:78077756	G	A	NM_000046	c.1255C>T:p.L419F	missense	0	-	0	0.241:T	1	0	0	0	-

ARSB	5:78111928	T	G	NM_198709	c.1223A>C:p.E408A	missense	-	-	-	0.281:T	1	0	0	0	-
ARSB	5:78135195	G	C	NM_000046	c.1197C>G:p.F399L	missense	0.0002	-	0.0002	0.850:D	2	0	0	0	P/LP
ARSB	5:78135202	G	A	NM_000046	c.1190C>T:p.P397L	missense	-	-	-	0.851:D	0	0	1	1	-
ARSB	5:78135241	C	T	NM_000046	c.1151G>A:p.S384N	missense	5.80E-05	0	0.0001	0.279:T	0	1	1	2	B
ARSB	5:78181423	C	T	NM_000046	c.1126G>A:p.V376M	missense	0.0138	0.0197	0.0133	0.333:T	51	43	29	26	B
ARSB	5:78181477	C	A	NM_000046	c.1072G>T:p.V358L	missense	-	-	-	0.636:T	0	1	0	0	-
ARSB	5:78181525	C	T	NM_000046	c.1024G>A:p.V342M	missense	0	-	0	0.354:T	2	0	0	0	-
ARSB	5:78181570	G	A	NM_000046	c.979C>T:p.R327X	stopgain	0	-	-	0.746:D	0	1	0	0	P
ARSB	5:78181645	C	T	NM_000046	c.904G>A:p.G302R	missense	0	-	0	1.000:D	0	1	0	0	US
ARSB	5:78251159	C	G	NM_000046	c.857G>C:p.S286T	missense	0	-	-	0.450:T	1	0	0	0	-
ARSB	5:78251178	C	T	NM_000046	c.838G>A:p.V280I	missense	-	-	-	0.492:T	0	1	0	0	-
ARSB	5:78251202	G	A	NM_000046	c.814C>T:p.L272F	missense	0.0001	-	-	0.518:T	0	1	0	2	-
ARSB	5:78260271	T	C	NM_000046	c.658A>G:p.I220V	missense	0.001	0.0006	0.0014	0.400:T	2	5	4	2	-
ARSB	5:78260274	C	A	NM_000046	c.655G>T:p.A219S	missense	-	-	-	0.874:D	0	0	1	0	-
ARSB	5:78260304	T	C	NM_000046	c.625A>G:p.M209V	missense	-	-	-	0.615:T	1	0	2	1	-
ARSB	5:78264830	A	C	NM_000046	c.498T>G:p.F166L	missense	-	-	-	0.794:D	0	0	1	0	-
ARSB	5:78264969	C	G	NM_000046	c.359G>C:p.S120T	missense	-	-	-	0.372:T	0	1	0	0	-
ARSB	5:78265013	-	AT	NM_000046	c.314_315insAT:p.R106Sfs*8	frameshift insertion	-	-	-	-	0	0	1	0	-
ARSB	5:78265016	C	A	NM_000046	c.313-1G>T	splicing	-	-	-	0.768:D	0	1	1	0	-
ARSB	5:78280774	T	C	NM_000046	c.298A>G:p.T100A	missense	-	-	-	0.885:D	0	1	0	1	-
ARSB	5:78280881	C	T	NM_000046	c.191G>A:p.G64D	missense	0.0002	-	0.0002	0.644:T	1	0	0	1	-
ARSB	5:78281029	G	C	NM_000046	c.43C>G:p.P15A	missense	0.0018	0.0025	0.0049	0.457:T	1	2	0	0	US
ARSB	5:78281383	-	AA	NM_198709	UTR5	splicing	-	0.0019	-	-	0	0	2	0	-
GM2A	5:150632778	A	G	NM_000405	c.1A>G:p.M1V	missense	-	-	-	0.878:D	1	0	0	0	-
GM2A	5:150632790	A	T	NM_000405	c.13A>T:p.M5L	missense	0.0006	0.0006	0.0006	0.348:T	2	5	5	2	US
GM2A	5:150632791	T	C	NM_000405	c.14T>C:p.M5T	missense	0	0	0	0.128:T	0	0	1	0	-
GM2A	5:150632800	C	G	NM_000405	c.23C>G:p.P8R	missense	-	-	-	0.313:T	0	0	1	1	-
GM2A	5:150632811	G	T	NM_000405	c.34G>T:p.A12S	missense	0	-	-	0.061:T	1	0	1	0	-
GM2A	5:150632812	C	T	NM_000405	c.35C>T:p.A12V	missense	5.8E-05	-	-	0.045:T	0	0	1	0	-
GM2A	5:150632832	G	T	NM_000405	c.55G>T:p.A19S	missense	-	-	-	0.033:T	0	1	0	0	-

GM2A	5:150632855	A	T	NM_000405	c.78A>T:p.K26N	missense	0.0059	0.0062	0.0049	0.020:T	39	15	24	19	LB
GM2A	5:150632856	A	G	NM_000405	c.79A>G:p.K27E	missense	5.80E-05	-	-	0.125:T	0	2	0	0	-
GM2A	5:150639392	TG	-	NM_000405	c.158_159del:p.L53Rfs*2	frameshift deletion	-	-	-	-	0	1	1	1	-
GM2A	5:150639407	T	C	NM_000405	c.173T>C:p.I58T	missense	0.0006	0.0018	0.0003	0.764:D	3	0	1	0	-
GM2A	5:150639412	G	T	NM_000405	c.178G>T:p.V60F	missense	0.0001	0	0.0001	0.414:T	3	0	0	0	-
GM2A	5:150639412	G	A	NM_000405	c.178G>A:p.V60I	missense	0	-	0	0.166:T	0	0	2	0	-
GM2A	5:150646436	C	T	NM_000405	c.388C>T:p.R130C	missense	0	-	0	0.428:T	1	0	0	0	-
GM2A	5:150646888	T	C	NM_000405	c.458T>C:p.V153A	missense	0.0159	0.0173	0.0149	0.021:T	82	74	87	43	B
GM2A	5:150646902	G	A	NM_000405	c.472G>A:p.E158K	missense	-	-	-	0.344:T	0	0	1	0	-
GM2A	5:150646926	G	A	NM_000405	c.496G>A:p.G166R	missense	0	-	0	0.925:D	0	0	1	0	-
GM2A	5:150646936	G	A	NM_000405	c.506G>A:p.R169H	missense	0	0	0	0.606:T	0	1	0	0	p.R169P
GM2A	5:150646947	G	A	NM_000405	c.517G>A:p.V173I	missense	0.0005	0.0006	0.0003	0.209:T	0	1	0	0	US
GM2A	5:150646981	T	A	NM_000405	c.551T>A:p.I184N	missense	-	-	0.0001	0.567:T	1	0	0	0	-
GM2A	5:150646981	T	C	NM_000405	c.551T>C:p.I184T	missense	-	-	-	0.547:T	0	0	0	1	-
GM2A	5:150647106	C	A	NM_001167607	c.466C>A:p.P156T	missense	-	-	-	-	1	0	0	0	-
GM2A	5:150647112	A	G	NM_001167607	c.472A>G:p.N158D	missense	0.0003	0.0006	0.0004	-	1	0	0	0	-
GM2A	5:150647187	G	A	NM_001167607	c.547G>A:p.A183T	missense	-	-	-	-	0	1	0	1	-
DTNBP1	6:15523242	CTCT	-	NM_001271669	c.912_915del:p.E305Pfs*42	frameshift deletion	5.80E-05	0	0	-	1	0	0	0	-
DTNBP1	6:15523259	A	C	NM_001271669	c.898T>G:p.S300A	missense	-	-	-	0.009:T	1	0	0	0	-
DTNBP1	6:15523277	C	A	NM_001271669	c.880G>T:p.D294Y	missense	0.0003	-	0.0002	0.819:D	0	4	0	0	-
DTNBP1	6:15523296	A	C	NM_001271669	c.861T>G:p.D287E	missense	0	-	0	0.362:T	0	1	0	1	-
DTNBP1	6:15523316	A	G	NM_001271669	c.841T>C:p.S281P	missense	0.0015	0.0006	0.0012	0.350:T	9	9	8	10	-
DTNBP1	6:15523339	C	T	NM_001271669	c.818G>A:p.R273Q	missense	0	-	0	0.179:T	0	1	0	0	-
DTNBP1	6:15523352	C	T	NM_001271669	c.805G>A:p.D269N	missense	0	0	0	0.270:T	0	0	1	0	-
DTNBP1	6:15523373	A	G	NM_001271669	c.784T>C:p.S262P	missense	0	-	-	0.325:T	1	0	0	0	-
DTNBP1	6:15523426	T	C	NM_001271669	c.731A>G:p.N244S	missense	-	-	-	0.085:T	3	2	2	0	-
DTNBP1	6:15523431	-	C	NM_001271669	c.725dupG:p.C242Wfs*3	frameshift insertion	0.0005	-	0.0006	-	1	0	0	0	-
DTNBP1	6:15524672	G	A	NM_183040	c.896C>T:p.P299L	missense	-	-	-	0.042:T	0	0	1	0	-

DTNBP1	6:15524679	G	A	NM_183040	c.889C>T:p.H297Y	missense	0	0	0.0001	0.045:T	0	1	0	0	B
DTNBP1	6:15524687	C	T	NM_183040	c.881G>A:p.R294H	missense	0	0	0	0.044:T	0	1	0	0	-
DTNBP1	6:15524727	G	A	NM_183040	c.841C>T:p.P281S	missense	0.0002	0	0.0001	0.149:T	2	1	2	0	-
DTNBP1	6:15524748	C	T	NM_183040	c.820G>A:p.D274N	missense	-	-	-	0.050:T	0	0	0	1	-
DTNBP1	6:15524763	C	T	NM_001271669	c.700G>A:p.A234T	missense	0.0001	0.0006	0	0.099:T	0	1	0	0	-
DTNBP1	6:15524768	G	T	NM_001271669	c.695C>A:p.S232Y	missense	0.0001	-	0.0002	0.541:T	0	1	0	0	-
DTNBP1	6:15524786	T	C	NM_001271669	c.677A>G:p.E226G	missense	0.0002	-	0.0001	0.613:T	1	0	1	0	-
DTNBP1	6:15524808	C	T	NM_001271669	c.655G>A:p.V219I	missense	0.0001	-	0.0001	0.425:T	0	0	0	1	-
DTNBP1	6:15533469	G	A	NM_001271669	c.562+2C>T	splicing	0	0	0	0.382:T	1	0	0	1	B
DTNBP1	6:15533473	C	T	NM_001271669	c.560G>A:p.R187Q	missense	0	0	0	0.499:T	0	0	1	0	-
DTNBP1	6:15533555	G	A	NM_001271669	c.478C>T:p.R160W	missense	0	-	0	0.777:D	0	1	0	0	-
DTNBP1	6:15615540	T	C	NM_001271669	c.341A>G:p.H114R	missense	5.80E-05	-	-	0.164:T	0	1	1	0	-
DTNBP1	6:15615596	G	T	NM_001271669	c.285C>A:p.N95K	missense	-	-	-	0.062:T	0	0	0	1	-
DTNBP1	6:15615603	A	G	NM_001271669	c.278T>C:p.V93A	missense	-	-	0	0.532:T	0	0	1	0	-
DTNBP1	6:15627582	G	C	NM_001271669	c.242C>G:p.A81G	missense	0.0005	-	0.0002	0.248:T	4	1	2	0	-
DTNBP1	6:15627600	T	C	NM_001271669	c.224A>G:p.D75G	missense	-	-	0.0001	0.624:T	1	0	0	0	-
DTNBP1	6:15627655	T	A	NM_001271669	c.169A>T:p.T57S	missense	-	-	-	0.029:T	1	0	0	0	-
DTNBP1	6:15627664	-	TGTG	NM_001271669	c.159_160insCACA:p.K54Hfs*38	frameshift insertion	-	-	-	-	0	0	0	1	-
DTNBP1	6:15627670	A	C	NM_001271669	c.154T>G:p.W52G	missense	-	-	-	0.885:D	0	0	0	1	-
DTNBP1	6:15627695	G	C	NM_001271669	c.129C>G:p.S43R	missense	-	-	-	0.592:T	0	1	1	1	-
DTNBP1	6:15638000	G	A	NM_001271669	c.92C>T:p.A31V	missense	-	-	-	0.489:T	0	1	0	0	-
DTNBP1	6:15638035	C	G	NM_183040	c.162G>C:p.R54S;-	missense	0.0012	-	0.0015	0.778:D	1	2	2	1	-
DTNBP1	6:15638037	T	A	NM_001271669	c.57-2A>T	splicing	0	-	0	0.575:T	0	0	1	0	-
DTNBP1	6:15652353	G	T	NM_183040	c.75C>A:p.D25E	missense	5.8E-05	-	-	0.370:T	0	0	2	0	-
DTNBP1	6:15652355	C	A	NM_183040	c.73G>T:p.D25Y	missense	5.8E-05	-	-	0.620:T	0	0	2	0	-
DTNBP1	6:15663094	C	G	NM_001271669	c.7G>C:p.E3Q	missense	-	-	-	0.178:T	0	0	1	0	-
SLC17A5	6:74304824	A	T	NM_012434	c.1464T>A:p.N488K	missense	-	-	-	0.011:T	0	0	1	0	-
SLC17A5	6:74304927	C	T	NM_012434	c.1361G>A:p.G454E	missense	-	-	-	0.142:T	0	1	0	0	-
SLC17A5	6:74310083	C	-	NM_012434	c.1341delG:p.T448Pfs*53	frameshift deletion	5.80E-05	-	0.0001	-	0	1	0	0	-

SLC17A5	6:74310100	C	T	NM_012434	c.1324G>A:p.V442I	missense	0.0068	0.0062	0.0074	0.067:T	44	29	38	18	CIP
SLC17A5	6:74310108	A	G	NM_012434	c.1316T>C:p.V439A	missense	0	0	0	0.188:T	0	0	1	0	-
SLC17A5	6:74320156	C	G	NM_012434	c.1226G>C:p.G409A	missense	-	-	-	0.954:D	0	0	1	0	-
SLC17A5	6:74320189	A	T	NM_012434	c.1193T>A:p.I398K	missense	-	-	-	0.776:D	0	0	1	0	-
SLC17A5	6:74320204	A	G	NM_012434	c.1178T>C:p.V393A	missense	-	-	-	0.724:D	0	2	0	0	-
SLC17A5	6:74320205	C	T	NM_012434	c.1177G>A:p.V393I	missense	5.80E-05	0	0	0.197:T	0	2	0	0	US
SLC17A5	6:74331628	C	A	NM_012434	c.877G>T:p.A293S	missense	-	-	-	0.505:T	1	0	0	0	-
SLC17A5	6:74331634	G	A	NM_012434	c.871C>T:p.L291F	missense	-	-	-	0.715:D	0	0	1	0	-
SLC17A5	6:74331663	G	A	NM_012434	c.842C>T:p.P281L	missense	0	0	-	0.947:D	0	0	0	1	-
SLC17A5	6:74345149	A	G	NM_012434	c.775T>C:p.S259P	missense	5.80E-05	0.0006	-	0.583:T	1	0	1	0	-
SLC17A5	6:74348162	T	C	NM_012434	c.586A>G:p.S196G	missense	-	-	-	0.890:D	0	0	1	0	-
SLC17A5	6:74348164	C	G	NM_012434	c.584G>C:p.R195T	missense	0	-	0	0.969:D	1	0	0	0	-
SLC17A5	6:74348185	G	T	NM_012434	c.563C>A:p.S188Y	missense	-	-	-	0.566:T	0	0	1	0	-
SLC17A5	6:74348216	T	G	NM_012434	c.532A>C:p.T178P	missense	5.8E-05	-	0.0001	0.909:D	0	0	0	1	-
SLC17A5	6:74351530	T	-	NM_012434	c.409delA:p.M137Cfs*2	frameshift deletion	-	-	-	-	1	0	0	0	P/LP
SLC17A5	6:74351532	T	C	NM_012434	c.407A>G:p.K136R	missense	0	-	-	0.700:D	1	0	0	0	p.K136E
SLC17A5	6:74351566	G	A	NM_012434	c.373C>T:p.P125S	missense	0.0002	0.0006	0.0005	0.938:D	6	7	2	4	-
SLC17A5	6:74351602	C	T	NM_012434	c.337G>A:p.G113S	missense	0	-	0	0.817:D	0	1	0	0	-
SLC17A5	6:74354137	T	C	NM_012434	c.284A>G:p.N95S	missense	-	-	-	0.086:T	1	0	0	0	-
SLC17A5	6:74354308	G	A	NM_012434	c.113C>T:p.A38V	missense	-	-	-	0.386:T	0	1	0	0	-
SLC17A5	6:74363539	G	A	NM_012434	c.71C>T:p.P24L	missense	6.3E-05	-	-	0.237:T	0	0	0	2	-
SLC17A5	6:74363573	C	A	NM_012434	c.37G>T:p.G13C	missense	-	-	-	0.487:T	1	0	0	0	-
SLC17A5	6:74363581	C	T	NM_012434	c.29G>A:p.R10Q	missense	-	-	-	0.136:T	1	1	0	0	-
SLC17A5	6:74363582	G	A	NM_012434	c.28C>T:p.R10W	missense	-	-	-	0.336:T	0	0	1	0	-
GUSB	7:65425921	G	C	NM_000181	c.1919C>G:p.A640G	missense	-	-	-	0.365:T	0	0	1	0	-
GUSB	7:65425948	T	C	NM_000181	c.1892A>G:p.N631S	missense	0	0	0	0.559:T	0	0	1	0	-
GUSB	7:65425990	C	A	NM_000181	c.1850G>T:p.S617I	missense	5.80E-05	-	0.0001	0.436:T	2	1	1	1	-
GUSB	7:65432891	G	A	NM_000181	c.1480C>T:p.P494S	missense	-	-	-	0.887:D	0	0	1	1	-
GUSB	7:65435349	C	T	NM_000181	c.1396G>A:p.V466M	missense	-	-	-	0.527:T	0	0	1	0	-
GUSB	7:65439369	C	T	NM_000181	c.1304G>A:p.R435H	missense	0	-	0	0.804:D	0	0	0	1	-
GUSB	7:65439379	C	T	NM_000181	c.1294G>A:p.E432K	missense	-	-	-	0.917:D	0	0	1	0	-

GUSB	7:65439406	G	C	NM_000181	c.1267C>G:p.L423V	missense	-	-	-	0.659:T	1	0	0	0	-
GUSB	7:65439412	C	T	NM_000181	c.1261G>A:p.V421I	missense	0	0	0	0.225:T	1	0	1	0	-
GUSB	7:65439544	C	T	NM_000181	c.1213G>A:p.D405N	missense	0.0016	0.0006	0.001	0.721:D	1	11	5	2	-
GUSB	7:65439636	C	T	NM_000181	c.1121G>A:p.R374H	missense	0	-	0	0.571:T	0	0	1	0	-
GUSB	7:65439666	G	A	NM_000181	c.1091C>T:p.P364L	missense	0	-	0	0.897:D	1	0	0	0	US
GUSB	7:65439685	C	T	NM_000181	c.1072G>A:p.G358R	missense	-	-	-	0.968:D	0	1	0	0	-
GUSB	7:65439929	C	T	NM_000181	c.1042G>A:p.V348I	missense	-	-	-	0.789:D	0	1	0	0	-
GUSB	7:65440055	G	C	NM_000181	c.916C>G:p.Q306E	missense	5.80E-05	-	-	0.390:T	1	0	1	0	-
GUSB	7:65441034	G	C	NM_000181	c.880C>G:p.H294D	missense	-	-	-	0.932:D	1	0	0	0	-
GUSB	7:65441100	T	C	NM_000181	c.814A>G:p.N272D	missense	-	-	-	0.267:T	0	0	1	0	-
GUSB	7:65441110	TT	-	NM_000181	c.803_804del:p.K268Sfs *11	frameshift deletion	-	-	-	-	1	0	0	0	-
GUSB	7:65441133	G	A	NM_000181	c.781C>T:p.R261C	missense	0.0001	-	0.0002	0.401:T	1	1	1	1	-
GUSB	7:65444452	G	C	NM_000181	c.658C>G:p.L220V	missense	0	0	0	0.880:D	1	0	0	0	US
GUSB	7:65444796	G	A	NM_000181	c.499C>T:p.R167X	stopgain	0	-	0	0.720:D	1	0	0	0	-
GUSB	7:65444805	A	G	NM_000181	c.490T>C:p.S164P	missense	-	-	-	0.389:T	3	0	2	0	-
GUSB	7:65445261	G	A	NM_000181	c.346C>T:p.R116C	missense	0	-	0	0.393:T	0	0	1	0	-
GUSB	7:65445277	T	C	NM_001293105	c.1A>G:p.M1V	missense	-	-	-	-	0	1	0	0	-
GUSB	7:65445279	G	A	NM_000181	c.328C>T:p.R110X	stopgain	-	-	-	0.651:T	1	0	0	0	P
GUSB	7:65445314	C	G	NM_000181	c.293G>C:p.W98S	missense	-	-	-	0.968:D	0	0	0	1	-
GUSB	7:65445356	T	C	NM_000181	c.251A>G:p.N84S	missense	0	-	0	0.436:T	0	1	0	1	-
GUSB	7:65445398	-	CCCACAGCGGCCGCCGGTACCACTGCTCCTCGAAGCCCCGGCGTCG	NM_000181	c.211-2->CGACGCCGGGGCTTCGAGGAGCAGTGGTACCGGCGGCCGCTGTGGG	splicing	-	-	-	-	0	0	0	1	-

GUSB	7:65446986	T	C	NM_000181	c.185A>G:p.Q62R	missense	0.0001	-	0.0004	0.288:T	4	1	1	0	US
GUSB	7:65447004	C	T	NM_000181	c.167G>A:p.R56H	missense	0	-	-	0.264:T	0	0	1	0	-
GUSB	7:65447010	T	C	NM_000181	c.161A>G:p.N54S	missense	0.0007	-	0.0011	0.262:T	3	1	1	1	-
GUSB	7:65447012	G	C	NM_000181	c.159C>G:p.D53E	missense	-	-	-	0.148:T	1	0	0	0	-
GUSB	7:65447060	C	A	NM_000181	c.111G>T:p.E37D	missense	5.98E-05	-	0.0001	0.768:D	0	1	0	0	-
GUSB	7:65447084	G	T	NM_000181	c.87C>A:p.Y29X	stopgain	-	-	-	0.454:T	1	0	1	0	-
GUSB	7:65447125	A	C	NM_000181	c.46T>G:p.L16V	missense	-	-	-	0.438:T	0	1	0	1	-
GUSB	7:65447154	G	T	NM_000181	c.17C>A:p.A6E	missense	0.0005	-	-	0.738:D	1	2	2	0	-
GUSB	7:65447158	A	G	NM_000181	c.13T>C:p.S5P	missense	0.0041	0.0043	0.0101	0.223:T	15	13	19	6	CIP
GUSB	7:65447163	C	G	NM_000181	c.8G>C:p.R3P	missense	0	-	-	0.185:T	1	0	0	0	-
KCTD7	7:66094073	G	C	NM_153033	c.22G>C:p.E8Q	missense	0	0.0006	-	0.299:T	1	1	0	0	-
KCTD7	7:66094076	C	G	NM_153033	c.25C>G:p.P9A	missense	-	-	-	0.211:T	1	0	0	0	-
KCTD7	7:66094158	C	T	NM_153033	c.107C>T:p.T36M	missense	0.0011	0.0012	0.0021	0.341:T	1	1	1	2	US
KCTD7	7:66098374	A	G	NM_153033	c.257A>G:p.Y86C	missense	0	-	0	0.729:D	1	0	3	2	US
KCTD7	7:66098419	GC	-	NM_153033	c.302_303del:p.G101Dfs*101	frameshift deletion	-	-	-	-	1	0	0	0	-
KCTD7	7:66103286	C	T	NM_153033	c.361C>T:p.R121C	missense	0	0	0	0.460:T	1	0	0	0	US
KCTD7	7:66103345	G	C	NM_153033	c.420G>C:p.Q140H	missense	-	-	-	0.577:T	1	0	0	0	-
KCTD7	7:66103355	A	G	NM_153033	c.430A>G:p.M144V	missense	0.0003	-	0.0001	0.576:T	0	1	1	0	US
KCTD7	7:66103855	G	A	NM_153033	c.506G>A:p.R169Q	missense	0	-	0	0.566:T	2	0	0	0	US
KCTD7	7:66103879	G	A	NM_153033	c.530G>A:p.R177H	missense	0	0	-	0.575:T	0	1	0	0	-
KCTD7	7:66103899	C	T	NM_153033	c.550C>T:p.R184C	missense	5.80E-05	-	0	0.780:D	1	1	0	0	P/LP
KCTD7	7:66103900	G	A	NM_153033	c.551G>A:p.R184H	missense	5.80E-05	-	0.0001	0.717:D	0	1	0	0	LP
KCTD7	7:66103944	A	G	NM_153033	c.595A>G:p.I199V	missense	0	-	-	0.332:T	1	1	2	0	-
KCTD7	7:66103978	T	C	NM_153033	c.629T>C:p.L210P	missense	-	-	-	0.942:D	0	1	0	0	-
KCTD7	7:66104099	G	-	NM_153033	c.750delG:p.D251Tfs*21	frameshift deletion	-	-	-	-	1	0	0	0	-
KCTD7	7:66104184	C	T	NM_153033	c.835C>T:p.R279C	missense	-	-	-	0.885:D	1	0	0	0	-
CLN8	8:1719097	G	C	NM_018941	UTR5	splicing	-	-	-	-	0	0	1	0	US
CLN8	8:1719098	A	G	NM_018941	UTR5	splicing	-	-	-	-	1	0	0	0	-
CLN8	8:1719236	G	T	NM_018941	c.16G>T:p.D6Y	missense	0.0003	-	0.0005	0.403:T	1	0	3	0	US
CLN8	8:1719285	G	A	NM_018941	c.65G>A:p.G22E	missense	5.80E-05	-	0.0001	0.250:T	1	0	0	0	-

CLN8	8:1719314	T	C	NM_018941	c.94T>C:p.F32L	missense	0.0002	0.0006	0.0001	0.854:D	1	2	1	1	US
CLN8	8:1719317	G	A	NM_018941	c.97G>A:p.V33I	missense	-	-	-	0.155:T	0	1	0	0	-
CLN8	8:1719332	G	A	NM_018941	c.112G>A:p.V38I	missense	0	0.0006	0	0.372:T	1	0	0	0	US
CLN8	8:1719380	C	T	NM_018941	c.160C>T:p.R54C	missense	0.0002	0.0006	0.0002	0.544:T	0	0	1	1	US
CLN8	8:1719390	T	C	NM_018941	c.170T>C:p.V57A	missense	-	-	-	0.194:T	1	0	1	0	-
CLN8	8:1719393	C	A	NM_018941	c.173C>A:p.A58D	missense	5.8E-05	-	-	0.676:T	0	0	1	0	-
CLN8	8:1719426	C	T	NM_018941	c.206C>T:p.T69M	missense	0	0	0	0.931:D	1	0	0	0	US
CLN8	8:1719500	G	A	NM_018941	c.280G>A:p.D94N	missense	0.0003	0.0012	0.0007	0.413:T	1	1	0	0	US
CLN8	8:1719510	G	A	NM_018941	c.290G>A:p.R97H	missense	0.0008	0.0019	0.0005	0.269:T	5	6	7	3	B/LB
CLN8	8:1719567	T	G	NM_018941	c.347T>G:p.F116C	missense	-	-	-	0.898:D	0	0	1	0	US
CLN8	8:1719605	C	T	NM_018941	c.385C>T:p.R129W	missense	0	0	0	0.380:T	2	0	0	0	US
CLN8	8:1719605	C	G	NM_018941	c.385C>G:p.R129G	missense	-	-	-	0.349:T	0	0	1	0	-
CLN8	8:1719621	T	A	NM_018941	c.401T>A:p.F134Y	missense	-	-	-	0.554:T	1	0	0	0	-
CLN8	8:1719675	A	G	NM_018941	c.455A>G:p.N152S	missense	0.0002	0	0.0002	0.353:T	0	0	1	0	US
CLN8	8:1728417	C	T	NM_018941	c.545C>T:p.A182V	missense	0	-	0	0.700:D	0	0	0	1	US
CLN8	8:1728509	T	G	NM_018941	c.637T>G:p.W213G	missense	-	-	-	0.975:D	0	1	0	0	-
CLN8	8:1728534	G	A	NM_018941	c.662G>A:p.G221D	missense	5.80E-05	-	0.0001	0.321:T	1	0	0	0	-
CLN8	8:1728557	C	G	NM_018941	c.685C>G:p.P229A	missense	5.8E-05	0	0.0001	0.600:T	0	0	1	0	-
CLN8	8:1728597	C	T	NM_018941	c.725C>T:p.T242M	missense	0.0049	0.0049	0.0052	0.915:D	19	12	18	7	CIP
CLN8	8:1728668	G	A	NM_018941	c.796G>A:p.A266T	missense	0.0008	0.0006	0.0007	0.307:T	1	1	2	2	US
CLN8	8:1728673	G	C	NM_018941	c.801G>C:p.Q267H	missense	5.80E-05	-	0.0001	0.271:T	1	0	1	0	-
CLN8	8:1728699	G	T	NM_018941	c.827G>T:p.G276V	missense	-	-	-	0.328:T	1	0	0	0	-
ASAH1	8:17915075	G	A	NM_004315	c.1204C>T:p.R402W	missense	0	-	0	0.967:D	1	0	0	0	US
ASAH1	8:17916355	C	T	NM_004315	c.1135G>A:p.V379I	missense	5.80E-05	-	-	0.361:T	1	1	0	0	-
ASAH1	8:17916363	G	C	NM_004315	c.1127C>G:p.T376R	missense	-	-	-	0.978:D	0	0	1	0	-
ASAH1	8:17916382	T	C	NM_004315	c.1108A>G:p.M370V	missense	0	-	0	0.293:T	0	0	0	1	-
ASAH1	8:17916848	A	G	NM_004315	c.1089+2T>C	splicing	-	-	-	0.643:T	1	0	0	0	-
ASAH1	8:17916860	G	A	NM_004315	c.1079C>T:p.T360I	missense	0.0002	-	0.0005	0.521:T	0	0	1	0	-
ASAH1	8:17916887	G	A	NM_004315	c.1052C>T:p.T351M	missense	0	0	0	0.571:T	1	0	0	0	CIP
ASAH1	8:17916897	C	T	NM_004315	c.1042G>A:p.D348N	missense	0	-	-	0.316:T	0	1	0	1	US
ASAH1	8:17916923	C	T	NM_004315	c.1016G>A:p.R339H	missense	0	0	0	0.385:T	0	1	0	0	-
ASAH1	8:17917088	C	G	NM_004315	c.958G>C:p.V320L	missense	0.0056	0.0062	0.0058	0.418:T	17	22	16	5	LB

ASAH1	8:17917180	G	C	NM_004315	c.866C>G:p.T289S	missense	-	-	-	0.593:T	0	0	1	0	-
ASAH1	8:17917192	A	G	NM_004315	c.854T>C:p.L285S	missense	5.80E-05	-	-	0.382:T	1	0	1	0	-
ASAH1	8:17917197	C	G	NM_004315	c.849G>C:p.K283N	missense	-	-	-	0.629:T	1	0	0	0	-
ASAH1	8:17918890	T	A	NM_004315	c.829A>T:p.T277S	missense	-	-	-	0.516:T	1	0	0	0	-
ASAH1	8:17918892	C	T	NM_004315	c.827G>A:p.S276N	missense	0.0002	-	0.0002	0.197:T	1	0	0	0	-
ASAH1	8:17919224	T	C	NM_004315	c.722A>G:p.E241G	missense	-	-	-	0.981:D	0	0	0	1	-
ASAH1	8:17919790	G	C	NM_004315	c.694C>G:p.P232A	missense	-	-	-	0.771:D	1	0	0	0	-
ASAH1	8:17919879	G	A	NM_004315	c.605C>T:p.T202I	missense	-	-	-	0.553:T	0	0	1	0	-
ASAH1	8:17919886	G	T	NM_004315	c.598C>A:p.P200T	missense	-	-	-	0.962:D	1	0	0	0	-
ASAH1	8:17920721	C	G	NM_004315	c.524G>C:p.R175T	missense	-	-	-	0.992:D	1	0	0	0	-
ASAH1	8:17920728	G	A	NM_004315	c.517C>T:p.H173Y	missense	0.0001	0.0006	0.0002	0.984:D	0	2	0	0	-
ASAH1	8:17921967	TTTT	-	NM_004315	c.501_504del:p.K168Vfs*2	frameshift deletion	-	-	-	-	0	1	0	0	-
ASAH1	8:17921971	T	G	NM_004315	c.500A>C:p.K167T	missense	-	-	-	0.236:T	0	1	0	1	-
ASAH1	8:17924739	A	T	NM_004315	c.420T>A:p.D140E	missense	0.0007	0	0.0009	0.100:T	3	5	3	3	B
ASAH1	8:17924764	A	G	NM_004315	c.395T>C:p.M132T	missense	-	-	-	0.702:D	0	0	1	0	-
ASAH1	8:17928855	G	A	NM_004315	c.218C>T:p.P73L	missense	0.0019	0.0031	0.0019	0.793:D	19	13	9	3	-
ASAH1	8:17930786	C	T	NM_001127505	c.174-1G>A	splicing	-	-	-	0.073:T	0	1	0	0	-
ASAH1	8:17933068	T	C	NM_004315	c.155A>G:p.Y52C	missense	-	-	-	0.957:D	0	0	1	0	LP
ASAH1	8:17941519	T	C	NM_177924	c.49A>G:p.S17G	missense	-	-	-	0.148:T	0	2	0	0	-
ASAH1	8:17941528	C	G	NM_177924	c.40G>C:p.A14P	missense	-	-	-	0.709:D	0	1	0	0	-
ASAH1	8:17941564	G	A	NM_177924	c.4C>T:p.P2S	missense	0	-	-	0.251:T	1	0	0	0	-
ASAH1	8:17942190	C	T	NM_004315	c.121G>A:p.A41T	missense	5.80E-05	-	0.0001	0.060:T	0	1	0	0	-
ASAH1	8:17942202	A	G	NM_004315	c.109T>C:p.F37L	missense	-	-	-	0.116:T	1	0	0	0	-
ASAH1	8:17942216	A	G	NM_004315	c.95T>C:p.L32P	missense	-	-	-	0.431:T	1	0	0	0	-
ASAH1	8:17942229	C	T	NM_004315	c.82G>A:p.E28K	missense	0.0124	0.0111	0.0116	0.318:T	26	34	27	20	-
ASAH1	8:17942274	C	G	NM_004315	c.37G>C:p.G13R	missense	-	-	-	0.376:T	1	0	0	0	-
ASAH1	8:17942279	G	C	NM_004315	c.32C>G:p.A11G	missense	5.81E-05	-	0.0002	0.093:T	1	0	0	0	-
ASAH1	8:17942285	T	C	NM_004315	c.26A>G:p.E9G	missense	0.0001	-	-	0.186:T	0	0	1	0	-
ASAH1	8:17942295	C	G	NM_004315	c.16G>C:p.G6R	missense	-	-	-	0.038:T	0	0	0	1	-
ASAH1	8:17942298	T	C	NM_004315	c.13A>G:p.I5V	missense	0	-	0	0.028:T	0	1	1	0	-
ASAH1	8:17942309	A	G	NM_004315	c.2T>C:p.M1T	missense	0	0	0	0.286:T	0	1	0	0	-

HGSNAT	8:42995656	G	A	NM_152419	c.17G>A:p.R6K	missense	-	0.0062	-	0.321:T	0	1	5	4	CIP
HGSNAT	8:42995712	C	G	NM_152419	c.73C>G:p.P25A	missense	-	-	-	0.208:T	0	0	1	0	-
HGSNAT	8:43002147	C	G	NM_152419	c.175C>G:p.H59D	missense	5.86E-05	-	0.0001	0.806:D	1	0	0	0	-
HGSNAT	8:43002177	G	A	NM_152419	c.205G>A:p.V69I	missense	0.0067	0.0043	0.0066	0.230:T	36	35	37	16	CIP
HGSNAT	8:43013742	G	A	NM_152419	c.259G>A:p.V87I	missense	0.0003	-	0.0003	0.132:T	0	1	8	0	US
HGSNAT	8:43013779	C	T	NM_152419	c.296C>T:p.A99V	missense	0.0002	-	0.0003	0.335:T	0	0	0	1	-
HGSNAT	8:43014150	C	G	NM_152419	c.456C>G:p.D152E	missense	-	-	-	0.553:T	1	0	0	0	-
HGSNAT	8:43014155	C	T	NM_152419	c.461C>T:p.A154V	missense	-	-	-	0.198:T	0	1	0	1	-
HGSNAT	8:43014169	C	T	NM_152419	c.475C>T:p.P159S	missense	0.0012	0.0043	0.0006	0.834:D	5	8	2	2	-
HGSNAT	8:43014173	T	C	NM_152419	c.479T>C:p.V160A	missense	0.0033	0.0043	0.003	0.446:T	13	5	6	5	LB
HGSNAT	8:43014188	G	A	NM_152419	c.493+1G>A	splicing	0.0002	0.0006	0	0.631:T	1	1	1	1	P
HGSNAT	8:43025756	C	G	NM_152419	c.662C>G:p.P221R	missense	-	-	-	0.251:T	0	1	0	0	-
HGSNAT	8:43025809	C	G	NM_152419	c.715C>G:p.R239G	missense	-	-	-	0.973:D	0	0	0	1	-
HGSNAT	8:43025810	G	A	NM_152419	c.716G>A:p.R239H	missense	6.1E-05	-	-	0.961:D	0	0	1	0	-
HGSNAT	8:43025815	C	T	NM_152419	c.721C>T:p.R241C	missense	0	0	0	0.939:D	0	1	0	0	-
HGSNAT	8:43025837	G	A	NM_152419	c.743G>A:p.G248E	missense	-	-	-	0.992:D	0	0	1	0	-
HGSNAT	8:43027491	G	A	NM_152419	c.782G>A:p.G261E	missense	-	-	-	0.981:D	1	0	0	0	-
HGSNAT	8:43033252	C	T	NM_152419	c.887C>T:p.S296L	missense	0	-	0	0.989:D	1	0	0	0	US
HGSNAT	8:43033273	G	A	NM_152419	c.908G>A:p.R303Q	missense	0.0008	0.0006	0.0009	0.525:T	1	1	2	2	-
HGSNAT	8:43037305	C	T	NM_152419	c.1030C>T:p.R344C	missense	0	-	0	0.838:D	2	2	0	0	P/LP
HGSNAT	8:43037323	C	T	NM_152419	c.1048C>T:p.Q350X	stopgain	5.8E-05	-	0.0001	0.457:T	0	0	0	1	P
HGSNAT	8:43037374	G	A	NM_152419	c.1099G>A:p.A367T	missense	0	-	-	0.173:T	0	0	1	0	US
HGSNAT	8:43037387	C	T	NM_152419	c.1112C>T:p.P371L	missense	-	-	-	0.222:T	1	0	0	2	US
HGSNAT	8:43046660	C	T	NM_152419	c.1172C>T:p.P391L	missense	-	-	-	0.424:T	1	0	0	0	US
HGSNAT	8:43047460	C	T	NM_152419	c.1264C>T:p.P422S	missense	0	-	0	0.435:T	0	0	1	0	US
HGSNAT	8:43047508	G	A	NM_152419	c.1312G>A:p.A438T	missense	0.0009	-	0.0008	0.673:T	3	0	1	1	-
HGSNAT	8:43047569	C	G	NM_152419	c.1373C>G:p.S458C	missense	-	-	-	0.481:T	1	1	0	1	-
HGSNAT	8:43048933	G	A	NM_152419	c.1411G>A:p.E471K	missense	0	-	0	0.805:D	1	0	0	0	-
HGSNAT	8:43048936	G	A	NM_152419	c.1414G>A:p.G472S	missense	5.80E-05	0.0006	-	0.881:D	0	1	0	1	-
HGSNAT	8:43051585	T	-	NM_001363227	c.1477delT:p.W493Gfs*7	frameshift deletion	-	-	-	-	1	0	0	0	-
HGSNAT	8:43051610	C	T	NM_001363227	c.1502C>T:p.T501M	missense	0.0006	0.0006	-	-	3	2	0	0	-

HGSNAT	8:43052118	G	C	NM_152419	c.1492G>C:p.A498P	missense	0.0002	0.0006	-	0.404:T	0	2	1	1	US
HGSNAT	8:43052122	G	A	NM_152419	c.1496G>A:p.R499Q	missense	0.0001	0	0	0.383:T	0	1	0	0	-
HGSNAT	8:43052135	C	G	NM_152419	c.1509C>G:p.I503M	missense	0.0002	-	0.0002	0.236:T	0	1	0	0	-
HGSNAT	8:43052164	T	C	NM_152419	c.1538T>C:p.I513T	missense	-	-	-	0.711:D	0	1	1	0	-
HGSNAT	8:43052814	G	A	NM_152419	c.1543-1G>A	splicing	-	-	-	0.643:T	0	0	1	0	-
HGSNAT	8:43054548	G	A	NM_152419	c.1744G>A:p.V582I	missense	-	-	-	0.698:T	1	0	0	0	-
HGSNAT	8:43054691	G	T	NM_152419	c.1887G>T:p.K629N	missense	-	-	-	0.632:T	1	0	0	0	-
HGSNAT	8:43054697	T	G	NM_152419	c.1893T>G:p.I631M	missense	-	-	-	0.600:T	0	0	0	1	-
GNE	9:36217451	C	T	NM_001128227	c.2173G>A:p.V725M	missense	0	-	-	0.698:T	1	0	0	0	-
GNE	9:36217488	G	T	NM_001128227	c.2136C>A:p.D712E	missense	-	-	-	0.571:T	0	1	0	1	US
GNE	9:36217508	A	G	NM_001128227	c.2116T>C:p.Y706H	missense	0	-	-	0.974:D	0	1	0	0	P
GNE	9:36217513	C	T	NM_001128227	c.2111G>A:p.S704N	missense	5.8E-05	-	-	0.338:T	0	0	0	1	-
GNE	9:36218182	G	T	NM_001128227	c.2024C>A:p.T675K	missense	-	-	-	0.596:T	0	1	0	0	-
GNE	9:36218208	A	C	NM_001128227	c.1998T>G:p.N666K	missense	-	-	-	0.696:T	1	0	0	0	-
GNE	9:36218242	G	A	NM_001128227	c.1964C>T:p.A655V	missense	0	0	0	0.883:D	0	0	1	0	US
GNE	9:36219983	-	AATT	NM_001128227	c.1760_1761insAATT:p.H589Dfs*21	frameshift insertion	-	-	-	-	1	0	1	0	-
GNE	9:36219990	T	C	NM_001128227	c.1754A>G:p.H585R	missense	5.80E-05	-	-	0.682:T	0	1	2	0	-
GNE	9:36220012	C	T	NM_001128227	c.1732G>A:p.G578S	missense	0	-	-	0.971:D	0	0	0	1	-
GNE	9:36222882	G	A	NM_001128227	c.1618C>T:p.H540Y	missense	5.80E-05	-	0.0002	0.757:D	0	1	0	1	LP
GNE	9:36222965	C	T	NM_001128227	c.1535G>A:p.R512Q	missense	0.0001	-	0.0006	0.383:T	0	1	0	0	US
GNE	9:36222974	A	G	NM_001128227	c.1526T>C:p.V509A	missense	-	-	-	0.965:D	0	0	1	0	-
GNE	9:36222989	-	A	NM_001128227	c.1510dupT:p.S504Ffs*28	frameshift insertion	-	-	-	-	0	0	0	1	-
GNE	9:36223432	A	G	NM_001128227	c.1442T>C:p.L481P	missense	-	-	-	0.974:D	0	1	1	0	-
GNE	9:36223492	A	G	NM_001128227	c.1382T>C:p.I461T	missense	-	-	-	0.947:D	1	0	0	0	-
GNE	9:36227267	C	T	NM_001128227	c.1352G>A:p.R451Q	missense	0	-	0	0.946:D	0	1	0	0	US
GNE	9:36227292	C	T	NM_001128227	c.1327G>A:p.V443I	missense	0.0002	-	0	0.557:T	0	0	1	0	CIP
GNE	9:36227397	T	C	NM_001128227	c.1222A>G:p.I408V	missense	5.80E-05	0	0.0001	0.734:D	2	1	0	0	US
GNE	9:36233937	C	T	NM_001128227	c.1055G>A:p.R352H	missense	0	-	-	0.935:D	0	0	1	0	-

GNE	9:36234015	C	T	NM_001128227	c.977G>A:p.G326D	missense	-	-	-	0.946:D	0	0	0	1	-
GNE	9:36234037	T	C	NM_001128227	c.955A>G:p.I319V	missense	5.8E-05	-	-	0.755:D	0	0	1	1	-
GNE	9:36234055	C	T	NM_001128227	c.937G>A:p.V313I	missense	0	-	0	0.408:T	0	0	0	1	-
GNE	9:36236861	C	T	NM_001190383	c.737G>A:p.R246Q	missense	0.0001	-	-	0.797:D	1	0	0	0	P/LP
GNE	9:36246117	T	A	NM_001128227	c.620A>T:p.D207V	missense	0.0006	0.0006	0.0005	0.950:D	7	4	8	3	P/LP
GNE	9:36246159	C	T	NM_001128227	c.578G>A:p.R193H	missense	0	-	0	0.852:D	0	0	1	0	p.R193C
GNE	9:36249220	C	T	NM_001128227	c.226G>A:p.V76I	missense	-	-	-	0.734:D	0	1	0	0	-
GNE	9:36249273	G	A	NM_001128227	c.173C>T:p.P58L	missense	-	0	-	0.966:D	1	0	0	0	LP
GNE	9:36249330	C	T	NM_001128227	c.116G>A:p.R39Q	missense	0	0	0	0.562:T	1	0	0	0	US
GNE	9:36249363	T	C	NM_001128227	c.83A>G:p.N28S	missense	-	-	-	0.275:T	0	1	0	0	-
GNE	9:36276924	A	T	NM_001128227	c.18T>A:p.Y6X	stopgain	0.0024	0.0006	0.0029	0.176:T	8	5	10	4	-
GNE	9:36276927	A	G	NM_001190388	c.113T>C:p.V38A	missense	0.0002	-	0.0001	0.107:T	2	0	1	1	-
GNE	9:36276951	C	T	NM_001190388	c.89G>A:p.R30H	missense	0	0	0	0.011:T	1	0	0	0	B/LB
GNE	9:36276952	G	A	NM_001190388	c.88C>T:p.R30C	missense	5.81E-05	-	0.0001	0.015:T	0	1	0	0	-
PSAP	10:73578842	C	A	NM_002778	c.1377G>T:p.E459D	missense	-	-	-	0.832:D	1	0	0	0	-
PSAP	10:73578844	C	T	NM_002778	c.1375G>A:p.E459K	missense	0	-	-	0.912:D	0	0	1	0	-
PSAP	10:73579250	C	T	NM_002778	c.1322G>A:p.S441N	missense	0.0001	-	0.0001	0.280:T	0	2	1	0	US
PSAP	10:73579275	G	-	NM_002778	c.1297delC:p.L433Wfs*29	frameshift deletion	-	-	-	-	1	0	0	0	-
PSAP	10:73579322	C	T	NM_002778	c.1250G>A:p.G417D	missense	0.0001	-	0.0001	0.427:T	1	0	0	0	-
PSAP	10:73579522	C	T	NM_002778	c.1141G>A:p.V381M	missense	-	-	-	0.848:D	1	1	0	1	-
PSAP	10:73579539	T	A	NM_002778	c.1124A>T:p.E375V	missense	-	0	-	0.607:T	2	0	0	0	-
PSAP	10:73579570	C	T	NM_002778	c.1093G>A:p.G365S	missense	0	-	-	0.844:D	1	0	0	0	-
PSAP	10:73579590	T	A	NM_002778	c.1073A>T:p.Q358L	missense	0.0002	0.0006	0.0001	0.625:T	0	1	0	1	-
PSAP	10:73579599	T	G	NM_002778	c.1064A>C:p.E355A	missense	0.0003	-	0.0005	0.145:T	0	0	1	0	-
PSAP	10:73579602	G	A	NM_002778	c.1061C>T:p.S354L	missense	0	-	0	0.510:T	0	1	0	0	-
PSAP	10:73579626	C	T	NM_002778	c.1037G>A:p.C346Y	missense	-	-	-	0.988:D	1	0	0	0	-
PSAP	10:73579645	C	T	NM_002778	c.1018G>A:p.D340N	missense	0.0001	-	0.0001	0.116:T	1	0	1	0	US
PSAP	10:73580094	-	TAATGGGCTCCACCAGTT	NM_002778	c.910-2->CCGCCAAAGTGGCCTCCAAGAATGTCATCCCTGCCCTGGAACTGGTGGAGC	splicing	-	-	-	-	0	0	0	1	-

			CCAGGGCAGGGATGACATTCTTGGAGGCCACTTTGGCGG		CCATTA										
PSAP	10:73581650	G	C	NM_002778	c.892C>G:p.L298V	missense	-	-	-	0.625:T	0	0	0	1	-
PSAP	10:73581676	G	A	NM_002778	c.866C>T:p.S289F	missense	-	-	-	0.281:T	1	0	0	0	-
PSAP	10:73581685	T	C	NM_002778	c.857A>G:p.K286R	missense	-	-	-	0.088:T	0	0	1	0	-
PSAP	10:73581695	C	G	NM_002778	c.847G>C:p.V283L	missense	0	-	0	0.221:T	0	0	0	1	-
PSAP	10:73581755	C	A	NM_002778	c.787G>T:p.E263X	stopgain	-	-	-	0.686:T	1	0	0	0	-
PSAP	10:73585647	T	A	NM_002778	c.724A>T:p.K242X	stopgain	-	-	-	0.781:D	0	0	1	0	-
PSAP	10:73587811	T	C	NM_002778	c.680A>G:p.K227R	missense	-	-	0.0001	0.245:T	0	0	0	1	-
PSAP	10:73587830	C	T	NM_002778	c.661G>A:p.A221T	missense	0.0003	0.0006	0.0005	0.097:T	4	0	2	0	-
PSAP	10:73587854	G	A	NM_002778	c.637C>T:p.R213W	missense	5.80E-05	0	0	0.741:D	1	1	0	0	-
PSAP	10:73587901	A	G	NM_002778	c.590T>C:p.V197A	missense	-	-	-	0.826:D	0	0	1	0	-
PSAP	10:73587902	C	T	NM_002778	c.589G>A:p.V197I	missense	5.80E-05	0	0	0.454:T	0	1	0	0	US
PSAP	10:73587902	C	A	NM_002778	c.589G>T:p.V197F	missense	0.0002	-	0.0002	0.862:D	1	1	1	0	-
PSAP	10:73587916	-	TTGGCTGGGGCTTGCTGCGGGGGCCGTCCTG	NM_002778	c.577-2->CAGGACGGCCCCCGCAGCAAGCCCCAGCCAA	splicing	-	-	-	-	0	0	0	1	-
PSAP	10:73588645	G	A	NM_002778	c.565C>T:p.P189S	missense	0.001	0.0006	0.0012	0.276:T	8	11	14	3	CIP
PSAP	10:73588653	-	G	NM_002778	c.556dupC:p.R186Pfs*8	frameshift insertion	-	-	-	-	1	0	0	0	-

PSAP	10:73588653	C	T	NM_002778	c.557G>A:p.R186H	missense	5.80E-05	0	0	0.071:T	1	1	0	0	CIP
PSAP	10:73588654	G	A	NM_002778	c.556C>T:p.R186C	missense	0	-	0	0.399:T	0	1	0	0	-
PSAP	10:73588659	C	T	NM_002778	c.551G>A:p.G184D	missense	-	-	-	0.263:T	1	0	0	1	-
PSAP	10:73588699	A	C	NM_002778	c.511T>G:p.F171V	missense	0.0001	-	-	0.882:D	2	1	0	0	-
PSAP	10:73588741	T	G	NM_002778	c.469A>C:p.N157H	missense	-	-	-	0.575:T	0	0	1	0	p.N157S
PSAP	10:73588756	T	C	NM_002778	c.454A>G:p.K152E	missense	-	-	-	0.096:T	0	0	1	0	-
PSAP	10:73588816	A	-	NM_002778	c.394delT:p.C132Afs*13	frameshift deletion	-	-	-	-	1	0	0	0	-
PSAP	10:73590965	G	A	NM_002778	c.293C>T:p.P98L	missense	0	0.0006	0	0.721:D	0	0	1	0	-
PSAP	10:73590993	A	G	NM_002778	c.265T>C:p.Y89H	missense	-	-	-	0.674:T	0	0	1	0	-
PSAP	10:73591626	T	A	NM_002778	c.226A>T:p.M76L	missense	-	-	-	0.260:T	0	1	0	0	-
PSAP	10:73591638	C	G	NM_002778	c.214G>C:p.A72P	missense	-	-	-	0.769:D	0	0	1	1	-
PSAP	10:73591667	G	C	NM_002778	c.185C>G:p.P62R	missense	5.80E-05	-	-	0.820:D	0	1	0	0	-
PSAP	10:73594149	C	T	NM_002778	c.154G>A:p.V52I	missense	0.0001	0	0	0.426:T	0	0	1	0	-
PSAP	10:73610951	G	A	NM_002778	c.28C>T:p.L10F	missense	9.81E-05	0	0	0.198:T	2	2	1	3	US
PSAP	10:73610971	G	C	NM_002778	c.8C>G:p.A3G	missense	0	-	-	0.314:T	0	0	1	0	US
PSAP	10:73610972	C	A	NM_002778	c.7G>T:p.A3S	missense	0	-	-	0.078:T	1	0	0	0	-
LIPA	10:90974614	T	C	NM_000235	c.1171A>G:p.I391V	missense	-	-	-	0.201:T	3	0	1	0	US
LIPA	10:90974627	C	G	NM_000235	c.1158G>C:p.R386S	missense	0.0004	-	0.0005	0.483:T	1	4	0	0	LP
LIPA	10:90974652	A	G	NM_000235	c.1133T>C:p.I378T	missense	5.8E-05	-	0.0001	0.698:T	0	0	3	0	-
LIPA	10:90974653	T	G	NM_000235	c.1132A>C:p.I378L	missense	-	-	-	0.211:T	0	0	1	0	-
LIPA	10:90974682	C	T	NM_000235	c.1103G>A:p.S368N	missense	0	0	-	0.038:T	1	0	0	0	-
LIPA	10:90974761	C	T	NM_000235	c.1024G>A:p.G342R	missense	0	0	0	0.985:D	1	0	0	0	P/LP
LIPA	10:90974776	T	C	NM_000235	c.1009A>G:p.T337A	missense	0.0098	0.0099	0.0094	0.755:D	27	21	24	14	CIP
LIPA	10:90983508	A	G	NM_000235	c.755T>C:p.I252T	missense	-	-	-	0.596:T	1	1	0	0	-
LIPA	10:90983509	T	A	NM_000235	c.754A>T:p.I252L	missense	0	0	0	0.207:T	1	1	0	0	CIP
LIPA	10:90983509	T	C	NM_000235	c.754A>G:p.I252V	missense	-	-	-	0.060:T	1	0	0	0	-
LIPA	10:90984876	T	G	NM_000235	c.648A>C:p.L216F	missense	0.0006	0.0012	0.0007	0.596:T	4	1	2	0	-
LIPA	10:90984905	C	A	NM_000235	c.619G>T:p.A207S	missense	-	-	0.0001	0.180:T	1	2	0	1	-
LIPA	10:90984923	C	T	NM_000235	c.601G>A:p.G201S	missense	-	-	-	0.430:T	0	0	0	1	-
LIPA	10:90986679	C	A	NM_000235	c.511G>T:p.V171L	missense	0.0002	0.0006	0.0003	0.615:T	2	2	4	2	-
LIPA	10:90988005	C	T	NM_000235	c.380G>A:p.R127Q	missense	5.80E-05	0	0	0.619:T	1	0	1	0	US

LIPA	10:90988006	G	A	NM_000235	c.379C>T:p.R127W	missense	0	0	0	0.769:D	0	1	0	1	US
LIPA	10:90988141	C	T	NM_000235	c.244G>A:p.V82I	missense	0	-	0	0.664:T	0	0	1	0	-
LIPA	10:91005437	G	C	NM_000235	c.225C>G:p.D75E	missense	-	-	-	0.197:T	0	0	1	0	-
LIPA	10:91007320	A	G	NM_000235	c.86T>C:p.V29A	missense	-	-	-	0.514:T	0	1	0	0	-
LIPA	10:91007369	C	T	NM_000235	c.37G>A:p.V13I	missense	5.8E-05	-	0	0.104:T	0	0	0	1	-
LIPA	10:91007395	C	T	NM_000235	c.11G>A:p.R4Q	missense	0	0	0	0.138:T	0	1	1	0	US
LIPA	10:91007396	G	A	NM_000235	c.10C>T:p.R4W	missense	5.8E-05	-	-	0.254:T	0	0	1	0	-
HPS1	10:100177337	C	T	NM_000195	c.2087G>A:p.R696H	missense	0	0	0	0.167:T	0	0	0	1	US
HPS1	10:100177338	G	C	NM_000195	c.2086C>G:p.R696G	missense	0.0004	-	0.0005	0.600:T	1	0	0	0	-
HPS1	10:100177359	G	A	NM_000195	c.2065C>T:p.R689W	missense	0.002	0.0025	0.0018	0.638:T	29	20	23	14	-
HPS1	10:100177428	C	T	NM_000195	c.1996G>A:p.E666K	missense	5.80E-05	-	0.0001	0.947:D	0	1	0	0	-
HPS1	10:100177431	A	G	NM_000195	c.1993T>C:p.Y665H	missense	-	-	-	0.418:T	0	1	0	0	-
HPS1	10:100177451	G	A	NM_000195	c.1973C>T:p.P658L	missense	0	0	0	0.193:T	0	1	0	0	-
HPS1	10:100177466	T	C	NM_000195	c.1958A>G:p.Y653C	missense	5.80E-05	-	0.0001	0.621:T	1	0	0	0	-
HPS1	10:100177472	C	T	NM_000195	c.1952G>A:p.R651H	missense	0	-	-	0.684:T	0	0	1	0	-
HPS1	10:100177473	G	A	NM_000195	c.1951C>T:p.R651C	missense	5.80E-05	0	0	0.818:D	1	0	0	0	US
HPS1	10:100177940	G	-	NM_000195	c.1932delC:p.Y645Tfs*79	frameshift deletion	5.83E-05	-	-	-	1	1	2	0	LP
HPS1	10:100177960	T	C	NM_000195	c.1912A>G:p.I638V	missense	0	0	-	0.121:T	0	0	1	0	-
HPS1	10:100177981	G	A	NM_000195	c.1891C>T:p.L631F	missense	-	-	-	0.494:T	1	0	0	0	-
HPS1	10:100177984	C	T	NM_000195	c.1888G>A:p.V630I	missense	0	0	0	0.193:T	0	1	0	0	CIP
HPS1	10:100179893	G	A	NM_000195	c.1766C>T:p.A589V	missense	0.0003	0.0012	0.0006	0.691:T	2	1	0	1	US
HPS1	10:100182151	G	A	NM_000195	c.1718C>T:p.P573L	missense	0	0	0	0.397:T	1	0	1	0	-
HPS1	10:100183549	A	C	NM_000195	c.1493T>G:p.L498R	missense	-	-	-	0.861:D	0	0	1	0	-
HPS1	10:100183595	G	A	NM_000195	c.1447C>T:p.R483W	missense	0	-	0	0.409:T	0	0	1	0	US
HPS1	10:100183604	C	T	NM_000195	c.1438G>A:p.A480T	missense	0.0001	0	0	0.020:T	0	1	0	0	US
HPS1	10:100183615	C	T	NM_000195	c.1427G>A:p.R476Q	missense	0	-	0	0.212:T	1	0	0	0	-
HPS1	10:100185304	C	A	NM_000195	c.1329G>T:p.E443D	missense	0	-	-	0.168:T	1	0	1	0	-
HPS1	10:100185341	C	T	NM_000195	c.1292G>A:p.R431K	missense	-	-	-	0.153:T	0	0	1	1	-
HPS1	10:100185375	G	A	NM_000195	c.1258C>T:p.R420C	missense	0	-	0	0.860:D	1	0	0	0	-
HPS1	10:100185395	G	A	NM_000195	c.1238C>T:p.P413L	missense	5.81E-05	0.0006	0	0.127:T	0	1	0	1	-
HPS1	10:100185437	A	G	NM_000195	c.1196T>C:p.M399T	missense	0.0003	-	0.0003	0.474:T	0	0	1	0	-

HPS1	10:100185468	C	G	NM_000195	c.1165G>C:p.A389P	missense	-	-	-	0.224:T	1	0	0	0	-
HPS1	10:100185477	T	C	NM_000195	c.1156A>G:p.S386G	missense	-	-	-	0.112:T	0	1	0	1	-
HPS1	10:100185668	A	T	NM_000195	c.1062T>A:p.D354E	missense	0.0011	-	0.0005	0.126:T	2	1	3	1	-
HPS1	10:100185706	A	G	NM_000195	c.1024T>C:p.C342R	missense	-	-	-	0.171:T	1	1	0	2	-
HPS1	10:100186986	-	G	NM_000195	c.972dupC:p.M325Hfs*127	frameshift insertion	0.0001	0	0.0015	-	1	0	0	0	-
HPS1	10:100186987	G	-	NM_000195	c.972delC:p.M325Wfs*5	frameshift deletion	0.0003	0	0	-	0	2	0	0	-
HPS1	10:100186997	C	T	NM_000195	c.962G>A:p.G321D	missense	0.0003	-	-	0.210:T	1	1	0	0	-
HPS1	10:100187007	G	C	NM_000195	c.952C>G:p.L318V	missense	0.0118	0.0129	0.0041	0.210:T	30	28	36	23	B/LB
HPS1	10:100187012	A	G	NM_000195	c.947T>C:p.I316T	missense	-	-	-	0.264:T	1	0	0	0	-
HPS1	10:100187015	G	T	NM_000195	c.944C>A:p.T315N	missense	9.6E-05	-	-	0.177:T	0	0	0	1	-
HPS1	10:100189239	C	T	NM_001322490	c.910G>A:p.V304M	missense	-	-	-	-	0	0	0	1	-
HPS1	10:100189266	T	A	NM_001322490	c.883A>T:p.I295F	missense	0.0005	0.0012	0.0006	-	2	6	0	1	-
HPS1	10:100189268	T	C	NM_001322490	c.881A>G:p.D294G	missense	0.0002	-	0.0002	-	1	0	0	0	-
HPS1	10:100189275	C	T	NM_001322490	c.874G>A:p.A292T	missense	0.0106	0.0136	0.0098	-	41	25	29	12	-
HPS1	10:100189284	G	A	NM_001322490	c.865C>T:p.R289X	stopgain	0	0	0	-	1	0	0	0	-
HPS1	10:100189304	T	C	NM_001322490	c.845A>G:p.E282G	missense	0.0001	-	0.0001	-	0	1	0	0	-
HPS1	10:100189320	C	T	NM_001322490	c.829G>A:p.G277R	missense	0.0001	-	0	0.073:T	7	6	6	4	-
HPS1	10:100189321	G	A	NM_001322491	c.847C>T:p.R283W	missense	0	-	0	0.089:T	0	0	1	0	LB
HPS1	10:100189354	G	T	NM_000195	c.913C>A:p.P305T	missense	-	-	-	0.270:T	0	0	1	1	-
HPS1	10:100189355	A	T	NM_001322490	c.794T>A:p.L265H	missense	-	-	-	-	0	0	1	1	-
HPS1	10:100189358	T	C	NM_001322490	c.791A>G:p.Q264R	missense	0.0001	0	0.0002	-	0	0	1	0	-
HPS1	10:100189567	C	G	NM_000195	c.848G>C:p.G283A	missense	0	0	0	0.026:T	0	0	1	0	B/LB
HPS1	10:100189568	C	A	NM_000195	c.847G>T:p.G283W	missense	0.0002	0.0012	0.0001	0.236:T	3	2	0	2	B/LB
HPS1	10:100189568	C	G	NM_000195	c.847G>C:p.G283R	missense	0.0009	0.0018	0.0008	0.028:T	1	1	3	1	LB
HPS1	10:100189568	C	T	NM_000195	c.847G>A:p.G283R	missense	0	0	0	0.035:T	1	0	0	0	LB
HPS1	10:100189597	G	A	NM_000195	c.818C>T:p.A273V	missense	-	-	-	0.148:T	0	0	1	0	-
HPS1	10:100189607	C	T	NM_000195	c.808G>A:p.V270M	missense	0	-	0	0.229:T	1	0	0	0	US
HPS1	10:100189635	C	T	NM_001322490	c.662G>A:p.G221E	missense	-	-	-	-	0	1	0	0	-
HPS1	10:100189636	C	T	NM_000195	c.779G>A:p.R260Q	missense	0.0001	0	0	0.009:T	2	0	0	0	B

HPS1	10:100189637	G	A	NM_000195	c.778C>T:p.R260W	missense	0	0	0	0.118:T	1	0	0	0	-
HPS1	10:100189638	C	T	NM_001322490	c.659G>A:p.R220H	missense	5.82E-05	0	0.0001	-	0	1	0	0	-
HPS1	10:100189639	G	A	NM_000195	c.776C>T:p.P259L	missense	0	-	0	0.109:T	1	0	0	0	US
HPS1	10:100190330	G	C	NM_000195	c.766C>G:p.Q256E	missense	-	-	-	0.213:T	0	0	1	0	-
HPS1	10:100190337	G	A	NM_001322490	c.641C>T:p.T214M	missense	0	0	0	-	1	1	1	1	-
HPS1	10:100190354	C	T	NM_000195	c.742G>A:p.E248K	missense	0.0006	-	0.0012	0.028:T	2	2	4	1	US
HPS1	10:100190373	A	T	NM_001322490	c.605T>A:p.F202Y	missense	-	-	-	-	1	0	0	0	US
HPS1	10:100190378	G	C	NM_000195	c.718C>G:p.L240V	missense	0.0009	-	-	0.169:T	2	0	3	1	-
HPS1	10:100190404	G	A	NM_000195	c.692C>T:p.P231L	missense	0	0	0	0.594:T	0	0	2	0	US
HPS1	10:100190422	C	T	NM_000195	c.674G>A:p.S225N	missense	-	-	-	0.004:T	1	1	0	0	-
HPS1	10:100190950	-	CC	NM_000195	c.605_606insGG:p.G203 Efs*128	frameshif t insertion	-	-	-	-	0	0	1	0	-
HPS1	10:100190955	G	A	NM_000195	c.601C>T:p.R201W	missense	0	-	-	0.533:T	2	0	0	0	-
HPS1	10:100190958	C	T	NM_000195	c.598G>A:p.E200K	missense	0	0	0	0.270:T	1	0	1	0	-
HPS1	10:100190999	G	A	NM_000195	c.557C>T:p.A186V	missense	0.0002	0	0.0002	0.152:T	0	1	0	0	B/LB
HPS1	10:100191016	A	C	NM_000195	c.540T>G:p.C180W	missense	-	-	-	0.911:D	0	0	4	0	-
HPS1	10:100193727	G	A	NM_001322490	c.520C>T:p.R174C	missense	0	0	0	-	0	1	0	0	CIP
HPS1	10:100193732	G	A	NM_001322490	c.515C>T:p.T172I	missense	-	-	-	-	0	0	0	1	-
HPS1	10:100193769	G	A	NM_000195	c.478C>T:p.R160W	missense	0	0	0	0.620:T	0	0	1	0	B/LB
HPS1	10:100193775	G	A	NM_000195	c.472C>T:p.R158C	missense	0	-	-	0.167:T	1	0	0	0	US
HPS1	10:100193840	G	A	NM_000195	c.407C>T:p.P136L	missense	-	-	-	0.886:D	1	0	0	0	-
HPS1	10:100195035	C	T	NM_000195	c.392G>A:p.R131Q	missense	0	-	0.0001	0.257:T	1	1	1	0	p.R131X
HPS1	10:100195054	C	T	NM_000195	c.373G>A:p.V125M	missense	-	-	-	0.097:T	2	2	0	1	-
HPS1	10:100195096	C	T	NM_000195	c.331G>A:p.V111M	missense	-	-	-	0.740:D	1	0	0	0	-
HPS1	10:100195110	C	T	NM_000195	c.317G>A:p.R106Q	missense	0	0	0	0.656:T	0	0	0	1	-
HPS1	10:100195111	G	A	NM_000195	c.316C>T:p.R106W	missense	0	0	0	0.897:D	0	0	0	1	-
HPS1	10:100195119	C	T	NM_000195	c.308G>A:p.G103E	missense	0.0005	-	0.0003	0.231:T	1	0	1	0	-
HPS1	10:100195123	C	T	NM_000195	c.304G>A:p.E102K	missense	5.80E-05	-	0	0.849:D	1	1	1	0	-
HPS1	10:100195405	T	A	NM_000195	c.242A>T:p.Y81F	missense	0.0008	0.0018	0.0009	0.502:T	6	3	1	2	US
HPS1	10:100195406	A	G	NM_000195	c.241T>C:p.Y81H	missense	-	-	-	0.465:T	0	0	1	0	-
HPS1	10:100195460	C	A	NM_000195	c.187G>T:p.E63X	stopgain	-	-	-	0.779:D	1	0	1	0	P

HPS1	10:100195468	G	A	NM_000195	c.179C>T:p.T60M	missense	0	0	0	0.590:T	0	0	1	0	CIP
HPS1	10:100202969	C	T	NM_000195	c.29G>A:p.G10D	missense	5.8E-05	-	-	0.529:T	0	0	1	0	-
HPS1	10:100202987	A	G	NM_000195	c.11T>C:p.V4A	missense	0.0077	0.0105	0.008	0.335:T	33	22	29	25	B/LB
HPS6	10:103825238	C	G	NM_024747	c.7C>G:p.R3G	missense	-	-	-	0.499:T	1	0	0	0	-
HPS6	10:103825280	-	GCGGC	NM_024747	c.49_50insGCGGC:p.L22Rfs*32	frameshift insertion	0	-	0	-	0	0	1	0	-
HPS6	10:103825341	G	A	NM_024747	c.110G>A:p.S37N	missense	-	-	-	0.364:T	1	0	1	0	-
HPS6	10:103825350	G	C	NM_024747	c.119G>C:p.G40A	missense	9.83E-05	-	-	0.489:T	1	0	0	0	-
HPS6	10:103825452	G	C	NM_024747	c.221G>C:p.G74A	missense	0.0001	-	0	0.142:T	0	0	1	0	-
HPS6	10:103825508	G	T	NM_024747	c.277G>T:p.V93L	missense	0.0042	0.0025	0	0.328:T	6	6	6	2	US
HPS6	10:103825578	A	G	NM_024747	c.347A>G:p.Q116R	missense	-	-	-	0.381:T	1	0	0	0	-
HPS6	10:103825588	G	T	NM_024747	c.357G>T:p.E119D	missense	-	-	-	0.434:T	0	0	1	0	-
HPS6	10:103825661	C	T	NM_024747	c.430C>T:p.R144W	missense	9.46E-05	-	-	0.439:T	0	1	0	0	-
HPS6	10:103825670	C	G	NM_024747	c.439C>G:p.R147G	missense	-	-	-	0.111:T	1	0	0	0	-
HPS6	10:103825676	G	A	NM_024747	c.445G>A:p.E149K	missense	-	-	-	0.183:T	1	3	1	1	-
HPS6	10:103825734	TG	-	NM_024747	c.503_504del:p.L168Rfs*6	frameshift deletion	-	-	-	-	0	0	1	0	-
HPS6	10:103825777	-	GTCCTGCTGCACCACT	NM_024747	c.546_547insGTCCTGCTGCACCACT:p.F191Afs*53	frameshift insertion	-	-	-	-	0	0	0	1	-
HPS6	10:103825847	G	A	NM_024747	c.616G>A:p.A206T	missense	-	-	-	0.188:T	0	1	0	0	-
HPS6	10:103825863	G	C	NM_024747	c.632G>C:p.G211A	missense	0.0065	0.0049	0.006	0.370:T	22	19	14	11	B/LB
HPS6	10:103825929	T	G	NM_024747	c.698T>G:p.L233R	missense	0	0	0	0.278:T	0	1	0	0	B/LB
HPS6	10:103825986	G	A	NM_024747	c.755G>A:p.R252Q	missense	-	-	-	0.192:T	1	0	0	0	-
HPS6	10:103826022	C	G	NM_024747	c.791C>G:p.P264R	missense	-	-	-	0.269:T	0	1	0	0	-
HPS6	10:103826123	A	G	NM_024747	c.892A>G:p.T298A	missense	-	-	-	0.101:T	0	0	1	0	-
HPS6	10:103826124	-	G	NM_024747	c.894dupG:p.R299Afs*67	frameshift insertion	-	-	-	-	0	1	0	0	-
HPS6	10:103826129	G	T	NM_024747	c.898G>T:p.A300S	missense	0.0001	-	-	0.159:T	1	0	0	0	-

HPS6	10:103826163	C	T	NM_024747	c.932C>T:p.P311L	missense	0.0001	-	0.0001	0.202:T	0	1	0	0	-
HPS6	10:103826166	G	T	NM_024747	c.935G>T:p.W312L	missense	0.0009	0.0006	0.0006	0.301:T	4	3	3	0	-
HPS6	10:103826167	G	A	NM_024747	c.936G>A:p.W312X	stopgain	-	-	-	0.268:T	0	1	0	0	-
HPS6	10:103826210	G	A	NM_024747	c.979G>A:p.V327M	missense	-	-	-	0.776:D	2	3	1	2	-
HPS6	10:103826250	G	T	NM_024747	c.1019G>T:p.G340V	missense	-	-	-	0.947:D	1	0	0	0	-
HPS6	10:103826276	A	G	NM_024747	c.1045A>G:p.S349G	missense	5.80E-05	0.0006	-	0.711:D	0	1	0	0	-
HPS6	10:103826287	G	T	NM_024747	c.1056G>T:p.R352S	missense	5.80E-05	-	-	0.815:D	0	1	0	0	-
HPS6	10:103826329	G	T	NM_024747	c.1098G>T:p.E366D	missense	-	-	-	0.229:T	0	2	0	0	-
HPS6	10:103826377	TC	-	NM_024747	c.1146_1147del:p.L383Vfs*11	frameshift deletion	-	-	-	-	0	2	0	0	-
HPS6	10:103826385	G	A	NM_024747	c.1154G>A:p.C385Y	missense	-	-	-	0.306:T	0	0	1	0	-
HPS6	10:103826448	A	G	NM_024747	c.1217A>G:p.E406G	missense	0.0001	-	0.0002	0.930:D	0	0	1	0	-
HPS6	10:103826484	G	A	NM_024747	c.1253G>A:p.G418D	missense	0.0002	0	0.0003	0.870:D	1	0	0	0	-
HPS6	10:103826492	C	A	NM_024747	c.1261C>A:p.L421I	missense	-	-	-	0.579:T	0	1	0	0	-
HPS6	10:103826529	G	A	NM_024747	c.1298G>A:p.R433Q	missense	5.80E-05	0.0006	0	0.218:T	0	1	0	0	-
HPS6	10:103826609	C	T	NM_024747	c.1378C>T:p.R460W	missense	0	0	0	0.660:T	2	1	2	0	-
HPS6	10:103826618	C	T	NM_024747	c.1387C>T:p.R463X	stopgain	0	-	-	0.557:T	0	1	2	0	-
HPS6	10:103826640	C	A	NM_024747	c.1409C>A:p.A470D	missense	-	-	-	0.675:T	0	1	0	0	-
HPS6	10:103826679	C	A	NM_024747	c.1448C>A:p.T483N	missense	-	-	-	0.318:T	0	0	1	0	-
HPS6	10:103826688	C	T	NM_024747	c.1457C>T:p.A486V	missense	0.0005	-	0.0003	0.265:T	2	0	2	0	-
HPS6	10:103826815	G	C	NM_024747	c.1584G>C:p.Q528H	missense	-	-	-	0.639:T	0	0	1	0	-
HPS6	10:103826838	G	T	NM_024747	c.1607G>T:p.R536M	missense	-	-	-	0.597:T	0	0	0	1	-
HPS6	10:103826839	G	T	NM_024747	c.1608G>T:p.R536S	missense	-	-	-	0.349:T	1	0	0	0	-
HPS6	10:103826840	-	C	NM_024747	c.1610dupC:p.Q538Pfs*4	frameshift insertion	-	-	-	-	1	0	0	0	-
HPS6	10:103826850	C	G	NM_024747	c.1619C>G:p.P540R	missense	-	-	-	0.715:D	1	1	0	1	-
HPS6	10:103826888	G	A	NM_024747	c.1657G>A:p.A553T	missense	0	0	0	0.256:T	0	0	0	1	-
HPS6	10:103826899	A	C	NM_024747	c.1668A>C:p.E556D	missense	0.0005	-	0.0001	0.131:T	0	0	1	0	-
HPS6	10:103826955	T	A	NM_024747	c.1724T>A:p.L575Q	missense	-	-	-	0.686:T	0	2	1	0	-
HPS6	10:103826960	C	T	NM_024747	c.1729C>T:p.P577S	missense	-	-	-	0.929:D	0	0	1	0	-
HPS6	10:103827009	C	T	NM_024747	c.1778C>T:p.P593L	missense	0	0	0	0.346:T	1	0	0	0	-

HPS6	10:103827009	C	G	NM_024747	c.1778C>G:p.P593R	missense	-	-	-	0.471:T	0	0	0	3	-
HPS6	10:103827014	T	G	NM_024747	c.1783T>G:p.W595G	missense	-	-	-	0.667:T	1	0	0	6	-
HPS6	10:103827020	G	A	NM_024747	c.1789G>A:p.A597T	missense	0	-	0.0001	0.321:T	1	1	1	0	-
HPS6	10:103827023	G	C	NM_024747	c.1792G>C:p.G598R	missense	5.86E-05	-	0.0001	0.441:T	0	1	0	0	-
HPS6	10:103827051	G	A	NM_024747	c.1820G>A:p.R607Q	missense	0	0	0	0.912:D	0	1	0	0	-
HPS6	10:103827182	C	T	NM_024747	c.1951C>T:p.R651W	missense	0.001	0.0025	0.0009	0.811:D	5	0	3	1	US
HPS6	10:103827218	A	G	NM_024747	c.1987A>G:p.S663G	missense	0.0002	-	0.0001	0.797:D	0	1	0	0	-
HPS6	10:103827230	C	T	NM_024747	c.1999C>T:p.R667X	stopgain	0	-	0	0.601:T	0	1	0	0	LP
HPS6	10:103827300	T	C	NM_024747	c.2069T>C:p.L690P	missense	-	-	-	0.810:D	1	0	0	0	-
HPS6	10:103827308	C	T	NM_024747	c.2077C>T:p.R693C	missense	0.0001	-	0	0.298:T	1	0	0	0	-
HPS6	10:103827309	G	A	NM_024747	c.2078G>A:p.R693H	missense	5.8E-05	0	0	0.305:T	0	0	2	0	-
HPS6	10:103827408	G	A	NM_024747	c.2177G>A:p.G726E	missense	-	-	-	0.562:T	0	0	1	0	-
HPS6	10:103827414	A	C	NM_024747	c.2183A>C:p.E728A	missense	-	-	-	0.523:T	0	0	1	0	-
HPS6	10:103827480	C	T	NM_024747	c.2249C>T:p.S750L	missense	0.0003	0.0012	0.0005	0.299:T	0	0	1	1	-
CTSD	11:1774810	C	T	NM_001909	c.1162G>A:p.V388I	missense	0	0	0	0.574:T	0	1	0	0	US
CTSD	11:1774843	G	A	NM_001909	c.1129C>T:p.P377S	missense	-	-	-	0.447:T	0	1	0	0	-
CTSD	11:1774843	G	T	NM_001909	c.1129C>A:p.P377T	missense	-	-	-	0.501:T	0	1	0	1	-
CTSD	11:1774858	C	T	NM_001909	c.1114G>A:p.G372S	missense	-	-	-	0.508:T	0	0	1	0	-
CTSD	11:1774875	C	A	NM_001909	c.1097G>T:p.C366F	missense	-	-	-	0.972:D	0	1	0	0	-
CTSD	11:1774888	C	T	NM_001909	c.1084G>A:p.G362R	missense	0	0	0	0.880:D	1	0	0	0	US
CTSD	11:1775053	G	A	NM_001909	c.1051C>T:p.P351S	missense	0	0	-	0.442:T	1	0	0	0	-
CTSD	11:1775056	A	G	NM_001909	c.1048T>C:p.S350P	missense	-	-	-	0.478:T	1	0	0	0	-
CTSD	11:1775059	G	T	NM_001909	c.1045C>A:p.L349M	missense	-	-	-	0.479:T	0	1	0	0	-
CTSD	11:1775095	C	T	NM_001909	c.1009G>A:p.A337T	missense	0.0029	0.0043	0.003	0.250:T	8	4	6	3	CIP
CTSD	11:1775251	G	C	NM_001909	c.945C>G:p.I315M	missense	-	-	-	0.470:T	1	0	0	0	-
CTSD	11:1775270	C	T	NM_001909	c.926G>A:p.R309H	missense	0.0008	0.0006	0.001	0.287:T	0	1	1	1	CIP
CTSD	11:1775333	T	C	NM_001909	c.863A>G:p.E288G	missense	0.0002	-	0.0001	0.180:T	2	1	0	0	-
CTSD	11:1775352	C	T	NM_001909	c.844G>A:p.G282R	missense	0.0001	0	0.0003	0.248:T	0	0	1	0	B/LB
CTSD	11:1775364	C	G	NM_001909	c.832G>C:p.E278Q	missense	-	-	-	0.117:T	0	1	0	1	-
CTSD	11:1776164	T	C	NM_001909	c.799A>G:p.K267E	missense	0	-	0	0.353:T	0	0	1	0	-
CTSD	11:1776205	T	C	NM_001909	c.758A>G:p.K253R	missense	0	0	0	0.198:T	0	0	1	0	US
CTSD	11:1776250	T	C	NM_001909	c.713A>G:p.D238G	missense	0	-	0	0.172:T	1	0	0	0	-

CTSD	11:1778698	C	G	NM_001909	c.560G>C:p.G187A	missense	-	-	-	0.734:D	0	0	0	1	-
CTSD	11:1778710	G	T	NM_001909	c.548C>A:p.T183N	missense	-	-	-	0.463:T	0	0	0	1	-
CTSD	11:1778734	T	C	NM_001909	c.524A>G:p.E175G	missense	-	-	-	0.263:T	0	1	0	0	-
CTSD	11:1778740	T	C	NM_001909	c.518A>G:p.K173R	missense	0	-	0	0.283:T	0	0	0	1	US
CTSD	11:1780248	T	C	NM_001909	c.422A>G:p.H141R	missense	5.80E-05	0.0006	0.0001	0.206:T	1	1	3	0	-
CTSD	11:1780294	C	T	NM_001909	c.376G>A:p.D126N	missense	-	0	-	0.008:T	0	0	1	0	US
CTSD	11:1780752	C	T	NM_001909	c.346G>A:p.A116T	missense	0	-	0	0.576:T	0	0	0	1	US
CTSD	11:1782606	T	C	NM_001909	c.161A>G:p.K54R	missense	-	-	-	0.270:T	1	0	0	1	-
CTSD	11:1782613	C	T	NM_001909	c.154G>A:p.V52I	missense	0.0024	0.0037	0.0028	0.060:T	10	3	7	6	CIP
CTSD	11:1782682	A	C	NM_001909	c.85T>G:p.F29V	missense	-	-	-	0.622:T	0	1	0	0	-
CTSD	11:1785076	C	G	NM_001909	c.14G>C:p.S5T	missense	0.0018	0.0019	0.0068	0.117:T	8	4	6	0	CIP
SMPD1	11:6411878	G	A	NM_000543	c.50G>A:p.R17Q	missense	0.0001	-	0.0001	0.128:T	0	0	1	0	-
SMPD1	11:6411959	C	T	NM_000543	c.131C>T:p.A44V	missense	5.8E-05	-	-	0.137:T	0	0	1	0	-
SMPD1	11:6412016	C	T	NM_000543	c.188C>T:p.P63L	missense	0	-	-	0.479:T	1	0	0	0	-
SMPD1	11:6412103	C	T	NM_000543	c.275C>T:p.P92L	missense	-	-	-	0.755:D	0	0	0	1	-
SMPD1	11:6412133	A	G	NM_000543	c.305A>G:p.N102S	missense	0	-	0	0.322:T	0	1	1	1	US
SMPD1	11:6412644	G	A	NM_000543	c.349G>A:p.V117M	missense	0.0039	0.0062	0.003	0.220:T	2	1	1	0	CIP
SMPD1	11:6412666	T	G	NM_000543	c.371T>G:p.L124R	missense	0.0004	0.0018	0.0003	0.510:T	5	4	2	2	-
SMPD1	11:6412689	G	A	NM_000543	c.394G>A:p.V132M	missense	0	0	0	0.838:D	1	1	0	0	US
SMPD1	11:6412715	T	G	NM_000543	c.420T>G:p.F140L	missense	5.80E-05	-	0.0001	0.815:D	1	1	1	0	-
SMPD1	11:6412728	G	A	NM_000543	c.433G>A:p.V145M	missense	5.80E-05	-	0.0001	0.806:D	0	1	0	0	-
SMPD1	11:6412743	C	T	NM_000543	c.448C>T:p.R150C	missense	0	0.0012	0	0.818:D	0	0	1	0	US
SMPD1	11:6412752	C	G	NM_000543	c.457C>G:p.L153V	missense	-	-	-	0.798:D	0	0	1	0	-
SMPD1	11:6412759	C	G	NM_000543	c.464C>G:p.P155R	missense	5.80E-05	-	-	0.920:D	2	0	0	1	LP
SMPD1	11:6412824	A	T	NM_000543	c.529A>T:p.N177Y	missense	0.0001	-	0.0003	0.819:D	0	1	1	1	US
SMPD1	11:6412836	C	T	NM_000543	c.541C>T:p.P181S	missense	-	-	-	0.611:T	0	1	0	0	-
SMPD1	11:6412852	C	T	NM_000543	c.557C>T:p.P186L	missense	0	0	-	0.877:D	1	0	0	0	P
SMPD1	11:6412853	-	C	NM_000543	c.559dupC:p.K189Qfs*3	frameshift insertion	9.6E-05	0.0013	0.0013	-	0	0	0	5	-
SMPD1	11:6412854	C	A	NM_000543	c.559C>A:p.P187T	missense	0	-	0	0.545:T	0	1	0	1	-
SMPD1	11:6412860	A	C	NM_000543	c.565A>C:p.K189Q	missense	0.0004	0.0026	-	0.309:T	0	0	1	4	-

SMPD1	11:6412861	A	C	NM_000543	c.566A>C:p.K189T	missense	-	-	-	0.421:T	0	0	1	1	-
SMPD1	11:6412933	A	C	NM_000543	c.638A>C:p.H213P	missense	-	-	-	0.754:D	0	2	0	1	-
SMPD1	11:6412951	C	T	NM_000543	c.656C>T:p.T219M	missense	5.85E-05	-	0.0001	0.150:T	1	0	0	0	US
SMPD1	11:6412972	C	A	NM_000543	c.677C>A:p.P226Q	missense	-	-	-	0.883:D	1	0	0	1	-
SMPD1	11:6412986	C	T	NM_000543	c.691C>T:p.R231W	missense	0	0	0	0.367:T	0	0	1	0	-
SMPD1	11:6412987	G	A	NM_000543	c.692G>A:p.R231Q	missense	0.0001	0	0.0001	0.154:T	0	0	1	0	-
SMPD1	11:6413001	C	T	NM_000543	c.706C>T:p.P236S	missense	0	-	-	0.459:T	0	0	1	0	-
SMPD1	11:6413007	G	A	NM_000543	c.712G>A:p.A238T	missense	0	-	0	0.005:T	0	0	1	0	US
SMPD1	11:6413037	G	A	NM_000543	c.742G>A:p.E248K	missense	0	0	-	0.819:D	1	0	0	0	P/LP
SMPD1	11:6413101	C	T	NM_000543	c.806C>T:p.A269V	missense	0.0004	0.0006	0.0001	0.502:T	0	1	0	0	-
SMPD1	11:6413106	C	A	NM_000543	c.811C>A:p.P271T	missense	-	-	-	0.777:D	0	0	1	0	-
SMPD1	11:6413134	-	CATCCCCG	NM_000543	c.839_840insCATCCCCG:p.H284Sfs*17	frameshift insertion	-	-	-	-	0	1	0	0	-
SMPD1	11:6413181	C	T	NM_000543	c.886C>T:p.R296W	missense	5.8E-05	-	-	0.659:T	0	0	1	0	US
SMPD1	11:6413182	G	A	NM_000543	c.887G>A:p.R296Q	missense	0	0	0	0.268:T	0	1	0	0	B/LB
SMPD1	11:6413200	C	T	NM_000543	c.905C>T:p.T302I	missense	-	-	-	0.781:D	0	0	1	0	-
SMPD1	11:6413240	C	A	NM_000543	c.945C>A:p.Y315X	stopgain	-	-	-	0.293:T	1	0	0	0	-
SMPD1	11:6413290	C	G	NM_000543	c.995C>G:p.P332R	missense	0.0056	0.0012	0.0052	0.892:D	43	14	36	17	CIP
SMPD1	11:6413290	C	A	NM_000543	c.995C>A:p.P332H	missense	0	0	0	0.779:D	0	0	1	0	-
SMPD1	11:6413296	T	C	NM_000543	c.1001T>C:p.I334T	missense	0	-	0	0.625:T	2	1	2	1	-
SMPD1	11:6413305	A	G	NM_000543	c.1010A>G:p.N337S	missense	5.80E-05	-	0	0.376:T	1	0	0	0	US
SMPD1	11:6413317	G	A	NM_000543	c.1022G>A:p.R341H	missense	0	-	0	0.227:T	1	1	0	1	-
SMPD1	11:6413349	G	C	NM_000543	c.1054G>C:p.E352Q	missense	0	-	-	0.253:T	1	0	0	0	-
SMPD1	11:6413366	C	T	NM_001318088	c.110C>T:p.P37L	missense	0	0	0	-	0	2	0	0	B/LB
SMPD1	11:6413377	G	A	NM_000543	c.1082G>A:p.R361H	missense	5.8E-05	0	0	0.340:T	0	0	0	1	US
SMPD1	11:6413385	A	G	NM_000543	c.1090A>G:p.R364G	missense	5.8E-05	-	-	0.922:D	0	0	0	1	LP
SMPD1	11:6414471	C	G	NM_000543	c.1117C>G:p.P373A	missense	-	-	-	0.496:T	0	1	0	0	P
SMPD1	11:6414487	G	A	NM_000543	c.1133G>A:p.R378H	missense	0.0002	0	0	0.991:D	0	2	0	1	CIP
SMPD1	11:6414558	G	A	NM_000543	c.1204G>A:p.A402T	missense	0	-	0.0001	0.879:D	2	0	1	0	-
SMPD1	11:6414602	G	-	NM_000543	c.1248delG:p.D417Ifs*7	frameshift deletion	-	-	-	-	0	0	0	1	-

SMPD1	11:6414607	G	A	NM_000543	c.1253G>A:p.R418Q	missense	0	0	0	0.259:T	0	1	0	1	p.R418X
SMPD1	11:6415131	A	C	NM_000543	c.1346A>C:p.E449A	missense	-	-	-	0.850:D	0	0	0	1	-
SMPD1	11:6415215	C	T	NM_000543	c.1430C>T:p.P477L	missense	0	0	0	0.917:D	0	1	0	0	P/LP
SMPD1	11:6415221	-	TGTA	NM_000543	c.1436_1437insTGTA:p.A481Cfs*14	frameshift insertion	-	-	-	-	1	0	0	0	-
SMPD1	11:6415243	T	G	NM_000543	c.1458T>G:p.S486R	missense	-	-	-	0.837:D	2	0	0	0	LP
SMPD1	11:6415245	C	T	NM_000543	c.1460C>T:p.A487V	missense	0	0	0	0.668:T	1	0	0	0	CIP
SMPD1	11:6415259	G	A	NM_000543	c.1474G>A:p.G492S	missense	0.0002	0.0006	0	0.316:T	3	1	0	0	CIP
SMPD1	11:6415416	A	T	NM_001318087	c.1495A>T:p.T499S	missense	-	-	-	-	0	0	0	1	-
SMPD1	11:6415434	G	C	NM_000543	c.1493G>C:p.R498P	missense	0	-	-	0.967:D	1	0	0	0	p.R498H/p.R498L
SMPD1	11:6415438	G	A	NM_001318087	c.1517G>A:p.C506Y	missense	0.0002	0.0012	0.0001	-	0	0	1	0	LB
SMPD1	11:6415439	T	C	NM_000543	c.1498T>C:p.Y500H	missense	0.0002	0.0012	0.0001	0.929:D	0	0	1	0	US
SMPD1	11:6415475	G	A	NM_000543	c.1534G>A:p.V512M	missense	0	0	0	0.363:T	0	1	0	0	LB
SMPD1	11:6415494	C	T	NM_000543	c.1553C>T:p.T518I	missense	-	-	-	0.931:D	0	0	0	1	LP
SMPD1	11:6415517	G	A	NM_000543	c.1576G>A:p.A526T	missense	-	-	-	0.753:D	0	0	0	1	-
SMPD1	11:6415539	C	T	NM_000543	c.1598C>T:p.P533L	missense	0.007	0.0136	0.0072	0.699:T	17	14	24	8	CIP
SMPD1	11:6415639	G	T	NM_000543	c.1698G>T:p.M566I	missense	0.0002	-	0.0001	0.258:T	0	1	0	0	US
SMPD1	11:6415640	C	T	NM_000543	c.1699C>T:p.Q567X	stopgain	-	-	-	0.386:T	1	1	0	0	-
SMPD1	11:6415685	C	T	NM_000543	c.1744C>T:p.P582S	missense	0.0001	-	0.0001	0.662:T	1	0	0	0	-
SMPD1	11:6415704	C	T	NM_000543	c.1763C>T:p.T588M	missense	0.001	0.0012	0.0008	0.280:T	5	5	5	2	B/LB
SMPD1	11:6415713	G	A	NM_000543	c.1772G>A:p.R591H	missense	0	0	0	0.657:T	0	0	1	0	US
SMPD1	11:6415730	G	A	NM_000543	c.1789G>A:p.A597T	missense	-	-	-	0.700:D	0	0	1	0	-
SMPD1	11:6415746	G	A	NM_000543	c.1805G>A:p.R602H	missense	0	-	0	0.781:D	2	0	1	1	P/LP
SMPD1	11:6415769	C	T	NM_000543	c.1828C>T:p.R610C	missense	0	0	0	0.778:D	0	1	0	0	-
SMPD1	11:6415770	G	A	NM_000543	c.1829G>A:p.R610H	missense	0	0	0	0.514:T	1	0	0	0	-
SMPD1	11:6415821	G	A	NM_000543	c.1880G>A:p.R627K	missense	-	-	-	0.054:T	0	0	1	1	-
SMPD1	11:6415833	G	A	NM_000543	c.1892G>A:p.C631Y	missense	-	-	-	0.714:D	0	1	0	0	-
TPP1	11:6635808	G	A	NM_000391	c.1661C>T:p.P554L	missense	-	-	-	0.907:D	0	1	0	0	-
TPP1	11:6636096	-	ATCA	NM_000391	c.1551+1->TGAT	splicing	5.80E-05	-	0.0001	-	0	1	0	0	-
TPP1	11:6636113	C	G	NM_000391	c.1535G>C:p.G512A	missense	-	-	-	0.805:D	1	0	0	0	-

TPP1	11:6636187	C	G	NM_000391	c.1461G>C:p.L487F	missense	0	0	0	0.867:D	1	0	0	0	-
TPP1	11:6636499	T	C	NM_000391	c.1328A>G:p.N443S	missense	0.0003	0.0006	0.0003	0.563:T	1	0	0	0	US
TPP1	11:6636547	G	A	NM_000391	c.1280C>T:p.T427M	missense	5.80E-05	0	0	0.332:T	1	0	1	0	CIP
TPP1	11:6636686	C	T	NM_000391	c.1253G>A:p.R418Q	missense	0	0	0	0.257:T	2	0	0	0	B/LB
TPP1	11:6636711	C	T	NM_000391	c.1228G>A:p.G410S	missense	5.8E-05	-	-	0.991:D	0	0	1	0	US
TPP1	11:6636722	T	C	NM_000391	c.1217A>G:p.Y406C	missense	0	-	0	0.953:D	0	1	0	0	US
TPP1	11:6637265	G	C	NM_000391	c.1116C>G:p.H372Q	missense	0.0003	0.0012	0.0002	0.508:T	3	0	1	0	US
TPP1	11:6637732	G	A	NM_000391	c.889C>T:p.R297W	missense	0	0	0	0.948:D	0	0	0	1	US
TPP1	11:6637945	T	C	NM_000391	c.833A>G:p.Q278R	missense	0	0	-	0.892:D	1	0	0	0	P/LP
TPP1	11:6637976	G	A	NM_000391	c.802C>T:p.R268W	missense	0	-	0	0.530:T	0	1	0	0	US
TPP1	11:6637982	G	A	NM_000391	c.796C>T:p.R266W	missense	0	0	0	0.568:T	0	0	1	0	CIP
TPP1	11:6638018	G	T	NM_000391	c.760C>A:p.Q254K	missense	-	-	-	0.416:T	1	0	0	0	US
TPP1	11:6638084	C	G	NM_000391	c.694G>C:p.E232Q	missense	-	-	-	0.753:D	1	0	0	0	-
TPP1	11:6638247	C	T	NM_000391	c.646G>A:p.V216M	missense	-	-	-	0.858:D	0	1	0	1	-
TPP1	11:6638270	C	T	NM_000391	c.623G>A:p.R208Q	missense	0.0001	0	0	0.871:D	2	1	1	0	p.R208X
TPP1	11:6638271	G	A	NM_000391	c.622C>T:p.R208X	stopgain	0	0	0	0.653:T	1	0	0	0	P
TPP1	11:6638273	T	C	NM_000391	c.620A>G:p.K207R	missense	-	-	-	0.340:T	1	0	0	0	-
TPP1	11:6638303	T	A	NM_000391	c.590A>T:p.H197L	missense	-	-	-	0.873:D	0	0	1	0	-
TPP1	11:6638340	G	A	NM_000391	c.553C>T:p.R185C	missense	0.0003	0	0.0001	0.286:T	0	1	1	0	B/LB
TPP1	11:6638577	G	A	NM_000391	c.463C>T:p.H155Y	missense	-	-	-	0.218:T	0	1	0	0	-
TPP1	11:6638923	G	C	NM_000391	c.314C>G:p.A105G	missense	-	-	-	0.089:T	1	0	0	0	-
TPP1	11:6638937	T	G	NM_000391	c.300A>C:p.Q100H	missense	5.80E-05	-	-	0.242:T	1	0	0	0	-
TPP1	11:6638980	G	C	NM_000391	c.257C>G:p.A86G	missense	0.0001	-	0.0001	0.398:T	0	1	0	1	-
TPP1	11:6639008	C	G	NM_000391	c.230-1G>C	splicing	-	-	-	0.666:T	1	0	0	0	LP
TPP1	11:6640048	T	A	NM_000391	c.188A>T:p.E63V	missense	0.0002	0.0006	0.0002	0.584:T	0	1	1	0	-
TPP1	11:6640117	C	T	NM_000391	c.119G>A:p.R40H	missense	0.0002	-	0.0003	0.689:T	2	1	0	0	US
TPP1	11:6640430	C	T	NM_000391	c.86G>A:p.R29K	missense	0.0002	-	-	0.203:T	1	0	2	0	-
TPP1	11:6640437	G	C	NM_000391	c.79C>G:p.Q27E	missense	0.0002	-	0.0002	0.431:T	0	1	1	0	US
TPP1	11:6640481	G	A	NM_000391	c.35C>T:p.A12V	missense	-	-	-	0.768:D	1	0	0	0	US
HPS5	11:18301473	T	C	NM_007216	c.3004A>G:p.M1002V	missense	-	-	-	0.639:T	5	3	0	0	-
HPS5	11:18301475	C	A	NM_007216	c.3002G>T:p.S1001I	missense	-	-	-	0.480:T	1	0	0	0	-
HPS5	11:18303569	C	A	NM_007216	c.2915G>T:p.G972V	missense	0.0003	-	0.0003	0.647:T	3	3	1	1	US

HPS5	11:18303597	G	A	NM_007216	c.2887C>T:p.R963W	missense	0.0187	0.0099	0.0201	0.567:T	66	73	61	38	B
HPS5	11:18303624	G	C	NM_007216	c.2860C>G:p.L954V	missense	-	-	-	0.553:T	1	0	0	0	-
HPS5	11:18303633	C	T	NM_007216	c.2851G>A:p.V951M	missense	-	-	-	0.633:T	0	0	1	0	-
HPS5	11:18303690	G	A	NM_007216	c.2794C>T:p.Q932X	stopgain	-	-	-	0.416:T	0	1	0	0	-
HPS5	11:18303704	G	A	NM_007216	c.2780C>T:p.T927M	missense	0.0002	0.0006	-	0.221:T	0	3	1	0	-
HPS5	11:18303759	G	C	NM_007216	c.2725C>G:p.P909A	missense	-	-	-	0.837:D	0	0	1	0	-
HPS5	11:18303764	C	G	NM_007216	c.2720G>C:p.W907S	missense	-	-	-	0.396:T	0	0	0	1	-
HPS5	11:18305355	C	T	NM_007216	c.2703G>A:p.M901I	missense	0	0	0	0.236:T	0	2	0	0	LB
HPS5	11:18305440	G	A	NM_007216	c.2618C>T:p.P873L	missense	-	-	-	0.559:T	0	0	0	1	-
HPS5	11:18306912	C	T	NM_007216	c.2590G>A:p.D864N	missense	-	-	-	0.137:T	0	0	1	0	-
HPS5	11:18306916	C	T	NM_007216	c.2586G>A:p.M862I	missense	0	-	0	0.562:T	1	0	0	0	-
HPS5	11:18308180	G	A	NM_007216	c.2453C>T:p.A818V	missense	0.0001	0.0006	-	0.696:T	0	0	1	0	-
HPS5	11:18309082	C	T	NM_007216	c.2375G>A:p.R792Q	missense	0.0003	0	0.0003	0.390:T	0	0	0	1	-
HPS5	11:18309085	T	C	NM_007216	c.2372A>G:p.Q791R	missense	-	-	-	0.121:T	0	0	1	0	-
HPS5	11:18309086	G	C	NM_007216	c.2371C>G:p.Q791E	missense	-	-	-	0.187:T	0	1	0	0	-
HPS5	11:18309140	G	T	NM_007216	c.2317C>A:p.P773T	missense	0	-	0	0.537:T	0	3	0	1	-
HPS5	11:18309211	G	A	NM_007216	c.2246C>T:p.A749V	missense	-	-	-	0.717:D	2	0	0	0	-
HPS5	11:18309441	G	A	NM_007216	c.2218C>T:p.R740W	missense	0	0	0	0.676:T	0	0	0	1	-
HPS5	11:18309464	G	A	NM_007216	c.2195C>T:p.P732L	missense	0.0002	0.0006	0.0001	0.882:D	0	1	0	0	B
HPS5	11:18309524	T	C	NM_007216	c.2135A>G:p.E712G	missense	0.0003	0.0006	0.0007	0.382:T	0	0	3	0	-
HPS5	11:18313078	A	G	NM_007216	c.2009T>C:p.L670P	missense	0.0001	0.0012	0.0002	0.961:D	0	0	1	0	-
HPS5	11:18313118	C	T	NM_007216	c.1969G>A:p.E657K	missense	0	-	0.0001	0.646:T	0	2	0	0	-
HPS5	11:18313229	G	A	NM_007216	c.1858C>T:p.R620W	missense	0	0	0	0.439:T	1	1	0	0	US
HPS5	11:18313250	A	G	NM_007216	c.1837T>C:p.C613R	missense	0	-	-	0.720:D	0	0	2	0	-
HPS5	11:18313304	A	G	NM_007216	c.1783T>C:p.C595R	missense	0	0	-	0.214:T	0	0	1	0	-
HPS5	11:18313324	A	G	NM_007216	c.1763T>C:p.V588A	missense	-	-	-	0.022:T	0	1	0	0	-
HPS5	11:18313363	T	C	NM_007216	c.1724A>G:p.E575G	missense	0	-	-	0.235:T	0	1	0	0	-
HPS5	11:18313528	T	G	NM_007216	c.1559A>C:p.E520A	missense	-	-	-	0.248:T	1	0	1	0	-
HPS5	11:18313529	C	T	NM_007216	c.1558G>A:p.E520K	missense	0.0059	0.0099	0.0064	0.299:T	37	27	47	18	LB
HPS5	11:18314471	G	T	NM_007216	c.1495C>A:p.L499I	missense	0.0002	0.0006	0.0001	0.572:T	3	0	2	0	US
HPS5	11:18316652	T	C	NM_007216	c.1357A>G:p.K453E	missense	-	-	-	0.386:T	0	0	1	0	-
HPS5	11:18316666	G	A	NM_007216	c.1343C>T:p.T448M	missense	5.8E-05	0	0.0001	0.230:T	0	0	1	0	-

HPS5	11:18316718	T	C	NM_007216	c.1293-2A>G	splicing	5.82E-05	-	-	0.712:D	1	0	0	0	-
HPS5	11:18317602	A	C	NM_007216	c.1236T>G:p.I412M	missense	-	-	-	0.352:T	1	0	0	0	-
HPS5	11:18317612	G	A	NM_007216	c.1226C>T:p.P409L	missense	0	-	0	0.642:T	1	0	0	0	-
HPS5	11:18317617	AA	-	NM_007216	c.1220_1221del:p.F407Sfs*26	frameshift deletion	-	-	-	-	0	0	0	1	-
HPS5	11:18318380	A	T	NM_007216	c.1133T>A:p.L378Q	missense	0.0004	0.0006	0.0006	0.120:T	3	2	5	4	US
HPS5	11:18318443	T	C	NM_007216	c.1070A>G:p.H357R	missense	-	-	-	0.302:T	0	1	0	1	-
HPS5	11:18318530	T	C	NM_007216	c.983A>G:p.E328G	missense	0.0003	-	0.0005	0.651:T	0	1	1	0	US
HPS5	11:18319132	T	G	NM_007216	c.955A>C:p.S319R	missense	-	-	-	0.736:D	0	1	0	0	-
HPS5	11:18320427	C	T	NM_007216	c.734G>A:p.R245H	missense	0	-	0	0.627:T	2	0	0	0	US
HPS5	11:18320493	C	G	NM_007216	c.668G>C:p.R223T	missense	-	-	-	0.374:T	1	0	0	0	-
HPS5	11:18322381	C	T	NM_007216	c.637G>A:p.V213I	missense	-	-	-	0.257:T	0	0	0	1	-
HPS5	11:18322396	G	C	NM_007216	c.622C>G:p.L208V	missense	-	-	-	0.490:T	0	0	1	0	-
HPS5	11:18322425	T	C	NM_007216	c.593A>G:p.Y198C	missense	0.0001	-	0.0002	0.944:D	5	4	1	5	-
HPS5	11:18322459	G	A	NM_007216	c.559C>T:p.H187Y	missense	-	-	-	0.537:T	0	1	0	1	-
HPS5	11:18327023	T	A	NM_007216	c.500A>T:p.D167V	missense	-	-	-	0.485:T	0	0	1	0	-
HPS5	11:18327029	T	C	NM_007216	c.494A>G:p.Q165R	missense	0.0001	-	0.0001	0.245:T	0	0	1	0	-
HPS5	11:18327778	G	C	NM_007216	c.386C>G:p.S129C	missense	0.0003	-	0.0002	0.847:D	1	0	0	0	-
HPS5	11:18327787	C	T	NM_007216	c.377G>A:p.R126H	missense	0	-	0	0.982:D	0	1	0	0	p.R126P
HPS5	11:18327787	C	G	NM_007216	c.377G>C:p.R126P	missense	0.0002	0.0006	-	0.987:D	1	1	0	0	P
HPS5	11:18327797	A	G	NM_007216	c.367T>C:p.Y123H	missense	0	-	0	0.886:D	0	0	1	0	-
HPS5	11:18327798	T	C	NM_007216	c.366A>G:p.I122M	missense	-	-	-	0.780:D	0	0	1	0	-
HPS5	11:18327865	T	C	NM_007216	c.299A>G:p.E100G	missense	-	-	-	0.759:D	0	0	0	1	-
HPS5	11:18330518	G	A	NM_007216	c.244C>T:p.R82X	stopgain	-	-	-	0.872:D	0	1	0	0	-
HPS5	11:18330536	G	A	NM_007216	c.226C>T:p.L76F	missense	-	-	-	0.927:D	0	1	0	0	-
HPS5	11:18330590	T	C	NM_007216	c.172A>G:p.I58V	missense	0.0002	0	0.0002	0.144:T	1	1	1	1	-
HPS5	11:18332362	T	C	NM_007216	c.61A>G:p.I21V	missense	0.0003	0.0006	0.0005	0.127:T	2	2	0	1	-
HPS5	11:18332410	A	G	NM_007216	c.13T>C:p.S5P	missense	0.0002	-	0.0003	0.577:T	0	0	0	1	-
HPS5	11:18332430	G	A	NM_181507	c.335C>T:p.P112L	missense	0	0	-	0.771:D	0	1	0	1	-
HPS5	11:18332443	G	A	NM_181507	c.322C>T:p.R108C	missense	0.0016	0.0018	0.0013	0.655:T	0	1	0	0	-
HPS5	11:18332475	C	A	NM_181507	c.290G>T:p.G97V	missense	-	-	-	0.985:D	1	0	0	0	-
HPS5	11:18332958	T	C	NM_181507	c.260A>G:p.D87G	missense	-	-	-	0.587:T	1	0	0	0	-

HPS5	11:18333549	C	T	NM_181507	c.131G>A:p.R44Q	missense	0.0001	-	0	0.397:T	1	0	2	1	-
HPS5	11:18333556	C	T	NM_181507	c.124G>A:p.V42M	missense	-	-	-	0.668:T	0	0	1	0	-
HPS5	11:18339333	G	C	NM_181507	c.73C>G:p.L25V	missense	-	-	-	0.417:T	1	0	0	0	-
HPS5	11:18339344	A	G	NM_181507	c.62T>C:p.L21P	missense	5.80E-05	-	-	0.945:D	0	1	0	0	-
HPS5	11:18339368	T	A	NM_181507	c.38A>T:p.H13L	missense	-	-	-	0.801:D	0	0	0	1	-
HPS5	11:18343492	C	A	NM_181507	UTR5	splicing	-	-	-	-	0	0	0	1	-
CTSF	11:66332103	A	G	NM_003793	c.1247T>C:p.I416T	missense	-	-	-	0.892:D	0	0	0	1	P
CTSF	11:66332107	C	T	NM_003793	c.1243G>A:p.G415R	missense	0	-	0	0.812:D	0	0	1	1	-
CTSF	11:66332356	A	C	NM_003793	c.1165+2T>G	splicing	0.0002	0.0012	0.0002	0.623:T	0	1	0	1	-
CTSF	11:66332382	C	T	NM_003793	c.1141G>A:p.V381M	missense	0.0006	0.0006	0.0006	0.348:T	2	3	1	1	-
CTSF	11:66332407	C	A	NM_003793	c.1116G>T:p.K372N	missense	0	-	-	0.314:T	1	0	0	0	-
CTSF	11:66332436	T	C	NM_003793	c.1087A>G:p.M363V	missense	-	-	-	0.137:T	0	0	1	0	-
CTSF	11:66332438	T	C	NM_003793	c.1085A>G:p.H362R	missense	-	-	-	0.412:T	0	1	0	0	-
CTSF	11:66332446	G	C	NM_003793	c.1077C>G:p.Y359X	stopgain	-	-	-	0.305:T	0	1	0	0	-
CTSF	11:66333154	T	C	NM_003793	c.1033A>G:p.I345V	missense	-	-	-	0.401:T	0	0	0	1	-
CTSF	11:66333159	G	A	NM_003793	c.1028C>T:p.S343L	missense	5.8E-05	0	-	0.116:T	0	0	1	0	-
CTSF	11:66333168	T	C	NM_003793	c.1019A>G:p.N340S	missense	5.80E-05	-	0	0.382:T	2	0	0	0	-
CTSF	11:66333192	G	C	NM_003793	c.995C>G:p.A332G	missense	-	-	-	0.509:T	0	1	0	0	-
CTSF	11:66333207	T	C	NM_003793	c.980A>G:p.D327G	missense	-	-	-	0.788:D	0	1	0	0	-
CTSF	11:66333394	AT	-	NM_003793	c.871_872del:p.M291Vfs*28	frameshift deletion	0.0002	0.0006	0.0001	-	1	1	2	1	-
CTSF	11:66333582	T	C	NM_003793	c.778A>G:p.K260E	missense	0.0001	-	0.0001	0.234:T	0	0	2	0	-
CTSF	11:66333598	C	-	NM_003793	c.762delG:p.E256Sfs*5;CTSF	frameshift deletion	-	-	-	-	0	1	0	0	-
CTSF	11:66333598	C	-	NM_003793	c.762delG:p.E256Sfs*5;CTSF	frameshift deletion	-	-	-	-	0	1	0	0	-
CTSF	11:66333609	T	G	NM_003793	c.751A>C:p.T251P	missense	-	-	-	0.435:T	0	1	0	0	-
CTSF	11:66333807	G	A	NM_003793	c.676C>T:p.R226C	missense	0.0001	0	0	0.702:D	1	0	0	0	CIP
CTSF	11:66334955	C	T	NM_003793	c.491G>A:p.S164N	missense	0	-	0	0.026:T	0	1	0	0	-
CTSF	11:66334959	A	G	NM_003793	c.487T>C:p.F163L	missense	-	-	-	0.052:T	0	0	1	0	-
CTSF	11:66335030	G	T	NM_003793	c.416C>A:p.S139X	stopgain	0.0004	0.0012	0.0003	0.385:T	1	1	0	0	-
CTSF	11:66335133	G	C	NM_003793	c.313C>G:p.L105V;-	missense	0.0002	-	0.0002	0.332:T	2	2	1	0	-

CTSF	11:66335519	G	A	NM_003793	c.248C>T:p.T83I	missense	0	-	-	0.359:T	0	0	1	0	-
CTSF	11:66335522	-	C	NM_003793	c.244dupG:p.A82Gfs*31	frameshift insertion	-	-	-	-	0	0	1	0	-
CTSF	11:66335531	G	A	NM_003793	c.236C>T:p.S79F	missense	-	-	-	0.634:T	1	0	0	0	-
CTSF	11:66335752	A	C	NM_003793	c.206T>G:p.V69G	missense	0.0002	0	-	0.260:T	1	0	0	0	-
CTSF	11:66335827	C	T	NM_003793	c.131G>A:p.R44H	missense	0	-	-	0.425:T	0	0	1	0	-
GNS	12:65110546	C	T	NM_002076	c.1634G>A:p.R545Q	missense	0	0	0	0.253:T	0	1	0	0	-
GNS	12:65110562	C	T	NM_002076	c.1618G>A:p.G540S	missense	0	-	0	0.441:T	0	1	0	0	US
GNS	12:65110564	C	T	NM_002076	c.1616G>A:p.R539H	missense	5.80E-05	-	0.0001	0.164:T	0	1	0	0	US
GNS	12:65110580	G	A	NM_002076	c.1600C>T:p.L534F	missense	-	-	-	0.251:T	0	1	1	0	-
GNS	12:65113911	T	C	NM_002076	c.1471A>G:p.I491V	missense	-	-	-	0.528:T	0	1	0	0	-
GNS	12:65113913	T	C	NM_002076	c.1469A>G:p.N490S	missense	0	-	-	0.662:T	1	0	0	0	-
GNS	12:65115410	A	C	NM_002076	c.1384T>G:p.W462G	missense	-	-	-	0.507:T	0	1	0	0	-
GNS	12:65116832	C	T	NM_002076	c.1262G>A:p.R421H	missense	0	-	0	0.266:T	0	0	1	0	US
GNS	12:65116894	C	G	NM_002076	c.1201-1G>C	splicing	-	-	-	0.680:T	0	1	0	0	-
GNS	12:65122749	A	G	NM_002076	c.1187T>C:p.L396S	missense	-	-	-	0.653:T	0	0	1	0	-
GNS	12:65122781	G	T	NM_002076	c.1155C>A:p.D385E	missense	0.0029	0.0025	0.0021	0.279:T	8	5	3	0	-
GNS	12:65133205	T	C	NM_002076	c.950A>G:p.N317S	missense	-	-	-	0.413:T	1	0	0	0	-
GNS	12:65134417	T	C	NM_002076	c.844A>G:p.I282V	missense	-	-	-	0.110:T	0	1	0	0	-
GNS	12:65136959	A	T	NM_002076	c.754T>A:p.F252I	missense	-	-	-	0.246:T	0	0	0	1	-
GNS	12:65137025	C	A	NM_002076	c.688G>T:p.A230S	missense	-	-	-	0.242:T	0	0	0	1	-
GNS	12:65137064	C	T	NM_002076	c.649G>A:p.D217N	missense	5.80E-05	-	0.0001	0.019:T	2	0	0	0	-
GNS	12:65137075	A	G	NM_002076	c.638T>C:p.L213S	missense	5.80E-05	-	0	0.860:D	0	1	0	0	-
GNS	12:65138612	C	T	NM_002076	c.601G>A:p.V201M	missense	0	-	-	0.471:T	0	2	0	0	-
GNS	12:65138620	T	C	NM_002076	c.593A>G:p.N198S	missense	-	-	-	0.417:T	0	0	2	0	-
GNS	12:65138636	G	A	NM_002076	c.577C>T:p.R193W	missense	0	-	0	0.291:T	0	0	3	0	CIP
GNS	12:65141536	T	C	NM_002076	c.415A>G:p.M139V	missense	0	0	0	0.469:T	1	0	0	0	-
GNS	12:65141554	G	A	NM_002076	c.397C>T:p.P133S	missense	-	-	-	0.892:D	0	0	1	0	-
GNS	12:65141566	G	C	NM_002076	c.385C>G:p.P129A	missense	-	-	-	0.580:T	1	0	0	0	-
GNS	12:65141643	T	A	NM_002076	c.308A>T:p.Y103F	missense	-	-	-	0.845:D	0	1	0	0	-
GNS	12:65141659	T	C	NM_002076	c.292A>G:p.I98V	missense	5.80E-05	-	0.0001	0.486:T	2	0	0	0	-

GNS	12:65152941	C	T	NM_002076	c.116G>A:p.G39E	missense	-	-	-	0.471:T	2	0	2	1	-
GNS	12:65152977	A	T	NM_002076	c.80T>A:p.L27Q	missense	-	-	-	0.878:D	1	0	0	0	-
GNS	12:65152978	G	C	NM_002076	c.79C>G:p.L27V	missense	-	0.0006	-	0.480:T	1	1	1	1	-
GNS	12:65152984	C	T	NM_002076	c.73G>A:p.A25T	missense	9.94E-05	0	0.0025	0.313:T	0	1	0	0	US
GNS	12:65153017	C	T	NM_002076	c.40G>A:p.G14S	missense	-	-	-	0.380:T	0	1	0	0	-
GNPTAB	12:102140955	C	T	NM_024312	c.3758G>A:p.R1253Q	missense	5.80E-05	-	0.0001	0.404:T	1	1	1	1	US
GNPTAB	12:102140976	T	C	NM_024312	c.3737A>G:p.H1246R	missense	-	-	-	0.339:T	1	0	1	0	-
GNPTAB	12:102141003	C	T	NM_024312	c.3710G>A:p.R1237Q	missense	0.0025	0	0.0024	0.848:D	4	3	5	1	CIP
GNPTAB	12:102142899	A	G	NM_024312	c.3673T>C:p.F1225L	missense	5.80E-05	-	0.0002	0.595:T	0	2	1	0	-
GNPTAB	12:102142915	C	A	NM_024312	c.3657G>T:p.L1219F	missense	-	-	-	0.757:D	0	0	1	0	-
GNPTAB	12:102147154	C	T	NM_024312	c.3598G>A:p.E1200K	missense	5.80E-05	0	0	0.957:D	0	1	0	1	-
GNPTAB	12:102147180	C	T	NM_024312	c.3572G>A:p.R1191H	missense	0.0009	0.0006	0.0007	0.937:D	3	1	3	2	-
GNPTAB	12:102147187	G	A	NM_024312	c.3565C>T:p.R1189X	stopgain	0.0001	-	0.0002	0.705:D	1	0	0	0	P
GNPTAB	12:102147237	T	C	NM_024312	c.3515A>G:p.Y1172C	missense	5.80E-05	0	0	0.965:D	0	1	0	0	-
GNPTAB	12:102147270	G	A	NM_024312	c.3482C>T:p.A1161V	missense	-	-	-	0.747:D	1	0	0	0	-
GNPTAB	12:102151384	T	G	NM_024312	c.3301A>C:p.I1101L	missense	-	-	-	0.576:T	1	0	0	0	-
GNPTAB	12:102151417	G	C	NM_024312	c.3268C>G:p.L1090V	missense	-	-	-	0.724:D	1	0	0	0	-
GNPTAB	12:102151431	G	A	NM_024312	c.3254C>T:p.P1085L	missense	0	-	0	0.898:D	1	0	2	0	-
GNPTAB	12:102153866	T	C	NM_024312	c.3191A>G:p.D1064G	missense	-	-	-	0.412:T	0	1	0	0	-
GNPTAB	12:102153867	C	G	NM_024312	c.3190G>C:p.D1064H	missense	0.0006	-	0.0009	0.418:T	0	1	0	1	-
GNPTAB	12:102154906	T	C	NM_024312	c.3134A>G:p.Q1045R;-	missense	-	-	-	0.725:D	1	0	0	0	-
GNPTAB	12:102154923	T	A	NM_024312	c.3117A>T:p.E1039D	missense	-	-	-	0.334:T	0	0	1	0	-
GNPTAB	12:102154930	A	G	NM_024312	c.3110T>C:p.I1037T	missense	0.0005	0.0006	0.0002	0.913:D	3	1	3	3	-
GNPTAB	12:102154946	T	-	NM_024312	c.3094delA:p.T1032Hfs *10	frameshif t deletion	-	-	-	-	0	1	0	0	P
GNPTAB	12:102154949	G	A	NM_024312	c.3091C>T:p.R1031X	stopgain	5.80E-05	-	-	0.870:D	0	1	0	1	P
GNPTAB	12:102154972	C	T	NM_024312	c.3068G>A:p.G1023D	missense	-	-	-	0.983:D	0	1	0	0	-
GNPTAB	12:102155057	AGGC	-	NM_024312	c.2980_2983del:p.A994 Sfs*7	frameshif t deletion	-	-	-	-	1	0	0	0	-
GNPTAB	12:102155071	T	A	NM_024312	c.2969A>T:p.D990V	missense	-	-	-	0.982:D	0	0	1	0	-
GNPTAB	12:102155089	T	C	NM_024312	c.2951A>G:p.K984R	missense	-	-	-	0.372:T	0	0	0	1	-
GNPTAB	12:102155492	T	A	NM_024312	c.2765A>T:p.N922I	missense	-	-	-	0.211:T	1	0	0	0	-

GNPTAB	12:102157979	C	T	NM_024312	c.2715+1G>A	splicing	0.0002	-	0.0001	0.717:D	0	2	2	0	P
GNPTAB	12:102157982	C	T	NM_024312	c.2713G>A:p.D905N	missense	5.80E-05	0	0	0.301:T	0	1	0	1	-
GNPTAB	12:102158002	T	-	NM_024312	c.2693delA:p.K898Sfs*12	frameshift deletion	0	-	-	-	0	0	0	1	US
GNPTAB	12:102158027	C	G	NM_024312	c.2668G>C:p.G890R	missense	-	-	-	0.918:D	0	0	1	0	-
GNPTAB	12:102158051	G	A	NM_024312	c.2644C>T:p.Q882X	stopgain	-	-	-	0.867:D	1	0	0	0	-
GNPTAB	12:102158104	-	C	NM_024312	c.2590dupG:p.E864Gfs*3	frameshift insertion	-	-	-	-	0	0	1	0	-
GNPTAB	12:102158153	T	C	NM_024312	c.2542A>G:p.K848E	missense	-	-	-	0.342:T	0	0	1	0	-
GNPTAB	12:102158164	C	T	NM_024312	c.2531G>A:p.S844N	missense	0.0002	-	0.0001	0.219:T	3	1	1	0	-
GNPTAB	12:102158191	G	A	NM_024312	c.2504C>T:p.P835L	missense	0.0007	-	0.0006	0.267:T	2	5	1	1	US
GNPTAB	12:102158240	C	A	NM_024312	c.2455G>T:p.E819X	stopgain	-	-	-	0.357:T	0	0	0	1	-
GNPTAB	12:102158377	C	A	NM_024312	c.2318G>T:p.S773I	missense	0.0001	0.0006	-	0.164:T	5	3	2	2	-
GNPTAB	12:102158378	T	G	NM_024312	c.2317A>C:p.S773R	missense	0.0001	0.0006	-	0.101:T	5	3	1	2	-
GNPTAB	12:102158458	G	C	NM_024312	c.2237C>G:p.A746G	missense	-	-	-	0.202:T	0	1	0	0	-
GNPTAB	12:102158464	T	C	NM_024312	c.2231A>G:p.Q744R	missense	-	-	-	0.213:T	1	0	0	0	-
GNPTAB	12:102158576	C	T	NM_024312	c.2119G>A:p.A707T	missense	0	0	0	0.102:T	0	0	1	0	US
GNPTAB	12:102158605	A	C	NM_024312	c.2090T>G:p.L697R	missense	-	-	-	0.456:T	1	0	0	0	-
GNPTAB	12:102158624	C	T	NM_024312	c.2071G>A:p.E691K	missense	-	-	-	0.356:T	1	0	0	1	US
GNPTAB	12:102158702	G	C	NM_024312	c.1993C>G:p.L665V	missense	0	-	0	0.265:T	0	1	0	0	-
GNPTAB	12:102158707	T	G	NM_024312	c.1988A>C:p.E663A	missense	-	-	-	0.537:T	0	0	0	1	-
GNPTAB	12:102158820	G	T	NM_024312	c.1875C>A:p.F625L	missense	0	-	0	0.929:D	0	0	0	1	US
GNPTAB	12:102158838	A	C	NM_024312	c.1857T>G:p.N619K	missense	-	-	-	0.241:T	1	0	0	0	-
GNPTAB	12:102158875	T	C	NM_024312	c.1820A>G:p.N607S	missense	-	-	0.0001	0.502:T	2	0	0	2	-
GNPTAB	12:102158890	A	G	NM_024312	c.1805T>C:p.M602T	missense	-	0	-	0.758:D	0	1	0	0	-
GNPTAB	12:102158945	G	T	NM_024312	c.1750C>A:p.P584T	missense	0.0083	0.0074	0.0089	0.793:D	29	42	28	15	B/LB
GNPTAB	12:102158947	T	C	NM_024312	c.1748A>G:p.N583S	missense	0	-	0	0.423:T	0	1	0	0	-
GNPTAB	12:102159031	T	G	NM_024312	c.1664A>C:p.Y555S	missense	-	-	-	0.976:D	0	0	0	1	-
GNPTAB	12:102159037	G	A	NM_024312	c.1658C>T:p.T553I	missense	-	-	-	0.891:D	0	0	0	2	-
GNPTAB	12:102159938	C	T	NM_024312	c.1543G>A:p.A515T	missense	0.0001	-	0.0003	0.782:D	0	1	0	1	-
GNPTAB	12:102160045	G	A	NM_024312	c.1436C>T:p.A479V	missense	-	-	-	0.330:T	0	1	0	0	-

GNPTAB	12:102161844	G	A	NM_024312	c.1379C>T:p.A460V	missense	-	-	-	0.877:D	0	1	0	1	-
GNPTAB	12:102161905	C	T	NM_024312	c.1318G>A:p.E440K	missense	-	-	-	0.899:D	0	0	1	0	-
GNPTAB	12:102163798	C	A	NM_024312	c.1284+1G>T	splicing	-	-	-	0.722:D	1	0	0	0	-
GNPTAB	12:102163842	T	G	NM_024312	c.1241A>C:p.D414A	missense	0	0	0	0.915:D	1	3	0	2	-
GNPTAB	12:102163902	C	A	NM_024312	c.1181G>T:p.R394L	missense	0.0002	-	-	0.980:D	1	0	0	2	-
GNPTAB	12:102164206	C	T	NM_024312	c.1091G>A:p.R364Q	missense	0.0008	-	0.0009	0.931:D	2	0	0	0	-
GNPTAB	12:102164207	G	A	NM_024312	c.1090C>T:p.R364X	stopgain	0.0001	-	-	0.688:T	1	2	2	0	P
GNPTAB	12:102164220	G	C	NM_024312	c.1077C>G:p.N359K	missense	-	-	-	0.788:D	1	0	0	0	-
GNPTAB	12:102164239	T	A	NM_024312	c.1058A>T:p.Q353L	missense	5.80E-05	-	-	0.917:D	2	2	0	1	-
GNPTAB	12:102164318	C	T	NM_024312	c.979G>A:p.E327K	missense	0	-	0	0.902:D	0	0	1	0	US
GNPTAB	12:102164333	G	T	NM_024312	c.964C>A:p.R322S	missense	-	-	-	0.956:D	0	0	1	0	-
GNPTAB	12:102164344	A	T	NM_024312	c.953T>A:p.I318N	missense	-	-	-	0.362:T	1	0	0	0	-
GNPTAB	12:102164833	C	T	NM_024312	c.874G>A:p.G292R	missense	5.80E-05	-	-	0.984:D	0	1	0	0	-
GNPTAB	12:102174027	T	C	NM_024312	c.674A>G:p.Q225R	missense	5.80E-05	-	-	0.377:T	1	0	0	0	-
GNPTAB	12:102174028	G	T	NM_024312	c.673C>A:p.Q225K	missense	5.80E-05	-	0.0001	0.586:T	0	1	2	0	-
GNPTAB	12:102174031	T	C	NM_024312	c.670A>G:p.M224V	missense	-	-	-	0.740:D	1	0	0	0	-
GNPTAB	12:102179792	T	C	NM_024312	c.569A>G:p.D190G	missense	0.0002	-	0.0001	0.365:T	1	2	1	1	-
GNPTAB	12:102179825	T	C	NM_024312	c.536A>G:p.N179S	missense	-	-	-	0.280:T	0	1	0	0	-
GNPTAB	12:102179825	T	A	NM_024312	c.536A>T:p.N179I	missense	-	-	-	0.690:T	0	0	1	0	-
GNPTAB	12:102179867	C	T	NM_024312	c.494G>A:p.S165N	missense	0	-	-	0.301:T	1	0	0	0	-
GNPTAB	12:102179909	A	C	NM_024312	c.452T>G:p.L151R	missense	0.0003	-	0.0002	0.962:D	2	1	2	0	US
GNPTAB	12:102179916	T	C	NM_024312	c.445A>G:p.I149V	missense	0	-	-	0.239:T	1	0	0	0	-
GNPTAB	12:102179930	G	T	NM_024312	c.431C>A:p.A144D	missense	-	-	-	0.849:D	1	3	0	0	-
GNPTAB	12:102183732	C	T	NM_024312	c.307G>A:p.E103K	missense	5.8E-05	0	-	0.830:D	0	0	0	1	-
GNPTAB	12:102183735	C	T	NM_024312	c.304G>A:p.E102K	missense	5.8E-05	-	-	0.684:T	0	0	0	1	-
GNPTAB	12:102183750	T	C	NM_024312	c.289A>G:p.R97G	missense	-	-	-	0.863:D	0	0	1	0	-
GNPTAB	12:102190455	C	T	NM_024312	c.203G>A:p.R68Q;-	missense	0.0003	-	0.0002	0.903:D	1	0	0	0	US
GNPTAB	12:102190456	G	A	NM_024312	c.202C>T:p.R68W;-	missense	0	-	0	0.950:D	0	1	0	0	-
GNPTAB	12:102190465	A	C	NM_024312	c.193T>G:p.F65V	missense	-	-	-	0.944:D	1	0	0	0	-
GNPTAB	12:102190492	A	G	NM_024312	c.166T>C:p.Y56H	missense	-	-	-	0.923:D	0	1	0	0	p.Y56X
GNPTAB	12:102224355	G	-	NM_024312	c.99delC:p.A34Pfs*48	frameshift deletion	-	-	-	-	0	2	0	0	P/LP

GNPTAB	12:102224365	GT	-	NM_024312	c.88_89del:p.T30Hfs*22	frameshift deletion	0.0001	-	0.0001	-	0	1	0	1	-
CLN5	13:77566138	C	T	NM_006493	c.52C>T:p.Q18X	stopgain	0	0	0	0.224:T	0	0	0	1	-
CLN5	13:77566139	A	C	NM_006493	c.53A>C:p.Q18P	missense	-	-	-	0.030:T	0	0	0	1	-
CLN5	13:77566160	T	C	NM_006493	c.74T>C:p.L25P	missense	0.0002	0.0006	-	0.021:T	0	0	1	0	US
CLN5	13:77566163	C	T	NM_006493	c.77C>T:p.A26V	missense	-	-	-	0.010:T	0	0	1	0	-
CLN5	13:77566202	C	T	NM_006493	c.116C>T:p.S39L	missense	0	-	0	0.032:T	0	0	1	0	-
CLN5	13:77566243	G	C	NM_006493	c.10G>C:p.E4Q	missense	-	-	-	0.088:T	1	0	0	1	-
CLN5	13:77566253	C	A	NM_006493	c.167C>A:p.T56K	missense	0	-	0	0.039:T	0	0	1	0	-
CLN5	13:77566325	C	A	NM_006493	c.10G>C:p.E4Q	missense	-	-	0	0.554:T	0	2	1	0	US
CLN5	13:77566375	C	T	NM_006493	c.289C>T:p.P97S	missense	-	-	-	0.016:T	0	0	1	0	-
CLN5	13:77566399	C	A	NM_006493	c.166C>A:p.P56T	missense	-	-	-	0.885:D	1	0	0	0	-
CLN5	13:77569200	G	A	NM_006493	c.176G>A:p.R59H	missense	0	0	0	0.823:D	1	0	0	0	US
CLN5	13:77569311	G	C	NM_006493	c.287G>C:p.R96P	missense	-	-	-	0.970:D	1	0	2	0	p.R96X
CLN5	13:77569350	T	C	NM_006493	c.326T>C:p.L109P	missense	-	-	-	0.994:D	1	1	2	0	-
CLN5	13:77570083	A	G	NM_006493	c.386A>G:p.K129R	missense	-	-	-	0.294:T	0	1	0	0	-
CLN5	13:77570121	G	C	NM_006493	c.424G>C:p.G142R	missense	0.0006	0.0006	0.0006	0.971:D	3	3	1	1	US
CLN5	13:77570202	G	A	NM_006493	c.652G>A:p.G218R	missense	-	0.0006	-	0.984:D	0	0	1	0	US
CLN5	13:77574606	C	A	NM_006493	c.579C>A:p.N193K	missense	0.0002	0	0	0.524:T	0	1	0	0	CIP
CLN5	13:77574689	G	A	NM_006493	c.662G>A:p.G221E	missense	0	0	0	0.361:T	0	1	0	0	US
CLN5	13:77574784	G	C	NM_006493	c.757G>C:p.E253Q	missense	5.80E-05	-	-	0.398:T	3	2	3	3	US
CLN5	13:77574800	G	A	NM_006493	c.773G>A:p.R258K	missense	0	-	0	0.300:T	0	1	1	0	US
CLN5	13:77574931	A	G	NM_006493	c.904A>G:p.K302E	missense	-	-	-	0.560:T	1	0	0	0	US
CLN5	13:77574962	T	C	NM_006493	c.935T>C:p.F312S	missense	0.0011	0.0012	0.0015	0.836:D	5	7	1	2	US
CLN5	13:77574991	T	C	NM_006493	c.1111T>C:p.Y371H	missense	-	-	-	0.970:D	0	0	1	0	-
CLN5	13:77574992	A	G	NM_006493	c.965A>G:p.Y322C	missense	-	-	-	0.972:D	1	1	0	0	-
CLN5	13:77575079	G	A	NM_006493	c.1199G>A:p.R400K	missense	-	-	-	0.029:T	0	0	1	0	-
CLN5	13:77575084	A	G	NM_006493	c.1204A>G:p.K402E	missense	0.0002	-	0.0005	0.055:T	0	0	0	1	-
NPC2	14:74946991	C	T	NM_006432	c.442G>A:p.V148I;-	missense	-	-	-	0.343:T	0	1	0	0	-
NPC2	14:74951129	C	T	NM_006432	c.352G>A:p.E118K	missense	0	-	0	0.680:T	0	0	0	1	US
NPC2	14:74951216	C	T	NM_006432	c.265G>A:p.E89K	missense	-	-	-	0.571:T	1	0	0	0	-
NPC2	14:74951264	C	T	NM_006432	c.217G>A:p.V73M	missense	0	-	-	0.440:T	1	0	1	0	-

NPC2	14:74951290	T	A	NM_006432	c.191A>T:p.N64I;-	missense	-	-	-	0.482:T	0	1	1	1	-
NPC2	14:74959975	C	A	NM_006432	c.3G>T:p.M1I	missense	0	-	0	0.928:D	0	0	1	0	LP
GALC	14:88401084	T	C	NM_000153	c.2050A>G:p.T684A	missense	5.80E-05	-	-	0.252:T	0	1	2	0	-
GALC	14:88401093	C	T	NM_000153	c.2041G>A:p.V681M	missense	0.0005	0.0006	0.0008	0.935:D	2	6	0	1	LP
GALC	14:88401120	C	G	NM_000153	c.2014G>C:p.E672Q	missense	0.0005	0.0013	0.0008	0.426:T	1	2	1	0	-
GALC	14:88401137	A	G	NM_000153	c.1997T>C:p.I666T	missense	5.80E-05	-	-	0.971:D	0	3	1	2	US
GALC	14:88401150	C	A	NM_000153	c.1984G>T:p.G662C	missense	-	-	-	0.990:D	0	0	1	0	-
GALC	14:88401202	C	T	NM_000153	c.1932G>A:p.M644I	missense	-	-	-	0.431:T	0	0	1	0	-
GALC	14:88401222	C	T	NM_000153	c.1912G>A:p.G638S	missense	0.0009	0.0006	0.0007	0.806:D	7	4	2	2	US
GALC	14:88406259	A	G	NM_000153	c.1901T>C:p.L634S	missense	0.0088	0.0057	0.009	0.996:D	36	35	33	20	CIP
GALC	14:88406299	G	A	NM_000153	c.1861C>T:p.R621C	missense	5.8E-05	0.0013	0	0.443:T	0	0	1	0	-
GALC	14:88407784	A	G	NM_000153	c.1789T>C:p.F597L	missense	-	-	-	0.931:D	0	0	1	0	-
GALC	14:88407816	C	T	NM_000153	c.1757G>A:p.G586D	missense	-	-	-	0.988:D	0	1	0	0	-
GALC	14:88407864	G	T	NM_000153	c.1709C>A:p.T570N	missense	0	-	0	0.378:T	0	0	1	0	US
GALC	14:88411900	T	C	NM_000153	c.1667A>G:p.N556S	missense	5.8E-05	-	-	0.246:T	0	0	1	0	-
GALC	14:88411963	T	C	NM_000153	c.1604A>G:p.N535S	missense	-	-	-	0.364:T	1	0	0	0	-
GALC	14:88411975	C	T	NM_000153	c.1592G>A:p.R531H	missense	0.0005	0	0.0007	0.961:D	2	1	2	2	P/LP
GALC	14:88411981	G	A	NM_000153	c.1586C>T:p.T529M	missense	0	0	0	0.944:D	0	2	0	0	P/LP
GALC	14:88411994	C	T	NM_000153	c.1573G>A:p.E525K	missense	0.0027	0.0045	0.0023	0.538:T	17	11	13	7	CIP
GALC	14:88412001	G	T	NM_000153	c.1566C>A:p.D522E	missense	5.8E-05	0	0.0001	0.723:D	0	0	0	1	-
GALC	14:88412015	T	C	NM_000153	c.1552A>G:p.T518A	missense	-	-	-	0.285:T	1	0	0	1	-
GALC	14:88414108	G	A	NM_000153	c.1453C>T:p.P485S	missense	0.0005	0.0019	0.0003	0.520:T	0	0	1	0	US
GALC	14:88414143	C	T	NM_000153	c.1418G>A:p.R473H	missense	0	-	0	0.245:T	1	1	1	1	US
GALC	14:88414181	T	A	NM_000153	c.1380A>T:p.E460D	missense	-	-	-	0.514:T	0	1	0	0	-
GALC	14:88416247	T	C	NM_000153	c.1280A>G:p.Y427C	missense	0.0005	0.0013	0.0002	0.950:D	8	1	6	1	US
GALC	14:88416268	A	G	NM_000153	c.1259T>C:p.I420T	missense	-	-	-	0.735:D	1	0	0	0	-
GALC	14:88417010	C	G	NM_000153	c.1244G>C:p.G415A	missense	-	-	-	0.943:D	0	0	1	0	-
GALC	14:88417046	T	C	NM_000153	c.1208A>G:p.N403S	missense	5.81E-05	0	0.0001	0.338:T	0	1	0	0	-
GALC	14:88417056	G	A	NM_000153	c.1198C>T:p.P400S	missense	-	-	-	0.662:T	0	1	0	1	-
GALC	14:88417068	G	A	NM_000153	c.1186C>T:p.R396W	missense	0	-	0	0.980:D	1	0	0	0	P
GALC	14:88417075	C	G	NM_000153	c.1179G>C:p.K393N	missense	-	-	-	0.157:T	1	0	0	0	-
GALC	14:88417081	A	C	NM_000153	c.1173T>G:p.H391Q	missense	-	-	-	0.292:T	0	1	0	0	-

GALC	14:88429780	T	A	NM_000153	c.1109A>T:p.Y370F	missense	0.0002	-	0.0001	0.592:T	1	1	0	2	-
GALC	14:88429807	A	C	NM_000153	c.1082T>G:p.V361G	missense	-	-	-	0.907:D	0	0	0	1	-
GALC	14:88429810	G	A	NM_000153	c.1079C>T:p.T360I	missense	5.80E-05	-	-	0.839:D	0	1	0	0	-
GALC	14:88429847	T	A	NM_000153	c.1042A>T:p.T348S	missense	5.8E-05	0	0	0.761:D	0	0	1	0	US
GALC	14:88429850	T	C	NM_000153	c.1039A>G:p.T347A	missense	-	-	-	0.839:D	1	0	0	0	-
GALC	14:88431861	C	G	NM_000153	c.1021G>C:p.V341L	missense	0.0002	0.0006	-	0.509:T	2	1	1	0	-
GALC	14:88431876	C	T	NM_000153	c.1006G>A:p.V336M	missense	0	0.0006	0	0.401:T	1	0	1	0	B/LB
GALC	14:88434685	A	T	NM_000153	c.902T>A:p.M301K	missense	-	-	-	0.988:D	0	0	1	0	-
GALC	14:88434697	A	T	NM_000153	c.890T>A:p.I297N	missense	0.0002	-	0.0002	0.761:D	0	0	0	1	-
GALC	14:88434701	A	G	NM_000153	c.886T>C:p.Y296H	missense	-	-	-	0.936:D	0	0	1	0	-
GALC	14:88434815	G	C	NM_000153	c.772C>G:p.H258D	missense	0	-	0	0.297:T	1	0	0	0	-
GALC	14:88442712	C	T	NM_000153	c.742G>A:p.D248N	missense	0.002	0.0012	0.0043	0.524:T	2	6	2	2	B
GALC	14:88442730	C	T	NM_000153	c.724G>A:p.E242K	missense	-	-	-	0.662:T	1	0	0	0	-
GALC	14:88442755	G	C	NM_000153	c.699C>G:p.I233M	missense	-	-	-	0.855:D	1	0	0	0	-
GALC	14:88450770	G	A	NM_000153	c.550C>T:p.R184C	missense	0.0021	0.0006	0.0028	0.463:T	3	7	10	5	B
GALC	14:88450776	C	T	NM_000153	c.544G>A:p.A182T	missense	0	0	-	0.852:D	0	0	1	0	-
GALC	14:88450820	T	C	NM_000153	c.500A>G:p.N167S	missense	0	-	0	0.381:T	0	0	1	0	US
GALC	14:88450836	C	T	NM_000153	c.484G>A:p.D162N	missense	0.0003	0.0006	0.0007	0.310:T	0	0	2	0	-
GALC	14:88450859	G	T	NM_000153	c.461C>A:p.P154H	missense	0.0002	-	0.0001	0.986:D	1	0	0	0	-
GALC	14:88452844	A	G	NM_000153	c.431T>C:p.I144T	missense	-	-	-	0.985:D	1	0	0	1	-
GALC	14:88452926	T	C	NM_000153	c.349A>G:p.M117V	missense	0	0	0	0.973:D	0	0	0	1	CIP
GALC	14:88454550	G	A	NM_000153	c.266C>T:p.P89L	missense	5.8E-05	0	0.0001	0.915:D	0	0	1	0	CIP
GALC	14:88454804	A	T	NM_000153	c.259T>A:p.F87I	missense	-	-	-	0.971:D	0	1	0	0	-
GALC	14:88454827	C	T	NM_000153	c.236G>A:p.R79H	missense	5.8E-05	-	0.0001	0.982:D	0	0	1	0	-
GALC	14:88454830	T	C	NM_000153	c.233A>G:p.Y78C	missense	0	-	0	0.853:D	1	0	0	0	US
GALC	14:88454869	-	A	NM_000153	c.196-2->T	splicing	0.0003	0	0.0002	-	0	0	0	2	-
GALC	14:88459373	C	A	NM_000153	c.136G>T:p.D46Y	missense	0.0006	-	0.0025	0.837:D	1	2	3	4	P
GALC	14:88459380	G	C	NM_000153	c.129C>G:p.Y43X	stopgain	-	0.0006	-	0.472:T	0	1	0	0	P/LP
GALC	14:88459416	C	G	NM_000153	c.93G>C:p.L31F	missense	-	-	-	0.472:T	1	0	0	0	-
GALC	14:88459432	C	T	NM_000153	c.77G>A:p.R26H	missense	-	-	-	0.392:T	0	0	1	0	-
GALC	14:88459444	C	A	NM_000153	c.65G>T:p.G22V	missense	-	-	-	0.713:D	0	0	1	0	-
GALC	14:88459448	C	G	NM_000153	c.61G>C:p.A21P	missense	0.0008	0.0012	0.0029	0.634:T	2	6	1	2	B

GALC	14:88459451	C	T	NM_000153	c.58G>A:p.A20T	missense	-	-	-	0.517:T	1	0	0	0	-
GALC	14:88459466	T	C	NM_000153	c.43A>G:p.K15E	missense	0	-	-	0.148:T	5	2	3	1	-
GALC	14:88459475	G	A	NM_000153	c.34C>T:p.R12C	missense	7.31E-05	-	-	0.470:T	1	3	1	2	-
GALC	14:88459481	A	G	NM_000153	c.28T>C:p.W10R	missense	0.0007	0.0006	0.0025	0.059:T	1	0	5	2	US
GALC	14:88459736	C	A	NM_001201402	c.108G>T:p.E36D	missense	9.98E-05	-	-	0.036:T	1	1	1	1	-
GALC	14:88459803	-	G	NM_001201402	c.40dupC:p.L14Pfs*12	frameshift insertion	-	-	-	-	0	1	0	0	US
GALC	14:88459813	G	C	NM_001201402	c.31C>G:p.H11D	missense	-	-	-	0.082:T	0	0	1	0	-
BLOC1S6	15:45879655	G	T	NM_012388	c.14G>T:p.G5V	missense	0	-	0	0.190:T	1	1	0	0	-
BLOC1S6	15:45879672	G	C	NM_012388	c.31G>C:p.G11R	missense	7.18E-05	-	0.0007	0.607:T	4	1	1	1	-
BLOC1S6	15:45879899	T	C	NM_001311255	c.2T>C:p.M1T	missense	0.0002	-	-	0.036:T	1	0	0	0	-
BLOC1S6	15:45879930	T	G	NM_001311255	c.33T>G:p.F11L	missense	0.0002	-	-	0.010:T	0	1	1	1	-
BLOC1S6	15:45879953	C	T	NM_001311255	c.56C>T:p.A19V	missense	0.0002	-	-	0.050:T	0	0	1	1	-
BLOC1S6	15:45884381	T	C	NM_001311255	c.146T>C:p.I49T	missense	0.0008	0	0.0008	0.035:T	3	2	0	2	US
BLOC1S6	15:45884405	T	A	NM_001311255	c.170T>A:p.L57Q	missense	-	-	-	0.992:D	0	0	1	0	-
BLOC1S6	15:45884474	C	A	NM_001311255	c.239C>A:p.T80K	missense	-	-	-	0.914:D	0	1	0	1	-
BLOC1S6	15:45897628	T	G	NM_001311256	c.242T>G:p.L81W	missense	0.0008	0.0018	0.0008	0.776:D	3	1	2	2	US
BLOC1S6	15:45898594	A	G	NM_001311255	c.416A>G:p.K139R	missense	-	-	-	0.434:T	0	1	0	1	-
BLOC1S6	15:45898653	C	T	NM_001311255	c.475C>T:p.R159X	stopgain	0.0001	-	0	0.710:D	0	1	0	0	-
MYO5A	15:52605952	C	T	NM_000259	c.5509G>A:p.A1837T	missense	0	0	0	0.555:T	0	0	0	1	-

MYO5A	15:52606014	T	C	NM_000259	c.5447A>G:p.Q1816R	missense	-	-	-	0.913:D	1	0	0	0	-
MYO5A	15:52606339	G	A	NM_000259	c.5396C>T:p.S1799L	missense	0	0	0	0.643:T	0	0	1	0	B
MYO5A	15:52609253	T	C	NM_000259	c.5326A>G:p.T1776A	missense	0.0018	0.0018	0.0017	0.766:D	11	3	4	3	-
MYO5A	15:52609280	T	C	NM_000259	c.5299A>G:p.I1767V	missense	5.8E-05	-	0.0001	0.716:D	0	0	1	0	-
MYO5A	15:52609382	T	G	NM_000259	c.5197A>C:p.K1733Q	missense	-	-	-	0.640:T	1	0	0	0	-
MYO5A	15:52609412	C	G	NM_000259	c.5167G>C:p.V1723L	missense	-	-	-	0.879:D	0	0	1	0	-
MYO5A	15:52611329	A	G	NM_000259	c.5087T>C:p.I1696T	missense	0	-	0	0.824:D	0	0	1	0	-
MYO5A	15:52611398	G	A	NM_000259	c.5018C>T:p.S1673L	missense	0.0002	0.0018	0.0002	0.854:D	0	0	3	1	LB
MYO5A	15:52611456	C	T	NM_000259	c.4960G>A:p.A1654T	missense	0	-	0	0.745:D	2	0	0	0	-
MYO5A	15:52613592	C	T	NM_000259	c.4840G>A:p.V1614M	missense	5.80E-05	0	0	0.461:T	0	1	0	0	-
MYO5A	15:52615589	C	T	NM_000259	c.4688G>A:p.R1563Q	missense	0	0	0	0.835:D	0	1	0	0	-
MYO5A	15:52615592	C	T	NM_000259	c.4685G>A:p.C1562Y	missense	-	-	-	0.919:D	1	0	0	0	-
MYO5A	15:52615611	A	G	NM_000259	c.4666T>C:p.F1556L	missense	-	-	-	0.791:D	1	0	0	0	-
MYO5A	15:52620132	G	A	NM_000259	c.4552C>T:p.R1518X	stopgain	0	-	-	0.637:T	0	1	0	1	-
MYO5A	15:52622651	G	T	NM_000259	c.4379C>A:p.P1460H	missense	-	-	-	0.646:T	0	1	0	0	-
MYO5A	15:52622660	A	G	NM_000259	c.4370T>C:p.I1457T	missense	5.8E-05	-	0	0.378:T	0	0	0	1	-
MYO5A	15:52628681	T	G	NM_000259	c.4312A>C:p.K1438Q	missense	-	-	-	0.533:T	0	0	0	1	-
MYO5A	15:52628719	G	A	NM_000259	c.4274C>T:p.T1425M	missense	0	0	0	0.551:T	0	1	0	0	-
MYO5A	15:52632498	G	T	NM_000259	c.4134C>A:p.N1378K	missense	0.0001	-	0.0003	0.476:T	0	0	0	1	-
MYO5A	15:52632522	C	A	NM_000259	c.4110G>T:p.E1370D	missense	-	-	-	0.618:T	0	1	0	0	-
MYO5A	15:52635394	T	A	NM_000259	c.3960A>T:p.R1320S;-	missense	0.0001	0	0.0002	0.451:T	0	2	0	0	B
MYO5A	15:52638600	C	A	NM_000259	c.3917G>T:p.G1306V	missense	-	-	-	0.522:T	1	0	1	0	-
MYO5A	15:52643525	G	A	NM_000259	c.3775C>T:p.L1259F	missense	-	-	-	0.664:T	1	0	0	1	-
MYO5A	15:52643563	C	T	NM_000259	c.3737G>A:p.R1246H	missense	0.0002	0	0.0001	0.318:T	4	1	1	0	-
MYO5A	15:52643570	C	T	NM_000259	c.3730G>A:p.A1244T	missense	0	-	-	0.443:T	0	0	1	0	-
MYO5A	15:52643585	C	T	NM_000259	c.3715G>A:p.A1239T	missense	0	-	0	0.493:T	0	1	0	1	-
MYO5A	15:52646081	C	T	NM_000259	c.3554G>A:p.R1185H	missense	0	-	0	0.433:T	0	1	0	0	-
MYO5A	15:52646103	G	A	NM_000259	c.3532C>T:p.R1178C	missense	0	-	0	0.236:T	1	0	0	0	-
MYO5A	15:52656819	G	C	NM_000259	c.3241C>G:p.L1081V	missense	0	-	0	0.568:T	0	1	0	0	-
MYO5A	15:52656880	T	A	NM_000259	c.3180A>T:p.L1060F	missense	-	-	-	0.354:T	0	0	1	0	-
MYO5A	15:52662437	G	T	NM_000259	c.2995C>A:p.L999M	missense	-	-	-	0.621:T	0	1	0	0	-
MYO5A	15:52662481	C	T	NM_000259	c.2951G>A:p.R984Q	missense	0	-	0	0.089:T	0	0	1	0	-

MYO5A	15:52662482	G	A	NM_000259	c.2950C>T:p.R984W	missense	0	-	-	0.471:T	0	0	0	1	-
MYO5A	15:52662604	T	C	NM_000259	c.2828A>G:p.Y943C	missense	0.0002	-	0.0004	0.454:T	0	2	1	1	-
MYO5A	15:52664389	G	A	NM_000259	c.2749C>T:p.R917C	missense	-	-	-	0.636:T	0	1	0	0	-
MYO5A	15:52664412	T	C	NM_000259	c.2726A>G:p.K909R	missense	0.0002	0.0006	0.0002	0.697:T	0	0	1	0	-
MYO5A	15:52664430	C	T	NM_000259	c.2708G>A:p.R903H	missense	5.8E-05	0.0006	0	0.875:D	0	0	0	1	-
MYO5A	15:52664445	C	T	NM_000259	c.2693G>A:p.R898Q	missense	0	0	0	0.888:D	0	1	0	0	-
MYO5A	15:52664502	C	T	NM_000259	c.2636G>A:p.R879H	missense	0.0001	0.0006	0.0001	0.761:D	1	0	0	0	-
MYO5A	15:52664523	C	T	NM_000259	c.2615G>A:p.R872Q	missense	5.82E-05	-	0	0.299:T	0	3	1	1	-
MYO5A	15:52664524	G	C	NM_000259	c.2614C>G:p.R872G	missense	5.82E-05	-	0.0001	0.259:T	0	1	2	6	-
MYO5A	15:52664526	T	A	NM_000259	c.2612A>T:p.K871M	missense	0.0017	0.0006	0.0005	0.834:D	9	8	6	4	-
MYO5A	15:52664554	G	C	NM_000259	c.2584C>G:p.R862G	missense	-	-	-	0.835:D	0	1	0	0	-
MYO5A	15:52667587	G	A	NM_000259	c.2491C>T:p.R831C	missense	0.0023	0.0025	0.0027	0.430:T	13	13	3	7	LB
MYO5A	15:52667599	T	A	NM_000259	c.2479A>T:p.M827L	missense	-	-	-	0.738:D	0	2	0	0	-
MYO5A	15:52667601	C	T	NM_000259	c.2477G>A:p.R826H	missense	0.004	0.0056	0.0026	0.855:D	14	9	15	6	CIP
MYO5A	15:52668596	G	A	NM_000259	c.2368C>T:p.R790W	missense	0	0	-	0.602:T	0	0	1	0	-
MYO5A	15:52668601	C	T	NM_000259	c.2363G>A:p.R788H	missense	5.82E-05	-	0	0.826:D	0	1	0	0	-
MYO5A	15:52668650	G	A	NM_000259	c.2314C>T:p.R772W	missense	0	0	0	0.579:T	0	1	0	1	-
MYO5A	15:52668673	T	A	NM_000259	c.2291A>T:p.D764V	missense	-	-	-	0.926:D	1	0	0	0	-
MYO5A	15:52668738	C	G	NM_000259	c.2226G>C:p.Q742H	missense	-	-	-	0.887:D	0	0	1	0	-
MYO5A	15:52675288	G	A	NM_000259	c.2012C>T:p.T671M	missense	0	0	0	0.518:T	0	0	0	1	-
MYO5A	15:52675352	G	A	NM_000259	c.1948C>T:p.L650F	missense	-	-	-	0.989:D	1	0	0	0	-
MYO5A	15:52676392	A	G	NM_000259	c.1880T>C:p.M627T	missense	0.018	0.0216	0.0176	0.207:T	60	53	52	35	B
MYO5A	15:52676396	G	T	NM_000259	c.1876C>A:p.Q626K	missense	0.0006	0.0012	0.0007	0.384:T	1	1	2	1	-
MYO5A	15:52676402	G	A	NM_000259	c.1870C>T:p.P624S	missense	0.001	-	0.0007	0.379:T	3	2	0	1	-
MYO5A	15:52676447	G	A	NM_000259	c.1825C>T:p.R609C	missense	0.0016	0.0012	0.0013	0.883:D	10	6	8	4	-
MYO5A	15:52680064	C	T	NM_000259	c.1714G>A:p.V572I	missense	0	-	0	0.733:D	0	0	1	0	-
MYO5A	15:52681467	T	C	NM_000259	c.1636A>G:p.K546E	missense	-	0.0006	-	0.611:T	2	3	3	4	-
MYO5A	15:52681478	C	T	NM_000259	c.1625G>A:p.R542H	missense	0	-	0	0.877:D	1	0	0	0	-
MYO5A	15:52684126	C	T	NM_000259	c.1542+1G>A	splicing	-	-	-	0.731:D	2	0	1	0	-
MYO5A	15:52689507	C	T	NM_000259	c.1210G>A:p.A404T	missense	0	0	-	0.867:D	1	0	0	0	-
MYO5A	15:52689527	G	A	NM_000259	c.1190C>T:p.T397I	missense	0	0.0006	0	0.539:T	0	2	0	0	-
MYO5A	15:52689555	T	C	NM_000259	c.1162A>G:p.I388V	missense	-	-	-	0.414:T	0	0	1	1	-

MYO5A	15:52689585	G	A	NM_000259	c.1132C>T:p.R378W	missense	0.0013	-	0.0016	0.907:D	3	1	2	2	-
MYO5A	15:52689587	T	C	NM_000259	c.1130A>G:p.H377R	missense	0	0	0.0001	0.873:D	0	2	0	1	-
MYO5A	15:52689614	T	C	NM_000259	c.1103A>G:p.Y368C	missense	0.0003	0.0006	0.0001	0.478:T	0	0	0	2	-
MYO5A	15:52697557	C	T	NM_000259	c.980G>A:p.R327Q	missense	5.81E-05	-	0.0002	0.392:T	1	2	0	0	-
MYO5A	15:52699558	T	C	NM_000259	c.877A>G:p.S293G	missense	0.0001	0.0012	0.0005	0.739:D	0	1	1	0	-
MYO5A	15:52708362	C	A	NM_000259	c.592G>T:p.A198S	missense	5.80E-05	-	0.0001	0.705:D	2	0	1	1	-
MYO5A	15:52708421	C	T	NM_000259	c.533G>A:p.R178Q	missense	-	-	-	0.922:D	1	0	0	0	-
MYO5A	15:52708473	C	T	NM_000259	c.481G>A:p.V161I	missense	0	-	0	0.862:D	1	0	0	0	-
MYO5A	15:52718082	C	A	NM_000259	c.400G>T:p.D134Y	missense	-	-	-	0.956:D	0	4	0	1	-
MYO5A	15:52725424	T	C	NM_000259	c.86A>G:p.K29R	missense	-	-	-	0.083:T	0	0	1	0	-
RAB27A	15:55497707	A	C	NM_004580	c.664T>G:p.X222G	stoploss	-	-	-	0.529:T	0	0	1	0	-
RAB27A	15:55497757	G	A	NM_004580	c.614C>T:p.A205V	missense	-	-	-	0.172:T	2	0	0	0	US
RAB27A	15:55497811	C	T	NM_004580	c.560G>A:p.R187Q	missense	0.007	0.0049	0.0062	0.523:T	14	5	11	7	CIP
RAB27A	15:55497812	G	A	NM_004580	c.559C>T:p.R187W	missense	0	0	0	0.782:D	0	1	0	1	US
RAB27A	15:55497820	C	T	NM_004580	c.551G>A:p.R184Q	missense	5.8E-05	0	0	0.513:T	0	0	1	1	US
RAB27A	15:55497848	C	T	NM_004580	c.523G>A:p.E175K	missense	-	-	-	0.579:T	1	0	1	1	-
RAB27A	15:55497861	TA	-	NM_004580	c.509_510del:p.I170Kfs*4	frameshift deletion	0.0003	0.0006	0.0002	-	1	0	0	0	-
RAB27A	15:55516105	G	A	NM_004580	c.449C>T:p.A150V	missense	-	-	-	0.426:T	1	0	0	1	-
RAB27A	15:55516171	A	G	NM_004580	c.383T>C:p.I128T	missense	0	0	0	0.866:D	1	0	0	0	US
RAB27A	15:55516177	G	A	NM_004580	c.377C>T:p.P126L	missense	-	-	-	0.758:D	1	1	0	1	-
RAB27A	15:55520871	C	T	NM_004580	c.279G>A:p.M93I	missense	-	-	-	0.641:T	0	0	0	1	-
RAB27A	15:55520890	G	A	NM_004580	c.260C>T:p.A87V	missense	-	-	0	0.662:T	0	0	0	1	-
RAB27A	15:55520896	G	A	NM_004580	c.254C>T:p.T85M	missense	0	-	0.0001	0.721:D	2	2	0	0	-
RAB27A	15:55526982	C	T	NM_004580	c.151G>A:p.V51M	missense	-	-	-	0.654:T	0	1	0	0	-
RAB27A	15:55527003	T	A	NM_004580	c.130A>T:p.I44F	missense	-	-	-	0.764:D	0	0	1	0	-
RAB27A	15:55527039	C	G	NM_004580	c.94G>C:p.G32R	missense	0.0002	-	0.0003	0.655:T	0	1	0	0	US
RAB27A	15:55527057	G	A	NM_004580	c.76C>T:p.L26F	missense	-	-	-	0.801:D	1	0	0	0	-
RAB27A	15:55527098	A	G	NM_004580	c.35T>C:p.F12S	missense	0.0002	-	-	0.927:D	0	3	0	1	-
RAB27A	15:55527122	C	A	NM_004580	c.11G>T:p.G4V	missense	0.0004	-	0.0009	0.592:T	4	4	4	1	US
CLN6	15:68500582	C	T	NM_017882	c.832G>A:p.A278T	missense	0	-	0	0.510:T	0	1	0	0	US
CLN6	15:68500585	C	G	NM_017882	c.829G>C:p.V277L	missense	-	-	-	0.867:D	0	0	0	1	-

CLN6	15:68500615	C	T	NM_017882	c.799G>A:p.A267T	missense	0	0	0	0.388:T	1	1	0	0	LB
CLN6	15:68500620	G	A	NM_017882	c.794C>T:p.S265F	missense	0	-	0	0.955:D	0	1	0	0	-
CLN6	15:68500665	C	G	NM_017882	c.749G>C:p.R250P	missense	-	-	-	0.957:D	0	1	1	1	-
CLN6	15:68500666	G	A	NM_017882	c.748C>T:p.R250C	missense	5.80E-05	0	-	0.892:D	1	0	0	0	-
CLN6	15:68500681	C	T	NM_017882	c.733G>A:p.V245I	missense	0	-	0	0.481:T	0	0	1	0	US
CLN6	15:68500696	C	T	NM_017882	c.718G>A:p.A240T	missense	5.80E-05	-	0.0001	0.822:D	1	0	0	0	US
CLN6	15:68501991	T	C	NM_017882	c.649A>G:p.S217G	missense	-	-	-	0.884:D	1	0	0	0	US
CLN6	15:68504027	T	G	NM_017882	c.472A>C:p.K158Q	missense	-	-	-	0.380:T	0	0	5	4	-
CLN6	15:68504054	G	A	NM_017882	c.445C>T:p.R149C	missense	0.0004	0.0006	0.0007	0.932:D	2	0	0	0	US
CLN6	15:68504093	G	A	NM_017882	c.406C>T:p.R136C	missense	-	-	-	0.952:D	1	0	0	1	US
CLN6	15:68504099	T	G	NM_017882	c.400A>C:p.N134H	missense	-	-	-	0.793:D	1	6	0	0	-
CLN6	15:68504102	C	T	NM_017882	c.397G>A:p.V133I	missense	-	-	-	0.403:T	0	0	0	1	-
CLN6	15:68504161	G	A	NM_017882	c.338C>T:p.T113M	missense	5.8E-05	-	0	0.349:T	0	0	2	0	US
CLN6	15:68504171	G	A	NM_017882	c.328C>T:p.R110C	missense	0.0005	0.0012	0.0006	0.865:D	3	0	1	0	US
CLN6	15:68504182	C	T	NM_017882	c.317G>A:p.R106H	missense	0.0003	-	0.0003	0.571:T	1	0	2	0	US
CLN6	15:68506670	G	C	NM_017882	c.255C>G:p.F85L	missense	5.80E-05	-	0.0001	0.810:D	0	1	0	0	US
CLN6	15:68506711	C	G	NM_017882	c.214G>C:p.E72Q	missense	0	0	0	0.797:D	1	0	0	0	B/LB
CLN6	15:68510953	G	T	NM_017882	c.119C>A:p.T40K	missense	0.0002	0.0006	0.0005	0.439:T	0	2	1	0	US
CLN6	15:68521840	C	G	NM_017882	c.83G>C:p.R28T	missense	-	-	-	0.704:D	0	0	0	1	p.R28K
CLN6	15:68521840	C	T	NM_017882	c.83G>A:p.R28K	missense	0	-	-	0.386:T	0	0	0	1	P
CLN6	15:68521847	G	A	NM_017882	c.76C>T:p.Q26X	stopgain	-	-	-	0.449:T	1	0	0	0	-
CLN6	15:68521859	C	T	NM_017882	c.64G>A:p.A22T	missense	0.0003	0	-	0.185:T	0	0	2	1	US
CLN6	15:68521900	T	G	NM_017882	c.23A>C:p.Q8P	missense	-	-	-	0.770:D	0	0	1	0	-
CLN6	15:68521909	C	A	NM_017882	c.14G>T:p.R5L	missense	-	-	-	0.771:D	1	0	0	0	-
CLN6	15:68521918	T	C	NM_017882	c.5A>G:p.E2G	missense	0.0069	0.0099	0.0154	0.704:D	51	30	36	19	CIP
HEXA	15:72636480	G	A	NM_000520	c.1528C>T:p.R510X	stopgain	-	-	-	0.697:T	0	0	1	0	P/LP
HEXA	15:72637878	C	T	NM_000520	c.1435G>A:p.A479T	missense	0.0082	0.0111	0.0091	0.855:D	46	36	34	23	CIP
HEXA	15:72638879	A	G	NM_000520	c.1319T>C:p.L440P	missense	0.0001	-	0.0002	0.888:D	0	3	0	0	US
HEXA	15:72638912	G	A	NM_000520	c.1286C>T:p.P429L	missense	-	-	-	0.653:T	1	0	0	0	-
HEXA	15:72638918	T	C	NM_000520	c.1280A>G:p.Y427C	missense	0	-	0	0.957:D	0	0	1	0	-
HEXA	15:72638923	T	C	NM_000520	c.1275A>G:p.I425M	missense	0.0001	-	0.0001	0.825:D	0	1	1	1	US
HEXA	15:72638925	T	C	NM_000520	c.1273A>G:p.I425V	missense	-	-	-	0.527:T	2	0	0	0	-

HEXA	15:72638927	C	T	NM_000520	c.1271G>A:p.R424H	missense	0	-	0	0.445:T	0	0	1	0	US
HEXA	15:72638943	G	C	NM_000520	c.1255C>G:p.P419A	missense	-	-	-	0.920:D	1	0	0	0	-
HEXA	15:72638961	G	A	NM_000520	c.1237C>T:p.R413W	missense	0.0011	0.0012	0.0006	0.844:D	7	3	4	3	US
HEXA	15:72638967	C	T	NM_000520	c.1231G>A:p.G411S	missense	0.0021	0.0019	0.0023	0.629:T	6	3	4	4	CIP
HEXA	15:72640032	C	T	NM_000520	c.1141G>A:p.V381I	missense	-	-	-	0.643:T	1	0	0	0	-
HEXA	15:72640037	T	C	NM_000520	c.1136A>G:p.N379S	missense	0	-	0	0.453:T	0	0	1	0	-
HEXA	15:72640059	CCACATAG	-	NM_000520	c.1107_1114del:p.Y370Vfs*5	frameshift deletion	-	-	-	-	1	0	0	0	-
HEXA	15:72640064	T	A	NM_000520	c.1109A>T:p.Y370F	missense	-	-	-	0.756:D	1	0	0	0	-
HEXA	15:72640064	T	-	NM_000520	c.1109delA:p.Y370Lfs*11	frameshift deletion	-	-	-	-	1	0	0	0	-
HEXA	15:72640086	C	T	NM_000520	c.1087G>A:p.V363I	missense	0.0002	-	0.0005	0.505:T	1	0	0	0	US
HEXA	15:72640089	T	C	NM_000520	c.1084A>G:p.I362V	missense	0.0005	-	0.0006	0.741:D	3	4	2	2	US
HEXA	15:72640394	G	C	NM_000520	c.1068C>G:p.I356M	missense	-	-	-	0.767:D	1	0	0	0	-
HEXA	15:72640429	C	T	NM_000520	c.1033G>A:p.G345S	missense	0	0	0	0.903:D	0	0	1	0	US
HEXA	15:72640453	C	T	NM_000520	c.1009G>A:p.D337N	missense	-	-	-	0.246:T	0	0	1	0	-
HEXA	15:72641513	T	C	NM_000520	c.893A>G:p.Y298C	missense	-	-	-	0.963:D	0	0	1	0	-
HEXA	15:72641570	G	A	NM_000520	c.836C>T:p.S279F	missense	-	-	-	0.662:T	0	1	0	0	-
HEXA	15:72642909	C	T	NM_000520	c.755G>A:p.R252H	missense	0	-	0	0.944:D	1	0	0	0	-
HEXA	15:72642919	G	A	NM_000520	c.745C>T:p.R249W	missense	0.0007	0.0012	0.0008	0.852:D	2	1	0	1	CIP
HEXA	15:72642936	A	G	NM_000520	c.728T>C:p.I243T	missense	-	-	-	0.902:D	1	0	0	0	-
HEXA	15:72642940	C	T	NM_000520	c.724G>A:p.V242I	missense	-	-	-	0.425:T	1	0	0	0	-
HEXA	15:72642963	T	C	NM_000520	c.701A>G:p.Y234C	missense	-	-	-	0.973:D	1	0	0	0	-
HEXA	15:72643541	T	G	NM_000520	c.605A>C:p.H202P	missense	-	-	-	0.856:D	0	0	1	0	-
HEXA	15:72643574	TCCTGTAGGT	-	NM_000520	c.571_572del:p.D191Cfs*5	frameshift deletion	-	-	-	-	1	0	0	0	-
HEXA	15:72645431	A	T	NM_000520	c.548T>A:p.L183H	missense	0.0053	0.0055	0.0054	0.802:D	30	24	28	18	LB
HEXA	15:72645432	-	T	NM_000520	c.546dupA:p.L183Tfs*2	frameshift insertion	-	-	-	-	0	0	0	1	P
HEXA	15:72648908	G	A	NM_000520	c.304C>T:p.P102S	missense	5.8E-05	-	-	0.410:T	0	0	0	1	US
HEXA	15:72648968	C	A	NM_001318825	c.277G>T:p.V93F	missense	-	-	-	0.238:T	0	1	0	0	-

HEXA	15:72668103	G	C	NM_000520	c.211C>G:p.L71V	missense	5.80E-05	-	-	0.530:T	2	1	0	0	-
HEXA	15:72668138	G	A	NM_000520	c.176C>T:p.S59L	missense	0	-	0	0.594:T	0	0	1	0	-
HEXA	15:72668145	C	A	NM_000520	c.169G>T:p.G57C	missense	-	-	-	0.846:D	0	0	1	0	-
HEXA	15:72668186	T	C	NM_000520	c.128A>G:p.N43S	missense	-	-	-	0.324:T	1	0	0	0	-
HEXA	15:72668304	A	G	NM_000520	c.10T>C:p.S4P	missense	0	0	0	0.737:D	0	0	0	1	US
GNPTG	16:1401971	C	T	NM_032520	c.5C>T:p.A2V	missense	0	0.0006	-	0.522:T	1	1	0	0	-
GNPTG	16:1401977	G	A	NM_032520	c.11G>A:p.G4E	missense	0	-	0	0.152:T	3	2	0	0	US
GNPTG	16:1401977	G	C	NM_032520	c.11G>C:p.G4A	missense	0	-	-	0.211:T	0	3	1	1	US
GNPTG	16:1402159	G	A	NM_032520	c.109G>A:p.G37R;-	missense	-	-	-	0.863:D	1	0	0	1	-
GNPTG	16:1411749	G	A	NM_032520	c.184G>A:p.V62M	missense	0	-	0	0.485:T	0	0	1	0	-
GNPTG	16:1411798	C	T	NM_032520	c.233C>T:p.T78M;-	missense	0.0006	0	0.0003	0.682:T	0	1	1	1	US
GNPTG	16:1411872	G	C	NM_032520	c.234-1G>C	splicing	-	-	-	0.672:T	0	1	1	1	LP
GNPTG	16:1411904	G	A	NM_032520	c.265G>A:p.V89M	missense	0	0	0	0.805:D	0	0	1	0	-
GNPTG	16:1411928	C	T	NM_032520	c.289C>T:p.R97C	missense	0	0	0	0.793:D	2	0	0	0	US
GNPTG	16:1411929	G	C	NM_032520	c.290G>C:p.R97P	missense	-	0	-	0.781:D	0	1	0	0	-
GNPTG	16:1411929	G	T	NM_032520	c.290G>T:p.R97L	missense	0	-	0	0.780:D	0	0	0	1	-
GNPTG	16:1411929	G	A	NM_032520	c.290G>A:p.R97H	missense	0	0.0006	0	0.723:D	0	0	1	0	-
GNPTG	16:1411943	A	G	NM_032520	c.304A>G:p.S102G	missense	0	0	0	0.762:D	1	0	0	0	-
GNPTG	16:1412060	C	G	NM_032520	c.339C>G:p.I113M	missense	0	-	0	0.378:T	0	1	0	1	-
GNPTG	16:1412065	A	G	NM_032520	c.344A>G:p.N115S	missense	5.8E-05	-	-	0.529:T	0	0	0	1	-
GNPTG	16:1412068	A	C	NM_032520	c.347A>C:p.N116T	missense	0.0011	0.0012	0.0009	0.514:T	2	1	1	2	-
GNPTG	16:1412094	G	A	NM_032520	c.373G>A:p.D125N	missense	0	-	-	0.341:T	0	0	0	1	-
GNPTG	16:1412110	G	A	NM_032520	c.389G>A:p.R130H	missense	0	-	0	0.528:T	0	0	1	0	-
GNPTG	16:1412220	G	A	NM_032520	c.425G>A:p.C142Y	missense	-	-	-	0.900:D	0	1	0	0	-
GNPTG	16:1412234	C	T	NM_032520	c.439C>T:p.R147W	missense	0	-	0	0.666:T	0	0	1	0	US
GNPTG	16:1412267	G	A	NM_032520	c.472G>A:p.V158I	missense	0	0	0	0.419:T	1	0	0	0	US
GNPTG	16:1412273	G	A	NM_032520	c.478G>A:p.A160T	missense	0	-	0	0.328:T	1	0	0	0	US
GNPTG	16:1412288	-	C	NM_032520	c.494dupC:p.L167Pfs*31	frameshift insertion	0	0	0	-	0	1	0	0	P
GNPTG	16:1412289	C	-	NM_032520	c.494delC:p.L167Sfs*8	frameshift deletion	0	-	0	-	0	1	0	1	-

GNPTG	16:1412297	G	A	NM_032520	c.502G>A:p.V168I	missense	0.006	0.0055	0.0054	0.636:T	31	29	20	25	LB
GNPTG	16:1412311	CG	-	NM_032520	c.516_517del:p.A173Lfs*24	frameshift deletion	-	-	-	-	1	0	0	0	-
GNPTG	16:1412311	C	A	NM_032520	c.516C>A:p.H172Q	missense	0.0001	-	0.0002	0.881:D	2	0	1	0	-
GNPTG	16:1412311	C	-	NM_032520	c.516delC:p.H172Qfs*3	frameshift deletion	-	-	-	-	1	0	0	0	-
GNPTG	16:1412312	G	A	NM_032520	c.517G>A:p.A173T	missense	0	-	0	0.328:T	2	0	0	0	-
GNPTG	16:1412312	G	-	NM_032520	c.517delG:p.A173Pfs*2	frameshift deletion	-	-	-	-	1	0	0	0	-
GNPTG	16:1412483	G	A	NM_032520	c.557G>A:p.R186Q	missense	5.8E-05	0	0	0.342:T	0	0	1	0	US
GNPTG	16:1412527	A	G	NM_032520	c.601A>G:p.T201A	missense	0.0002	0.0006	0.0001	0.889:D	0	1	0	0	-
GNPTG	16:1412649	C	T	NM_032520	c.647C>T:p.A216V	missense	0.0012	0.0019	0.001	0.823:D	3	2	3	2	-
GNPTG	16:1412662	G	T	NM_032520	c.660G>T:p.K220N	missense	-	-	-	0.348:T	0	0	1	0	-
GNPTG	16:1412695	G	C	NM_032520	c.693G>C:p.E231D	missense	-	-	-	0.565:T	0	1	0	0	-
GNPTG	16:1412715	G	A	NM_032520	c.713G>A:p.G238E	missense	0.0038	0.0055	0.0041	0.272:T	10	9	7	10	CIP
GNPTG	16:1412825	G	C	NM_032520	c.742-1G>C	splicing	-	-	-	0.609:T	0	1	0	0	CIP
GNPTG	16:1412883	G	A	NM_032520	c.799G>A:p.G267S	missense	0	0.0012	0	0.645:T	0	0	1	0	-
GNPTG	16:1412886	A	G	NM_032520	c.802A>G:p.I268V	missense	-	-	-	0.410:T	0	0	0	1	-
GNPTG	16:1412896	C	T	NM_032520	c.812C>T:p.T271M	missense	-	-	-	0.436:T	0	0	0	1	-
GNPTG	16:1413034	CCAGAGCCAAGTCT	-	NM_032520	c.860_873del:p.K290Afs*3	frameshift deletion	-	-	-	-	1	1	0	0	-
GNPTG	16:1413043	A	G	NM_032520	c.869A>G:p.K290R	missense	-	-	-	0.185:T	0	0	1	0	-
GNPTG	16:1413060	C	T	NM_032520	c.886C>T:p.R296W	missense	5.80E-05	0	0.0002	0.394:T	0	1	0	0	-
GNPTG	16:1413061	G	A	NM_032520	c.887G>A:p.R296Q	missense	5.8E-05	0	0	0.343:T	0	0	1	0	US
GNPTG	16:1413069	C	T	NM_032520	c.895C>T:p.P299S	missense	-	-	-	0.383:T	1	0	0	0	-
GNPTG	16:1413079	G	A	NM_032520	c.905G>A:p.R302H	missense	0.0001	-	0	0.350:T	0	0	0	1	-
CLN3	16:28488859	T	G	NM_001286105	c.995A>C:p.D332A	missense	-	-	-	0.531:T	0	0	2	0	-
CLN3	16:28488869	G	T	NM_001286105	c.985C>A:p.P329T	missense	0	-	-	0.742:D	0	0	0	1	-
CLN3	16:28488886	G	A	NM_001286105	c.968C>T:p.S323L	missense	0	-	0	0.678:T	0	1	0	0	-
CLN3	16:28488911	A	C	NM_001286105	c.943T>G:p.S315A	missense	0	-	-	0.362:T	0	1	0	1	-
CLN3	16:28488940	C	T	NM_001286105	c.914G>A:p.R305Q	missense	0	-	0	0.572:T	1	0	0	0	US

CLN3	16:28489158	C	T	NM_001286105	c.797G>A:p.G266D	missense	5.8E-05	-	0	0.440:T	0	0	1	0	-
CLN3	16:28489159	C	T	NM_001286105	c.796G>A:p.G266S	missense	0	-	-	0.239:T	1	0	0	0	-
CLN3	16:28489169	G	C	NM_001286105	c.786C>G:p.D262E	missense	0.0005	0.0006	0.0003	0.458:T	3	4	3	1	US
CLN3	16:28489191	T	C	NM_001286105	c.764A>G:p.N255S	missense	-	-	-	0.545:T	0	1	0	0	-
CLN3	16:28493439	A	C	NM_001286105	c.743T>G:p.L248R	missense	-	-	-	0.901:D	0	0	2	0	-
CLN3	16:28493460	C	T	NM_001286105	c.722G>A:p.R241H	missense	0.0025	0.0025	0.0027	0.501:T	26	15	24	10	LB
CLN3	16:28493469	C	T	NM_001286105	c.713G>A:p.R238H	missense	0.0003	-	0.0001	0.431:T	1	4	1	2	US
CLN3	16:28493469	C	G	NM_001286105	c.713G>C:p.R238P	missense	-	-	-	0.790:D	0	0	0	1	-
CLN3	16:28493470	G	A	NM_001286105	c.712C>T:p.R238C	missense	0.0003	0.0006	0.0003	0.626:T	2	0	1	1	US
CLN3	16:28493652	G	A	NM_001286105	c.658C>T:p.R220C	missense	0	-	0	0.762:D	0	0	1	0	US
CLN3	16:28493678	G	A	NM_001286105	c.632C>T:p.T211I	missense	-	-	-	0.442:T	1	0	0	0	-
CLN3	16:28493819	C	G	NM_001286105	c.585G>C:p.E195D	missense	-	-	-	0.980:D	1	0	0	0	-
CLN3	16:28493948	T	C	NM_001286105	c.536A>G:p.K179R	missense	0.0001	-	-	0.541:T	2	0	0	0	-
CLN3	16:28495344	G	A	NM_001286105	c.473C>T:p.A158V	missense	5.89E-05	-	0.0004	0.153:T	0	1	1	0	US
CLN3	16:28495429	A	T	NM_001286105	c.388T>A:p.L130M	missense	-	-	-	0.563:T	1	1	1	0	US
CLN3	16:28497706	C	-	NM_001286105	c.339delG:p.L114Cfs*39	frameshift deletion	-	-	-	-	1	1	1	2	-
CLN3	16:28497710	G	T	NM_001286105	c.335C>A:p.T112N	missense	5.81E-05	0.0006	-	0.740:D	2	0	0	0	-
CLN3	16:28497719	G	C	NM_001286105	c.326C>G:p.P109R	missense	-	-	-	0.945:D	0	0	2	0	-
CLN3	16:28497729	C	T	NM_001286105	c.316G>A:p.G106S	missense	0.0002	0.0012	0.0004	0.823:D	1	0	0	0	US
CLN3	16:28498858	G	-	NM_001286105	c.79delC:p.R27Gfs*53	frameshift deletion	5.80E-05	-	-	-	1	0	1	0	P/LP
CLN3	16:28498858	G	A	NM_001286105	c.79C>T:p.R27W	missense	0.0009	0.0012	0.001	0.993:D	1	1	1	1	US
CLN3	16:28498858	G	-	NM_001286105	c.79delC:p.R27Gfs*53	frameshift deletion	5.80E-05	-	-	-	1	0	1	0	P/LP
CLN3	16:28499964	G	A	NM_001286105	c.22C>T:p.R8X	stopgain	0.0001	0	0.0001	0.514:T	0	1	0	0	CIP
CLN3	16:28500673	C	T	NM_000086	c.160G>A:p.V54M	missense	-	-	-	0.969:D	0	1	0	0	-
CLN3	16:28502840	G	A	NM_000086	c.88C>T:p.H30Y	missense	-	-	-	0.201:T	0	0	1	0	-
CLN3	16:28503074	C	T	NM_000086	c.7G>A:p.G3S	missense	-	-	-	0.401:T	1	0	0	0	-
GALNS	16:88880849	A	C	NM_000512	c.1567T>G:p.X523E	stoploss	-	-	-	0.468:T	0	1	0	0	-
GALNS	16:88880926	G	T	NM_000512	c.1490C>A:p.A497E	missense	-	-	-	0.896:D	0	1	0	0	-
GALNS	16:88884414	C	T	NM_000512	c.1482+1G>A	splicing	-	-	-	0.628:T	1	0	0	0	-

GALNS	16:88884422	G	A	NM_000512	c.1475C>T:p.A492V	missense	0	0.0006	0	0.867:D	1	0	0	0	p.A492T
GALNS	16:88884438	T	A	NM_000512	c.1459A>T:p.N487Y	missense	-	-	-	0.941:D	0	4	1	0	-
GALNS	16:88884443	T	A	NM_000512	c.1454A>T:p.Q485L	missense	0.0001	-	-	0.872:D	0	0	0	1	-
GALNS	16:88884479	T	C	NM_000512	c.1418A>G:p.Q473R	missense	-	-	-	0.427:T	0	0	0	1	US
GALNS	16:88889054	G	A	NM_000512	c.1307C>T:p.T436M	missense	0	-	0	0.749:D	0	0	0	1	-
GALNS	16:88889106	A	C	NM_000512	c.1255T>G:p.C419G	missense	-	-	-	0.919:D	0	1	0	0	-
GALNS	16:88891176	T	C	NM_000512	c.1241A>G:p.Q414R	missense	-	-	-	0.424:T	0	0	1	0	p.Q414X
GALNS	16:88891219	C	T	NM_000512	c.1198G>A:p.A400T	missense	0.0005	0.0006	0.0005	0.899:D	10	6	10	2	US
GALNS	16:88891248	A	G	NM_000512	c.1169T>C:p.L390P	missense	-	-	-	0.957:D	0	0	1	0	-
GALNS	16:88891261	G	A	NM_000512	c.1156C>T:p.R386C	missense	0	0	0	0.970:D	1	0	0	0	P/LP
GALNS	16:88893122	C	T	NM_000512	c.1127G>A:p.R376Q	missense	0.0013	0.0012	0.001	0.782:D	2	4	4	0	CIP
GALNS	16:88893141	G	A	NM_000512	c.1108C>T:p.P370S	missense	0.0002	-	0.0004	0.906:D	1	0	0	0	US
GALNS	16:88893182	G	A	NM_000512	c.1067C>T:p.T356M	missense	0	-	-	0.433:T	0	0	2	0	US
GALNS	16:88893191	G	A	NM_000512	c.1058C>T:p.A353V	missense	0	0	0	0.852:D	0	0	1	0	-
GALNS	16:88898438	C	A	NM_000512	c.970G>T:p.A324S	missense	0	0	0	0.867:D	1	0	0	0	-
GALNS	16:88898456	T	A	NM_000512	c.952A>T:p.M318L	missense	0.001	0.0006	0.0008	0.549:T	2	3	0	0	US
GALNS	16:88898498	C	T	NM_000512	c.910G>A:p.G304S	missense	0	-	0	0.943:D	0	0	2	0	US
GALNS	16:88901632	G	A	NM_000512	c.887C>T:p.A296V	missense	0.0037	0.0049	0.0043	0.592:T	4	10	4	5	-
GALNS	16:88901662	G	A	NM_000512	c.857C>T:p.T286M	missense	0.0029	0.0025	0.0049	0.963:D	10	10	13	4	CIP
GALNS	16:88901725	A	G	NM_000512	c.794T>C:p.I265T	missense	-	-	-	0.821:D	0	1	0	0	-
GALNS	16:88901737	A	G	NM_000512	c.782T>C:p.I261T	missense	0.0001	0.0006	0.0001	0.743:D	0	0	0	1	-
GALNS	16:88902134	G	A	NM_000512	c.757C>T:p.R253W	missense	0	-	0.0001	0.927:D	0	0	1	0	US
GALNS	16:88902187	G	A	NM_000512	c.704C>T:p.T235M	missense	0	0.0006	0	0.953:D	0	0	1	0	-
GALNS	16:88902194	C	G	NM_000512	c.697G>C:p.D233H	missense	-	-	-	0.940:D	0	0	1	0	-
GALNS	16:88902199	G	C	NM_000512	c.692C>G:p.A231G	missense	0.0005	0.0012	0.0007	0.601:T	0	3	0	1	B
GALNS	16:88902253	G	A	NM_000512	c.638C>T:p.A213V	missense	0.0001	0.0006	0.0001	0.832:D	2	1	0	0	-
GALNS	16:88902619	A	G	NM_000512	c.623T>C:p.I208T	missense	0.0002	-	0.0001	0.609:T	0	0	1	0	-
GALNS	16:88902667	T	C	NM_000512	c.575A>G:p.E192G	missense	-	-	-	0.922:D	0	0	4	0	-
GALNS	16:88907455	G	C	NM_000512	c.367C>G:p.L123V	missense	-	-	-	0.429:T	1	0	0	0	-
GALNS	16:88907493	G	A	NM_000512	c.329C>T:p.P110L	missense	-	0	-	0.769:D	0	1	0	1	-
GALNS	16:88909177	G	A	NM_000512	c.181C>T:p.R61W	missense	0	0	0	0.868:D	1	0	0	0	LP

GALNS	16:88909189	G	A	NM_000512	c.169C>T:p.P57S	missense	0	0.0006	0	0.992:D	1	0	1	0	-
GALNS	16:88909207	C	T	NM_000512	c.151G>A:p.E51K	missense	0	-	-	0.798:D	0	0	1	0	-
GALNS	16:88909215	A	G	NM_000512	c.143T>C:p.V48A	missense	0.0041	0.0062	0.0042	0.541:T	5	7	4	7	-
GALNS	16:88909219	C	T	NM_000512	c.139G>A:p.G47R	missense	5.81E-05	-	0	1.000:D	0	1	0	0	P/LP
GALNS	16:88909387	CAGCATCGCCTGCGTGCGTGCACGATGGGGCCGCTCCACGCCAGCCCAAACCT	-	NM_001323544	c.137_138del:p.E46Dfs*4	frameshift deletion	-	0	-	-	1	0	0	0	-
GALNS	16:88909551	C	G	NM_001323544	c.25G>C:p.E9Q	missense	-	-	-	-	1	0	1	1	-
GALNS	16:88923198	G	A	NM_000512	c.88C>T:p.P30S	missense	9.23E-05	-	-	0.424:T	1	0	0	0	-
GALNS	16:88923218	C	G	NM_000512	c.68G>C:p.G23A	missense	-	-	-	0.444:T	0	1	0	0	-
CTNS	17:3540004	T	A	NM_001031681	UTR5	splicing	-	-	-	-	0	0	0	1	-
CTNS	17:3543508	G	A	NM_001031681	c.8G>A:p.R3K	missense	-	-	-	0.391:T	0	0	1	0	-
CTNS	17:3550791	T	C	NM_001031681	c.115T>C:p.S39P	missense	-	-	-	0.816:D	0	0	1	0	-
CTNS	17:3550798	A	C	NM_001031681	c.122A>C:p.N41T	missense	0	0	0	0.470:T	0	1	0	1	-
CTNS	17:3552185	T	G	NM_001031681	c.185T>G:p.F62C	missense	-	-	-	0.627:T	1	0	0	0	-
CTNS	17:3558293	T	C	NM_001031681	c.227T>C:p.V76A	missense	0	-	0	0.812:D	0	1	0	0	-
CTNS	17:3558525	C	T	NM_001031681	c.340C>T:p.R114C	missense	5.8E-05	-	0	0.587:T	0	0	1	0	-
CTNS	17:3558526	G	A	NM_001031681	c.341G>A:p.R114H	missense	5.80E-05	0	0	0.461:T	1	0	0	0	LB
CTNS	17:3558540	C	T	NM_001031681	c.355C>T:p.R119C	missense	0	-	0.0001	0.672:T	1	0	0	0	US
CTNS	17:3558541	G	A	NM_001031681	c.356G>A:p.R119H	missense	0.0008	0	0.0009	0.415:T	6	6	5	5	B
CTNS	17:3558549	G	A	NM_001031681	c.364G>A:p.A122T	missense	0.0027	0.0018	0.0018	0.315:T	9	7	6	5	B
CTNS	17:3558553	T	G	NM_001031681	c.368T>G:p.I123S	missense	0.0034	0.0031	0.0035	0.620:T	8	3	6	5	LB
CTNS	17:3558589	T	G	NM_001031681	c.404T>G:p.F135C	missense	0.0001	0.0006	0.0002	0.974:D	0	0	1	0	-
CTNS	17:3558600	T	G	NM_001031681	c.415T>G:p.S139A	missense	-	-	-	0.776:D	5	1	2	1	-
CTNS	17:3558615	C	G	NM_001031681	c.430C>G:p.P144A	missense	0	-	-	0.973:D	0	0	1	0	-
CTNS	17:3558627	A	T	NM_001031681	c.442A>T:p.M148L	missense	5.80E-05	0.0006	0.0001	0.403:T	6	5	0	5	-

CTNS	17:3558640	G	A	NM_001031681	c.455G>A:p.R152Q	missense	0	0.0006	0	0.947:D	0	4	1	1	-
CTNS	17:3559781	T	G	NM_001031681	c.462T>G:p.S154R	missense	0.0001	0.0006	0.0002	0.952:D	1	0	0	0	-
CTNS	17:3559800	G	A	NM_001031681	c.481G>A:p.D161N	missense	0.0003	0	0.0007	0.686:T	2	0	0	0	US
CTNS	17:3559997	G	A	NM_001031681	c.589G>A:p.G197R	missense	0.0003	0	0.0001	0.991:D	0	2	1	0	P/LP
CTNS	17:3560000	G	T	NM_001031681	c.592G>T:p.V198L	missense	-	-	-	0.831:D	0	1	0	2	-
CTNS	17:3560043	C	T	NM_001031681	c.635C>T:p.A212V	missense	0	0	0	0.922:D	0	0	1	0	US
CTNS	17:3560055	C	G	NM_001031681	c.647C>G:p.T216R	missense	-	-	-	0.949:D	1	0	0	0	-
CTNS	17:3560055	C	T	NM_001031681	c.647C>T:p.T216M	missense	0	-	-	0.846:D	0	1	0	0	-
CTNS	17:3560069	G	A	NM_001031681	c.661G>A:p.V221M	missense	0	0	0	0.288:T	1	0	0	0	US
CTNS	17:3561302	G	A	NM_001031681	c.685G>A:p.G229S	missense	0.0002	0	0	0.853:D	0	1	0	0	-
CTNS	17:3561303	G	A	NM_001031681	c.686G>A:p.G229D	missense	-	-	-	0.911:D	1	0	2	0	-
CTNS	17:3561312	G	A	NM_001031681	c.695G>A:p.R232H	missense	0	0	0	0.316:T	1	1	0	0	US
CTNS	17:3561332	G	A	NM_001031681	c.715G>A:p.G239S	missense	0	0	0	0.303:T	1	1	1	1	-
CTNS	17:3561354	T	C	NM_001031681	c.737T>C:p.L246P	missense	5.8E-05	-	-	0.924:D	0	0	1	0	-
CTNS	17:3561359	G	A	NM_001031681	c.742G>A:p.A248T	missense	0	0	0	0.308:T	1	0	0	0	-
CTNS	17:3561431	A	G	NM_001031681	c.814A>G:p.I272V	missense	0.0002	0.0006	0.0002	0.547:T	2	1	0	1	-
CTNS	17:3563228	G	A	NM_001031681	c.929G>A:p.S310N	missense	-	-	-	0.619:T	0	1	0	0	-
CTNS	17:3563263	A	G	NM_001031681	c.964A>G:p.N322D	missense	-	-	-	0.843:D	0	1	0	0	-
CTNS	17:3563535	T	C	NM_001031681	c.976T>C:p.W326R	missense	0.0003	0.0006	0.0001	0.994:D	0	0	1	0	-
CTNS	17:3563589	G	A	NM_001031681	c.1030G>A:p.V344I	missense	0	0	0	0.372:T	2	1	1	1	-
CTNS	17:3563601	G	A	NM_001031681	c.1042G>A:p.V348I	missense	5.8E-05	0.0006	0	0.359:T	0	0	2	1	-
CTNS	17:3563635	A	G	NM_001031681	c.1076A>G:p.K359R	missense	5.80E-05	-	-	0.389:T	0	1	0	0	-
CTNS	17:3563641	C	T	NM_001031681	c.1082C>T:p.P361L	missense	0.0002	-	0.0002	0.423:T	2	0	0	0	US
CTNS	17:3563922	C	T	NM_001031681	c.1097C>T:p.A366V	missense	5.80E-05	-	0.0001	0.524:T	0	1	0	0	-
CTNS	17:3563925	G	A	NM_001031681	c.1100G>A:p.R367H	missense	0.0036	0.0043	0.003	0.279:T	10	6	4	3	US
CTNS	17:3563942	C	T	NM_001031681	c.1117C>T:p.R373C	missense	0	-	-	0.331:T	0	1	0	0	-
CTNS	17:3563972	C	T	NM_001031681	c.1147C>T:p.Q383X	stopgain	-	0.0006	-	0.343:T	1	0	0	1	-
NAGLU	17:40688637	C	T	NM_000263	c.347C>T:p.A116V	missense	-	-	-	0.292:T	0	0	1	0	-
NAGLU	17:40689432	A	G	NM_000263	c.400A>G:p.N134D	missense	-	-	-	0.825:D	1	0	0	0	-
NAGLU	17:40689469	A	T	NM_000263	c.437A>T:p.D146V	missense	-	-	-	0.897:D	1	0	0	0	-
NAGLU	17:40689478	G	T	NM_000263	c.446G>T:p.R149L	missense	-	-	-	0.960:D	0	0	1	0	-

NAGLU	17:40689493	T	G	NM_000263	c.461T>G:p.I154R	missense	5.80E-05	-	0.0001	0.975:D	0	1	0	0	US
NAGLU	17:40690389	G	C	NM_000263	c.564G>C:p.E188D	missense	-	-	-	0.700:D	0	0	1	0	-
NAGLU	17:40690394	A	G	NM_000263	c.569A>G:p.N190S	missense	5.80E-05	-	0	0.375:T	2	0	0	1	US
NAGLU	17:40690401	C	A	NM_000263	c.576C>A:p.F192L	missense	0	-	0	0.662:T	1	0	0	0	-
NAGLU	17:40690437	G	T	NM_000263	c.612G>T:p.M204I	missense	0.0002	-	0	0.923:D	1	0	1	0	-
NAGLU	17:40690438	G	A	NM_000263	c.613G>A:p.G205S	missense	-	-	-	0.924:D	0	0	1	0	-
NAGLU	17:40690475	C	T	NM_000263	c.650C>T:p.S217F	missense	-	-	-	0.542:T	1	0	1	0	-
NAGLU	17:40690709	C	T	NM_000263	c.700C>T:p.R234C	missense	0	0.0012	0	0.953:D	0	0	0	1	P
NAGLU	17:40690727	C	G	NM_000263	c.718C>G:p.P240A	missense	-	-	-	0.569:T	0	1	0	0	-
NAGLU	17:40690746	C	T	NM_000263	c.737C>T:p.A246V	missense	5.81E-05	-	-	0.496:T	1	0	1	0	-
NAGLU	17:40690766	G	A	NM_000263	c.757G>A:p.V253I	missense	5.81E-05	-	0.0001	0.380:T	2	1	0	0	-
NAGLU	17:40692969	G	C	NM_000263	c.766G>C:p.V256L;-	missense	-	-	-	0.430:T	1	0	0	0	-
NAGLU	17:40692985	A	G	NM_000263	c.782A>G:p.N261S	missense	0.0002	-	0.0001	0.384:T	0	1	0	0	-
NAGLU	17:40692991	C	T	NM_000263	c.788C>T:p.T263M	missense	0	0	0	0.797:D	0	0	0	1	US
NAGLU	17:40693048	C	T	NM_000263	c.845C>T:p.A282V	missense	0	0	0	0.455:T	0	0	1	0	US
NAGLU	17:40693111	T	A	NM_000263	c.908T>A:p.F303Y	missense	5.8E-05	-	-	0.829:D	0	0	1	0	-
NAGLU	17:40693172	G	T	NM_000263	c.969G>T:p.E323D	missense	-	-	-	0.360:T	1	0	0	0	-
NAGLU	17:40693192	C	T	NM_000263	c.989C>T:p.A330V	missense	-	-	-	0.483:T	1	0	0	0	-
NAGLU	17:40693207	A	G	NM_000263	c.1004A>G:p.Y335C	missense	0	0	-	0.886:D	1	0	0	0	-
NAGLU	17:40693226	T	C	NM_000263	c.1021+2T>C	splicing	-	-	-	0.610:T	0	1	0	0	-
NAGLU	17:40695105	T	C	NM_000263	c.1081T>C:p.W361R	missense	0.0002	-	0.0002	0.999:D	0	0	1	0	-
NAGLU	17:40695144	C	T	NM_000263	c.1120C>T:p.P374S	missense	5.81E-05	-	0	0.828:D	0	1	0	0	-
NAGLU	17:40695147	C	T	NM_000263	c.1123C>T:p.R375C	missense	0	-	0	0.555:T	1	0	1	0	-
NAGLU	17:40695148	G	A	NM_000263	c.1124G>A:p.R375H	missense	0	-	0	0.460:T	0	0	0	1	-
NAGLU	17:40695196	A	G	NM_000263	c.1172A>G:p.Y391C	missense	-	-	-	0.875:D	0	0	1	0	-
NAGLU	17:40695318	C	G	NM_000263	c.1294C>G:p.L432V	missense	-	-	-	0.374:T	0	0	0	1	-
NAGLU	17:40695346	C	T	NM_000263	c.1322C>T:p.T441M	missense	0.0005	-	0.0005	0.753:D	2	3	6	1	CIP
NAGLU	17:40695562	A	G	NM_000263	c.1538A>G:p.N513S	missense	5.91E-05	-	0	0.542:T	1	1	1	0	-
NAGLU	17:40695565	G	A	NM_000263	c.1541G>A:p.R514H	missense	0.0004	-	0.0003	0.395:T	1	0	1	0	-
NAGLU	17:40695582	C	T	NM_000263	c.1558C>T:p.R520W	missense	0	-	-	0.965:D	0	0	1	0	LP
NAGLU	17:40695586	C	T	NM_000263	c.1562C>T:p.P521L	missense	0	0	0	0.958:D	0	0	1	0	P

NAGLU	17:40695621	C	T	NM_000263	c.1597C>T:p.R533X	stopgain	-	-	-	0.490:T	1	0	0	0	P/LP
NAGLU	17:40695664	C	T	NM_000263	c.1640C>T:p.A547V	missense	-	-	-	0.440:T	0	0	1	0	-
NAGLU	17:40695687	G	A	NM_000263	c.1663G>A:p.A555T	missense	0.0001	-	0.0001	0.261:T	0	1	0	0	-
NAGLU	17:40695699	G	T	NM_000263	c.1675G>T:p.D559Y	missense	-	-	-	0.994:D	1	0	0	0	-
NAGLU	17:40695727	T	G	NM_000263	c.1703T>G:p.V568G	missense	0.0006	-	0.0008	0.701:D	1	0	3	1	-
NAGLU	17:40695758	G	C	NM_000263	c.1734G>C:p.E578D	missense	-	-	-	0.381:T	0	0	0	1	-
NAGLU	17:40695807	G	A	NM_000263	c.1783G>A:p.G595R	missense	0.0002	-	0.0005	0.845:D	0	0	2	0	-
NAGLU	17:40695867	C	T	NM_000263	c.1843C>T:p.R615C	missense	0.0011	-	0.001	0.575:T	2	4	1	2	LB
NAGLU	17:40695930	G	A	NM_000263	c.1906G>A:p.D636N	missense	0.0004	0	0.0005	0.290:T	0	0	0	1	US
NAGLU	17:40695973	G	A	NM_000263	c.1949G>A:p.G650E	missense	0	-	-	0.999:D	0	0	1	0	P/LP
NAGLU	17:40696045	G	A	NM_000263	c.2021G>A:p.R674H	missense	0	-	0	0.989:D	0	0	0	1	-
NAGLU	17:40696083	G	A	NM_000263	c.2059G>A:p.A687T	missense	-	-	-	0.318:T	1	0	0	0	-
NAGLU	17:40696092	A	G	NM_000263	c.2068A>G:p.I690V	missense	-	-	-	0.331:T	0	0	0	1	-
NAGLU	17:40696149	G	A	NM_000263	c.2125G>A:p.V709I	missense	0	-	0	0.295:T	1	0	0	0	-
NAGLU	17:40696228	A	G	NM_000263	c.2204A>G:p.Y735C	missense	-	-	-	0.544:T	0	1	0	0	-
NAGLU	17:40696233	C	A	NM_000263	c.2209C>A:p.R737S	missense	0	0	0	0.470:T	0	1	1	0	B
NAGLU	17:40696242	G	C	NM_000263	c.2218G>C:p.A740P	missense	0.0006	0.0019	0.0008	0.518:T	2	2	0	3	-
NAGLU	17:40696242	G	A	NM_000263	c.2218G>A:p.A740T	missense	-	-	-	0.260:T	0	1	0	0	-
GRN	17:42426540	C	T	NM_002087	c.8C>T:p.T3I	missense	0	-	0	0.184:T	0	1	0	0	-
GRN	17:42426546	T	C	NM_002087	c.14T>C:p.V5A	missense	-	-	-	0.671:T	1	0	0	0	-
GRN	17:42426552	G	A	NM_002087	c.20G>A:p.W7X	stopgain	-	-	-	0.585:T	1	1	0	0	-
GRN	17:42426558	C	T	NM_002087	c.26C>T:p.A9V	missense	0	-	0	0.631:T	1	0	0	0	-
GRN	17:42426587	C	T	NM_002087	c.55C>T:p.R19W	missense	0	0	0	0.197:T	1	1	0	0	B
GRN	17:42426635	G	A	NM_002087	c.103G>A:p.G35R	missense	0	-	0	0.465:T	0	1	0	0	P
GRN	17:42426660	G	A	NM_002087	c.128G>A:p.R43H	missense	0.0002	0	0.0005	0.184:T	1	1	0	1	-
GRN	17:42426833	C	T	NM_002087	c.178C>T:p.P60S	missense	-	-	-	0.239:T	0	1	0	1	-
GRN	17:42426836	T	G	NM_002087	c.181T>G:p.C61G	missense	5.80E-05	-	-	0.848:D	1	0	0	0	-
GRN	17:42426854	T	C	NM_002087	c.199T>C:p.C67R	missense	-	-	0.0001	0.939:D	0	0	1	0	-
GRN	17:42426860	G	A	NM_002087	c.205G>A:p.A69T	missense	5.80E-05	-	-	0.104:T	2	0	0	0	-
GRN	17:42426909	C	T	NM_002087	c.254C>T:p.P85L	missense	-	-	-	0.886:D	0	0	1	0	-
GRN	17:42427038	G	A	NM_002087	c.268G>A:p.V90M	missense	5.80E-05	0	0	0.539:T	1	0	0	0	CIP

GRN	17:42427072	G	A	NM_002087	c.302G>A:p.R101Q	missense	0.0001	-	0.0001	0.159:T	5	0	5	5	US
GRN	17:42427099	G	A	NM_002087	c.329G>A:p.R110Q	missense	0.0001	0	0.0001	0.272:T	2	1	2	0	LB
GRN	17:42427607	G	A	NM_002087	c.361G>A:p.V121M	missense	0.0001	0.0006	0.0005	0.193:T	0	0	0	1	-
GRN	17:42427640	G	A	NM_002087	c.394G>A:p.E132K	missense	0	0	0	0.507:T	0	1	0	0	-
GRN	17:42427647	C	T	NM_002087	c.401C>T:p.P134L	missense	0	-	-	0.776:D	0	0	2	0	-
GRN	17:42427837	T	C	NM_002087	c.490T>C:p.C164R	missense	-	-	0.0001	0.952:D	0	0	0	1	-
GRN	17:42427861	G	A	NM_002087	c.514G>A:p.D172N	missense	5.80E-05	-	-	0.643:T	0	1	0	0	-
GRN	17:42427892	C	T	NM_002087	c.545C>T:p.T182M	missense	5.80E-05	0	0	0.377:T	1	0	0	1	B
GRN	17:42427921	C	T	NM_002087	c.574C>T:p.P192S	missense	-	-	-	0.690:T	0	0	0	1	-
GRN	17:42428077	T	C	NM_002087	c.617T>C:p.V206A	missense	0.0002	-	0.0002	0.580:T	2	5	2	4	-
GRN	17:42428091	G	A	NM_002087	c.631G>A:p.A211T	missense	5.8E-05	-	0	0.210:T	0	0	1	0	-
GRN	17:42428101	G	A	NM_002087	c.641G>A:p.R214Q	missense	0	-	-	0.362:T	1	0	0	0	US
GRN	17:42428122	G	C	NM_002087	c.662G>C:p.C221S	missense	0.0012	0.0025	0.0015	0.966:D	2	1	3	1	US
GRN	17:42428146	A	G	NM_002087	c.686A>G:p.Y229C	missense	-	-	-	0.790:D	1	0	0	1	-
GRN	17:42428168	C	A	NM_002087	c.708C>A:p.N236K	missense	5.8E-05	-	0.0001	0.300:T	0	0	2	0	-
GRN	17:42428169	G	A	NM_002087	c.708+1G>A	splicing	0	-	0	0.617:T	1	0	0	0	P/LP
GRN	17:42428441	C	G	NM_002087	c.745C>G:p.Q249E	missense	-	-	-	0.325:T	0	0	1	0	-
GRN	17:42428471	A	G	NM_002087	c.775A>G:p.K259E	missense	-	-	-	0.345:T	1	0	0	0	-
GRN	17:42428472	A	C	NM_002087	c.776A>C:p.K259T	missense	-	-	-	0.548:T	1	0	0	0	-
GRN	17:42428498	A	C	NM_002087	c.802A>C:p.T268P	missense	-	-	-	0.200:T	0	0	1	0	-
GRN	17:42428519	C	T	NM_002087	c.823C>T:p.P275S	missense	-	-	-	0.688:T	0	0	1	0	-
GRN	17:42428802	G	T	NM_002087	c.907G>T:p.A303S	missense	5.80E-05	-	-	0.216:T	1	0	0	0	-
GRN	17:42428955	C	G	NM_002087	c.971C>G:p.A324G	missense	-	-	-	0.313:T	0	0	1	0	-
GRN	17:42428973	C	T	NM_002087	c.989C>T:p.T330M	missense	0	0	0	0.519:T	0	1	1	1	-
GRN	17:42428994	AG	-	NM_002087	c.1010_1011del:p.Q337Rfs*28	frameshift deletion	-	-	-	-	0	1	0	0	LP
GRN	17:42429069	G	A	NM_002087	c.1085G>A:p.R362K	missense	-	-	-	0.076:T	0	1	0	0	-
GRN	17:42429122	C	G	NM_002087	c.1138C>G:p.Q380E	missense	0.0004	0.0012	0.0002	0.290:T	3	2	1	1	US
GRN	17:42429122	C	T	NM_002087	c.1138C>T:p.Q380X	stopgain	-	-	-	0.289:T	1	0	0	0	-
GRN	17:42429155	A	G	NM_002087	c.1171A>G:p.I391V	missense	-	-	-	0.172:T	0	0	1	0	-
GRN	17:42429432	G	A	NM_002087	c.1229G>A:p.C410Y	missense	0	-	-	0.949:D	1	0	0	0	-
GRN	17:42429441	A	C	NM_002087	c.1238A>C:p.E413A	missense	5.80E-05	-	-	0.295:T	2	4	3	1	-

GRN	17:42429498	G	A	NM_002087	c.1295G>A:p.R432H	missense	0	0	-	0.103:T	1	0	0	0	-
GRN	17:42429500	C	T	NM_002087	c.1297C>T:p.R433W	missense	0	0	0	0.448:T	1	0	0	0	CIP
GRN	17:42429530	G	A	NM_002087	c.1327G>A:p.G443S	missense	0.0006	0.0012	0.0002	0.342:T	1	3	2	3	-
GRN	17:42429555	C	T	NM_002087	c.1352C>T:p.P451L	missense	0	-	0	0.905:D	0	1	0	1	US
GRN	17:42429755	C	T	NM_002087	c.1460C>T:p.T487I	missense	0	0	0	0.804:D	1	0	0	0	-
GRN	17:42429845	A	G	NM_002087	c.1550A>G:p.K517R	missense	-	-	-	0.179:T	1	0	0	0	-
GRN	17:42429850	G	A	NM_002087	c.1555G>A:p.V519M	missense	0	-	0	0.678:T	1	1	0	1	US
GRN	17:42429898	C	T	NM_002087	c.1603C>T:p.R535X	stopgain	5.8E-05	-	0.0001	0.446:T	0	0	1	0	-
GRN	17:42430047	C	T	NM_002087	c.1663C>T:p.R555W	missense	0.0002	0.0012	0.0002	0.461:T	0	0	0	2	US
GRN	17:42430050	C	T	NM_002087	c.1666C>T:p.R556C	missense	0	-	-	0.656:T	0	0	1	0	-
GRN	17:42430063	C	T	NM_002087	c.1679C>T:p.P560L	missense	-	-	-	0.953:D	0	0	1	0	-
GRN	17:42430074	C	T	NM_002087	c.1690C>T:p.R564C	missense	0.0001	-	0.0001	0.650:T	3	0	3	1	-
GRN	17:42430075	G	A	NM_002087	c.1691G>A:p.R564H	missense	-	-	-	0.200:T	1	0	0	0	-
GRN	17:42430077	T	C	NM_002087	c.1693T>C:p.C565R	missense	0	-	-	0.906:D	0	0	1	0	-
GRN	17:42430090	G	A	NM_002087	c.1706G>A:p.G569D	missense	0.0002	-	0.0003	0.836:D	0	0	1	0	-
GRN	17:42430105	G	A	NM_002087	c.1721G>A:p.R574H	missense	0.0002	0	0.0003	0.131:T	0	0	1	0	US
GRN	17:42430119	C	T	NM_002087	c.1735C>T:p.R579C	missense	0	-	0	0.528:T	0	2	0	0	US
GAA	17:78078416	C	T	NM_000152	c.31C>T:p.R11W	missense	5.81E-05	0.0006	0.0001	0.403:T	0	1	2	1	-
GAA	17:78078417	G	A	NM_000152	c.32G>A:p.R11Q	missense	5.81E-05	0	0	0.361:T	1	1	0	0	-
GAA	17:78078428	G	A	NM_000152	c.43G>A:p.V15I	missense	0.0002	0.0006	0.0002	0.228:T	0	1	0	0	-
GAA	17:78078452	A	G	NM_000152	c.67A>G:p.T23A	missense	0.0016	0.0012	0.0016	0.452:T	6	9	11	9	-
GAA	17:78078459	C	T	NM_000152	c.74C>T:p.A25V	missense	0.0001	-	0.0001	0.360:T	3	5	3	2	-
GAA	17:78078602	G	A	NM_000152	c.217G>A:p.G73S	missense	0	0	0	0.257:T	0	0	1	0	-
GAA	17:78078626	C	T	NM_000152	c.241C>T:p.Q81X	stopgain	-	-	-	0.402:T	0	1	0	0	P
GAA	17:78078635	G	A	NM_000152	c.250G>A:p.V84I	missense	0	0	0	0.061:T	0	0	0	1	-
GAA	17:78078642	C	G	NM_000152	c.257C>G:p.P86R	missense	0.0076	0.0068	0.0074	0.590:T	33	20	16	16	-
GAA	17:78078645	A	G	NM_000152	c.260A>G:p.N87S	missense	0.0001	-	-	0.060:T	0	0	0	1	-
GAA	17:78078650	C	T	NM_000152	c.265C>T:p.R89C	missense	0	0	0	0.939:D	1	0	0	0	-
GAA	17:78078656	G	A	NM_000152	c.271G>A:p.D91N	missense	0.0002	0	0.0001	0.465:T	0	0	1	0	-
GAA	17:78078695	G	A	NM_000152	c.310G>A:p.E104K	missense	0	0	0	0.659:T	1	0	0	0	-
GAA	17:78078702	G	A	NM_000152	c.317G>A:p.R106H	missense	0.0001	-	0.0001	0.815:D	0	0	0	1	-

GAA	17:78078719	C	A	NM_000152	c.334C>A:p.P112T	missense	-	-	-	0.751:D	0	0	1	0	-
GAA	17:78078726	A	G	NM_000152	c.341A>G:p.K114R	missense	5.83E-05	-	0.0001	0.026:T	1	0	1	0	-
GAA	17:78078753	G	A	NM_000152	c.368G>A:p.G123E	missense	0	0	0.0001	0.542:T	0	0	0	1	-
GAA	17:78078805	C	A	NM_000152	c.420C>A:p.N140K	missense	0	0	0	0.346:T	0	1	0	1	-
GAA	17:78078888	G	A	NM_000152	c.503G>A:p.R168Q	missense	0.0015	0.0006	0.0018	0.701:D	2	2	3	0	-
GAA	17:78078918	G	A	NM_000152	c.533G>A:p.R178H	missense	0	0	0	0.827:D	0	1	0	1	-
GAA	17:78079552	A	G	NM_000152	c.551A>G:p.K184R	missense	-	-	-	0.440:T	1	2	1	2	-
GAA	17:78079635	G	A	NM_000152	c.634G>A:p.E212K	missense	0.0005	0.0006	0.0007	0.523:T	2	4	0	2	-
GAA	17:78079659	G	T	NM_000152	c.658G>T:p.V220L	missense	0.0008	0.0006	0.0001	0.383:T	1	2	1	1	US
GAA	17:78079665	G	A	NM_000152	c.664G>A:p.V222M	missense	5.8E-05	0.0006	0.0001	0.596:T	0	0	2	0	-
GAA	17:78079683	G	A	NM_000152	c.682G>A:p.G228S	missense	5.81E-05	-	0.0001	0.702:D	0	1	0	0	-
GAA	17:78081367	C	T	NM_000152	c.704C>T:p.T235M	missense	0	-	0	0.715:D	0	0	1	0	-
GAA	17:78081415	C	T	NM_000152	c.752C>T:p.S251L	missense	0.0028	0.0018	0.0029	0.350:T	1	5	7	1	-
GAA	17:78081424	C	T	NM_000152	c.761C>T:p.S254L	missense	0.0028	0.0019	0.0029	0.789:D	1	5	7	1	-
GAA	17:78081444	G	A	NM_000152	c.781G>A:p.A261T	missense	5.8E-05	0	0.0001	0.623:T	0	0	1	0	-
GAA	17:78081459	C	T	NM_000152	c.796C>T:p.P266S	missense	-	-	-	0.660:T	2	1	0	1	-
GAA	17:78081520	C	T	NM_000152	c.857C>T:p.T286M	missense	0	0	0	0.365:T	1	0	0	0	-
GAA	17:78081522	G	A	NM_000152	c.858+1G>A	splicing	-	-	-	0.628:T	0	0	1	0	-
GAA	17:78081668	G	A	NM_000152	c.928G>A:p.V310M	missense	-	-	-	0.909:D	1	0	0	0	-
GAA	17:78082104	C	T	NM_000152	c.971C>T:p.P324L	missense	0	0	0	0.907:D	0	1	0	0	-
GAA	17:78082181	G	A	NM_000152	c.1048G>A:p.V350M	missense	0	0	0	0.889:D	1	0	0	0	-
GAA	17:78082184	G	A	NM_000152	c.1051G>A:p.V351M	missense	-	-	-	0.780:D	0	0	1	0	-
GAA	17:78082202	G	A	NM_000152	c.1069G>A:p.V357I	missense	0.0002	-	0.0002	0.448:T	3	5	4	1	-
GAA	17:78082312	G	A	NM_000152	c.1100G>A:p.W367X	stopgain	0	-	-	0.812:D	0	0	1	0	-
GAA	17:78082359	A	G	NM_000152	c.1147A>G:p.I383V	missense	5.8E-05	-	-	0.443:T	0	0	0	1	-
GAA	17:78082398	T	A	NM_000152	c.1186T>A:p.F396I	missense	-	-	-	0.629:T	0	0	0	1	-
GAA	17:78082565	C	T	NM_000152	c.1264C>T:p.R422W	missense	5.96E-05	0	0.0002	0.387:T	1	0	0	0	-
GAA	17:78082566	G	A	NM_000152	c.1265G>A:p.R422Q	missense	0.0032	0.0043	0.0041	0.367:T	4	3	1	1	-
GAA	17:78082586	C	G	NM_000152	c.1285C>G:p.Q429E	missense	0	0	0	0.415:T	0	0	0	1	-
GAA	17:78082586	C	A	NM_000152	c.1285C>A:p.Q429K	missense	-	-	-	0.404:T	0	0	1	0	-
GAA	17:78082587	A	G	NM_000152	c.1286A>G:p.Q429R	missense	0.0105	0.008	0.0161	0.360:T	49	38	28	21	B

GAA	17:78082599	A	G	NM_000152	c.1298A>G:p.Q433R	missense	6.29E-05	0.0006	0	0.315:T	1	0	0	1	-
GAA	17:78082605	G	A	NM_000152	c.1304G>A:p.G435D	missense	-	-	-	0.966:D	2	1	0	1	-
GAA	17:78082607	C	T	NM_000152	c.1306C>T:p.R436W	missense	0.0004	-	0.0006	0.744:D	0	1	0	0	-
GAA	17:78083760	G	C	NM_000152	c.1343G>C:p.S448T	missense	0	0	0	0.422:T	0	0	0	1	-
GAA	17:78083792	G	A	NM_000152	c.1375G>A:p.D459N	missense	0	-	0	0.431:T	2	1	0	0	-
GAA	17:78083830	G	C	NM_000152	c.1413G>C:p.E471D	missense	-	-	-	0.179:T	0	2	1	0	-
GAA	17:78083849	G	A	NM_000152	c.1432G>A:p.G478R	missense	0	-	0	0.998:D	0	1	0	1	-
GAA	17:78084535	G	A	NM_000152	c.1447G>A:p.G483R	missense	0	-	0	0.976:D	0	1	0	0	US
GAA	17:78084541	A	G	NM_000152	c.1453A>G:p.T485A	missense	0	-	-	0.691:T	0	0	1	0	-
GAA	17:78084616	G	C	NM_000152	c.1528G>C:p.V510L	missense	-	-	-	0.893:D	0	0	1	0	-
GAA	17:78084628	G	A	NM_000152	c.1540G>A:p.G514S	missense	0	-	0	0.992:D	0	0	1	0	-
GAA	17:78084632	T	C	NM_000152	c.1544T>C:p.M515T	missense	0	-	-	0.812:D	0	0	0	1	-
GAA	17:78084782	G	A	NM_000152	c.1594G>A:p.G532S	missense	0.0001	-	0.0002	0.927:D	1	0	0	0	-
GAA	17:78085844	A	C	NM_000152	c.1699A>C:p.T567P	missense	-	-	-	0.847:D	1	0	1	0	-
GAA	17:78086379	C	T	NM_000152	c.1757C>T:p.A586V	missense	0.0002	0.0006	0.0001	0.917:D	2	2	1	0	-
GAA	17:78086443	C	-	NM_000152	c.1821delC:p.R608Dfs*87	frameshift deletion	-	-	-	-	0	1	0	0	-
GAA	17:78086465	G	A	NM_000152	c.1843G>A:p.G615R	missense	0.0002	-	0.0003	0.954:D	0	0	0	1	US
GAA	17:78086477	A	C	NM_000152	c.1855A>C:p.S619R	missense	-	-	-	0.948:D	0	1	0	0	-
GAA	17:78086495	G	A	NM_000152	c.1873G>A:p.A625T	missense	0	-	0	0.364:T	0	1	0	0	-
GAA	17:78086744	C	A	NM_000152	c.1958C>A:p.T653N	missense	0.0002	0.0006	0.0004	0.682:T	1	0	1	0	-
GAA	17:78086746	T	C	NM_000152	c.1960T>C:p.S654P	missense	-	-	-	0.884:D	0	1	0	0	-
GAA	17:78086773	C	T	NM_000152	c.1987C>T:p.Q663X	stopgain	-	-	-	0.845:D	0	0	0	1	-
GAA	17:78087027	C	A	NM_000152	c.2051C>A:p.P684Q	missense	-	-	-	0.965:D	0	1	0	0	-
GAA	17:78087054	A	G	NM_000152	c.2078A>G:p.Q693R	missense	6.32E-05	-	0.0002	0.192:T	1	0	0	0	-
GAA	17:78087080	C	T	NM_000152	c.2104C>T:p.R702C	missense	0	0	-	0.998:D	0	0	1	0	-
GAA	17:78087081	G	A	NM_000152	c.2105G>A:p.R702H	missense	0	0	0.0002	0.995:D	2	0	0	0	US
GAA	17:78087108	C	G	NM_000152	c.2132C>G:p.T711R	missense	0.0014	0	0.0019	0.968:D	12	3	11	2	-
GAA	17:78087131	G	A	NM_000152	c.2155G>A:p.A719T	missense	0	0	0	0.262:T	0	0	1	0	-
GAA	17:78087166	G	A	NM_000152	c.2189+1G>A	splicing	0	-	-	0.711:D	1	0	0	0	-
GAA	17:78090800	C	A	NM_000152	c.2223C>A:p.D741E	missense	-	-	-	0.849:D	1	0	0	0	-
GAA	17:78090813	T	G	NM_000152	c.2236T>G:p.W746G	missense	-	0.0006	-	0.996:D	1	1	2	1	-

GAA	17:78090814	G	C	NM_000152	c.2237G>C:p.W746S	missense	0	0	0	0.993:D	0	0	1	0	-
GAA	17:78090815	G	C	NM_000152	c.2238G>C:p.W746C	missense	0.0003	0	0.0002	0.988:D	2	1	2	0	US
GAA	17:78090816	G	A	NM_000152	c.2239G>A:p.G747R	missense	-	-	-	0.999:D	1	0	0	0	-
GAA	17:78091448	G	A	NM_000152	c.2381G>A:p.R794H	missense	0.0009	0.0019	0.0012	0.532:T	15	11	14	7	-
GAA	17:78091466	G	T	NM_000152	c.2399G>T:p.S800I	missense	-	-	-	0.830:D	0	1	0	1	-
GAA	17:78091470	G	C	NM_000152	c.2403G>C:p.E801D	missense	-	-	-	0.328:T	0	0	0	1	-
GAA	17:78091523	G	A	NM_000152	c.2456G>A:p.R819Q	missense	0	0	0	0.980:D	1	0	0	0	-
GAA	17:78092070	C	T	NM_000152	c.2560C>T:p.R854X	stopgain	0	0	0	0.379:T	0	1	0	0	P
GAA	17:78092091	G	A	NM_000152	c.2581G>A:p.D861N	missense	0	-	0	0.714:D	2	0	0	0	-
GAA	17:78092100	A	G	NM_000152	c.2590A>G:p.S864G	missense	-	-	-	0.541:T	1	0	0	0	-
GAA	17:78092101	G	T	NM_000152	c.2591G>T:p.S864I	missense	-	-	-	0.892:D	0	0	0	1	-
GAA	17:78092142	T	C	NM_000152	c.2632T>C:p.F878L	missense	-	-	-	0.896:D	0	1	0	0	-
GAA	17:78092151	A	G	NM_000152	c.2641A>G:p.R881G	missense	0.0008	0.0012	0.0005	0.238:T	2	1	1	0	-
GAA	17:78092461	G	C	NM_000152	c.2656G>C:p.V886L	missense	-	-	-	0.227:T	0	1	0	1	-
GAA	17:78092467	G	T	NM_000152	c.2662G>T:p.E888X	stopgain	0.0003	-	0.0002	0.307:T	1	0	0	1	P
GAA	17:78092473	G	C	NM_000152	c.2668G>C:p.V890L	missense	0.0005	0	0.0003	0.325:T	2	1	1	1	-
GAA	17:78092476	C	T	NM_000152	c.2671C>T:p.R891C	missense	0.0002	-	0.0001	0.476:T	0	0	1	0	-
GAA	17:78092530	G	A	NM_000152	c.2725G>A:p.V909M	missense	0	0	0	0.703:D	0	0	1	1	-
GAA	17:78092549	A	T	NM_000152	c.2744A>T:p.Q915L	missense	-	-	-	0.551:T	0	0	1	0	-
GAA	17:78092570	C	G	NM_000152	c.2765C>G:p.P922R	missense	-	-	-	0.559:T	0	1	0	0	-
GAA	17:78092570	C	T	NM_000152	c.2765C>T:p.P922L	missense	5.80E-05	-	-	0.522:T	1	0	0	0	-
GAA	17:78092585	C	T	NM_000152	c.2780C>T:p.T927I	missense	0.0007	0.0006	0.0009	0.406:T	0	1	3	1	-
GAA	17:78093083	TG	-	NM_000152	c.2812_2813del:p.V939Lfs*77	frameshift deletion	0.0002	-	0.0002	-	0	1	0	1	-
GAA	17:78093117	T	C	NM_000152	c.2846T>C:p.V949A	missense	-	-	-	0.466:T	1	0	0	0	-
SGSH	17:78184259	C	T	NM_000199	c.1501G>A:p.E501K	missense	-	-	-	0.565:T	0	1	0	0	-
SGSH	17:78184261	T	C	NM_000199	c.1499A>G:p.N500S	missense	5.89E-05	-	0.0002	0.467:T	3	0	1	0	US
SGSH	17:78184272	C	A	NM_000199	c.1488G>T:p.Q496H	missense	-	-	-	0.284:T	1	0	0	0	-
SGSH	17:78184277	A	G	NM_000199	c.1483T>C:p.C495R	missense	-	-	-	0.989:D	0	0	1	0	-
SGSH	17:78184298	C	T	NM_000199	c.1462G>A:p.E488K	missense	0	0	0	0.893:D	0	1	0	0	US
SGSH	17:78184310	C	T	NM_000199	c.1450G>A:p.D484N	missense	0.0007	-	0.0013	0.353:T	1	3	3	1	-
SGSH	17:78184384	T	C	NM_000199	c.1376A>G:p.Q459R	missense	-	-	-	0.255:T	1	0	0	0	-

SGSH	17:78184435	C	A	NM_000199	c.1325G>T:p.S442I	missense	-	-	-	0.309:T	0	1	0	0	-
SGSH	17:78184438	C	T	NM_000199	c.1322G>A:p.R441Q	missense	0	0	0	0.327:T	1	0	0	0	US
SGSH	17:78184439	G	A	NM_000199	c.1321C>T:p.R441W	missense	5.8E-05	0	0	0.430:T	0	0	1	0	US
SGSH	17:78184459	G	A	NM_000199	c.1301C>T:p.A434V	missense	5.80E-05	0	0	0.360:T	1	1	0	0	US
SGSH	17:78184463	G	A	NM_000199	c.1297C>T:p.R433W	missense	0	0	0	0.985:D	1	0	0	1	P/LP
SGSH	17:78184531	T	C	NM_000199	c.1229A>G:p.D410G	missense	5.80E-05	0.0006	0.0001	0.959:D	1	0	0	0	-
SGSH	17:78184550	C	T	NM_000199	c.1210G>A:p.V404I	missense	0.0003	-	0.0003	0.218:T	0	1	2	0	LB
SGSH	17:78184598	G	A	NM_000199	c.1162C>T:p.H388Y	missense	0.0001	-	0.0002	0.936:D	1	0	0	0	-
SGSH	17:78184601	C	T	NM_000199	c.1159G>A:p.V387M	missense	0.0001	0.0006	0.0001	0.301:T	0	0	2	0	B
SGSH	17:78184615	C	T	NM_000199	c.1145G>A:p.R382Q	missense	0	0	0	0.274:T	2	0	0	0	-
SGSH	17:78184679	C	T	NM_000199	c.1081G>A:p.V361I	missense	0.0002	0.0006	0.0002	0.325:T	1	0	2	0	B
SGSH	17:78184697	C	T	NM_000199	c.1063G>A:p.E355K	missense	0.0001	0	0.0002	0.978:D	1	1	0	0	CIP
SGSH	17:78184708	G	A	NM_000199	c.1052C>T:p.A351V	missense	0	0	-	0.463:T	1	0	0	0	-
SGSH	17:78184723	C	T	NM_000199	c.1037G>A:p.R346Q	missense	0	-	0	0.429:T	0	0	1	0	-
SGSH	17:78184747	G	A	NM_000199	c.1013C>T:p.S338L	missense	0	-	0	0.565:T	0	1	0	0	-
SGSH	17:78184762	T	C	NM_000199	c.998A>G:p.Y333C	missense	-	-	-	0.909:D	1	0	0	0	-
SGSH	17:78184801	G	A	NM_000199	c.959C>T:p.P320L	missense	-	-	-	0.994:D	0	1	0	0	-
SGSH	17:78184848	C	G	NM_001352921	c.1088G>C:p.X363S	stoploss	0.0001	0.0012	0.0001	-	3	2	0	0	-
SGSH	17:78184858	G	C	NM_001352921	c.1078C>G:p.P360A	missense	-	-	-	-	1	0	0	0	-
SGSH	17:78184869	G	A	NM_001352921	c.1067C>T:p.P356L	missense	-	0.0012	-	-	1	0	0	0	-
SGSH	17:78184881	C	T	NM_001352921	c.1055G>A:p.G352D	missense	-	-	-	-	2	0	0	0	-
SGSH	17:78185677	A	T	NM_001352922	c.995T>A:p.L332X	stopgain	-	-	-	0.176:T	0	1	0	0	-
SGSH	17:78185891	C	T	NM_000199	c.928G>A:p.E310K	missense	0	-	0	0.436:T	0	0	1	1	-
SGSH	17:78185906	A	C	NM_000199	c.913T>G:p.W305G	missense	-	-	-	0.916:D	0	0	0	1	-
SGSH	17:78185934	-	A	NM_000199	c.884dupT:p.V296Gfs*205	frameshift insertion	-	-	-	-	0	1	0	0	-
SGSH	17:78186005	A	G	NM_000199	c.814T>C:p.S272P	missense	-	-	-	0.999:D	0	1	0	0	-
SGSH	17:78186026	C	T	NM_000199	c.793G>A:p.D265N	missense	0	-	-	0.338:T	0	1	0	0	-
SGSH	17:78186041	C	T	NM_000199	c.778G>A:p.A260T	missense	0	-	0	0.764:D	1	0	0	0	-
SGSH	17:78186068	C	T	NM_000199	c.751G>A:p.G251R	missense	0.0002	-	0.0002	0.982:D	0	3	0	0	-
SGSH	17:78187618	C	T	NM_000199	c.730G>A:p.G244S	missense	6.14E-05	0	0	0.441:T	1	0	0	0	US

SGSH	17:78187645	C	T	NM_000199	c.703G>A:p.D235N	missense	0.0002	0	0.0002	0.663:T	0	1	2	0	P/LP
SGSH	17:78187651	G	A	NM_000199	c.697C>T:p.R233X	stopgain	0	0	0	0.642:T	1	0	0	0	P/LP
SGSH	17:78187659	G	A	NM_000199	c.689C>T:p.P230L	missense	0.0001	-	0.0002	0.539:T	0	0	3	1	US
SGSH	17:78187979	-	CA	NM_000199	c.654_655insTG:p.D219Wfs*45	frameshift insertion	-	-	-	-	0	0	0	1	-
SGSH	17:78188017	C	T	NM_000199	c.617G>A:p.R206H	missense	0	-	0	0.458:T	0	0	0	1	LP
SGSH	17:78188027	C	T	NM_000199	c.607G>A:p.G203S	missense	0	0	-	0.503:T	0	0	1	0	-
SGSH	17:78188036	C	T	NM_000199	c.598G>A:p.G200R	missense	0	-	0	0.497:T	0	1	0	0	-
SGSH	17:78188063	C	T	NM_000199	c.571G>A:p.G191R	missense	0	-	0	0.774:D	2	1	0	0	LP
SGSH	17:78188089	C	T	NM_000199	c.545G>A:p.R182H	missense	0.0001	-	0	0.936:D	2	1	0	0	p.R182C
SGSH	17:78188126	G	C	NM_000199	c.508C>G:p.P170A	missense	0.0005	0.0006	0.0002	0.944:D	2	0	2	1	-
SGSH	17:78188441	C	T	NM_000199	c.479G>A:p.R160Q	missense	0	-	0	0.587:T	1	1	0	1	-
SGSH	17:78188442	G	A	NM_000199	c.478C>T:p.R160W	missense	5.81E-05	-	0.0001	0.862:D	1	0	0	0	-
SGSH	17:78188465	A	C	NM_000199	c.455T>G:p.I152S	missense	5.80E-05	-	-	0.927:D	1	0	0	0	-
SGSH	17:78188517	C	G	NM_000199	c.403G>C:p.D135H	missense	-	-	-	0.605:T	0	1	0	0	p.D135X
SGSH	17:78188523	G	T	NM_000199	c.397C>A:p.P133T	missense	0.0032	0.0018	0.0031	0.950:D	4	3	9	2	LB
SGSH	17:78188525	T	C	NM_000199	c.395A>G:p.Y132C	missense	-	-	-	0.881:D	0	1	0	0	-
SGSH	17:78188529	C	T	NM_000199	c.391G>A:p.V131M	missense	5.8E-05	-	0.0001	0.940:D	0	0	1	0	-
SGSH	17:78188837	C	T	NM_000199	c.350G>A:p.R117H	missense	5.81E-05	0	0.0002	0.538:T	2	0	0	0	-
SGSH	17:78188843	C	A	NM_000199	c.344G>T:p.G115V	missense	0.0002	-	0.0001	0.438:T	2	0	3	0	-
SGSH	17:78188873	C	T	NM_000199	c.314G>A:p.R105Q	missense	5.81E-05	0	0.0001	0.429:T	0	2	0	1	-
SGSH	17:78188913	G	A	NM_000199	c.274C>T:p.H92Y	missense	-	-	-	0.889:D	2	0	0	0	-
SGSH	17:78190895	T	C	NM_000199	c.185A>G:p.N62S	missense	0	-	-	0.678:T	0	1	0	1	-
SGSH	17:78190898	C	T	NM_000199	c.182G>A:p.R61H	missense	6.20E-05	-	0.0002	0.409:T	1	0	0	0	-
SGSH	17:78190957	G	T	NM_000199	c.123C>A:p.N41K	missense	-	-	-	0.507:T	0	1	0	0	US
SGSH	17:78190965	C	T	NM_000199	c.115G>A:p.A39T	missense	0	-	0	0.414:T	1	1	0	0	-
SGSH	17:78194093	G	A	NM_000199	c.20C>T:p.A7V	missense	-	-	-	0.398:T	0	0	1	0	-
SGSH	17:78194111	A	C	NM_000199	c.2T>G:p.M1R	missense	-	-	-	0.898:D	0	0	5	5	-
NPC1	18:21112180	G	A	NM_000271	c.3823C>T:p.L1275F	missense	-	-	-	0.692:T	1	0	0	0	-
NPC1	18:21112186	C	T	NM_000271	c.3817G>A:p.E1273K	missense	-	0	-	0.727:D	0	0	1	0	-
NPC1	18:21112189	G	A	NM_000271	c.3814C>T:p.R1272C	missense	0.0002	0.0006	0.0002	0.813:D	3	2	4	1	US

NPC1	18:21112209	T	A	NM_000271	c.3794A>T:p.E1265V	missense	0.0002	0.0012	0.0005	0.332:T	0	1	0	1	US
NPC1	18:21112235	A	T	NM_000271	c.3768T>A:p.N1256K	missense	-	-	-	0.652:T	1	0	0	0	-
NPC1	18:21112244	-	G	NM_000271	c.3758dupC:p.S1254Ifs*3	frameshift insertion	-	-	-	-	0	0	1	0	-
NPC1	18:21113324	T	G	NM_000271	c.3749A>C:p.Y1250S	missense	-	-	-	0.977:D	0	1	0	0	-
NPC1	18:21113349	T	G	NM_000271	c.3724A>C:p.I1242L	missense	-	-	-	0.676:T	1	0	0	0	-
NPC1	18:21114427	C	T	NM_000271	c.3574G>A:p.A1192T	missense	0	-	-	0.317:T	0	1	0	0	-
NPC1	18:21114441	G	A	NM_000271	c.3560C>T:p.A1187V	missense	0	0	0.0001	0.917:D	0	1	0	0	-
NPC1	18:21114474	G	A	NM_000271	c.3527C>T:p.T1176M	missense	0.0004	0.0012	0.0002	0.485:T	5	0	3	3	US
NPC1	18:21114508	C	A	NM_000271	c.3493G>T:p.V1165L	missense	5.80E-05	-	0.0001	0.994:D	1	0	0	0	-
NPC1	18:21115431	A	G	NM_000271	c.3477+2T>C	splicing	0.0001	0.0012	0.0002	0.763:D	1	1	0	0	P
NPC1	18:21115473	C	T	NM_000271	c.3437G>A:p.G1146D	missense	-	-	-	0.776:D	0	1	0	0	-
NPC1	18:21115570	T	C	NM_000271	c.3340A>G:p.M1114V	missense	-	-	-	0.274:T	0	0	1	0	-
NPC1	18:21115603	C	T	NM_000271	c.3307G>A:p.G1103S	missense	-	0	-	0.295:T	0	0	0	1	-
NPC1	18:21116650	C	A	NM_000271	c.3232G>T:p.V1078L	missense	-	0.0006	-	0.770:D	0	0	0	1	-
NPC1	18:21116652	C	T	NM_000271	c.3230G>A:p.R1077Q	missense	0	-	0	0.800:D	0	0	1	0	US
NPC1	18:21116752	C	T	NM_000271	c.3130G>A:p.V1044M	missense	0.0006	0.0006	0.0006	0.684:T	1	3	1	1	US
NPC1	18:21116803	G	C	NM_000271	c.3079C>G:p.L1027V	missense	-	-	-	0.217:T	0	0	1	0	-
NPC1	18:21118573	C	G	NM_000271	c.2974G>C:p.G992R	missense	0	0	0	0.973:D	0	0	1	0	P/LP
NPC1	18:21118574	CT	-	NM_000271	c.2972_2973del:p.Q991Rfs*14	frameshift deletion	0	0	0	-	0	1	0	0	CIP
NPC1	18:21119397	C	T	NM_000271	c.2833G>A:p.D945N	missense	0	-	-	0.633:T	0	1	0	0	US
NPC1	18:21119794	C	T	NM_000271	c.2776G>A:p.A926T	missense	5.80E-05	-	0.0001	0.976:D	0	1	0	0	LP
NPC1	18:21119839	C	T	NM_000271	c.2731G>A:p.G911S	missense	0	0	0.0001	0.793:D	0	1	0	0	B
NPC1	18:21119874	T	A	NM_000271	c.2696A>T:p.Y899F	missense	-	-	-	0.861:D	0	0	1	0	-
NPC1	18:21120436	C	G	NM_000271	c.2580G>C:p.L860F	missense	-	-	-	0.896:D	1	1	0	1	-
NPC1	18:21120495	T	C	NM_000271	c.2521A>G:p.I841V	missense	0	-	0	0.314:T	3	0	0	0	-
NPC1	18:21121090	C	T	NM_000271	c.2456G>A:p.R819H	missense	0	0	0	0.272:T	0	1	1	1	-
NPC1	18:21121133	C	T	NM_000271	c.2413G>A:p.E805K	missense	0.0008	0.0018	0.0005	0.394:T	1	0	1	0	-
NPC1	18:21121138	C	G	NM_000271	c.2408G>C:p.G803A	missense	-	-	-	0.329:T	0	0	0	1	-
NPC1	18:21123436	G	A	NM_000271	c.2228C>T:p.T743I	missense	0.0002	0.0006	0.0001	0.775:D	1	0	0	0	-

NPC1	18:21123533	T	C	NM_000271	c.2131A>G:p.R711G;-	missense	0.0019	0.0121	0.003	0.985:D	2	0	1	1	US
NPC1	18:21124336	T	C	NM_000271	c.2102A>G:p.N701S	missense	-	-	-	0.807:D	0	1	0	0	-
NPC1	18:21124486	T	G	NM_000271	c.1952A>C:p.D651A	missense	5.80E-05	-	0.0002	0.985:D	4	0	0	1	-
NPC1	18:21125001	C	T	NM_000271	c.1870G>A:p.V624I	missense	0.0008	0	0.001	0.335:T	3	5	0	4	US
NPC1	18:21125027	C	T	NM_000271	c.1844G>A:p.R615H	missense	5.8E-05	-	-	0.883:D	0	0	0	1	-
NPC1	18:21125039	T	C	NM_000271	c.1832A>G:p.D611G	missense	-	-	-	0.978:D	0	1	0	0	P
NPC1	18:21125079	T	G	NM_000271	c.1792A>C:p.N598H	missense	5.8E-05	-	-	0.738:D	0	0	1	4	-
NPC1	18:21128024	T	C	NM_000271	c.1703A>G:p.N568S	missense	-	-	-	0.380:T	0	0	0	1	-
NPC1	18:21128067	T	C	NM_000271	c.1660A>G:p.N554D	missense	-	-	-	0.477:T	0	1	0	0	-
NPC1	18:21131609	C	T	NM_000271	c.1636G>A:p.V546M	missense	5.8E-05	-	-	0.845:D	0	0	1	0	-
NPC1	18:21131669	T	C	NM_000271	c.1576A>G:p.T526A	missense	0	-	0	0.814:D	1	0	0	0	-
NPC1	18:21131674	T	C	NM_000271	c.1571A>G:p.N524S	missense	-	-	-	0.505:T	0	1	0	0	-
NPC1	18:21131684	C	T	NM_000271	c.1561G>A:p.A521T	missense	0.0001	0.0006	0.0001	0.359:T	0	1	0	1	-
NPC1	18:21134726	C	T	NM_000271	c.1549G>A:p.V517I	missense	0.0007	-	0.0008	0.497:T	3	3	6	2	US
NPC1	18:21134753	C	T	NM_000271	c.1522G>A:p.D508N	missense	0	-	0	0.421:T	0	1	0	0	-
NPC1	18:21136225	G	C	NM_000271	c.1308C>G:p.D436E	missense	-	-	-	0.355:T	1	0	0	0	-
NPC1	18:21136254	C	T	NM_000271	c.1279G>A:p.A427T	missense	-	-	0.0001	0.315:T	0	1	1	0	-
NPC1	18:21136263	G	A	NM_000271	c.1270C>T:p.P424S	missense	0.0001	0.0006	-	0.759:D	0	1	0	0	-
NPC1	18:21136301	C	T	NM_000271	c.1232G>A:p.R411Q	missense	5.80E-05	0	0	0.308:T	0	1	1	0	B/LB
NPC1	18:21136304	A	G	NM_000271	c.1229T>C:p.I410T	missense	-	-	-	0.994:D	0	1	0	1	-
NPC1	18:21136319	G	A	NM_000271	c.1214C>T:p.T405M	missense	0	-	0	0.739:D	0	0	1	0	US
NPC1	18:21136322	C	T	NM_000271	c.1211G>A:p.R404Q	missense	0	-	0	0.942:D	0	1	0	0	P
NPC1	18:21136352	T	C	NM_000271	c.1181A>G:p.Y394C	missense	-	-	-	0.872:D	0	0	1	0	-
NPC1	18:21136401	C	T	NM_000271	c.1132G>A:p.V378I	missense	-	-	-	0.329:T	0	1	0	0	-
NPC1	18:21136494	C	T	NM_000271	c.1039G>A:p.V347I	missense	5.80E-05	0	0	0.416:T	1	0	0	0	US
NPC1	18:21136571	G	A	NM_000271	c.962C>T:p.A321V	missense	0.0003	0.0018	0.0004	0.233:T	5	4	1	2	US
NPC1	18:21137120	C	T	NM_000271	c.916G>A:p.D306N	missense	0	-	0	0.402:T	0	1	0	0	US
NPC1	18:21137128	G	A	NM_000271	c.908C>T:p.T303I	missense	-	-	-	0.612:T	0	1	0	0	-
NPC1	18:21137149	C	T	NM_000271	c.887G>A:p.R296Q	missense	0	0	0	0.498:T	2	0	2	1	US
NPC1	18:21140313	G	A	NM_000271	c.763C>T:p.P255S	missense	5.80E-05	-	0	0.516:T	1	0	0	0	-
NPC1	18:21140327	T	G	NM_000271	c.749A>C:p.K250T	missense	0.0002	-	0.0002	0.395:T	1	0	0	1	US

NPC1	18:21140406	T	C	NM_000271	c.670A>G:p.T224A	missense	-	-	-	0.838:D	2	1	0	1	-
NPC1	18:21140411	T	C	NM_000271	c.665A>G:p.N222S	missense	5.8E-05	0	0	0.464:T	0	0	1	0	CIP
NPC1	18:21141404	C	T	NM_000271	c.551G>A:p.C184Y	missense	-	-	-	0.992:D	1	0	0	0	US
NPC1	18:21141407	G	A	NM_000271	c.548C>T:p.A183V	missense	5.80E-05	-	0.0001	0.338:T	0	2	0	0	-
NPC1	18:21141414	C	T	NM_000271	c.541G>A:p.A181T	missense	0.0006	0.0012	0.0005	0.597:T	1	1	3	1	US
NPC1	18:21141452	C	A	NM_000271	c.503G>T:p.S168I	missense	-	-	-	0.918:D	1	0	0	0	-
NPC1	18:21141452	C	T	NM_000271	c.503G>A:p.S168N	missense	-	-	-	0.567:T	0	0	0	1	-
NPC1	18:21148810	T	C	NM_000271	c.440A>G:p.Y147C	missense	-	-	-	0.738:D	0	0	1	0	-
NPC1	18:21152111	T	G	NM_000271	c.214A>C:p.S72R	missense	-	-	-	0.513:T	0	0	1	0	-
NPC1	18:21152116	T	C	NM_000271	c.209A>G:p.N70S	missense	0.0001	-	0.0001	0.337:T	0	2	0	0	US
NPC1	18:21153486	T	C	NM_000271	c.110A>G:p.D37G	missense	0.001	0.0012	0.001	0.508:T	4	8	3	3	-
NPC1	18:21153502	C	T	NM_000271	c.94G>A:p.G32R	missense	0.0001	-	0.0001	0.896:D	1	0	0	0	-
NPC1	18:21166282	C	T	NM_000271	c.26G>A:p.G9D	missense	-	-	-	0.688:T	1	0	0	0	-
AP3D1	19:2102224	G	A	NM_001261826	c.3596C>T:p.T1199M	missense	0.0001	0	0.0002	0.370:T	0	2	0	3	-
AP3D1	19:2102255	C	A	NM_001261826	c.3565G>T:p.V1189F	missense	5.80E-05	-	-	0.702:D	2	0	0	0	-
AP3D1	19:2108726	C	T	NM_001261826	c.3512G>A:p.R1171H	missense	7.28E-05	-	0	0.658:T	1	0	0	0	-
AP3D1	19:2108757	G	C	NM_001261826	c.3481C>G:p.R1161G	missense	-	-	-	0.696:T	0	0	1	0	-
AP3D1	19:2109085	C	T	NM_001261826	c.3472G>A:p.V1158I;-	missense	0	0	0	0.356:T	1	0	2	0	-
AP3D1	19:2109114	G	A	NM_001261826	c.3443C>T:p.A1148V	missense	0	-	0	0.522:T	0	0	0	1	US
AP3D1	19:2109157	T	C	NM_001261826	c.3400A>G:p.I1134V	missense	0.0002	0	0.0001	0.009:T	1	4	0	1	US
AP3D1	19:2109202	C	T	NM_001261826	c.3355G>A:p.A1119T	missense	0	-	0	0.475:T	0	0	1	1	-
AP3D1	19:2109889	G	C	NM_001261826	c.3333C>G:p.I1111M	missense	-	-	-	0.279:T	0	1	0	1	-
AP3D1	19:2109930	G	C	NM_001261826	c.3292C>G:p.L1098V	missense	5.80E-05	-	-	0.676:T	1	1	0	0	-
AP3D1	19:2110709	G	A	NM_001261826	c.3172C>T:p.P1058S	missense	-	-	-	0.662:T	0	1	0	0	-
AP3D1	19:2110727	C	T	NM_001261826	c.3154G>A:p.V1052M	missense	0.0004	-	0.0012	0.640:T	1	2	0	0	-
AP3D1	19:2110739	C	T	NM_001261826	c.3142G>A:p.D1048N	missense	0	0	0	0.368:T	0	1	0	0	-
AP3D1	19:2110745	C	T	NM_001261826	c.3136G>A:p.V1046I	missense	0.0001	0	0.0003	0.298:T	1	0	0	0	-
AP3D1	19:2110750	G	A	NM_001261826	c.3131C>T:p.S1044F	missense	0	-	0	0.417:T	2	1	0	0	-
AP3D1	19:2110759	G	A	NM_001261826	c.3122C>T:p.P1041L	missense	0	-	0	0.773:D	0	1	1	0	-
AP3D1	19:2110762	C	T	NM_001261826	c.3119G>A:p.R1040Q	missense	0	-	0	0.675:T	0	1	0	0	-
AP3D1	19:2110763	G	A	NM_001261826	c.3118C>T:p.R1040W	missense	5.8E-05	0	0.0001	0.882:D	0	0	0	1	-

AP3D1	19:2111679	G	A	NM_001261826	c.2936C>T:p.P979L	missense	0	-	0	0.573:T	0	0	1	0	-
AP3D1	19:2111685	T	C	NM_001261826	c.2930A>G:p.Q977R	missense	-	-	-	0.085:T	1	0	0	0	-
AP3D1	19:2111692	C	T	NM_001261826	c.2923G>A:p.E975K	missense	-	-	-	0.384:T	0	1	0	0	-
AP3D1	19:2111701	C	T	NM_001261826	c.2914G>A:p.A972T	missense	0.0001	0	0.0003	0.031:T	0	1	0	1	-
AP3D1	19:2111710	G	C	NM_001261826	c.2905C>G:p.Q969E	missense	-	-	-	0.141:T	1	0	0	0	-
AP3D1	19:2111724	G	A	NM_001261826	c.2891C>T:p.A964V	missense	0.0023	0.0018	0.0036	0.158:T	5	8	6	6	-
AP3D1	19:2111725	C	T	NM_001261826	c.2890G>A:p.A964T	missense	6.14E-05	-	-	0.014:T	2	0	1	0	-
AP3D1	19:2111743	G	A	NM_001261826	c.2872C>T:p.P958S	missense	0.0001	-	0.0002	0.066:T	1	1	0	0	-
AP3D1	19:2111779	G	A	NM_001261826	c.2836C>T:p.R946W	missense	0.0002	0	0.0001	0.360:T	2	3	1	0	LB
AP3D1	19:2111803	T	G	NM_001261826	c.2812A>C:p.K938Q	missense	0.0001	0	0	0.339:T	1	2	0	2	LB
AP3D1	19:2111803	T	-	NM_001261826	c.2812delA:p.K938Sfs*57	frameshift deletion	-	-	-	-	2	0	0	0	-
AP3D1	19:2111823	G	T	NM_001261826	c.2792C>A:p.S931Y	missense	-	-	-	0.374:T	1	0	0	0	-
AP3D1	19:2112917	T	C	NM_001261826	c.2729A>G:p.D910G	missense	5.81E-05	-	0.0001	0.078:T	0	1	0	1	-
AP3D1	19:2113361	G	A	NM_001261826	c.2653C>T:p.P885S	missense	-	-	-	0.113:T	0	0	1	1	-
AP3D1	19:2113364	C	T	NM_001261826	c.2650G>A:p.A884T	missense	0	-	0	0.042:T	0	0	2	0	-
AP3D1	19:2113375	G	T	NM_001261826	c.2639C>A:p.P880Q	missense	-	-	-	0.060:T	1	0	0	0	-
AP3D1	19:2114125	T	A	NM_001261826	c.2600A>T:p.K867M	missense	9.28E-05	-	0	0.347:T	4	2	5	1	-
AP3D1	19:2114126	T	C	NM_001261826	c.2599A>G:p.K867E	missense	0.0002	-	0	0.200:T	4	2	5	1	-
AP3D1	19:2114129	T	C	NM_001261826	c.2596A>G:p.K866E	missense	-	-	-	0.154:T	1	0	0	0	-
AP3D1	19:2114141	T	C	NM_001261826	c.2584A>G:p.K862E	missense	8.96E-05	-	0	0.233:T	1	2	2	0	-
AP3D1	19:2114141	T	-	NM_001261826	c.2584delA:p.K862Rfs*36	frameshift deletion	-	-	-	-	0	1	0	0	-
AP3D1	19:2114192	T	C	NM_001261826	c.2533A>G:p.K845E	missense	0.0002	-	0.0007	0.424:T	0	1	2	1	-
AP3D1	19:2114792	T	C	NM_001261826	c.2378A>G:p.K793R	missense	0	-	-	0.140:T	1	0	0	0	-
AP3D1	19:2115282	G	A	NM_001261826	c.2285C>T:p.T762M	missense	0	0	0	0.605:T	1	0	0	0	-
AP3D1	19:2115291	G	A	NM_001261826	c.2276C>T:p.S759L	missense	0	0	0	0.435:T	0	1	1	0	-
AP3D1	19:2115295	T	C	NM_001261826	c.2272A>G:p.S758G	missense	-	-	-	0.057:T	1	0	0	0	-
AP3D1	19:2115300	C	T	NM_001261826	c.2267G>A:p.R756H	missense	0	0.0006	0	0.481:T	0	0	1	0	-
AP3D1	19:2115315	T	C	NM_001261826	c.2252A>G:p.K751R	missense	-	-	-	0.041:T	0	1	0	0	-
AP3D1	19:2115330	C	T	NM_001261826	c.2237G>A:p.R746K	missense	0	-	-	0.085:T	1	0	0	0	-
AP3D1	19:2115345	T	G	NM_001261826	c.2222A>C:p.K741T	missense	0.0001	-	-	0.392:T	0	1	0	1	-

AP3D1	19:2115373	G	A	NM_001261826	c.2194C>T:p.R732W	missense	0	-	0	0.588:T	0	1	0	0	-
AP3D1	19:2115573	C	T	NM_001261826	c.2113G>A:p.V705M	missense	-	-	-	0.542:T	1	0	0	0	-
AP3D1	19:2115576	C	T	NM_001261826	c.2110G>A:p.V704M	missense	0	-	0	0.683:T	1	0	0	0	-
AP3D1	19:2115599	G	A	NM_001261826	c.2087C>T:p.T696I	missense	5.8E-05	-	0.0001	0.131:T	0	0	2	0	-
AP3D1	19:2115602	T	C	NM_001261826	c.2084A>G:p.D695G	missense	-	-	-	0.535:T	0	0	2	0	-
AP3D1	19:2116250	C	A	NM_001261826	c.2029G>T:p.A677S	missense	-	-	-	0.432:T	1	0	0	0	-
AP3D1	19:2116605	C	T	NM_001261826	c.2000G>A:p.R667Q	missense	0.0002	0	0.0002	0.299:T	0	1	0	2	-
AP3D1	19:2116618	C	T	NM_001261826	c.1987G>A:p.E663K	missense	6.36E-05	-	-	0.221:T	0	1	0	0	-
AP3D1	19:2116638	C	T	NM_001261826	c.1967G>A:p.R656Q	missense	0	0	0	0.241:T	1	0	0	0	-
AP3D1	19:2116653	C	T	NM_001261826	c.1952G>A:p.R651Q	missense	6.1E-05	-	0	0.264:T	0	0	1	0	-
AP3D1	19:2116675	C	T	NM_001261826	c.1930G>A:p.V644I	missense	0.0015	0.0018	0.0015	0.033:T	6	4	6	1	-
AP3D1	19:2116695	T	C	NM_001261826	c.1910A>G:p.E637G	missense	0.0004	-	0.0004	0.739:D	5	4	3	2	-
AP3D1	19:2116719	T	G	NM_001261826	c.1886A>C:p.E629A	missense	0.0002	0.0006	-	0.552:T	3	5	4	2	-
AP3D1	19:2116732	C	T	NM_001261826	c.1873G>A:p.A625T	missense	0	0.0006	0	0.567:T	1	0	0	0	-
AP3D1	19:2117309	G	A	NM_001261826	c.1771C>T:p.P591S	missense	0.0002	-	0.0001	0.255:T	1	0	0	0	-
AP3D1	19:2117312	C	T	NM_001261826	c.1768G>A:p.V590M	missense	0.001	0.0012	0.0008	0.398:T	3	2	4	0	-
AP3D1	19:2117327	G	A	NM_001261826	c.1753C>T:p.L585F	missense	-	-	-	0.448:T	1	0	0	0	-
AP3D1	19:2118636	C	A	NM_001261826	c.1677G>T:p.Q559H	missense	5.81E-05	-	0.0001	0.249:T	1	0	0	0	-
AP3D1	19:2118646	C	T	NM_001261826	c.1667G>A:p.R556Q	missense	0.0009	0.0031	0.0008	0.663:T	1	0	0	0	-
AP3D1	19:2118677	C	T	NM_001261826	c.1636G>A:p.A546T	missense	5.80E-05	0	0.0001	0.494:T	0	1	0	1	-
AP3D1	19:2118692	C	T	NM_001261826	c.1621G>A:p.G541R	missense	0.0002	0	0.0001	0.166:T	1	4	1	0	-
AP3D1	19:2118722	C	T	NM_001261826	c.1591G>A:p.A531T	missense	0	-	0	0.583:T	1	0	0	0	-
AP3D1	19:2120919	G	A	NM_001261826	c.1423C>T:p.R475W	missense	5.81E-05	-	-	0.698:T	1	0	0	0	-
AP3D1	19:2120920	C	A	NM_001261826	c.1422G>T:p.Q474H	missense	0.0002	-	0.0004	0.476:T	1	0	0	0	-
AP3D1	19:2121166	C	T	NM_001261826	c.1246G>A:p.E416K	missense	5.80E-05	-	0.0001	0.569:T	1	0	1	0	US
AP3D1	19:2121247	C	T	NM_001261826	c.1165G>A:p.G389S	missense	-	-	-	0.649:T	0	0	1	0	-
AP3D1	19:2121749	T	C	NM_001261826	c.1085A>G:p.D362G	missense	0	-	0.0001	0.913:D	1	0	0	0	-
AP3D1	19:2121783	C	T	NM_001261826	c.1051G>A:p.D351N	missense	5.8E-05	-	0.0001	0.502:T	0	0	1	0	-
AP3D1	19:2129352	T	C	NM_001261826	c.697A>G:p.T233A	missense	-	-	-	0.688:T	0	0	1	0	-
AP3D1	19:2129358	A	T	NM_001261826	c.691T>A:p.S231T	missense	-	-	-	0.569:T	0	0	1	0	-
AP3D1	19:2129435	T	C	NM_001261826	c.614A>G:p.N205S	missense	0	0	0	0.433:T	0	0	1	0	-

AP3D1	19:2130411	G	T	NM_001261826	c.588C>A:p.D196E	missense	0.0002	-	0.0002	0.629:T	0	3	0	0	-
AP3D1	19:2130416	G	A	NM_001261826	c.583C>T:p.P195S	missense	0.0006	-	0.0005	0.692:T	1	2	2	1	-
AP3D1	19:2130449	C	A	NM_001261826	c.550G>T:p.A184S	missense	-	-	-	0.579:T	0	1	0	0	-
AP3D1	19:2132531	C	T	NM_001261826	c.401G>A:p.G134D	missense	-	-	-	0.732:D	1	0	0	0	-
MCOLN1	19:7587647	C	T	NM_020533	c.11C>T:p.P4L	missense	-	-	-	0.407:T	1	1	0	0	-
MCOLN1	19:7589867	C	A	NM_020533	c.52C>A:p.P18T	missense	5.8E-05	-	0.0001	0.492:T	0	0	0	1	-
MCOLN1	19:7589876	G	A	NM_020533	c.61G>A:p.G21R	missense	0	-	0	0.338:T	0	1	0	0	US
MCOLN1	19:7589912	C	T	NM_020533	c.97C>T:p.P33S	missense	0.0002	-	0.0002	0.211:T	2	2	0	3	-
MCOLN1	19:7589940	G	A	NM_020533	c.125G>A:p.R42H	missense	0	-	0	0.831:D	1	0	0	0	-
MCOLN1	19:7589988	C	T	NM_020533	c.173C>T:p.A58V	missense	-	-	-	0.608:T	0	0	1	0	-
MCOLN1	19:7591367	C	T	NM_020533	c.280C>T:p.R94W	missense	0	-	0	0.687:T	0	1	0	0	-
MCOLN1	19:7591385	G	A	NM_020533	c.298G>A:p.A100T	missense	0	-	0.0001	0.207:T	1	0	1	1	-
MCOLN1	19:7591424	G	A	NM_020533	c.337G>A:p.A113T	missense	-	-	-	0.107:T	0	1	0	0	LB
MCOLN1	19:7591439	G	A	NM_020533	c.352G>A:p.A118T	missense	0	-	0	0.692:T	0	1	0	1	-
MCOLN1	19:7591654	C	T	NM_020533	c.413C>T:p.A138V	missense	0	0	0	0.065:T	1	1	0	0	US
MCOLN1	19:7591660	C	T	NM_020533	c.419C>T:p.P140L	missense	-	-	-	0.626:T	0	1	0	0	-
MCOLN1	19:7591677	C	T	NM_020533	c.436C>T:p.R146W	missense	-	-	-	0.614:T	0	1	0	0	-
MCOLN1	19:7591734	C	G	NM_020533	c.493C>G:p.L165V	missense	-	-	-	0.415:T	0	0	1	0	-

MCOLN1	19:7591806	G	T	NM_020533	c.565G>T:p.V189F	missense	5.8E-05	-	-	0.353:T	0	0	1	0	-
MCOLN1	19:7592429	G	A	NM_020533	c.595G>A:p.E199K	missense	-	-	-	0.430:T	0	1	0	0	-
MCOLN1	19:7592432	C	T	NM_020533	c.598C>T:p.R200W	missense	0	-	0	0.434:T	0	1	0	1	-
MCOLN1	19:7592456	G	A	NM_020533	c.622G>A:p.D208N	missense	0.0001	0.0006	0.0001	0.237:T	5	1	1	0	-
MCOLN1	19:7592499	C	T	NM_020533	c.665C>T:p.T222M	missense	0	-	0	0.794:D	0	0	1	0	-
MCOLN1	19:7593048	C	T	NM_020533	c.782C>T:p.T261M	missense	0	0	0	0.347:T	1	0	0	0	CIP
MCOLN1	19:7593062	G	A	NM_020533	c.796G>A:p.A266T	missense	-	-	-	0.789:D	0	0	1	0	-
MCOLN1	19:7593487	C	A	NM_020533	c.882C>A:p.D294E	missense	-	-	-	0.307:T	0	0	0	2	-
MCOLN1	19:7593512	G	A	NM_020533	c.907G>A:p.V303M	missense	0	-	0	0.326:T	1	0	0	0	-
MCOLN1	19:7593578	C	G	NM_020533	c.973C>G:p.L325V	missense	-	-	-	0.357:T	0	0	1	0	-
MCOLN1	19:7593714	T	G	NM_020533	c.992T>G:p.V331G	missense	0.0002	0.0006	0.0003	0.593:T	3	0	0	1	-
MCOLN1	19:7593723	T	C	NM_020533	c.1001T>C:p.M334T	missense	0	0	0	0.383:T	0	1	0	1	-
MCOLN1	19:7593728	C	T	NM_020533	c.1006C>T:p.R336W	missense	-	-	-	0.668:T	0	1	0	0	US
MCOLN1	19:7593740	C	T	NM_020533	c.1018C>T:p.R340W	missense	0.0001	0.0006	0.0001	0.637:T	1	1	0	0	-
MCOLN1	19:7593790	C	G	NM_020533	c.1068C>G:p.I356M	missense	-	-	-	0.852:D	0	1	0	1	-
MCOLN1	19:7593797	G	A	NM_020533	c.1075G>A:p.V359I	missense	0	0	0	0.351:T	0	1	0	0	-

MCOLN1	19:7594087	A	G	NM_020533	c.1235A>G:p.N412S	missense	-	-	-	0.621:T	0	0	1	0	-
MCOLN1	19:7594486	C	T	NM_020533	c.1247C>T:p.A416V	missense	0.0009	0.0006	0.0009	0.380:T	0	1	0	0	-
MCOLN1	19:7594495	G	A	NM_020533	c.1256G>A:p.R419Q	missense	0	-	0	0.672:T	0	0	1	0	-
MCOLN1	19:7594518	C	T	NM_020533	c.1279C>T:p.R427C	missense	0	0	0	0.937:D	1	0	0	0	US
MCOLN1	19:7594533	G	A	NM_020533	c.1294G>A:p.V432M	missense	-	-	-	0.884:D	1	0	0	0	-
MCOLN1	19:7595175	C	T	NM_020533	c.1363C>T:p.R455C	missense	0	-	0	0.953:D	0	0	1	0	-
MCOLN1	19:7595236	T	A	NM_020533	c.1424T>A:p.V475E	missense	-	-	-	0.854:D	1	0	0	0	-
MCOLN1	19:7595239	C	T	NM_020533	c.1427C>T:p.T476M	missense	-	-	-	0.974:D	0	1	0	0	-
MCOLN1	19:7595269	G	A	NM_020533	c.1457G>A:p.R486H	missense	0.0001	0	0.0001	0.206:T	1	1	2	0	-
MCOLN1	19:7595355	G	A	NM_020533	c.1543G>A:p.A515T	missense	0	-	-	0.900:D	0	0	1	0	-
MCOLN1	19:7598425	G	C	NM_020533	c.1592G>C:p.G531A	missense	-	-	-	0.303:T	0	1	0	0	-
MCOLN1	19:7598533	G	A	NM_020533	c.1700G>A:p.C567Y	missense	5.82E-05	-	0.0001	0.952:D	1	0	2	0	-
MAN2B1	19:12757477	C	T	NM_000528	c.2993G>A:p.R998H	missense	0.0119	0.0112	0.0124	0.865:D	16	13	13	10	B
MAN2B1	19:12757498	G	C	NM_000528	c.2972C>G:p.T991R	missense	-	-	-	0.717:D	0	0	1	0	-
MAN2B1	19:12757510	G	A	NM_000528	c.2960C>T:p.P987L	missense	0	0	0	0.377:T	1	0	0	0	-
MAN2B1	19:12757514	C	G	NM_000528	c.2956G>C:p.D986H	missense	0.0003	-	0.0003	0.492:T	1	0	0	0	-
MAN2B1	19:12757523	A	G	NM_000528	c.2947T>C:p.Y983H	missense	-	-	-	0.209:T	0	0	1	0	-
MAN2B1	19:12758062	A	C	NM_000528	c.2908T>G:p.W970G	missense	-	-	-	0.933:D	1	0	0	0	-
MAN2B1	19:12758077	C	T	NM_000528	c.2893G>A:p.A965T	missense	0.0002	0.0006	0.0001	0.518:T	0	0	3	1	-
MAN2B1	19:12758106	G	A	NM_000528	c.2864C>T:p.T955M	missense	0	-	0	0.451:T	0	1	0	1	-

MAN2B1	19:12758139	G	C	NM_000528	c.2831C>G:p.S944C	missense	-	-	-	0.702:D	1	0	0	0	-
MAN2B1	19:12758304	C	T	NM_000528	c.2773G>A:p.E925K	missense	5.80E-05	-	-	0.715:D	1	0	0	0	-
MAN2B1	19:12758310	C	T	NM_000528	c.2767G>A:p.V923I	missense	5.80E-05	0.0006	0.0001	0.259:T	1	2	1	3	US
MAN2B1	19:12758333	A	G	NM_000528	c.2744T>C:p.L915P	missense	-	-	-	0.994:D	1	0	0	0	CIP
MAN2B1	19:12759041	A	C	NM_000528	c.2612T>G:p.L871R	missense	-	-	-	0.954:D	0	0	1	0	-
MAN2B1	19:12759089	C	A	NM_000528	c.2564G>T:p.G855V	missense	-	-	-	0.346:T	0	0	1	0	-
MAN2B1	19:12759137	C	G	NM_000528	c.2516G>C:p.R839P	missense	-	-	-	0.961:D	0	1	0	0	-
MAN2B1	19:12759164	A	G	NM_000528	c.2489T>C:p.M830T	missense	-	0.0006	-	0.372:T	0	0	1	0	-
MAN2B1	19:12759176	G	A	NM_000528	c.2477C>T:p.S826L	missense	5.8E-05	-	-	0.395:T	0	0	1	0	-
MAN2B1	19:12759982	T	C	NM_000528	c.2404A>G:p.S802G	missense	0.0043	0.0068	0.0042	0.609:T	26	12	18	16	LB
MAN2B1	19:12759988	C	G	NM_000528	c.2398G>C:p.G800R	missense	5.80E-05	-	-	0.994:D	1	0	0	0	CIP
MAN2B1	19:12759996	C	T	NM_000528	c.2390G>A:p.R797H	missense	-	-	-	0.931:D	1	0	0	0	-
MAN2B1	19:12759997	G	A	NM_000528	c.2389C>T:p.R797C	missense	0	-	0	0.880:D	1	0	0	0	US
MAN2B1	19:12760171	G	A	NM_000528	c.2339C>T:p.T780I	missense	-	-	-	0.651:T	0	0	1	0	-
MAN2B1	19:12760207	G	A	NM_000528	c.2303C>T:p.T768M	missense	0.0001	-	0.0001	0.743:D	0	0	0	1	-
MAN2B1	19:12760216	A	C	NM_000528	c.2294T>G:p.L765R	missense	-	-	-	0.971:D	0	0	1	0	-
MAN2B1	19:12760241	G	A	NM_000528	c.2269C>T:p.R757W	missense	0.0002	-	0.0001	0.653:T	0	2	0	0	-
MAN2B1	19:12760763	T	C	NM_000528	c.2231A>G:p.Y744C	missense	-	-	-	0.868:D	2	1	1	0	-
MAN2B1	19:12760776	T	G	NM_000528	c.2218A>C:p.K740Q	missense	-	-	-	0.352:T	0	0	0	1	-
MAN2B1	19:12760780	CTCC	-	NM_000528	c.2211_2214del:p.E738Qfs*26	frameshift deletion	-	-	-	-	0	0	0	1	-
MAN2B1	19:12760787	G	A	NM_000528	c.2207C>T:p.P736L	missense	0	-	-	0.393:T	1	0	0	0	-
MAN2B1	19:12760800	G	A	NM_000528	c.2194C>T:p.R732C	missense	0	-	0	0.825:D	0	1	0	0	US
MAN2B1	19:12760824	T	A	NM_000528	c.2170A>T:p.T724S	missense	-	-	-	0.126:T	0	0	1	0	-
MAN2B1	19:12760952	C	G	NM_000528	c.2131G>C:p.E711Q	missense	-	-	-	0.902:D	1	0	0	0	-
MAN2B1	19:12760999	G	A	NM_000528	c.2084C>T:p.A695V	missense	-	-	-	0.498:T	0	0	1	0	-
MAN2B1	19:12762978	G	A	NM_000528	c.2035C>T:p.H679Y	missense	-	0	-	0.270:T	1	0	0	0	-
MAN2B1	19:12763001	G	A	NM_000528	c.2012C>T:p.P671L	missense	-	0.0006	-	0.235:T	0	0	1	0	-
MAN2B1	19:12763019	T	-	NM_000528	c.1994delA:p.N665Tfs*7	frameshift deletion	-	-	-	-	0	1	0	0	-
MAN2B1	19:12763025	C	T	NM_000528	c.1988G>A:p.R663K	missense	0.0001	0.0006	0.0001	0.794:D	1	1	0	0	-
MAN2B1	19:12763188	C	A	NM_000528	c.1917G>T:p.Q639H	missense	-	-	-	0.888:D	1	0	0	0	-

MAN2B1	19:12763261	G	A	NM_000528	c.1844C>T:p.T615M	missense	0	0	0	0.466:T	0	1	0	0	-
MAN2B1	19:12763267	C	T	NM_000528	c.1838G>A:p.R613Q	missense	0	-	0	0.365:T	0	1	0	0	CIP
MAN2B1	19:12763268	G	A	NM_000528	c.1837C>T:p.R613W	missense	0.0002	0	0.0003	0.805:D	0	1	0	1	-
MAN2B1	19:12766542	C	A	NM_000528	c.1796G>T:p.R599I	missense	0.0017	0.0012	0.0021	0.294:T	7	4	9	3	US
MAN2B1	19:12766545	C	T	NM_000528	c.1793G>A:p.R598K	missense	-	-	-	0.130:T	1	0	0	0	-
MAN2B1	19:12767500	A	C	NM_000528	c.1529T>G:p.F510C	missense	-	-	-	0.935:D	1	0	0	0	-
MAN2B1	19:12767773	G	A	NM_000528	c.1517C>T:p.T506M	missense	0	0	-	0.435:T	0	0	1	0	-
MAN2B1	19:12767854	G	A	NM_000528	c.1436C>T:p.A479V	missense	0.0002	-	0.0001	0.381:T	1	0	0	0	-
MAN2B1	19:12767855	C	A	NM_000528	c.1435G>T:p.A479S	missense	0.0003	0	0.0003	0.225:T	0	2	1	0	US
MAN2B1	19:12768271	C	T	NM_000528	c.1408G>A:p.G470R	missense	-	-	-	0.160:T	0	0	1	0	-
MAN2B1	19:12768368	A	T	NM_000528	c.1311T>A:p.N437K	missense	-	-	-	0.274:T	0	0	0	1	-
MAN2B1	19:12768948	T	C	NM_000528	c.1238A>G:p.N413S	missense	0.0001	0	0.0001	0.227:T	1	0	0	0	B
MAN2B1	19:12768955	C	T	NM_000528	c.1231G>A:p.V411M	missense	0.0003	0.0006	-	0.633:T	1	1	1	1	-
MAN2B1	19:12769081	G	A	NM_000528	c.1187C>T:p.P396L	missense	5.80E-05	-	0.0001	0.915:D	2	0	0	0	-
MAN2B1	19:12769112	G	T	NM_000528	c.1156C>A:p.Q386K	missense	5.80E-05	-	-	0.321:T	0	1	0	0	-
MAN2B1	19:12769147	T	C	NM_000528	c.1121A>G:p.H374R	missense	-	-	-	0.264:T	0	0	1	0	-
MAN2B1	19:12769250	G	C	NM_000528	c.1101C>G:p.N367K	missense	0	0	0	0.508:T	0	1	0	0	-
MAN2B1	19:12769302	-	G	NM_000528	c.1048dupC:p.H350Pfs*25	frameshift insertion	-	-	-	-	0	2	0	1	-
MAN2B1	19:12774532	C	A	NM_000528	c.748G>T:p.A250S	missense	0.0171	0.0129	0.0237	0.687:T	34	21	18	20	B
MAN2B1	19:12774561	C	T	NM_000528	c.719G>A:p.R240Q	missense	0.0098	0.0062	0.0105	0.812:D	37	36	32	19	B/LB
MAN2B1	19:12774562	G	A	NM_000528	c.718C>T:p.R240W	missense	0.0001	0	0	0.933:D	1	1	2	1	US
MAN2B1	19:12774586	T	C	NM_000528	c.694A>G:p.K232E	missense	-	-	-	0.142:T	0	4	1	1	-
MAN2B1	19:12774622	G	A	NM_000528	c.658C>T:p.R220C	missense	5.8E-05	-	0.0001	0.981:D	0	0	1	0	-
MAN2B1	19:12774637	C	T	NM_000528	c.643G>A:p.G215S	missense	0	-	-	0.913:D	0	0	0	1	-
MAN2B1	19:12774645	C	A	NM_000528	c.635G>T:p.G212V	missense	0.0002	-	0.0003	0.976:D	0	1	0	0	-
MAN2B1	19:12775652	A	G	NM_000528	c.584T>C:p.I195T	missense	-	-	-	0.983:D	1	0	0	0	-
MAN2B1	19:12775671	G	T	NM_000528	c.565C>A:p.P189T	missense	0.0013	-	0.0011	0.985:D	2	0	0	0	US
MAN2B1	19:12775721	A	G	NM_000528	c.515T>C:p.M172T	missense	-	-	-	0.950:D	1	0	0	0	-
MAN2B1	19:12775781	T	C	NM_000528	c.455A>G:p.N152S	missense	0.0001	0	0.0001	0.547:T	0	1	2	0	CIP
MAN2B1	19:12776183	C	T	NM_000528	c.419G>A:p.R140Q	missense	0	0	0	0.362:T	0	1	1	0	LB

MAN2B1	19:12776205	T	C	NM_000528	c.397A>G:p.N133D	missense	5.8E-05	-	0.0001	0.349:T	0	0	0	1	-
MAN2B1	19:12776207	G	A	NM_000528	c.395C>T:p.T132I	missense	-	-	-	0.579:T	0	0	1	0	-
MAN2B1	19:12776316	C	T	NM_000528	c.286G>A:p.G96S	missense	5.8E-05	0	0.0001	0.466:T	0	0	0	2	-
MAN2B1	19:12776328	T	C	NM_000528	c.274A>G:p.I92V	missense	0.0011	-	0.0015	0.339:T	6	0	3	1	US
MAN2B1	19:12776612	G	A	NM_000528	c.167C>T:p.P56L	missense	-	-	-	0.603:T	0	1	0	0	-
MAN2B1	19:12776619	T	G	NM_000528	c.160A>C:p.T54P	missense	-	-	-	0.682:T	1	0	0	0	-
MAN2B1	19:12777457	C	A	NM_000528	c.59G>T:p.G20V	missense	-	-	-	0.284:T	1	0	0	0	-
MAN2B1	19:12777514	AT	-	NM_000528	c.1_2del	frameshift deletion	9.98E-05	-	-	-	1	0	1	0	US
BLOC1S3	19:45682568	G	T	NM_212550	c.14G>T:p.G5V	missense	6.60E-05	-	-	0.302:T	0	2	0	1	-
BLOC1S3	19:45682589	G	A	NM_212550	c.35G>A:p.R12Q	missense	-	-	-	0.069:T	0	0	1	1	-
BLOC1S3	19:45682609	C	T	NM_212550	c.55C>T:p.P19S	missense	6.49E-05	-	-	0.051:T	1	0	0	0	-
BLOC1S3	19:45682654	T	G	NM_212550	c.100T>G:p.S34A	missense	0.0003	0	0.0007	0.261:T	1	2	1	0	-
BLOC1S3	19:45682655	C	A	NM_212550	c.101C>A:p.S34X	stopgain	0.0004	0	0.0007	0.472:T	1	2	1	0	-
BLOC1S3	19:45682666	G	C	NM_212550	c.112G>C:p.E38Q	missense	-	-	-	0.343:T	0	1	0	1	-
BLOC1S3	19:45682702	C	T	NM_212550	c.148C>T:p.R50C	missense	0.0021	0.0006	0.0038	0.651:T	15	10	19	4	-
BLOC1S3	19:45682705	C	A	NM_212550	c.151C>A:p.P51T	missense	-	-	-	0.266:T	0	1	0	1	-
BLOC1S3	19:45682748	C	T	NM_212550	c.194C>T:p.S65L	missense	0	-	-	0.402:T	0	0	1	0	-
BLOC1S3	19:45682758	G	T	NM_212550	c.204G>T:p.E68D	missense	0.0031	0.0006	0	0.116:T	14	4	4	5	-
BLOC1S3	19:45682759	C	T	NM_212550	c.205C>T:p.P69S	missense	0.0031	0.0006	0	0.083:T	14	4	4	5	-
BLOC1S	19:45682829	A	C	NM_212550	c.275A>C:p.E92A	missense	0	0	-	0.099:T	0	1	1	1	-

3															
BLOC1S3	19:45682830	G	-	NM_212550	c.276delG:p.A93Pfs*35	frameshift deletion	-	-	-	-	0	0	0	1	-
BLOC1S3	19:45682841	C	T	NM_212550	c.287C>T:p.T96M	missense	-	-	-	0.037:T	0	0	0	1	-
BLOC1S3	19:45682876	C	G	NM_212550	c.322C>G:p.L108V	missense	0	0.0006	0	0.448:T	1	0	1	0	B/LB
BLOC1S3	19:45682883	A	G	NM_212550	c.329A>G:p.Q110R	missense	-	-	-	0.524:T	1	0	0	0	-
BLOC1S3	19:45682940	G	A	NM_212550	c.386G>A:p.S129N	missense	-	-	-	0.078:T	1	0	0	0	-
BLOC1S3	19:45682973	C	A	NM_212550	c.419C>A:p.A140D	missense	-	-	-	0.445:T	0	0	1	0	-
BLOC1S3	19:45682979	T	C	NM_212550	c.425T>C:p.L142P	missense	-	-	-	0.839:D	1	0	0	0	-
BLOC1S3	19:45683096	G	A	NM_212550	c.542G>A:p.R181H	missense	0.0001	-	0.0001	0.191:T	1	0	0	0	-
CTSA	20:44519984	C	A	NM_000308	c.20C>A:p.A7E	missense	7.71E-05	-	0	0.070:T	1	0	0	0	-
CTSA	20:44520007	G	T	NM_000308	c.43G>T:p.G15W	missense	-	-	-	0.066:T	0	0	1	0	-
CTSA	20:44520010	G	A	NM_000308	c.46G>A:p.G16R	missense	-	-	-	0.021:T	0	1	0	1	-
CTSA	20:44520260	T	G	NM_000308	c.107T>G:p.L36R	missense	0.0001	0.0006	0.0001	0.624:T	2	0	0	0	-
CTSA	20:44520274	G	C	NM_000308	c.121G>C:p.A41P	missense	-	-	-	0.206:T	0	1	0	0	-
CTSA	20:44520337	C	A	NM_000308	c.184C>A:p.Q62K	missense	-	-	-	0.531:T	0	0	1	0	-
CTSA	20:44520341	C	A	NM_000308	c.188C>A:p.P63Q	missense	5.81E-05	-	0.0001	0.777:D	0	1	1	0	-
CTSA	20:44520341	C	T	NM_000308	c.188C>T:p.P63L	missense	0.0006	0.0012	0.0002	0.723:D	0	0	2	0	-
CTSA	20:44520578	C	G	NM_000308	c.272C>G:p.P91R	missense	-	-	-	0.787:D	1	0	0	0	-
CTSA	20:44520586	A	G	NM_000308	c.280A>G:p.S94G	missense	0	-	-	0.547:T	0	0	1	0	-
CTSA	20:44520637	G	A	NM_000308	c.331G>A:p.G111R	missense	-	-	-	0.883:D	0	1	0	0	-
CTSA	20:44520640	C	G	NM_000308	c.334C>G:p.L112V	missense	-	-	-	0.563:T	0	1	0	0	-
CTSA	20:44521044	A	G	NM_000308	c.419A>G:p.N140S	missense	0	0	0	0.460:T	0	2	1	0	-
CTSA	20:44521421	A	G	NM_000308	c.556A>G:p.K186E	missense	-	-	-	0.417:T	0	1	0	0	-
CTSA	20:44521431	A	G	NM_000308	c.566A>G:p.K189R	missense	-	-	-	0.311:T	0	1	0	1	-

CTSA	20:44521518	A	G	NM_000308	c.653A>G:p.Q218R	missense	-	-	-	0.616:T	1	0	0	0	-
CTSA	20:44521863	T	C	NM_000308	c.659T>C:p.L220P	missense	-	-	-	0.803:D	0	0	1	0	-
CTSA	20:44521883	T	G	NM_000308	c.679T>G:p.S227A	missense	-	-	-	0.497:T	0	0	0	2	-
CTSA	20:44521897	G	C	NM_000308	c.693G>C:p.Q231H	missense	-	-	-	0.422:T	0	0	1	0	-
CTSA	20:44522686	A	G	NM_000308	c.806A>G:p.N269S	missense	-	-	-	0.267:T	1	0	0	0	-
CTSA	20:44523304	C	T	NM_000308	c.847C>T:p.R283C	missense	0.0001	0	0.0001	0.443:T	0	0	1	1	US
CTSA	20:44523305	G	A	NM_000308	c.848G>A:p.R283H	missense	0	-	-	0.090:T	0	1	0	0	-
CTSA	20:44523350	C	T	NM_000308	c.893C>T:p.P298L	missense	5.80E-05	-	0	0.644:T	0	2	0	1	US
CTSA	20:44523359	G	C	NM_000308	c.902G>C:p.G301A	missense	5.80E-05	-	-	0.704:D	1	0	0	1	-
CTSA	20:44523359	G	A	NM_000308	c.902G>A:p.G301E	missense	-	-	-	0.690:T	1	0	0	0	-
CTSA	20:44523651	G	A	NM_000308	c.1021G>A:p.D341N	missense	0	-	0	0.177:T	0	0	1	0	-
CTSA	20:44523660	C	T	NM_000308	c.1030C>T:p.R344C	missense	5.80E-05	-	0.0001	0.618:T	1	1	2	1	-
CTSA	20:44523661	G	A	NM_000308	c.1031G>A:p.R344H	missense	5.8E-05	0	0.0001	0.311:T	0	0	1	0	US
CTSA	20:44523664	T	C	NM_000308	c.1034T>C:p.M345T	missense	0.0004	0.0006	0.0003	0.355:T	2	2	2	0	US
CTSA	20:44523693	G	C	NM_000308	c.1063G>C:p.A355P	missense	0.0008	-	0.0008	0.363:T	0	0	1	0	US
CTSA	20:44523720	G	A	NM_000308	c.1090G>A:p.V364M	missense	0.0034	0.0012	0.004	0.781:D	12	10	6	5	LB
CTSA	20:44523728	G	C	NM_000308	c.1098G>C:p.K366N	missense	0.0002	-	0.0002	0.426:T	2	0	0	0	-
CTSA	20:44523742	C	T	NM_000308	c.1112C>T:p.P371L	missense	5.80E-05	0	0	0.586:T	1	0	0	0	-
CTSA	20:44523765	A	G	NM_000308	c.1135A>G:p.M379V	missense	0.0007	0.0012	0.0008	0.299:T	4	4	3	3	-
CTSA	20:44525626	C	T	NM_000308	c.1165C>T:p.R389C	missense	0	-	0	0.531:T	0	0	0	1	-
CTSA	20:44526425	G	A	NM_000308	c.1288G>A:p.V430M	missense	0.0001	-	-	0.804:D	0	0	1	0	-
CTSA	20:44526645	T	C	NM_000308	c.1310T>C:p.M437T	missense	-	-	-	0.396:T	0	0	0	1	-
CTSA	20:44526657	G	A	NM_000308	c.1322G>A:p.R441H	missense	0	0	0	0.781:D	0	1	0	0	-
CTSA	20:44526704	G	A	NM_000308	c.1369G>A:p.G457S	missense	0	0	0	0.907:D	1	1	0	0	P
CTSA	20:44526732	C	T	NM_000308	c.1397C>T:p.A466V	missense	0.0011	-	0.0007	0.514:T	10	11	7	7	-
CTSA	20:44526749	G	A	NM_000308	c.1413+1G>A	splicing	0	-	0	0.748:D	0	1	0	1	-
CTSA	20:44527012	G	A	NM_000308	c.1420G>A:p.G474S	missense	0	-	0	0.893:D	0	0	2	0	US
CTSA	20:44527042	G	A	NM_000308	c.1450G>A:p.A484T	missense	5.80E-05	-	-	0.775:D	0	1	0	0	-
DNAJC5	20:62559804	C	T	NM_025219	c.106C>T:p.R36W	missense	-	-	-	0.897:D	0	0	1	0	-
DNAJC5	20:62560775	G	A	NM_025219	c.218G>A:p.R73K	missense	5.80E-05	-	-	0.705:D	1	0	2	1	-
DNAJC5	20:62560868	G	C	NM_025219	c.311G>C:p.W104S	missense	-	-	-	0.910:D	0	1	0	0	-

DNAJC5	20:62562232	C	T	NM_025219	c.350C>T:p.T117M	missense	0	-	0	0.827:D	0	0	1	0	-
DNAJC5	20:62562301	C	T	NM_025219	c.419C>T:p.A140V	missense	0	0	0	0.311:T	0	1	0	0	CIP
DNAJC5	20:62562303	C	G	NM_025219	c.421C>G:p.P141A	missense	0.0002	-	0.0001	0.326:T	3	4	1	0	-
DNAJC5	20:62562319	C	T	NM_025219	c.437C>T:p.T146M	missense	5.80E-05	-	0.0001	0.281:T	0	1	0	1	US
DNAJC5	20:62562835	A	G	NM_025219	c.511A>G:p.I171V	missense	-	-	-	0.265:T	1	0	0	0	-
DNAJC5	20:62562881	C	T	NM_025219	c.557C>T:p.A186V	missense	-	-	-	0.389:T	0	0	1	0	-
HPS4	22:26840657	A	C	NM_001349904	c.1959T>G:p.C653W	missense	-	-	-	-	0	0	0	1	-
HPS4	22:26849237	T	C	NM_152841	c.2074A>G:p.K692E	missense	0	-	0	0.390:T	0	0	1	0	-
HPS4	22:26849247	G	A	NM_001349902	c.1837C>T:p.R613W	missense	0.0002	0.0006	0.0001	-	1	2	0	2	B/LB
HPS4	22:26849257	A	G	NM_152841	c.2054T>C:p.F685S	missense	-	-	-	0.648:T	1	0	0	0	-
HPS4	22:26849261	C	T	NM_152841	c.2050G>A:p.A684T	missense	0	-	0	0.317:T	0	0	1	0	-
HPS4	22:26849280	G	A	NM_001349902	c.1804C>T:p.P602S	missense	0	-	0	-	0	1	0	1	-
HPS4	22:26849293	C	T	NM_152841	c.2018G>A:p.R673Q	missense	0	-	0	0.369:T	1	0	0	0	-
HPS4	22:26849303	G	A	NM_152841	c.2008C>T:p.P670S	missense	0	-	-	0.005:T	1	0	1	0	-
HPS4	22:26849349	G	A	NM_001349902	c.1735C>T:p.R579C	missense	0.0002	0.0006	0.0001	-	0	2	0	0	-
HPS4	22:26849359	G	A	NM_152841	c.1952C>T:p.T651M	missense	5.80E-05	0	0	0.584:T	0	1	0	0	-
HPS4	22:26849362	G	C	NM_152841	c.1949C>G:p.S650C	missense	-	-	-	0.539:T	0	1	0	0	-
HPS4	22:26849364	G	A	NM_001349902	c.1720C>T:p.L574F	missense	0	0	0	-	0	1	0	0	B
HPS4	22:26853833	C	T	NM_152841	c.1932G>A:p.M644I	missense	0.0176	0.0154	0.0167	0.211:T	69	72	52	37	US
HPS4	22:26853904	C	T	NM_152841	c.1861G>A:p.D621N	missense	-	-	-	0.061:T	0	1	0	0	-
HPS4	22:26853909	G	A	NM_152841	c.1856C>T:p.P619L	missense	0.0006	0.0006	0.0004	0.182:T	1	0	0	0	-
HPS4	22:26854412	C	G	NM_152841	c.1830G>C:p.M610I	missense	5.8E-05	-	-	0.113:T	0	0	0	1	-
HPS4	22:26854432	G	A	NM_152841	c.1810C>T:p.R604C	missense	5.80E-05	-	0.0001	0.139:T	2	0	0	1	-
HPS4	22:26854479	T	A	NM_152841	c.1763A>T:p.D588V	missense	5.80E-05	-	0.0001	0.293:T	1	2	1	0	-
HPS4	22:26854491	G	A	NM_152841	c.1751C>T:p.T584M	missense	0	0	0	0.419:T	1	0	1	0	-
HPS4	22:26854534	T	C	NM_152841	c.1708A>G:p.S570G	missense	-	-	-	0.759:D	1	0	0	0	-
HPS4	22:26859893	A	G	NM_152841	c.1688T>C:p.I563T	missense	0.0001	-	0.0002	0.508:T	1	0	0	0	-
HPS4	22:26859908	C	T	NM_152841	c.1673G>A:p.G558E	missense	0	-	-	0.187:T	0	1	0	0	-
HPS4	22:26859954	C	T	NM_152841	c.1627G>A:p.V543I	missense	0.001	-	0.001	0.298:T	0	0	2	1	-
HPS4	22:26859955	G	C	NM_152841	c.1626C>G:p.C542W	missense	0	-	-	0.481:T	0	1	0	1	-
HPS4	22:26859956	C	G	NM_152841	c.1625G>C:p.C542S	missense	0.0021	0.0018	0.0024	0.168:T	10	10	6	5	-

HPS4	22:26860005	G	A	NM_152841	c.1576C>T:p.P526S	missense	0	-	0	0.050:T	0	0	0	1	-
HPS4	22:26860052	C	A	NM_152841	c.1529G>T:p.C510F	missense	-	0.0006	-	0.062:T	0	0	0	1	-
HPS4	22:26860067	G	A	NM_152841	c.1514C>T:p.S505L	missense	-	-	-	0.166:T	0	0	1	0	-
HPS4	22:26860074	A	G	NM_152841	c.1507T>C:p.C503R	missense	-	-	-	0.006:T	0	0	1	0	-
HPS4	22:26860154	T	C	NM_152841	c.1427A>G:p.N476S	missense	5.80E-05	0.0006	0.0001	0.000:T	2	0	0	0	-
HPS4	22:26860158	CT	-	NM_152841	c.1422_1423del:p.G475Kfs*11	frameshift deletion	-	-	-	-	0	1	0	0	-
HPS4	22:26860161	T	A	NM_152841	c.1420A>T:p.R474X	stopgain	-	-	-	0.329:T	0	1	0	0	-
HPS4	22:26860199	C	T	NM_152841	c.1382G>A:p.R461H	missense	0	0	0	0.047:T	0	1	0	0	US
HPS4	22:26860226	C	G	NM_152841	c.1355G>C:p.R452T	missense	0	-	0	0.178:T	0	1	0	0	-
HPS4	22:26860236	G	T	NM_152841	c.1345C>A:p.P449T	missense	5.80E-05	-	0.0001	0.143:T	1	0	0	0	-
HPS4	22:26860266	C	A	NM_152841	c.1315G>T:p.E439X	stopgain	-	-	-	0.289:T	1	1	0	0	-
HPS4	22:26860289	T	C	NM_152841	c.1292A>G:p.Q431R	missense	-	-	-	0.184:T	1	0	1	1	-
HPS4	22:26860320	G	A	NM_152841	c.1261C>T:p.R421C	missense	-	0	-	0.107:T	0	0	0	1	-
HPS4	22:26860352	G	A	NM_152841	c.1229C>T:p.T410M	missense	0	0	0	0.028:T	0	0	0	1	-
HPS4	22:26860374	C	T	NM_152841	c.1207G>A:p.A403T	missense	5.80E-05	0	0	0.024:T	0	1	0	0	US
HPS4	22:26860394	T	A	NM_152841	c.1187A>T:p.Y396F	missense	0.0002	-	0.0002	0.069:T	0	0	1	0	-
HPS4	22:26860529	A	G	NM_152841	c.1052T>C:p.L351P	missense	5.8E-05	-	-	0.123:T	0	0	0	1	-
HPS4	22:26860539	T	C	NM_152841	c.1042A>G:p.S348G	missense	0.0004	-	0.0003	0.017:T	1	0	1	0	-
HPS4	22:26860581	C	T	NM_152841	c.1000G>A:p.A334T	missense	0	-	-	0.014:T	0	1	0	1	-
HPS4	22:26860608	C	T	NM_152841	c.973G>A:p.G325S	missense	0.0001	0.0006	-	0.151:T	2	1	0	0	-
HPS4	22:26860655	G	A	NM_152841	c.926C>T:p.P309L	missense	-	-	-	0.146:T	0	1	0	0	-
HPS4	22:26860668	C	G	NM_152841	c.913G>C:p.D305H	missense	0.0001	-	0.0002	0.211:T	1	0	1	0	-
HPS4	22:26860707	C	T	NM_152841	c.874G>A:p.A292T	missense	0	-	0	0.199:T	0	0	1	0	-
HPS4	22:26861502	G	A	NM_152841	c.707C>T:p.P236L	missense	0.0002	-	0.0003	0.253:T	1	0	0	0	-
HPS4	22:26861514	G	A	NM_152841	c.695C>T:p.A232V	missense	0.001	0	0.0017	0.303:T	0	0	1	0	CIP
HPS4	22:26862032	G	A	NM_001349900	c.745C>T:p.H249Y	missense	8.47E-05	-	-	0.317:T	0	1	1	0	-
HPS4	22:26862067	T	C	NM_001349900	c.710A>G:p.K237R	missense	0.0022	0.0025	0.004	0.090:T	7	5	10	5	-
HPS4	22:26862203	G	A	NM_152841	c.680C>T:p.P227L	missense	0.0002	-	0.0001	0.363:T	1	0	0	1	-
HPS4	22:26864561	G	C	NM_152841	c.610C>G:p.L204V	missense	0.0001	-	0.0001	0.370:T	0	1	0	0	-
HPS4	22:26864587	A	G	NM_152841	c.584T>C:p.I195T	missense	-	-	-	0.765:D	3	1	2	1	-
HPS4	22:26866738	C	A	NM_152841	c.528G>T:p.Q176H	missense	0	0	-	0.914:D	2	2	0	2	-

HPS4	22:26868323	T	C	NM_152841	c.431A>G:p.N144S	missense	-	-	-	0.118:T	0	1	0	0	-
HPS4	22:26868356	T	C	NM_152841	c.398A>G:p.E133G	missense	-	-	-	0.543:T	0	1	0	0	-
HPS4	22:26868359	G	A	NM_152841	c.395C>T:p.T132M	missense	0	0	0	0.489:T	1	0	0	0	-
HPS4	22:26868808	A	G	NM_152841	c.359T>C:p.L120P	missense	-	-	-	0.877:D	2	0	1	2	-
HPS4	22:26868809	G	C	NM_152841	c.358C>G:p.L120V	missense	0.0042	0.0037	0.0034	0.449:T	21	19	15	18	US
HPS4	22:26868881	G	A	NM_152841	c.286C>T:p.P96S	missense	5.8E-05	-	0	0.501:T	0	0	0	1	-
HPS4	22:26868899	C	T	NM_152841	c.268G>A:p.G90S	missense	0.0002	-	0.0001	0.979:D	3	2	1	0	-
HPS4	22:26873008	C	T	NM_152841	c.212G>A:p.R71H	missense	0.0005	0	0.0003	0.704:D	0	0	1	0	-
HPS4	22:26873029	T	C	NM_152841	c.191A>G:p.D64G	missense	5.8E-05	-	0.0001	0.279:T	0	0	2	0	-
HPS4	22:26873065	A	G	NM_152841	c.155T>C:p.I52T	missense	0.0002	-	0.0001	0.931:D	0	0	1	0	-
HPS4	22:26875345	C	T	NM_152841	c.3G>A:p.M1I	missense	-	-	-	0.306:T	0	1	0	0	-
HPS4	22:26877691	G	A	NM_001349896	c.38C>T:p.S13L	missense	5.80E-05	-	0.0001	0.575:T	1	0	0	0	US
HPS4	22:26877709	G	C	NM_001349896	c.20C>G:p.T7R	missense	0.0006	0.0012	0.0005	0.366:T	1	2	0	1	-
HPS4	22:26877716	T	C	NM_001349896	c.13A>G:p.T5A	missense	0	-	0	0.272:T	0	1	1	0	US
NAGA	22:42456298	C	A	NM_000262	c.1221G>T:p.E407D	missense	-	-	-	0.360:T	0	1	0	0	-
NAGA	22:42456369	T	G	NM_000262	c.1150A>C:p.T384P	missense	-	-	-	0.614:T	0	0	1	0	-
NAGA	22:42456417	C	T	NM_000262	c.1102G>A:p.A368T	missense	-	-	-	0.448:T	0	1	0	0	-
NAGA	22:42456998	C	T	NM_000262	c.1031G>A:p.R344K	missense	-	-	-	0.800:D	1	0	0	0	-
NAGA	22:42457019	G	A	NM_000262	c.1010C>T:p.A337V	missense	-	-	-	0.955:D	2	0	0	0	-
NAGA	22:42457020	C	T	NM_000262	c.1009G>A:p.A337T	missense	0.0001	-	0.0002	0.962:D	0	1	1	0	-
NAGA	22:42457052	A	G	NM_000262	c.977T>C:p.V326A	missense	0	-	0	0.952:D	0	1	0	1	-
NAGA	22:42457057	G	C	NM_000262	c.972C>G:p.I324M	missense	0.0001	-	0.0002	0.896:D	0	0	1	0	-
NAGA	22:42458871	T	C	NM_000262	c.917A>G:p.N306S	missense	0.0001	-	0.0001	0.487:T	1	0	0	0	-
NAGA	22:42458917	C	T	NM_000262	c.871G>A:p.A291T	missense	0.0003	0.0025	0.0002	0.155:T	2	1	3	1	-
NAGA	22:42458929	G	A	NM_000262	c.859C>T:p.R287C	missense	0.0002	0.0006	0.0002	0.978:D	4	4	5	1	US
NAGA	22:42458955	G	T	NM_000262	c.833C>A:p.A278D	missense	-	-	-	0.996:D	0	1	0	0	-
NAGA	22:42458980	T	C	NM_000262	c.808A>G:p.M270V	missense	5.8E-05	-	-	0.961:D	0	0	1	0	-
NAGA	22:42459000	A	G	NM_000262	c.788T>C:p.L263S	missense	-	-	-	0.527:T	1	1	0	3	-
NAGA	22:42461744	TG	-	NM_000262	c.756_757del:p.M253Afs*82	frameshift deletion	-	-	-	-	0	0	0	1	-
NAGA	22:42461744	T	C	NM_000262	c.757A>G:p.M253V	missense	-	-	-	0.991:D	0	0	1	0	-
NAGA	22:42461746	T	A	NM_000262	c.755A>T:p.D252V	missense	-	-	-	1.000:D	0	0	0	1	-

NAGA	22:42461828	C	T	NM_000262	c.673G>A:p.V225M	missense	0	0	0	0.924:D	0	1	1	0	-
NAGA	22:42461864	G	A	NM_000262	c.637C>T:p.R213C	missense	0	0	0	0.995:D	1	0	0	0	-
NAGA	22:42461884	G	A	NM_000262	c.617C>T:p.A206V	missense	0	0.0006	-	0.381:T	0	0	4	0	-
NAGA	22:42462719	G	T	NM_000262	c.592C>A:p.P198T	missense	0.0006	0.0012	0.0015	0.877:D	3	3	0	3	-
NAGA	22:42462770	G	A	NM_000262	c.541C>T:p.R181C	missense	0.0007	0.0012	0.0016	0.952:D	1	1	1	5	-
NAGA	22:42462804	G	T	NM_000262	c.507C>A:p.Y169X	stopgain	-	-	-	0.187:T	1	0	0	0	-
NAGA	22:42463126	G	A	NM_000262	c.493C>T:p.R165W	missense	0.0002	0	0.0001	0.710:D	1	0	0	0	US
NAGA	22:42463165	T	C	NM_000262	c.454A>G:p.M152V	missense	-	-	-	0.948:D	0	0	0	1	-
NAGA	22:42463180	C	T	NM_000262	c.439G>A:p.E147K	missense	0	0	0	0.087:T	1	0	0	0	-
NAGA	22:42463183	C	T	NM_000262	c.436G>A:p.A146T	missense	0	0	0	0.782:D	0	0	1	0	-
NAGA	22:42463210	T	C	NM_000262	c.409A>G:p.K137E	missense	0.0001	0.0006	-	0.319:T	1	0	1	0	-
NAGA	22:42463260	G	A	NM_000262	c.359C>T:p.A120V	missense	0.0001	0	0.0001	0.484:T	2	2	0	0	-
NAGA	22:42463261	C	T	NM_000262	c.358G>A:p.A120T	missense	0.0001	-	0.0001	0.413:T	1	0	1	0	US
NAGA	22:42463768	C	G	NM_000262	c.324+1G>C	splicing	-	-	-	0.607:T	0	0	1	0	LP
NAGA	22:42463769	G	-	NM_000262	c.324delC:p.Y108fs*0	stopgain	0	-	0	-	0	1	0	0	-
NAGA	22:42463769	G	-	NM_000262	c.324delC:p.Y108fs*0	stopgain	0	-	0	-	0	1	0	0	-
NAGA	22:42463779	A	G	NM_000262	c.314T>C:p.L105P	missense	0.0003	0.0012	0.0003	1.000:D	0	0	1	0	-
NAGA	22:42463788	A	G	NM_000262	c.305T>C:p.I102T	missense	0.0001	0.0006	0.0002	0.974:D	2	1	0	1	-
NAGA	22:42463824	C	T	NM_000262	c.269G>A:p.R90H	missense	0	-	-	0.346:T	0	1	0	0	-
NAGA	22:42463825	G	A	NM_000262	c.268C>T:p.R90C	missense	5.80E-05	0	0.0001	0.579:T	0	1	0	0	-
NAGA	22:42463831	T	C	NM_000262	c.262A>G:p.S88G	missense	0.0006	0.0018	0.0008	0.281:T	3	0	1	1	-
NAGA	22:42463837	C	T	NM_000262	c.256G>A:p.D86N	missense	5.8E-05	-	0	0.455:T	0	0	1	0	-
NAGA	22:42463846	C	T	NM_000262	c.247G>A:p.G83S	missense	5.8E-05	-	0.0001	0.322:T	0	0	0	1	-
NAGA	22:42463891	G	A	NM_000262	c.202C>T:p.R68W	missense	0	0	0	0.879:D	1	0	0	0	-
NAGA	22:42463897	C	T	NM_000262	c.196G>A:p.G66R	missense	0	-	-	0.966:D	1	2	2	1	-
NAGA	22:42464486	G	A	NM_000262	c.109C>T:p.R37C	missense	-	-	-	0.970:D	1	0	0	0	-
ARSA	22:51063610	C	T	NM_000487	c.1493G>A:p.R498H	missense	0	0.0006	0	0.262:T	0	1	0	0	B/LB
ARSA	22:51063610	C	A	NM_000487	c.1493G>T:p.R498L	missense	0.0007	-	0.001	0.294:T	4	1	2	0	US
ARSA	22:51063611	G	A	NM_000487	c.1492C>T:p.R498C	missense	0	0	-	0.383:T	0	2	0	1	-
ARSA	22:51063625	G	-	NM_000487	c.1478delC:p.P493Lfs*26	frameshift deletion	-	-	-	-	1	0	0	0	-
ARSA	22:51063661	C	T	NM_000487	c.1442G>A:p.R481Q	missense	5.84E-05	-	0	0.170:T	1	0	0	0	-

ARSA	22:51063669	C	G	NM_000487	c.1434G>C:p.Q478H	missense	0	-	-	0.697:T	0	1	0	0	US
ARSA	22:51063674	-	G	NM_000487	c.1428dupC:p.S477Qfs*95	frameshift insertion	-	-	-	-	0	0	1	0	-
ARSA	22:51063758	C	T	NM_000487	c.1345G>A:p.G449R	missense	0.0034	0.0031	0.0034	0.389:T	18	13	9	4	LB
ARSA	22:51063758	-	G	NM_000487	c.1344dupC:p.G449Rfs*123	frameshift insertion	0.0003	0.0006	0.0003	-	1	0	2	0	P/LP
ARSA	22:51063767	C	A	NM_000487	c.1336G>T:p.G446C	missense	-	-	-	0.505:T	0	1	0	0	-
ARSA	22:51063778	T	C	NM_000487	c.1325A>G:p.N442S	missense	0	0	0	0.384:T	0	1	0	0	LB
ARSA	22:51063806	G	C	NM_000487	c.1297C>G:p.L433V	missense	0.0038	0.0068	0.004	0.768:D	5	7	4	6	LB
ARSA	22:51063835	G	C	NM_000487	c.1268C>G:p.T423S	missense	-	-	-	0.465:T	1	0	0	0	-
ARSA	22:51063865	T	C	NM_000487	c.1238A>G:p.D413G	missense	5.81E-05	-	-	0.757:D	0	1	0	0	-
ARSA	22:51063866	C	T	NM_000487	c.1237G>A:p.D413N	missense	-	-	-	0.517:T	0	1	0	0	-
ARSA	22:51063869	C	T	NM_000487	c.1234G>A:p.A412T	missense	-	-	-	0.417:T	1	0	0	0	-
ARSA	22:51064057	C	G	NM_000487	c.1160G>C:p.G387A	missense	0	-	-	0.826:D	1	1	0	0	-
ARSA	22:51064064	C	G	NM_000487	c.1153G>C:p.V385L	missense	-	-	-	0.425:T	0	0	1	0	-
ARSA	22:51064067	C	T	NM_000487	c.1150G>A:p.E384K	missense	0	-	0	0.545:T	0	0	1	0	P/LP
ARSA	22:51064102	C	T	NM_000487	c.1115G>A:p.R372Q	missense	0	0	0	0.820:D	0	0	1	0	US
ARSA	22:51064489	C	T	NM_000487	c.982G>A:p.V328M	missense	0.0056	0.0074	0.0087	0.815:D	20	26	22	16	LB
ARSA	22:51064624	G	A	NM_000487	c.937C>T:p.R313X	stopgain	0	-	-	0.780:D	0	0	1	0	P
ARSA	22:51064630	C	T	NM_000487	c.931G>A:p.G311S	missense	0	-	0	0.872:D	0	1	0	0	CIP
ARSA	22:51064636	C	T	NM_000487	c.925G>A:p.E309K	missense	5.87E-05	0	-	0.861:D	0	1	0	0	P
ARSA	22:51064659	C	T	NM_000487	c.902G>A:p.R301Q	missense	0.0002	-	0.0003	0.683:T	2	2	1	0	US
ARSA	22:51064683	C	T	NM_000487	c.878G>A:p.R293Q	missense	0.0002	-	0.0002	0.503:T	1	0	0	0	p.R293X
ARSA	22:51064702	C	G	NM_000487	c.859G>C:p.E287Q	missense	0.0001	-	-	0.788:D	0	1	2	1	-
ARSA	22:51065046	G	A	NM_000487	c.827C>T:p.T276M	missense	0	-	0	0.804:D	1	0	0	0	P/LP
ARSA	22:51065079	G	A	NM_000487	c.794C>T:p.T265I	missense	-	-	-	0.511:T	1	0	0	0	-
ARSA	22:51065090	C	-	NM_000487	c.783delG:p.T262Pfs*1	frameshift deletion	-	-	-	-	0	1	0	0	-
ARSA	22:51065136	C	T	NM_000487	c.737G>A:p.R246H	missense	5.80E-05	-	0	0.838:D	1	0	1	0	P/LP
ARSA	22:51065290	C	T	NM_000487	c.656G>A:p.R219H	missense	0	0	0	0.656:T	1	0	0	0	US

ARSA	22:51065300	G	A	NM_000487	c.646C>T:p.R216C	missense	0	-	-	0.480:T	0	0	0	1	-
ARSA	22:51065324	G	A	NM_000487	c.622C>T:p.H208Y	missense	5.80E-05	-	-	0.399:T	2	1	0	0	-
ARSA	22:51065339	A	G	NM_000487	c.607T>C:p.Y203H	missense	-	-	-	0.827:D	0	0	1	0	P
ARSA	22:51065353	C	T	NM_000487	c.593G>A:p.G198E	missense	-	-	-	0.478:T	1	0	0	0	-
ARSA	22:51065354	C	T	NM_000487	c.592G>A:p.G198R	missense	5.81E-05	-	0.0001	0.398:T	1	1	1	0	US
ARSA	22:51065361	C	A	NM_000487	c.585G>T:p.W195C	missense	0.0002	0.0006	0.0001	0.498:T	1	1	0	0	B
ARSA	22:51065366	G	A	NM_000487	c.580C>T:p.P194S	missense	-	-	-	0.564:T	0	1	0	0	-
ARSA	22:51065381	C	T	NM_000487	c.565G>A:p.V189M	missense	0.0003	-	0.0002	0.598:T	0	0	1	0	-
ARSA	22:51065420	G	A	NM_000487	c.526C>T:p.Q176X	stopgain	0.0001	-	0.0001	0.679:T	0	0	1	0	P/LP
ARSA	22:51065432	C	A	NM_000487	c.514G>T:p.G172C	missense	0.0002	-	0.0002	0.777:D	2	2	2	0	-
ARSA	22:51065435	C	T	NM_000487	c.511G>A:p.D171N	missense	0.0023	0.0019	0.0027	0.228:T	3	3	1	3	CIP
ARSA	22:51065473	C	A	NM_000487	c.473G>T:p.C158F	missense	0	-	-	0.755:D	0	1	0	1	-
ARSA	22:51065604	G	A	NM_000487	c.455C>T:p.S152F	missense	-	-	-	0.785:D	0	1	0	0	-
ARSA	22:51065611	G	A	NM_000487	c.448C>T:p.P150S	missense	-	-	-	0.770:D	0	0	0	1	-
ARSA	22:51065625	C	T	NM_000487	c.434G>A:p.R145Q	missense	0	0	0	0.368:T	0	0	0	1	US
ARSA	22:51065712	C	T	NM_000487	c.347G>A:p.R116Q	missense	0	-	0	0.327:T	1	0	1	0	-
ARSA	22:51065743	C	A	NM_000487	c.316G>T:p.E106X	stopgain	-	-	-	0.763:D	0	0	1	0	-
ARSA	22:51065757	C	A	NM_000487	c.302G>T:p.G101V	missense	0.0002	-	0.0003	0.818:D	0	0	1	1	P
ARSA	22:51065763	C	T	NM_000487	c.296G>A:p.R99Q	missense	0	0	0	0.204:T	0	1	0	0	US
ARSA	22:51065802	C	T	NM_000487	c.257G>A:p.R86Q	missense	0.0001	0	0	0.804:D	0	0	0	1	P
ARSA	22:51065803	G	A	NM_000487	c.256C>T:p.R86W	missense	-	-	-	0.797:D	1	0	0	0	CIP
ARSA	22:51065990	G	A	NM_000487	c.218C>T:p.P73L	missense	8.7E-05	0	0.001	0.802:D	0	0	0	1	-
ARSA	22:51066102	C	T	NM_000487	c.106G>A:p.G36R	missense	-	-	-	0.777:D	0	0	1	0	-
ARSA	22:51066135	T	C	NM_000487	c.73A>G:p.I25V	missense	0	-	0	0.279:T	0	0	1	0	-
ARSA	22:51066174	C	T	NM_000487	c.34G>A:p.A12T	missense	-	-	-	0.514:T	1	0	0	0	-
ARSA	22:51066201	T	C	NM_000487	c.7A>G:p.M3V	missense	-	-	-	0.550:T	1	0	0	0	-
GLA	X:100652790	AAACATTTTAAA	-	NM_000169	c.1286_1297del:p.L429_*430delinsHFIA	frameshift deletion	-	-	-	-	0	0	1	0	-
GLA	X:100653020	C	T	NM_000169	c.1067G>A:p.R356Q	missense	0	-	-	0.769:D	0	0	0	3	CIP
GLA	X:100653446	C	G	NM_000169	c.911G>C:p.S304T	missense	-	-	-	0.393:T	0	0	0	2	US
GLA	X:100653494	G	A	NM_000169	c.863C>T:p.A288V	missense	-	-	-	0.945:D	0	1	0	0	-
GLA	X:100653810	T	A	NM_000169	c.764A>T:p.D255V	missense	-	-	-	0.555:T	0	0	2	0	-

GLA	X:100655692	A	C	NM_000169	c.601T>G:p.S201A	missense	0.0004	-	0.0003	0.943:D	3	0	2	0	US
GLA	X:100656739	G	A	NM_000169	c.428C>T:p.A143V	missense	-	-	-	0.895:D	0	0	2	0	-
GLA	X:100658972	C	G	NM_000169	c.196G>C:p.E66Q;-	missense	0.0015	0.001	0.0017	0.915:D	6	2	3	1	CIP
GLA	X:100662887	T	C	NM_000169	c.5A>G:p.Q2R	missense	-	-	-	0.188:T	0	0	1	0	p.Q2X
LAMP2	X:119562380	T	C	NM_001122606	c.1195A>G:p.I399V	missense	-	-	-	0.468:T	0	1	0	0	-
LAMP2	X:119565206	T	C	NM_002294	c.1205A>G:p.H402R	missense	-	-	-	0.182:T	0	0	0	1	-
LAMP2	X:119565254	G	C	NM_002294	c.1157C>G:p.A386G	missense	-	-	-	0.181:T	0	0	0	1	-
LAMP2	X:119565269	A	G	NM_002294	c.1142T>C:p.V381A	missense	0.0004	0.001	0.0005	0.904:D	3	1	3	1	CIP
LAMP2	X:119573071	C	T	NM_013995	c.1171G>A:p.V391I	missense	0.0003	0.001	0.0003	0.232:T	0	2	0	1	CIP
LAMP2	X:119573116	G	T	NM_013995	c.1126C>A:p.L376I	missense	0.0002	-	0.0002	0.148:T	5	1	0	0	LB
LAMP2	X:119580225	A	G	NM_013995	c.799T>C:p.S267P	missense	7.77E-05	-	-	0.360:T	0	1	0	1	-
LAMP2	X:119581764	C	A	NM_013995	c.673G>T:p.V225F	missense	-	-	-	0.551:T	2	0	0	0	-
LAMP2	X:119589250	G	A	NM_013995	c.359C>T:p.T120I	missense	-	-	-	0.219:T	1	0	0	0	-
LAMP2	X:119589256	T	C	NM_013995	c.353A>G:p.Y118C	missense	0	-	0	0.950:D	0	0	0	1	-
LAMP2	X:119589398	T	C	NM_013995	c.211A>G:p.T71A	missense	7.8E-05	-	0	0.003:T	0	0	0	1	-
LAMP2	X:119590510	G	C	NM_013995	c.179C>G:p.T60S	missense	-	-	-	0.005:T	0	0	2	0	-
LAMP2	X:119590532	G	A	NM_013995	c.157C>T:p.R53C	missense	0	0	0	0.322:T	0	2	0	0	CIP
LAMP2	X:119590573	G	A	NM_013995	c.116C>T:p.A39V	missense	-	-	-	0.171:T	0	0	2	0	-
LAMP2	X:119602978	A	G	NM_013995	c.47T>C:p.L16P	missense	-	-	-	0.773:D	2	0	0	0	-
IDS	X:148564281	G	C	NM_000202	c.1649C>G:p.P550R	missense	-	-	-	0.623:T	1	2	1	0	-
IDS	X:148564326	A	G	NM_000202	c.1604T>C:p.M535T	missense	0.0002	-	0.0003	0.468:T	0	0	1	1	-
IDS	X:148564431	G	A	NM_000202	c.1499C>T:p.T500I	missense	0.0006	0.0028	0.0012	0.919:D	10	5	0	2	B
IDS	X:148564444	C	T	NM_000202	c.1486G>A:p.D496N	missense	-	-	-	0.438:T	0	0	0	1	-
IDS	X:148564452	C	T	NM_000202	c.1478G>A:p.R493H	missense	0.0002	-	0.0003	0.907:D	0	0	2	0	US
IDS	X:148564558	G	A	NM_000202	c.1372C>T:p.R458C	missense	0.0002	-	0	0.546:T	2	0	3	0	-
IDS	X:148564578	G	A	NM_000202	c.1352C>T:p.P451L	missense	7.77E-05	0	0.0002	0.366:T	1	0	0	0	-
IDS	X:148564660	C	T	NM_000202	c.1270G>A:p.V424I	missense	0.0004	0	0.0005	0.305:T	0	0	1	1	US
IDS	X:148564669	G	A	NM_000202	c.1261C>T:p.R421C	missense	0	-	0	0.538:T	2	0	2	0	-
IDS	X:148564678	C	T	NM_000202	c.1252G>A:p.V418I	missense	-	-	-	0.382:T	1	0	0	1	-
IDS	X:148564708	G	C	NM_000202	c.1222C>G:p.P408A	missense	0.0002	-	-	0.550:T	2	0	0	0	-
IDS	X:148568524	G	A	NM_000202	c.1112C>T:p.P371L	missense	0.0002	-	0	0.565:T	2	0	1	1	LB

IDS	X:148568536	G	A	NM_000202	c.1100C>T:p.T367M	missense	0.0002	0.001	0	0.876:D	0	1	0	0	p.T367S
IDS	X:148571913	C	T	NM_000202	c.938G>A:p.R313H	missense	-	-	-	0.258:T	0	2	0	0	-
IDS	X:148571914	G	A	NM_000202	c.937C>T:p.R313C	missense	0	-	0	0.818:D	0	2	0	2	B
IDS	X:148577905	G	A	NM_000202	c.851C>T:p.P284L	missense	0.0012	0.001	0.0015	0.899:D	3	4	3	4	B
IDS	X:148577975	G	C	NM_000202	c.781C>G:p.P261A	missense	0.001	0.001	0.0011	0.723:D	0	3	1	1	B/LB
IDS	X:148577999	G	A	NM_000202	c.757C>T:p.P253S	missense	-	-	-	0.556:T	0	0	1	0	-
IDS	X:148578019	T	A	NM_000202	c.737A>T:p.N246I	missense	0.0002	-	0.0002	0.746:D	4	0	1	0	-
IDS	X:148579730	T	C	NM_000202	c.616A>G:p.I206V	missense	-	-	-	0.407:T	0	0	0	1	-
IDS	X:148584959	G	A	NM_000202	c.301C>T:p.R101C	missense	0.0011	0.001	0.0008	0.961:D	0	0	2	1	CIP
IDS	X:148585712	A	C	NM_000202	c.215T>G:p.L72R	missense	-	-	-	0.868:D	0	0	0	2	-
IDS	X:148585797	C	T	NM_000202	c.130G>A:p.V44M	missense	-	-	-	0.739:D	0	1	3	1	-
IDS	X:148585808	A	C	NM_000202	c.119T>G:p.L40R	missense	-	-	-	0.993:D	0	0	2	0	-
IDS	X:148586571	T	A	NM_000202	c.97A>T:p.T33S	missense	0.0014	0	0.0015	0.310:T	1	0	0	0	LB
IDS	X:148586576	T	C	NM_000202	c.92A>G:p.N31S	missense	0.0002	-	-	0.257:T	3	0	1	0	-

a. Variants minor allele frequencies from gnomAD_genome_EAS, gnomAD_exome_EAS and ExAC_EAS.

b. (The score of prediction software):(predictive results); D = Damaging; T = Tolerate.

c. variant were annotated by reported pathogenicity for LSDs, based on ClinVar (https://www.ncbi.nlm.nih.gov/clinvar/), as follows: B, benign; LB, likely benign; P, pathogenic; LP, likely pathogenic; CIP, conflicting interpretations of pathogenicity; US, uncertain significance. In some cases, it is noted if there is an alternative variant (p.X###Y) is established to be pathogenic at the same position, and other variants without documentation in ClinVar are indicated with a dash (-).

Supplementary Table 4. Rare damaging homozygous variants of LSD genes identified in patients from WES cohort and WGS cohort

Gene	Position (hg19)	Ref	Alt	NM number	AAChange	Consequence [a]	gnomAD_exome_EAS [b]	gnomAD_genome_EAS [b]	ExAC_EAS [b]	ReVe [c]	Sample number
GBA	chr1:155207203	T	C	NM_000157	c.928A>G:p.S310G	missense	0.0002	0.0006	0	0.921:D	1
HPS3	chr3:148876472	C	T	NM_032383	c.1711C>T:p.H571Y	missense	0.0053	0.0031	0.0069	0.733:D	2
SMPD1	chr11:6413290	C	G	NM_000543	c.995C>G:p.P332R	missense	0.0056	0.0012	0.0052	0.892:D	2
GLB1	chr3:33038569	T	A	NM_000404	c.2002A>T:p.K668X	stopgain	0.0002	0.0012	0.0001	0.331:T	1
HPS3	chr3:148858972	A	G	NM_032383	c.881A>G:p.Y294C	missense	0	0.0006	-	0.887:D	1
IDUA	chr4:996555	G	C	NM_000203	c.1225G>C:p.G409R	missense	0.0147	0.0204	0.0231	0.703:D	2
KCTD7	chr7:66098374	A	G	NM_153033	c.257A>G:p.Y86C	missense	0	-	0	0.729:D	1
LIPA	chr10:90974776	T	C	NM_000235	c.1009A>G:p.T337A	missense	0.0098	0.0099	0.0094	0.755:D	1
GNPTAB	chr12:102141003	C	T	NM_024312	c.3710G>A:p.R1237Q	missense	0.0025	0	0.0024	0.848:D	1
GNPTAB	chr12:102158945	G	T	NM_024312	c.1750C>A:p.P584T	missense	0.0083	0.0074	0.0089	0.793:D	1

a. Variants minor allele frequencies from gnomAD_genome_EAS, gnomAD_exome_EAS and ExAC_EAS.

b. (The score of prediction software):(predictive results); D = Damaging; T = Tolerate.
